# Genomic insights into a diarrheal outbreak in Bangladesh reveal novel ETEC lineages and expansion of CS23 colonization factor

**DOI:** 10.1128/spectrum.03315-24

**Published:** 2025-09-10

**Authors:** Sadia Isfat Ara Rahman, Mohammad Jubair, Marjahan Akhtar, Afroza Akter, Imam Tauheed, Yasmin Ara Begum, Piyash Bhattacharjee, Mokibul Hassan Afrad, Farhana Khanam, Md Taufiqul Islam, Ashraful Islam Khan, Mustafizur Rahman, Edward T. Ryan, James M. Fleckenstein, Taufiqur Rahman Bhuiyan, Nicholas Robert Thomson, Firdausi Qadri, Astrid von Mentzer, Fahima Chowdhury

**Affiliations:** 1 International Centre for Diarrheal Disease Research, Bangladesh (icddr,b) 56291, Dhaka, Bangladesh; 2 Division of Infectious Diseases, Massachusetts General Hospital 2348, Boston, Massachusetts, USA; 3 The Molecular Microbiology and Microbial Pathogenesis Program, Division of Biology and Biomedical Sciences, Washington University in St. Louis 7548, St. Louis, Missouri, USA; 4 Wellcome Sanger Institute, Wellcome Genome Campus 497772 , Hinxton, Cambridge, United Kingdom; 5 Department of Microbiology and Immunology, Sahlgrenska Academy, University of Gothenburg 70712, Gothenburg, Sweden; 6 SciLifeLab, University of Gothenburg 3570, Gothenburg, Sweden; Ludwig-Maximilians-Universitat Munchen Pettenkofer Institute, München, Germany

**Keywords:** diarrhea, whole genome sequencing, enterotoxigenic *E. coli*, colonization factors, toxin profile, Bangladesh

## Abstract

Enterotoxigenic *Escherichia coli* (ETEC), a leading cause of diarrhea, is defined by heat-stable (ST) and/or heat-labile (LT) toxins and associated colonization factors (CFs). However, there is still a knowledge gap in understanding ETEC’s evolution, particularly in endemic regions like Bangladesh. This study investigates the genomic attributes contributing to the rise of ETEC-associated diarrhea in Bangladesh during 2022–2023. Whole genome sequencing of 325 ETEC isolates (2022–2023), compared with historical strains (1980–2021), revealed significant evolutionary changes. Our findings showed a significant shift in ETEC toxin from LT to ST over the period 2013–2023. The most frequent virulence profile during this period was CFA/I + CS21 compared with previous years (1980–2021). The emergence of CS23-positive ETEC was reported for the first time in Bangladesh, which was considered a less common CF in previous studies. Notably, we report the four novel lineages “L26–L29” in this study through phylogenetic analysis, partly encompassing emergent CS23-positive ETEC strains. Additionally, the high prevalence of multi-drug-resistant ETEC strains and the presence of ESBL-CTX-M-resistant gene during 2022–2023 are a matter of great concern, underscoring the need for preventive measures. The switch of distinct toxin and CF combinations, the rapid emergence of CS23, ESBL-CTX-M resistance, and novel lineages may be the reason behind the increased number of ETEC diarrheal cases between 2022 and 2023. These findings highlight the rapid ETEC evolution that underscores the necessity of continued genomic surveillance to track ongoing changes.

**IMPORTANCE:**

This study expands on previous evidence, demonstrating a remarkable genomic diversity in ETEC strains from 2022 to 2023, particularly in virulence factors and AMR genes. The combined findings from these studies will be important for mitigating future diarrheal outbreaks by informing preventive measures, including future vaccine targets, and implementing antibiotic stewardship programs against ETEC infection. Importantly, this research underscores the necessity of continued genomic surveillance to track ongoing changes in ETEC. Such monitoring is essential for understanding the pathogen’s evolving population structure, transmission dynamics, and resistance mechanisms.

## INTRODUCTION

Enterotoxigenic *Escherichia coli* (ETEC) remains a leading cause of moderate-to-severe diarrhea in low- and middle-income countries (LMICs) and is also the primary cause of traveler’s diarrhea (1, 2). Globally, ETEC is responsible for approximately 220 million diarrheal episodes annually, resulting in 18,700–42,000 deaths per year in children under 5 years (3). In a birth cohort study at the International Centre for Diarrheal Disease Research, Bangladesh (icddr,b), 20% of the children between 0 and 2 years of age with diarrhea were reported with an incidence of 0.5 diarrheal episodes per year (1). The systemic surveillance system of icddr,b confirms the presence of ETEC causing diarrhea in 10%–15% of all diarrheal cases annually (4, 5).

ETEC is defined by the presence of heat-labile toxin (LT) and/or heat-stable toxin (ST), comprising STh and STp variants (6), resulting in secretory diarrhea and dehydration (2). Colonization by ETEC bacteria is mediated by colonization factors (CFs), outer membrane structures enabling close interaction with the host intestinal cells. Over 30 distinct CFs have been identified in ETEC, with CFA/I, CS1-CS6, and CS21 being the most prevalent CFs globally (2, 6, 7). The presence of other less prevalent CFs may be linked to specific geographical locations. For example, CS23, first described in an ETEC strain collected from a child with diarrhea in Chile in 2012, has shown surprising prevalence in ETEC from clinical and environmental settings in Bolivia during 2013–2014 (8, 9). Additionally, non-canonical virulence factors such as *EtpA* (extracellular adhesin) and *EatA* (serine protease autotransporter) also play a crucial role in pathogenesis (10).

In 2014, von Mentzer et al. used whole genome sequencing (WGS) and phylogenetic analysis of a collection of 362 global ETEC strains isolated from 1980 to 2011. A total of 21 globally distributed lineages (L1–L21) were described, where L1–L10 were characterized by distinct CFs and enterotoxin profiles (11). In 2017, using the same data set, four more lineages (L22–L25) were described as associated with new O-genotypes (12). Genomic studies have revealed that, alongside lineage diversification, the emergence of multidrug-resistant (MDR) and extended-spectrum β-lactamase (ESBL)-producing extensively drug-resistant (XDR) ETEC strains is largely driven by the indiscriminate use of antibiotics (13–15). As this poses new challenges for clinical treatment and public health, it underscores the need for continued genomic surveillance to track the changes in virulence factors and resistance. Despite these advances in ETEC research, comprehensive genomic analysis of Bangladeshi strains has been limited, with no in-depth studies conducted since 2011 to understand the factors contributing to its increased prominence in recent years. This gap highlights the necessity for genomic studies to fully understand the changing epidemiology of ETEC in Bangladesh.

In 2022, Dhaka, the capital city of Bangladesh, faced a sudden upsurge of diarrheal cases (4). The icddr,b hospital, the world’s largest diarrheal treatment facility, responded to this massive outbreak, receiving a historical record number of over 1,300 patients per day (4, 16). The systemic surveillance system of icddr,b identified *Vibrio cholerae* O1 (*V. cholerae* O1) and ETEC as the most frequently isolated pathogens, highlighting their trends during the two diarrheal peaks of 2022 (January to the 1st week of November) (4). However, the increasing trend of ETEC diarrhea continues in 2023. In the present study, we aimed to unravel the genomic attributes of clinical ETEC isolates collected between 2022 and 2023 by comparing them with historical ETEC isolates from 1980 to 2011. Using comparative analysis of ETEC genomics data from Bangladesh spanning 43 years (1980 until 2023), we described the changing pattern of ETEC toxin types, CFs, lineages, and antimicrobial resistance (AMR) profile. This research is crucial for informing strategies to combat ETEC-associated diarrheal disease, including antibiotic stewardship programs and vaccine development.

## RESULTS

### ETEC contributed as one of the major causes of diarrhea during 2022–2023 in Bangladesh

**Fig 1:**
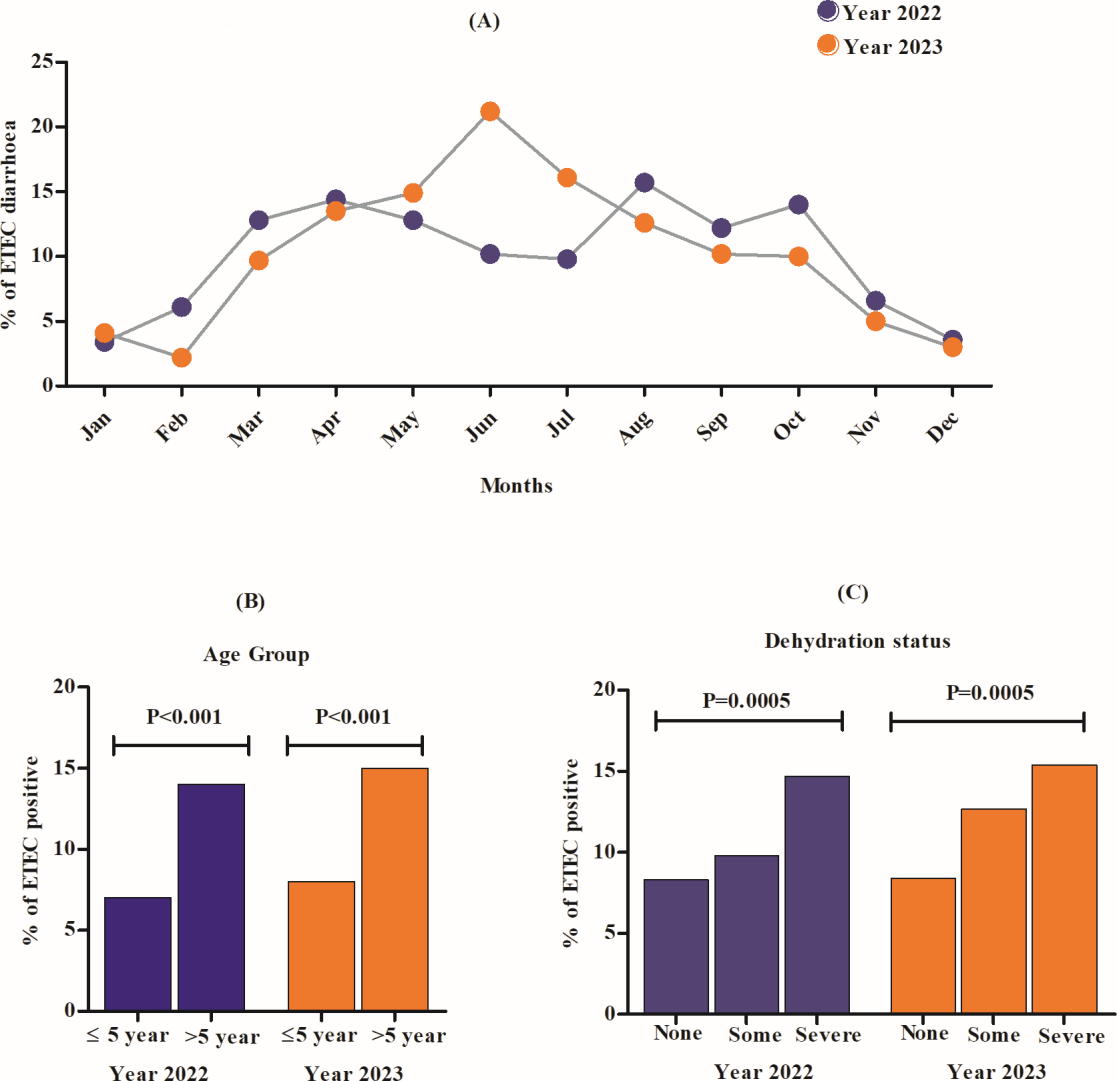
Epidemiological features of ETEC diarrhea in 2022–2023. (**A**) Month wise (%), (**B**) age-group wise (%), and (**C**) dehydration-status wise (%) ETEC cases. A χ^2^ test at 5% level of significance was done to identify significant differences (*P* value).

In 2022, a total of 3,437 diarrheal patients were enrolled, of which 18.1% and 10.5% tested positive for *V. cholerae* O1 and ETEC, respectively. Cholera cases declined drastically from 31.8% in the first peak (March–April) to 11.9% in the second peak period (October–November) in 2022, and the declining pattern continued in 2023 (8.2%). On the other hand, ETEC cases remained as major causative agents of diarrhea in both years (10.5% in 2022 and 10.3% in 2023) (Fig. S1). From Fig. 1A, it was evident that ETEC showed seasonality in 2022, with two peaks between March–May (13.5%) and August–October (14%). Although in 2023, the peak period continued from Mar to Oct (14%), of which the highest isolation rate was seen in June (21.1%). In 2022–2023, ETEC isolation was significantly higher in patients above 5 years of age compared with those under 5 (14.5% vs 7.9%; *P* < 0.0001) (Fig. 1B). Stratification of ETEC cases by dehydration status revealed significantly higher rates (*P* < 0.0001) among severely (14.9%) and moderately (11.2%) dehydrated patients compared with those without dehydration (8.3%) (Fig. 1C).

### ST-only ETEC has become more prevalent in recent years in Bangladesh

**Fig 2:**
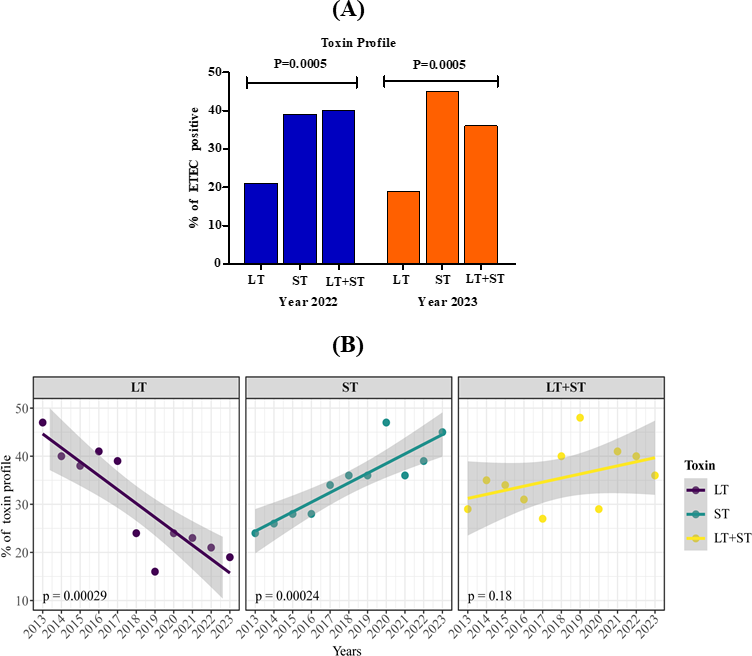
(**A**) Distribution of ETEC toxin (LT, ST, and LT + ST) isolated from diarrheal patients in 2022–2023. (**B**) Trend analysis of ETEC toxin distribution from 2013 to 2023. Each dot represents the percentage of toxins per year. The line in each box shows the year-wise trend of ETEC toxins. ETEC toxins are colored as described in the inset legend. A χ^2^ test at a 5% level of significance was done to identify significant differences (*P* value).

Among the 649 ETEC-positive cases in 2022-2023, it was evident that 41.3% ST toxin (33% STh, 8.3% STp) and 38.5% ST + LT toxin (31.6% LT + STh, 6.6% LT+ STp) were present at a higher frequency compared with LT-positive-only isolates (20.0%) (*P* = 0.0005) (Fig. 2A). To understand if this was a longer-term trend, we examined the pattern of toxin distribution between 2013 and 2023 showing a steady decline of LT-only (*P* = 0.00029) and a significant increase in ST-only positive isolates (*P* = 0.00024). This confirmed a significant shift in toxin type from LT to ST-ETEC over this period (Fig. 2B).

### The emergence of CS23-positive ETEC strains collected from adult patients with severe diarrhea

**Fig 3:**
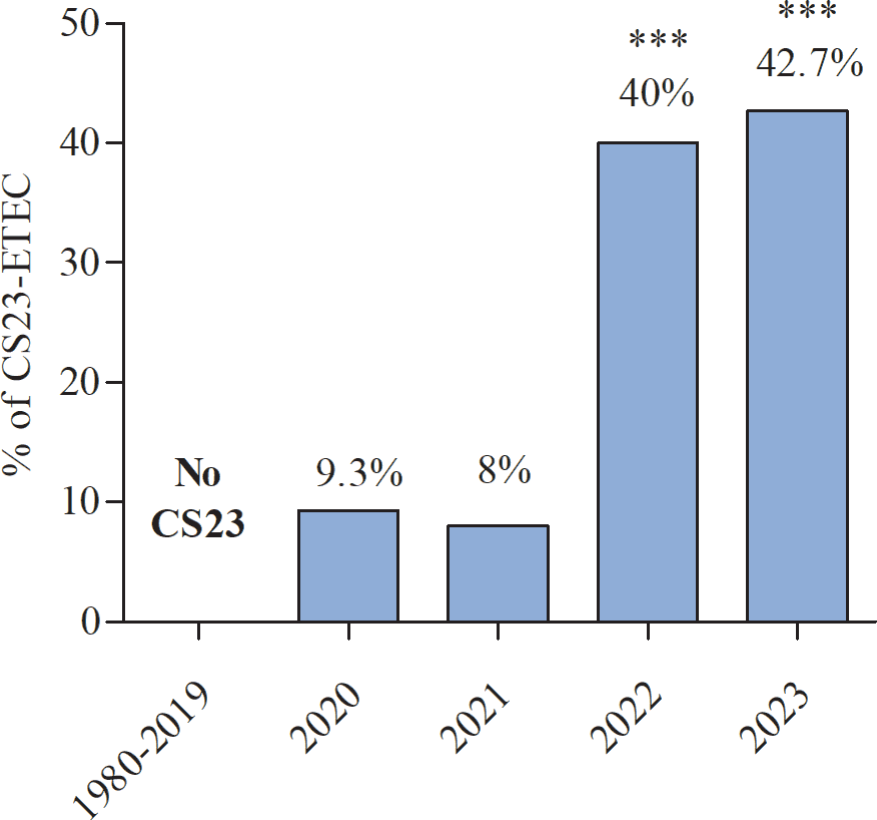
Prevalence of CS23-positive ETEC genome (*n* = 74) from Bangladesh over time. Statistical analysis was performed between 2020 versus other years using the χ^2^ test. ***P < 0.001.

We combined our conventional surveillance approach with a genomic approach to comprehensively study the distribution of CFs in Bangladesh. This analysis used a subset of 325 ETEC genomes, representing the ETEC isolates collected between 2022 and 2023. Although conventional laboratory methods (i.e., dot-blot and PCR) detect only 13 common CFs, genomic analysis enables a more comprehensive identification of CF-positive isolates, including less common CFs. Our bioinformatic analysis revealed 71.4% (232/325) of these ETEC genomes harbored one or more major subunits of CF genes, compared with the conventional laboratory-based methods (42%, 136/325). Genomic data uncovered less prevalent CFs (CS13, CS20, CS23, CS27, and CS28), highlighting the enhanced sensitivity of genomic approaches in uncovering a broader spectrum of CFs present in ETEC strains. We found that CS23-positive ETEC isolates, harboring the major subunit *aalE*, were first observed in Bangladesh in 2020 and have increased significantly, from 9.3% (7/75) in 2020 to 42.7% (32/75) in 2023, *P* < 0.0001 (Fig. 3). Among the 62 CS23-positive ETEC isolated between 2022 and 2023, 59 cases suffered from severe dehydration, and 45 were collected from patients between 21 and 60 years of age (Table S1). Comparing the nucleotide encoding the major subunit in these genomes with the reference CS23 *aalE* gene revealed a level of diversity, as the percentage identity ranged from 79.7% to 99.7%. Due to repetitive or complex regions, the majority of the gene clusters were fragmented across more than one contig, making it difficult to perform a more detailed analysis of the gene cluster. In general, common CFs are expressed in combination with other CFs, but we observed in this study that CS23 was present alone, except in one isolate, which also harbored CS13 *cshE* major subunit. Additionally, we analyzed the plasmid profiles of 836 ETEC genomes to identify patterns associated with different CFs (Table S2). Notably, the *IncFIA* plasmid was predominantly found in CS23-positive isolates (69/74, 92%) but largely absent in isolates expressing more common CFs. Conversely, the *IncB/O/K/Z* plasmid was absent in CS23-positive isolates but identified in other CF-positive isolates. The bandage analysis confirmed that these plasmid replicons were not collocated on the same CFs containing contigs due to limitations in short-read assemblies.

We also conducted comparative genomic analysis to examine the CF patterns with the previous ETEC isolates (1980–2021) and observed that CFA/I + CS21 and CS23 were significantly more prevalent in 2022–2023 (*P* = 0.003 and 0.00, respectively*)*, whereas CS14 and CS17 were significantly higher in the previous period (1980-2021) (*P* = 0.008 and 0.013, respectively) (Table 1). Beyond CFs, we identified genes encoding non-canonical virulence factors among 2022–2023 ETEC isolates: *eatA* (13.8%, 45/325), *etpBAC* (6.5%, 21/325), and both *eatA+ etpBAC* (72/325, 22.2%). In addition, we also observed the coinfection status of our ETEC-positive diarrheal patients. Among the 325 ETEC-positive isolates, 20.3% (66/325) were coinfected with *V. cholerae* O1 serotype. Although investigating CFs, we did not observe any significant or discernible patterns among patients with coinfections. This observation highlights the complex nature of ETEC and *V. cholerae* interactions in diarrheal infections.

**TABLE 1:** Comparative genomic analysis of CF distribution between the study period (2022–2023) and the previous period (1980–2021)

CFs	Study period (2022–2023) *n* (%)	Previous period (1980–2021) *n* (%)	*P^a^* study period (2022–2023)	*P^a^* previous period (1980–2021)
CFA1 + CS21	34 (10.5)	8 (3.8)	0.003*^a^*	0.999
CS1 + CS3 ± CS21	11 (3.4)	12 (5.8)	0.937	0.135
CS2 ± CS3 + CS21	9 (2.8)	8 (3.8)	0.828	0.326
CS1 + CS3 ± CS21 + CS12	1 (0.3)	0	0.610	1.000
CS3 + CS21	0	2 (1.0)	1.000	0.152
CS4 ± CS5 ± CS6	50 (15.4)	42 (20.2)	0.939	0.095
CS6 + PCFO71	1 (0.3)	0	0.610	1.000
CS6 + CS12/CS21	9 (2.8)	3 (1.4)	0.130	0.967
±CS6 + CS8	4 (1.2)	2 (1.0)	0.565	0.753
CS7	6 (1.8)	6 (2.9)	0.861	0.308
CS12 (PCF0159)	9 (2.8)	5 (2.4)	0.516	0.698
CS13	3 (0.9)	0	0.226	1.000
CS13 + CS23	1 (0.3)	0	0.610	1.000
CS13 + CS27	2 (0.6)	0	0.371	1.000
CS14	6 (1.8)	13 (6.3)	0.998	0.008^*a*^
CS15	1 (0.3)	0	0.610	1.000
CS17	4 (1.2)	10 (4.8)	0.997	0.013^*a*^
CS19	0	1 (0.5)	1.000	0.390
CS20	6 (1.8)	2 (1.0)	0.335	0.885
CS21	9 (2.8)	5 (2.4)	0.516	0.698
CS23	61 (18.8)	13 (6.3)	0.000^*a*^	1.000
CS27	2 (0.6)	0	0.371	1.000
CS28	3 (0.9)	0	0.130	1.000

^*a*^ *P*-value calculated using a hypergeometric test.

### Phylogeny analysis revealed four novel lineages “L26–L29”

**Fig 4:**
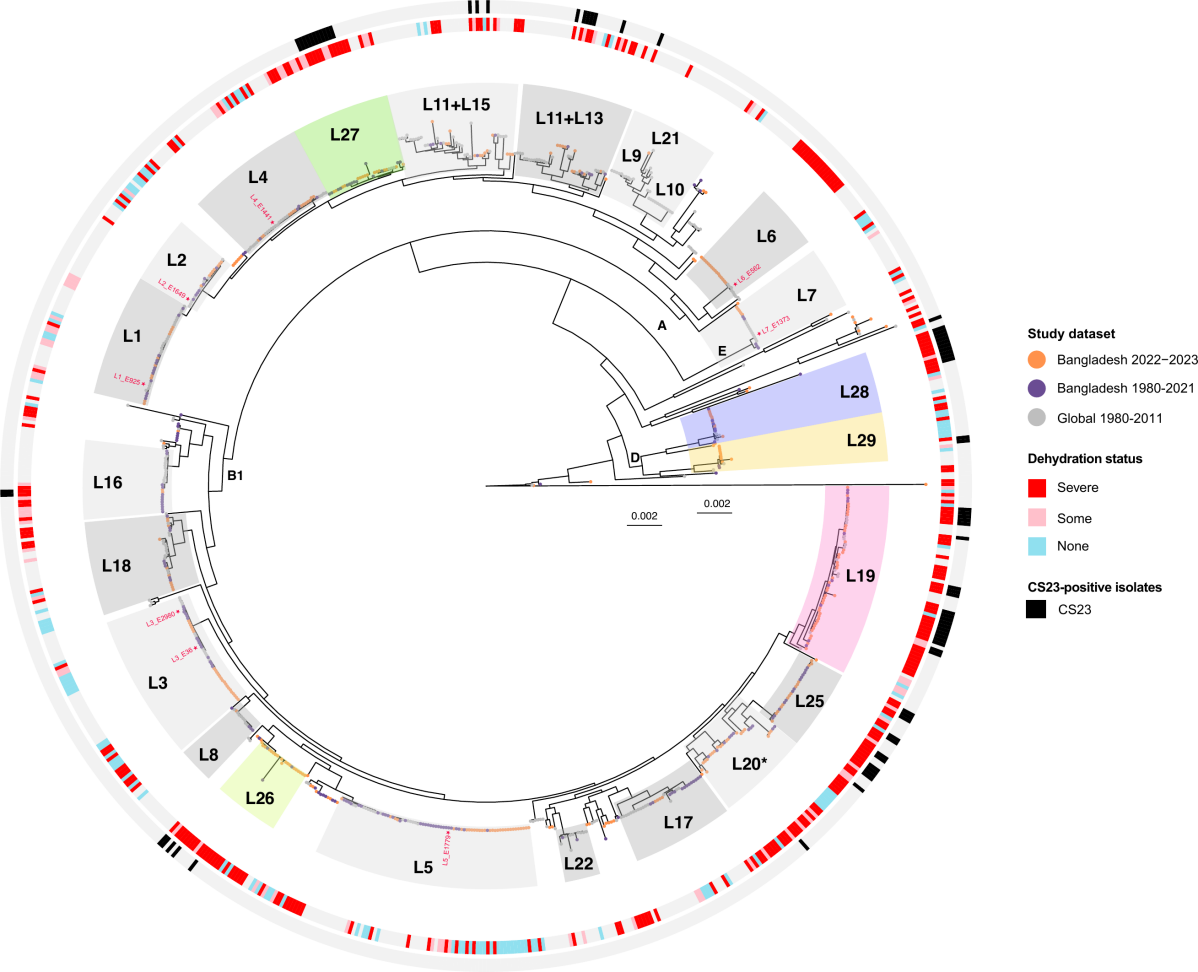
The distribution of CS23-positive ETEC isolates in a maximum likelihood phylogeny of 827 ETEC genomes. The phylogenetic tree is based on core genome analysis. Tree tips are color-coded according to the study data set. Two rings surround the tree: the inner ring indicates the dehydration status of patients from whom ETEC isolates were collected, whereas the outer ring shows the distribution of CS23 across the phylogeny. Previously defined ETEC lineages (L1–L25) are highlighted in grayscale, except for L19. Newly identified lineages (L26–L29) and L19 are highlighted in color. L1–L7 lineage-specific reference strains were highlighted in red text in the phylogeny. The scale bar represents 0.002 substitutions per variable site.

We inferred the core genome phylogeny from 836 ETEC genomes (including 303 global ETEC genomes) to investigate the phylogenetic relationship between Bangladeshi and global ETEC isolates (Fig. 4; Fig. S2). The analysis showed that Bangladeshi isolates were distributed throughout the ETEC global phylogeny and intermingled within previously defined ETEC lineages, except L9, L10, L12, L14, L20, and L24. Notably, the introduction of our 475 Bangladeshi ETEC isolates into the published phylogeny (von Mentzer et al. in 2014) created four novel lineages (denoted as L26, L27, L28, and L29) and also contributed substantially to an expansion of previously described lineages L19, L25. Among the four novel lineages, the L26 and L27 lineages were only Bangladeshi-specific. Moreover, the majority of the Bangladeshi isolates from 2022 to 2023 belonged to L19 (41/325, 12.6%), L20 (32/325, 9.8%), L5 (30/325, 9.2%), and L3 (23/325, 7.1%). Specific combinations of CFs and toxin profiles were also observed in certain lineages: L3 featured CFA/I + CS21 with STh; L5 showed CS5 + CS6 with LT + STh. L19 lineage included 21 isolates with CS23 in combination with LT + STh (*n* = 7), STh (*n* = 5), or LT (*n* = 1). Furthermore, the CS23-positive isolates among the Bangladeshi strains (75/533, 14.1%) were found across 10 ETEC lineages (L11 + L13, L16, L19–L20, and L25–L29), where the majority (62/75, 82.7%) were collected during 2022-2023. Among the Bangladeshi ETEC isolates, 35 did not reside in any of the defined ETEC lineages.

### Increasing antibiotic resistance in ETEC

**Fig 5:**
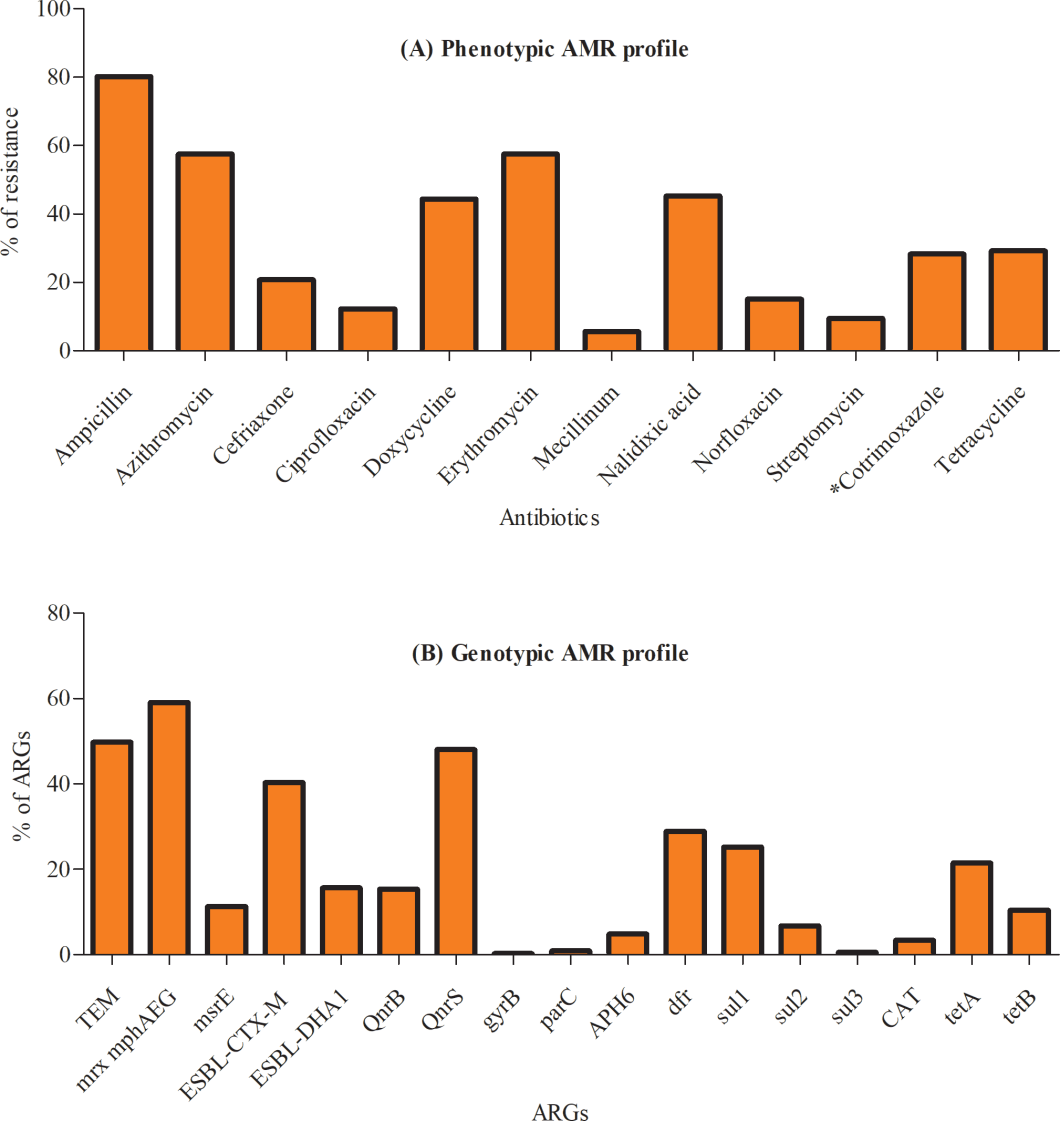
AMR profile (%) of Bangladeshi ETEC isolated in 2022-23. (**A**) Phenotypic resistance profile of ETEC strains (*n* = 106). (**B**) Genotypic resistance profile of ETEC genomes (*n* = 325). *Cotrimoxazole = trimethoprim-sulfonamide. TEM = ampicillin; mrx mphAEG and msrE = macrolide class (azithromycin/erythromycin); ESBL-CTX-M-15 and ESBL-DHA1 = third generation cephalosporin (ceftriaxone/cefixime); QnrB and QnrS = plasmid-mediated fluoroquinolone (nalidixic acid, ciprofloxacin, norfloxacin); gyrB and ParC = chromosomal mediated fluoroquinolone; APH6 = streptomycin A; dfr, sul1, sul2, and sul3 = trimethoprim-sulfonamide (cotrimoxazole); tetA and tetB = tetracycline.

We assayed a subset of the Bangladeshi ETEC strains (*n* = 106) for phenotypic resistance profiling against 12 different antibiotics during 2022–2023 (Fig. 5A). The majority of the ETEC strains (78%) were MDR to more than three different antibiotic classes. Moreover, 56% and 23% of the ETEC strains were resistant to azithromycin (macrolide class) and ceftriaxone (third-generation cephalosporin family), respectively. Furthermore, we also explored the genotypic resistance profile among the 325 Bangladeshi ETEC genomes during 2022–2023 (Fig. 5B) as well as a total of 836 ETEC genomes (Table S3) using bioinformatics analysis. Among the Bangladeshi ETEC genome collection, the *CTX-M15* type of extended-spectrum beta-lactamase (ESBL) and the macrolide-resistant gene were first identified in 2010 and 2011, respectively, and have gradually increased in recent years. In the year 2022–2023, 40% (131/325) and 59% (192/325) Bangladeshi ETEC isolates carried *ESBL-CTX-M15* and *mrx, mphA, mphE,* and *mphG* genes conferring resistance to third-generation cephalosporin and macrolide, respectively. Furthermore, we observed different genetic mechanisms associated with reduced susceptibility to fluoroquinolone. Plasmid-borne quinolone resistance gene *qnrS* that protects DNA gyrase and topoisomerase IV from quinolone action was detected in 48% (156/325) isolates, whereas chromosomal-mediated mutations (*gyrB, parC)* that alter these target enzymes to reduce drug binding were absent in most of the isolates.

## DISCUSSION

Evidence generated from this study suggests that ETEC was a major cause of diarrhea in 2022 and 2023 in Bangladesh after *V. cholerae* O1 (17, 18). The serotype shift of *V. cholerae* O1 from Ogawa to Inaba likely fueled the 2022 Dhaka outbreak, with the epidemic driven by the gradual rise in ETEC cases from 2022 onward (4, 16). Previously, data taken from records of patients attending the icddr,b hospital, peaks in ETEC-attributed diarrhea also occurred in 2018 (14% isolation rate) and during floods in 2004 (18% isolation rate). In general, ETEC diarrhea follows bi-annual peaks, that is, increases twice in the hot summer season and decreases thereafter in the winter (19). This apparent seasonality was also observed in our present analysis, suggesting that rising temperature, humid atmosphere, contaminated drinking water, and the increased bacterial spread in the community by the fecal-oral route might be the major contributors to the diarrheal outbreak. Overall, our data showed that Bangladeshi ETEC isolates fall into multiple lineages, of which lineages L3, L5, L19, and L20 (11) were the most frequent during 2022–2023, and lineages L26–L29 were newly defined in our study. Tracking of these new and previously described lineages in association with the emergence of CS23 will reveal if these lineages will become stable in time, and whether CS23 will become one of the major CF profiles, and if additional new CFs within these lineages can be identified. Furthermore, the toxin profile over the last 10 years (2013–2023) revealed that ST toxin-positive ETEC isolates are replacing LT toxin-positive ETEC in Bangladesh as a major cause of ETEC diarrhea (20, 21). Outside of Bangladesh, ST toxin was also found to be prevalent in Egyptian (22) and Peruvian (23) children. This trend is of great concern for ETEC vaccine development. It is difficult to induce protective immunity against ST toxin-positive ETEC largely due to its small size and cross-reactivity with human guanylin and uroguanylin (24). However, until now, the majority of the ETEC vaccines have focused on LT toxin and the most epidemiologically prevalent CFs to achieve broad protection (25). Here, we highlight the need to reconsider this for future ETEC vaccine development.

Investigating the complete virulence profile from both conventional and genomic approaches, we showed that CFA/I + CS21-positive ETEC associated with STh dominated during 2022–2023 and we detected CS23 for the first time from Bangladesh. Monitoring of CS23 is important for national/regional surveillance and needs to be included in routine laboratory methods to facilitate its detection for future epidemiological studies. Although previous studies did not find any association between CS23 and disease severity, our current investigation reveals that majority of the CS23 positive ETEC diarrheal cases from 2022–2023 involved patients aged 21 to 60 years who presented with severe dehydration. This contrasts with the traditional view of ETEC as primarily a childhood pathogen. Supporting this shift, Akhtar et al. showed that 56% of ETEC cases occured in individuals over 18 years of age, highlighting its significant impact on adult populations (26). Hence, adults are at high risk of severe diarrhea, which may be due to CS23 being a novel circulating CF with low or no homology to more prevalent CFs. During a study in Bolivia, it was shown that CS23 may be transmitted from the environment to clinical settings through the contaminated Choqueyapu River, which was a potential reservoir for CS23 ETEC isolates (9). This finding suggests that CS23-positive ETEC isolates may also be transmitted from environmental settings such as polluted water sources of rivers, wastewater, and contaminated drinking water in Bangladesh. The expansion of existing and new lineages in association with the emergence of CS23 might be one of the possible reasons behind the increased ETEC causing diarrhea in 2022 and 2023. Moreover, the presence of clinical and environmental-associated CS23 ETEC strains emphasizes the need for the implementation of a One Health strategy for the prevention of ETEC infections in the future. A One Health strategy for ETEC seeks to sustainably reduce infections by addressing human, animal, and environmental sources rather than focusing on human cases.

In addition to the changes in the lineages and their virulence gene profiles, it was also evident from our data that a significantly high proportion of Bangladeshi ETEC isolated from 2022 to 2023 were MDR. This is consistent with published data of diarrheal patients attending the Mathbaria Health Complex in 2014–2016, showing that approximately 60% of the ETEC strains were MDR (14). Moreover, around 88% of ETEC strains isolated between 2005 and 2009 from the 2% surveillance system at icddr,b were MDR against nalidixic acid-ciprofloxacin, including reduced susceptibility to azithromycin and ceftriaxone (13). In Bangladesh, the widespread use of antibiotics without prescription and AMR testing of strains is a very common practice. The overuse of azithromycin and third-generation cephalosporins for the treatment of diarrheal and respiratory diseases in recent years, including during the COVID-19 pandemic, probably contributed to increased acquisition of *mrx, mphA, mphE,* and *mphG* genes that confer resistance to macrolide class (azithromycin and erythromycin) and extended-spectrum beta-lactamases ESBL resistance gene *blaCTX-M15* seen in 2022–2023.

This study has some limitations. First, there is a paucity of ETEC genome data available globally, and hence, we were limited in our ability to compare the patterns of disease seen in Bangladesh more broadly. All the 303 published global ETEC genomes used in this study were collected from 1983 to 2008. Second, we selected 325 ETEC strains from the total number of 626 ETEC-positive patients collected between January 2022 and October 2023 based on the month-wise distribution of isolates. In addition, due to funding constraints, we could only sequence 150 ETEC strains from the years 2012 to 2021. Finally, short-read sequencing limits the ability to accurately resolve the plasmid structures and determine the co-localization of CFs and ARGs. Future studies should employ long-read or hybrid sequencing approaches to overcome these challenges and achieve more comprehensive plasmid reconstructions. However, so far, this is the largest collection of ETEC genomic data from Bangladesh that forms an important basis for future genomic analysis of this important pathogen.

Finally, it can be concluded that observed genetic changes, including shifting of toxin, prevalence of CS23, and high frequency of azithromycin, ESBL-CTX-M15 resistance, are possibly associated with the sudden increase in number of cases in 2022 and 2023. This data shed light on the feasibility of large-scale genomic epidemiology as an important public health tool for detailed molecular characterization, informing vaccine development strategies, and controlling ETEC diarrheal cases.

## MATERIALS AND METHODS

### Study design

We utilized our ongoing 2% diarrheal systematic surveillance system operated by icddr,b Dhaka hospital, in which stool samples are collected from every 50th diarrheal patient seeking hospital care for microbiological screening of enteric pathogens (*V. cholerae,* ETEC, *Salmonella* spp., *Shigella* spp.). Diarrhea was defined as either (i) loose or liquid stools ≥ 3 times; (ii) loose or liquid stools causing dehydration <3 times; or (iii) at least one bloody loose stool in the last 24 h. Severe diarrhea was defined according to the World Health Organization (WHO) as episodes of diarrhea with fever, vomiting, requiring intravenous rehydration, or the need for hospitalization (5). Socio-demographic and clinical information was collected from the legal guardians of child participants and from adult participants. We integrated the epidemiological data from the icddr,b hospital-based Diarrheal Disease Surveillance System, with the largest collection of 475 Bangladeshi ETEC sequenced in this study from 2012 to 2023.

### Microbiological testing and detection of ETEC toxins and CFs

Stool samples were cultured on MacConkey agar and incubated overnight at 37°C. After overnight incubation, six lactose-fermenting colonies from the MacConkey agar plate were pooled and tested by a multiplex PCR for the presence of ETEC toxin genes encoding for LT and ST (STh, STp) (5, 27). Next, each of the colonies from positive samples was tested individually using multiplex PCR for ETEC strain confirmation and then preserved in 80% colonization factor antigen (CFA) broth with 20% glycerol at −70°C for further studies.

Thirteen common CFs of ETEC were identified by dot-blot immunoassay and/or multiplex PCR. For the dot-blot assay, ETEC colonies were plated on CFA agar with and without bile salts and tested for the expression of 12 different common CFs (CFA/I, CS1, CS2, CS3, CS4, CS5, CS6, CS7, CS8, CS12, CS14, and CS17) using CF-specific monoclonal antibodies. For CS21 detection, strains were additionally cultured on a trypticase soy agar (TSA) plate (5, 13, 27). For multiplex PCR, CFs were detected by different pairs of primers targeting genes specific for the same set of 13 CFs used for the dot-blot assay (28).

### Phenotypic antimicrobial susceptibility test

Every fifth ETEC-positive strain (*n* = 106) isolated during 2022–2023 was included for antibiotic susceptibility testing by the disk diffusion method using a panel of commercially available antibiotic discs (Oxoid, Basingstoke, United Kingdom). The zone size (in mm) of inhibition was measured by following the guidelines of the Clinical and Laboratory Standards Institute (CLSI). *E. coli* ATCC 25922, susceptible to all antimicrobials, was used as a control strain (13).

### ETEC strain and sequence data for genomic analysis

In total, 836 ETEC genomes collected across 43 years (1980–2023) were utilized for downstream genomic analysis. The metadata for each isolate is summarized in Table S1. These strains were categorized throughout this study as follows:

Between January 2022 and December 2023, a total of 649 hospitalized diarrheal patients were found positive for ETEC. Around half of the PCR-positive ETEC strains (*n* = 325) covering each month from January 2022 to October 2023 were included for genomics analysis. These 325 ETEC genomes from Bangladesh were considered herein as a representative collection of the “Bangladesh 2022–2023.”

For comparative analysis, 150 stored ETEC strains collected between 2012 and 2021 from icddr,b surveillance system (15 ETEC strains per year) and 58 previously published sequenced ETEC genomes collected between 1980 and 2011 were also included in this analysis. These 208 ETEC genomes from Bangladesh were considered herein as a representative collection of ETEC strains from the “Bangladesh 1980–2021” (11). To provide a global context for the Bangladeshi ETEC genome analysis, 303 published global ETEC genomes collected between 1983 and 2008 were included, which were considered the “global 1983–2008” (11).

### DNA extraction and whole genome sequencing

Genomic DNA was extracted from the selected ETEC strains (*n* = 475) using the DNAeasy Blood and Tissue kit (250). WGS was carried out at icddr,b Genome Center (iGC) using the Illumina NextSeq 500 system (Illumina, San Diego, CA, USA). Index-tagged paired-end sequencing libraries were prepared from 300 to 350 ng of pure genomic DNA to generate 150 bp paired-end short sequence reads. The quality of the raw read was assessed using the FastQC tool (http://www.bioinformatics.babraham.ac.uk/projects/fastqc). Adapter and other Illumina-specific sequences were trimmed using Trimmomatic v0.39 (29).

### Assembly, annotation, core gene, and SNP detection

Trimmed sequence reads were *de novo* assembled using the Shovill assembler (https://github.com/tseemann/shovill) and annotated using Bakta v1.8.2 (30). A total of 2,711 core genes (defined as present in ≥95% of all 836 ETEC genomes) were identified using Roary v3.13 (31). The core-gene alignment was created by Roary using the MAFFT aligner, and single-nucleotide polymorphism (SNP) sites were extracted using SNP-sites v2.5.1 (32).

### Phylogenetic analysis

A maximum-likelihood (ML) phylogenetic tree was inferred from the core-gene SNP alignment by IQTree v2.2.0.3 (33) using the GTR + F + I model and 1,000 ultra-fast bootstraps (-bb), with the constant sites 26,100, 23,316, 27,024, and 24,729 for linear scaling of the branch lengths (-fconst). The resulting phylogeny was visualized and annotated using R v4.4.0 (see R code for specific packages).

We performed a hierarchical Bayesian analysis of population structure (BAPS) using RhierBAPS v1.1.3 (34). Our analysis estimated 9 and 42 clusters at levels 1 and 2 of the hierarchy, respectively. Although 25 ETEC lineages (L1–L25) were previously reported (11, 12), our expanded data set led to some reorganization of clusters. We used both core genome-based phylogeny and updated BAPS output to define lineages of Bangladeshi ETEC genomes. New ETEC lineages were identified based on phylogenetic tree topology and revised BAPS clustering results, reflecting the impact of the expanded data set on population structure analysis.

### Genomic profiling of virulence factors, antimicrobial resistance genes (ARGs), and plasmid replicons

ABRicate v1.0.1 (https://github.com/tseemann/ABRicate) was used to identify virulence factors, including CFs, noncanonical antigens (EatA, EtpBAC), and toxins, using a custom database (https://github.com/avonm/etec_vir_db). The presence of the structural major subunit for each CF was used to define the CF-positive for each strain. The gene encoding the major subunits in all CS23-positive genomes was extracted and compared with the reference *aalE* (accession number: JQ434477.1) using BLASTn (35). ARGs and plasmid replicons were detected using ARIBAv3.6.0 in conjunction with the comprehensive antibiotic resistance database (CARD) (36, 37) and PlasmidFinder database (38). The assembly graphs were visualized using the Bandage v0.8.1 tool to explore the connection between contigs and assess whether ARGs and CFs resided on the same plasmid contigs (39).

### Statistical analysis

Descriptive statistics for sociodemographic, clinical characteristics, and resistance profiles were presented as frequencies and percentages. χ^2^ tests compared ETEC infection across age groups, dehydration status, and toxin profile. A hypergeometric test was used to compare the CFs distribution between the study period and the previous time period. All the statistical tests were conducted at a 5% significance level. Statistical analysis and data visualization were performed by using R v4.4.0 and GraphPad Prism v5.0 software.

## Data Availability

Sequence data for 475 ETEC isolates of this study have been submitted to the Sequence Read Archive (SRA) under the BioProject number PRJNA1130082 (SRR12345678–SRR12345690). All the accession numbers of each read pair are available at https://www.ncbi.nlm.nih.gov/bioproject/1130082. The detailed metadata of a total of 836 ETEC isolates, including published ETEC isolates (11), are also available in Table S1. Additionally, SRR accession numbers for the 475 ETEC reads generated in this study (SRR29657530 to SRR29658004) are included in the Table S1 for clarity and ease of access. The code used for generating plots in this study are available in our GitHub repository at https://github.com/vonMentzer-Group/ETEC_Upsurge_Bangladesh_2022-2023.

## SUPPLEMENTAL MATERIAL

Fig. S1 spectrum.03315-24-s0001.pdf Month-wise ETEC and *V. cholerae* O1 distribution in (A) 2022 and (B) 2023.

Fig. S2 spectrum.03315-24-s0002.pdf Core-genomes SNP-based phylogenetic maximum-likelihood tree.

Table S1 spectrum.03315-24-s0003.pdf Metadata of a total of 836 ETEC isolates, including 475 Bangladeshi ETEC isolates of this study.

Table S2 spectrum.03315-24-s0004.pdf Data set of plasmid profile for a total of 836 ETEC isolates, including 475 Bangladeshi ETEC isolates of this study.

Table S3 spectrum.03315-24-s0005.pdf Data set of AMR genes for a total of 836 ETEC isolates, including 475 Bangladeshi ETEC isolates of this study.

ASM does not own the copyrights to Supplemental Material that may be linked to, or accessed through, an article. The authors have granted ASM a non-exclusive, world-wide license to publish the Supplemental Material files. Please contact the corresponding author directly for reuse.
