# Supplementary material for: Genomic insights into a diarrheal outbreak in Bangladesh reveal novel ETEC lineages and expansion of CS23 colonization factor
Source: Microbiol Spectr. 2025 Sep 10;13(10):e03315-24. doi: 10.1128/spectrum.03315-24 (PMC12502627; doi:10.1128/spectrum.03315-24)
Supplement: Fig. S1 — Month-wise ETEC and V. cholerae O1 distribution in (A) 2022 and (B) 2023. [file spectrum.03315-24-s0001.pdf]

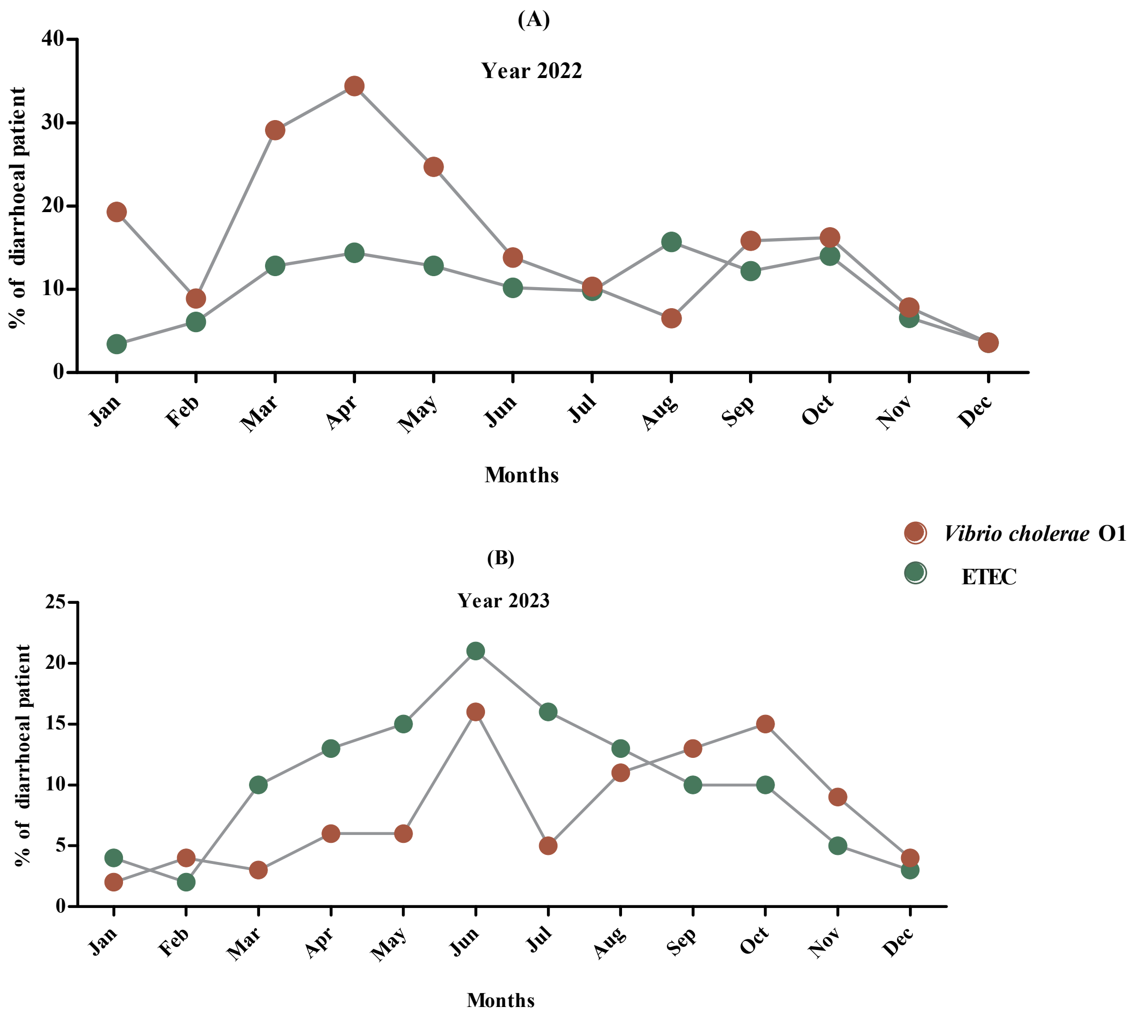

Figure S1: Month-wise ETEC and *V. cholerae* O1 distribution in (A) 2022 and (B) 2023. Each dot represents the percentage of ETEC (green coloured) and *V.cholerae* O1 (red coloured) positive diarrhoeal patients among the total enrolled diarrhoeal patients for each month
