# Supplementary material for: Genomic insights into a diarrheal outbreak in Bangladesh reveal novel ETEC lineages and expansion of CS23 colonization factor
Source: Microbiol Spectr. 2025 Sep 10;13(10):e03315-24. doi: 10.1128/spectrum.03315-24 (PMC12502627; doi:10.1128/spectrum.03315-24)
Supplement: Fig. S2 — Core-genomes SNP-based phylogenetic maximum-likelihood tree. [file spectrum.03315-24-s0002.pdf]

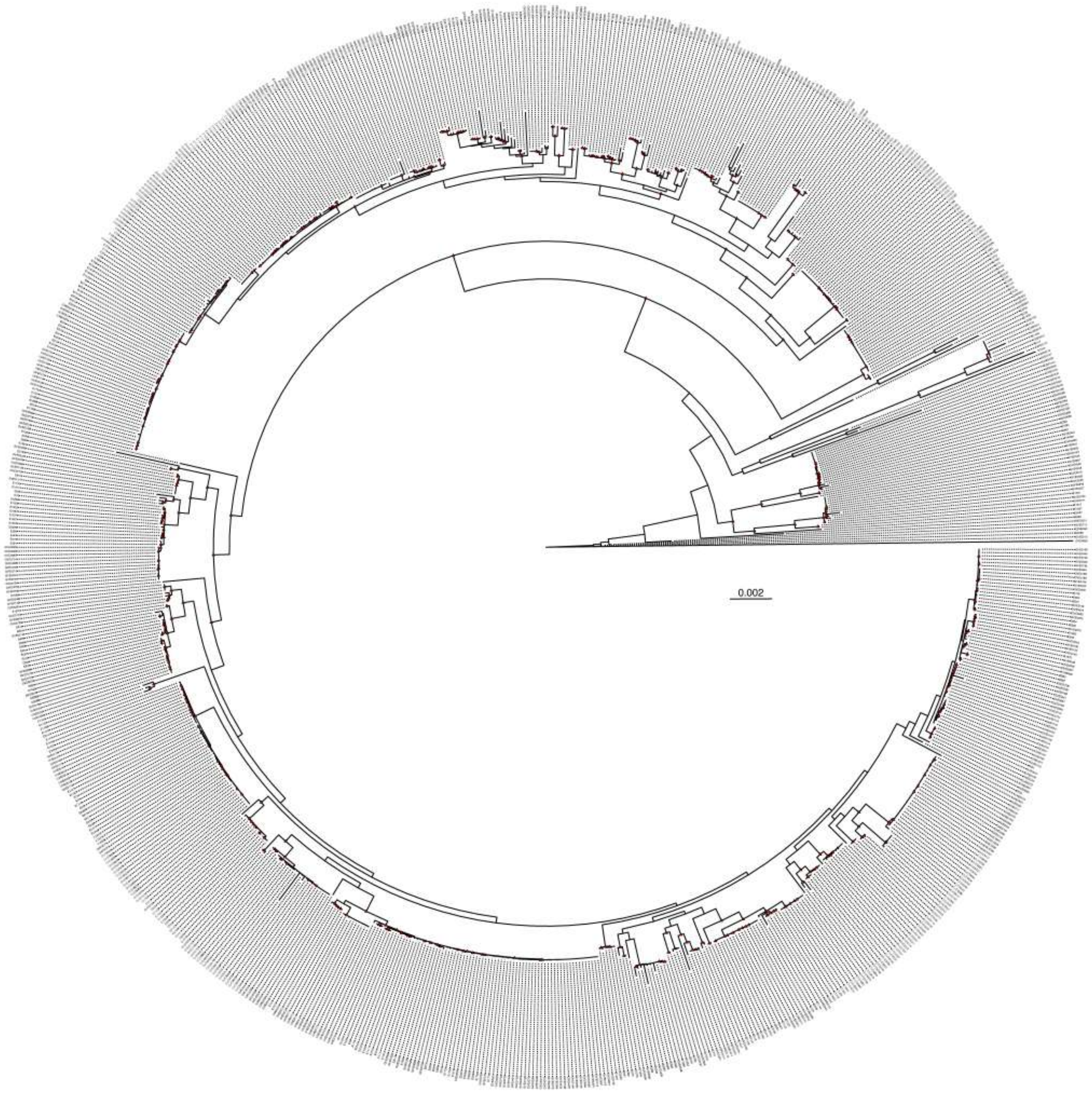

Figure S2. Core-genomes SNP-based phylogenetic maximum-likelihood tree. Nodes with a bootstrap values above 95 are indicated with a red triangle. The StrainID are added to the tips. The scale bar represents 0.002 substitutions per variable sites.
