# Supplementary material for: Genomic insights into a diarrheal outbreak in Bangladesh reveal novel ETEC lineages and expansion of CS23 colonization factor
Source: Microbiol Spectr. 2025 Sep 10;13(10):e03315-24. doi: 10.1128/spectrum.03315-24 (PMC12502627; doi:10.1128/spectrum.03315-24)
Supplement: Table S1 — Metadata of a total of 836 ETEC isolates, including 475 Bangladeshi ETEC isolates of this study. [file spectrum.03315-24-s0003.pdf]

Table S1: Metadata of a total of 836 ETEC isolates including 475 Bangladeshi ETEC isolates of this study

StrainID	Biosample Accession number	Run Accession number	Study dataset	Year	Country	Age (in year)	Age (in month)	Age (in day)	Dehydration	VC culture result	xin (from PC	Simplified_ Toxin_profile	CF profile (Dotblot/PCR)	CF profile(Genomics)	etp,eat (Genomics)	Phylogroup	MLST	Lineage	BAPS.level.1	BAPS.level.2
GVE0025	SAMN42182163	SRR29657865	Bangladesh 2022-2023 (This study)	2023	Bangladesh	0	6	15	Some	Negative	LT+STh	LT+ST	CS1+CS3+CS21	CS1+CS3+CS21	eatA,etpBC	A	-	L1	6	26
GVE0026	SAMN42182164	SRR29657864	Bangladesh 2022-2023 (This study)	2023	Bangladesh	45	0	0	Severe	Negative	STh	ST	CFA/I+CS21	CFA/I+CS21	eatA,etpBC	B1	-	L3	4	12
GVE0027	SAMN42182165	SRR29657817	Bangladesh 2022-2023 (This study)	2023	Bangladesh	50	0	0	Severe	Negative	LT+STh	LT+ST	Negative	CFnegative	Negative	B1	108	L26	4	19
GVE0028	SAMN42182166	SRR29657770	Bangladesh 2022-2023 (This study)	2023	Bangladesh	57	0	0	None	Negative	STp	ST	CS6	CS6	Negative	E	1010	L7	1	1
GVE0029	SAMN42182167	SRR29657755	Bangladesh 2022-2023 (This study)	2023	Bangladesh	45	0	0	Severe	Negative	STh	ST	CFA/I+CS21	CFA/I+CS21	eatA,etpBC	A	-	L6	5	22
GVE0030	SAMN42182168	SRR29657920	Bangladesh 2022-2023 (This study)	2023	Bangladesh	36	0	0	None	Negative	STh	ST	CS6	CS6+CS21	Negative	A	155	L4	6	28
GVE0031	SAMN42182169	SRR29657909	Bangladesh 2022-2023 (This study)	2023	Bangladesh	27	0	0	Severe	Negative	LT+STh	LT+ST	Negative	CFnegative	Negative	B1	632	L27	4	20
GVE0032	SAMN42182170	SRR29657898	Bangladesh 2022-2023 (This study)	2023	Bangladesh	0	6	1	None	Negative	LT	LT	CS7	CS7	eatA,etpABC	B1	-	L3	4	12
GVE0033	SAMN42182171	SRR29657887	Bangladesh 2022-2023 (This study)	2023	Bangladesh	0	11	0	Severe	Negative	LT	LT	Negative	CFnegative	Negative	A	-	L25	6	29
GVE0034	SAMN42182172	SRR29657876	Bangladesh 2022-2023 (This study)	2023	Bangladesh	1	5	8	Severe	Negative	STh	ST	Negative	CFnegative	Negative	B1	-	L20	4	14
GVE0035	SAMN42182173	SRR29657863	Bangladesh 2022-2023 (This study)	2023	Bangladesh	0	7	11	Severe	Negative	LT+STh	LT+ST	Negative	CFnegative	Negative	D	511	NA	8	38
GVE0036	SAMN42182174	SRR29657852	Bangladesh 2022-2023 (This study)	2023	Bangladesh	60	0	0	Some	Negative	STh	ST	CS14	CS14	etpBC	B1	87	L19	4	15
GVE0037	SAMN42182175	SRR29657841	Bangladesh 2022-2023 (This study)	2023	Bangladesh	26	0	0	None	Negative	LT	LT	CS7	CS7	eatA,etpABC	B1	-	L3	4	12
GVE0038	SAMN42182176	SRR29657734	Bangladesh 2022-2023 (This study)	2023	Bangladesh	1	7	0	Severe	Negative	STh	ST	CFA/I+CS21	CFA/I+CS21	eatA,etpBC	B1	-	L3	4	12
GVE0039	SAMN42182177	SRR29657723	Bangladesh 2022-2023 (This study)	2023	Bangladesh	19	0	0	Severe	Negative	STp	ST	Negative	F41a	Negative	B1	-	L20	4	14
GVE0040	SAMN42182178	SRR29657712	Bangladesh 2022-2023 (This study)	2023	Bangladesh	60	0	0	Severe	Negative	STh	ST	CFA/I+CS21	CFA/I+CS21	eatA	A	-	L6	5	22
GVE0041	SAMN42182179	SRR29657701	Bangladesh 2022-2023 (This study)	2023	Bangladesh	49	2	29	Severe	Negative	LT+STh	LT+ST	Negative	CFnegative	Negative	A	-	L25	6	29
GVE0042	SAMN42182180	SRR29657690	Bangladesh 2022-2023 (This study)	2023	Bangladesh	0	11	0	Some	Negative	LT+STh	LT+ST	CS1+CS3+CS21	CS1+CS3+CS21	eatA,etpBC	A	215	L1	6	26
GVE0043	SAMN42182181	SRR29657679	Bangladesh 2022-2023 (This study)	2023	Bangladesh	44	0	0	Severe	Negative	STh	ST	CFA/I+CS21	CFA/I+CS21	eatA,etpBC	A	-	L6	5	22
GVE0044	SAMN42182182	SRR29657828	Bangladesh 2022-2023 (This study)	2023	Bangladesh	0	9	0	None	Negative	STh	ST	CS21	CS21	eatA,etpBC	A	843	NA	6	31
GVE0045	SAMN42182183	SRR29657816	Bangladesh 2022-2023 (This study)	2023	Bangladesh	23	0	0	Severe	Negative	STh	ST	Negative	CFnegative	etpC	A	-	L12+L14	2	7
GVE0046	SAMN42182184	SRR29657805	Bangladesh 2022-2023 (This study)	2023	Bangladesh	0	3	0	Severe	Negative	STh	ST	CFA/I+CS21	CFA/I+CS21	eatA,etpBC	A	-	L6	5	22
GVE0047	SAMN42182185	SRR29657538	Bangladesh 2022-2023 (This study)	2023	Bangladesh	32	0	0	Some	Negative	LT+STh	LT+ST	CS1+CS3+CS21	CS1+CS3+CS21	eatA,etpBC	A	-	L1	6	26
GVE0048	SAMN42182186	SRR29658002	Bangladesh 2022-2023 (This study)	2023	Bangladesh	32	0	0	Severe	Negative	STh	ST	CFA/I+CS21	CFA/I+CS21	eatA,etpBC	B1	-	L3	4	12
GVE0049	SAMN42182187	SRR29657991	Bangladesh 2022-2023 (This study)	2023	Bangladesh	0	11	0	None	Negative	LT+STh	LT+ST	CS5+CS6	CS5+CS6	eatA	B1	5	L5	3	8
GVE0050	SAMN42182188	SRR29657665	Bangladesh 2022-2023 (This study)	2023	Bangladesh	20	0	0	Severe	Negative	LT+STh	LT+ST	Negative	CFnegative	Negative	A	-	L25	6	29
GVE0051	SAMN42182189	SRR29657654	Bangladesh 2022-2023 (This study)	2023	Bangladesh	46	0	0	Severe	Negative	STh	ST	Negative	CS23	Negative	B1	21	L19	4	15
GVE0052	SAMN42182190	SRR29657643	Bangladesh 2022-2023 (This study)	2023	Bangladesh	39	0	0	Severe	Negative	LT+STh	LT+ST	Negative	CFnegative	Negative	B1	108	L26	4	19
GVE0053	SAMN42182191	SRR29657792	Bangladesh 2022-2023 (This study)	2023	Bangladesh	1	10	0	None	Negative	LT+STp	LT+ST	CS12	CS12	Negative	D	535	L29	9	41
GVE0054	SAMN42182192	SRR29657781	Bangladesh 2022-2023 (This study)	2023	Bangladesh	0	8	15	Severe	Negative	LT+STh	LT+ST	Negative	CFnegative	Negative	B1	19	L20	4	14
GVE0055	SAMN42182193	SRR29657769	Bangladesh 2022-2023 (This study)	2023	Bangladesh	40	0	0	Severe	Negative	STh	ST	CFA/I+CS21	CFA/I+CS21	eatA,etpBC	A	-	L6	5	22
GVE0056	SAMN42182194	SRR29657945	Bangladesh 2022-2023 (This study)	2023	Bangladesh	55	0	0	None	Negative	LT+STh	LT+ST	CS5+CS6	CS5+CS6	eatA	B1	5	L5	3	8
GVE0057	SAMN42182195	SRR29657934	Bangladesh 2022-2023 (This study)	2023	Bangladesh	0	8	2	None	Negative	STp	ST	CS6	CS6	Negative	E	1010	L7	1	1
GVE0058	SAMN42182196	SRR29657987	Bangladesh 2022-2023 (This study)	2023	Bangladesh	27	0	0	Severe	Ogawa	STh	ST	Negative	CFnegative	Negative	B1	-	L26	4	19
GVE0059	SAMN42182197	SRR29657976	Bangladesh 2022-2023 (This study)	2023	Bangladesh	38	0	0	Severe	Negative	LT+STh	LT+ST	Negative	CS23	Negative	B1	21	L19	4	15
GVE0060	SAMN42182198	SRR29657965	Bangladesh 2022-2023 (This study)	2023	Bangladesh	57	0	0	Severe	Negative	STh	ST	CFA/I+CS21	CFA/I+CS21	eatA,etpABC	A	-	L6	5	22
GVE0061	SAMN42182199	SRR29657639	Bangladesh 2022-2023 (This study)	2023	Bangladesh	1	0	13	Severe	Negative	LT	LT	Negative	CFnegative	Negative	A	-	L11+L15	2	4
GVE0062	SAMN42182200	SRR29657628	Bangladesh 2022-2023 (This study)	2023	Bangladesh	0	4	23	Severe	Negative	STh	ST	CFA/I+CS21	CFA/I+CS21	eatA,etpBC	A	-	L6	5	22
GVE0063	SAMN42182201	SRR29657617	Bangladesh 2022-2023 (This study)	2023	Bangladesh	32	0	0	Severe	Negative	STh	ST	Negative	CFnegative	etpBC	A	843	L11+L15	2	4
GVE0064	SAMN42182202	SRR29657766	Bangladesh 2022-2023 (This study)	2023	Bangladesh	19	0	0	None	Negative	LT	LT	CS7	CS7	eatA,etpABC	B1	-	L3	4	12
GVE0065	SAMN42182203	SRR29657754	Bangladesh 2022-2023 (This study)	2023	Bangladesh	1	1	15	None	Negative	STp	ST	CS6	CS6	Negative	B1	86	L18	4	16
GVE0066	SAMN42182204	SRR29657743	Bangladesh 2022-2023 (This study)	2023	Bangladesh	31	0	0	Severe	Negative	LT	LT	Negative	CS12	Negative	B1	-	L20	4	14
GVE0067	SAMN42182205	SRR29657604	Bangladesh 2022-2023 (This study)	2023	Bangladesh	36	0	0	Some	Negative	LT	LT	CS6+CS8	CS6+CS8	eatA	A	-	L4	6	28
GVE0068	SAMN42182206	SRR29657593	Bangladesh 2022-2023 (This study)	2023	Bangladesh	55	0	0	None	Negative	LT+STh	LT+ST	CS5+CS6	CS5+CS6	eatA	B1	5	L5	3	8
GVE0069	SAMN42182207	SRR29657582	Bangladesh 2022-2023 (This study)	2023	Bangladesh	1	3	19	None	Negative	STh	ST	CS21	CS21	etpBC	A	843	NA	6	31
GVE0070	SAMN42182208	SRR29657571	Bangladesh 2022-2023 (This study)	2023	Bangladesh	0	1	10	None	Negative	LT	LT	CS6	CS6	Negative	B1	-	NA	3	9
GVE0071	SAMN42182209	SRR29657560	Bangladesh 2022-2023 (This study)	2023	Bangladesh	35	0	0	Severe	Negative	STh	ST	CFA/I+CS21	CFA/I+CS21	eatA,etpBC	A	-	L6	5	22
GVE0072	SAMN42182210	SRR29657549	Bangladesh 2022-2023 (This study)	2023	Bangladesh	42	0	0	Severe	Negative	LT+STh	LT+ST	Negative	CS23	Negative	A	-	L25	6	29
GVE0073	SAMN42182211	SRR29657922	Bangladesh 2022-2023 (This study)	2023	Bangladesh	1	10	0	None	Negative	LT+STh	LT+ST	CS5+CS6	CS5+CS6	eatA	B1	5	L5	3	8
GVE0074	SAMN42182212	SRR29657921	Bangladesh 2022-2023 (This study)	2023	Bangladesh	0	4	10	Severe	Negative	STh	ST	Negative	CFnegative	Negative	B1	-	L20	4	14
GVE0075	SAMN42182213	SRR29657919	Bangladesh 2022-2023 (This study)	2023	Bangladesh	50	0	0	None	Negative	LT+STh	LT+ST	CS5+CS6	CS5+CS6	eatA	B1	5	L5	3	8
GVE0076	SAMN42182214	SRR29657918	Bangladesh 2022-2023 (This study)	2023	Bangladesh	0	9	5	None	Negative	LT+STh	LT+ST	CS5+CS6	CS5+CS6	eatA	B1	5	L5	3	8
GVE0077	SAMN42182215	SRR29657917	Bangladesh 2022-2023 (This study)	2023	Bangladesh	1	6	0	Severe	Negative	STh	ST	CFA/I+CS21	CFA/I+CS21	eatA,etpBC	B1	-	L3	4	12
GVE0078	SAMN42182216	SRR29657916	Bangladesh 2022-2023 (This study)	2023	Bangladesh	19	0	0	None	Negative	STh	ST	CS4+CS6	CS6	eatA	A	-	NA	2	5
GVE0079	SAMN42182217	SRR29657915	Bangladesh 2022-2023 (This study)	2023	Bangladesh	3	6	0	Severe	Negative	STh	ST	Negative	CFnegative	etpBC	A	843	L11+L15	2	4
GVE0080	SAMN42182218	SRR29657914	Bangladesh 2022-2023 (This study)	2023	Bangladesh	1	2	24	None	Negative	STp	ST	CS6	CS6+PCFO71	Negative	B1	-	L19	4	15
GVE0081	SAMN42182219	SRR29657913	Bangladesh 2022-2023 (This study)	2023	Bangladesh	1	8	0	None	Negative	LT+STh	LT+ST	CS5+CS6	CS5+CS6	eatA	B1	5	L5	3	8
GVE0082	SAMN42182220	SRR29657912	Bangladesh 2022-2023 (This study)	2023	Bangladesh	1	8	0	Severe	Negative	STh	ST	Negative	CFnegative	etpBC	A	843	L11+L15	2	4
GVE0083	SAMN42182221	SRR29657911	Bangladesh 2022-2023 (This study)	2023	Bangladesh	1	6	0	Severe	Negative	STh	ST	CFA/I+CS21	CFA/I+CS21	eatA,etpABC	A	-	L6	5	22
GVE0084	SAMN42182222	SRR29657910	Bangladesh 2022-2023 (This study)	2023	Bangladesh	30	0	0	Severe	Ogawa	STh	ST	CFA/I+CS21	CFA/I+CS21	eatA,etpABC	B1	-	L3	4	12
GVE0085	SAMN42182223	SRR29657908	Bangladesh 2022-2023 (This study)	2023	Bangladesh	40	0	0	Severe	Negative	LT+STh	LT+ST	Negative	CS23	Negative	B1	108	L26	4	19
GVE0093	SAMN42182224	SRR29657907	Bangladesh 2022-2023 (This study)	2023	Bangladesh	0	6	24	Some	Negative	LT+STh	LT+ST	CS1+CS3+CS21	CS1+CS3+CS21	eatA,etpBC	A	-	L1	6	26
GVE0094	SAMN42182225	SRR29657906	Bangladesh 2022-2023 (This study)	2023	Bangladesh	45	0	0	Severe	Ogawa	STh	ST	Negative	CFnegative	etpC	A	2	L11+L15	2	4
GVE0095	SAMN42182226	SRR29657905	Bangladesh 2022-2023 (This study)	2023	Bangladesh	0	11	0	None	Negative	LT+STh	LT+ST	CS5+CS6	CS5+CS6	eatA	B1	5	L5	3	8
GVE0096	SAMN42182227	SRR29657904	Bangladesh 2022-2023 (This study)	2023	Bangladesh	32	0	0	None	Negative	STp	ST	CS6	CS6	Negative	B1	86	L18	4	16
GVE0097	SAMN42182228	SRR29657903	Bangladesh 2022-2023 (This study)	2023	Bangladesh	1	6	0	Severe	Negative	LT	LT	Negative	CFnegative	etpA,etpC	B1	537	NA	3	10
GVE0098	SAMN42182229	SRR29657902	Bangladesh 2022-2023 (This study)	2023	Bangladesh	1	0	0	None	Negative	STh	ST	CFA/I	CFA/I+CS21	eatA,etpBC	B1	-	L3	4	12
GVE0099	SAMN42182230	SRR29657901	Bangladesh 2022-2023 (This study)	2023	Bangladesh	9	0	0	Severe	Negative	STh	ST	CFA/I+CS21	CFA/I+CS21	eatA,etpBC	B1	-	L3	4	12
GVE0100	SAMN42182231	SRR29657900	Bangladesh 2022-2023 (This study)	2023	Bangladesh	3	0	0	Severe	Ogawa	STh	ST	Negative	CFnegative	Negative	B1	21	L19	4	15
GVE0101	SAMN42182232	SRR29657899	Bangladesh 2022-2023 (This study)	2023	Bangladesh	40	0	0	Severe	Negative	STh	ST	CFA/I+CS21	CFA/I+CS21	eatA,etpBC	B1	-	L3	4	12
GVE0102	SAMN42182233	SRR29657897	Bangladesh 2022-2023 (This study)	2023	Bangladesh	20	0	0	None	Ogawa	LT+STh	LT+ST	CS5+CS6	CS5+CS6	eatA	B1	5	L5	3	8
GVE0103	SAMN42182234	SRR29657896	Bangladesh 2022-2023 (This study)	2023	Bangladesh	30	0	0	Severe	Negative	STh	ST	Negative	CS23	Negative	B1	87	L19	4	15
GVE0104	SAMN42182235	SRR29657895	Bangladesh 2022-2023 (This study)	2023	Bangladesh	0	6	24	Severe	Negative	LT	LT	Negative	CS13	Negative	B1	21	L19	4	15
GVE0105	SAMN42182236	SRR29657894	Bangladesh 2022-2023 (This study)	2023	Bangladesh	19	5	0	Severe	Negative	STh	ST	Negative	CS23	Negative	A	-	L25	6	29
GVE0106	SAMN42182237	SRR29657893	Bangladesh 2022-2023 (This study)	2023	Bangladesh	0	11	18	Severe	Negative	LT	LT	Negative	CS13	Negative	A	-	NA	5	25
GVE0107	SAMN42182238	SRR29657892	Bangladesh 2022-2023 (This study)	2023	Bangladesh	45	0	0	Severe	Ogawa	LT+STh	LT+ST	Negative	CS23	Negative	F	-	NA	9	39
GVE0108	SAMN42182239	SRR29657891	Bangladesh 2022-2023 (This study)	2023	Bangladesh	45	0	0	Severe	Negative	STh	ST	Negative	CS23	Negative	A	-	L25	6	29
GVE0109	SAMN42182240	SRR29657890	Bangladesh 2022-2023 (This study)	2023	Bangladesh	0	7	0	Severe	Negative	LT	LT	Negative	CFnegative	Negative	B1	21	L19	4	15
GVE0110	SAMN42182241	SRR29657889	Bangladesh 2022-2023 (This study)	2023	Bangladesh	10	0	0	None	Negative	LT	LT	CS7	CS7	eatA,etpABC	B1	-	L3	4	12
GVE0111	SAMN42182242	SRR29657888	Bangladesh 2022-2023 (This study)	2023	Bangladesh	16	0	0	Severe	Negative	STh	ST	Negative	CFnegative	Negative	B1	-	NA	5	24
GVE0112	SAMN42182243	SRR29657886	Bangladesh 2022-2023 (This study)	2023	Bangladesh	0	7	16	Severe	Negative	STh	ST	Negative	CFnegative	eatA,etpBC	B1	-	L20	4	14
GVE0126	SAMN42182244	SRR29657885	Bangladesh 2022-2023 (This study)	2023	Bangladesh	0	7	25	None	Negative	STh	ST	CS21	CS21	eatA,etpBC	A	843	NA	6	31
GVE0127	SAMN42182245	SRR29657884	Bangladesh 2022-2023 (This study)	2023	Bangladesh	4	6	27	None	Negative	STp	ST	CS6+CS21	CS6	Negative	B1	357	L17	4	11
GVE0128	SAMN42182246	SRR29657883	Bangladesh 2022-2023 (This study)	2023	Bangladesh	0	5	10	Severe	Ogawa	LT+STp	LT+ST	Negative	CS20	Negative	D	-	L28	8	36
GVE0129	SAMN42182247	SRR29657882	Bangladesh 2022-2023 (This study)	2023	Bangladesh	45	0	0	Severe	Negative	LT+STh	LT+ST	Negative	CFnegative	Negative	A	-	L25	6	29
GVE0130	SAMN42182248	SRR29657881	Bangladesh 2022-2023 (This study)	2023	Bangladesh	50	0	0	Severe	Negative	LT	LT	Negative	CFnegative	Negative	B1	632	L27	4	20
GVE0131	SAMN42182249	SRR29657880	Bangladesh 2022-2023 (This study)	2023	Bangladesh	6	0	0	Severe	Negative	STh	ST	Negative	CFnegative	etpC	A	-	L12+L14	2	7
GVE0132	SAMN42182250	SRR29657879	Bangladesh 2022-2023 (This study)	2023	Bangladesh	50	0	0	None	Negative	LT+STh	LT+ST	CS5+CS6	CS5+CS6	eatA	B1	5	L5	3	8
GVE0133	SAMN42182251	SRR29657878	Bangladesh 2022-2023 (This study)	2023	Bangladesh	50	0	0	Severe	Negative	STh	ST	Negative	CFnegative	Negative	A	-	L25	6	29
GVE0134	SAMN42182252	SRR29657877	Bangladesh 2022-2023 (This study)	2023	Bangladesh	45	0	0	Severe	Negative	STh	ST	Negative	CS23	Negative	A	475	NA	6	30
GVE0135	SAMN42182253	SRR29657875	Bangladesh 2022-2023 (This study)	2023	Bangladesh	0	7	0	Severe	Negative	LT+STp	LT+ST	Negative	CS20	Negative	D	-	L28	8	36
GVE0136	SAMN42182254	SRR29657874	Bangladesh 2022-2023 (This study)	2023	Bangladesh	3	0	0	Some	Negative	STh	ST	CS14	CS14	etpBC	B1	-	L19	4	15
GVE0137	SAMN42182255	SRR29657873	Bangladesh 2022-2023 (This study)	2023	Bangladesh	45	0	0	None	Negative	LT	LT	CS6+CS21	CS6+CS21	eatA	A	155	L4	6	28
GVE0138	SAMN42182256	SRR29657872	Bangladesh 2022-2023 (This study)	2023	Bangladesh	26	0	0	Severe	Negative	STh	ST	Negative	CS23	Negative	B1	21	L19	4	15
GVE0139	SAMN42182257	SRR29657871	Bangladesh 2022-2023 (This study)	2023	Bangladesh	0	11	0	None	Negative	LT	LT	CS17	CS17	Negative	B1	-	L5	3	8
GVE0140	SAMN42182258	SRR29657870	Bangladesh 2022-2023 (This study)	2023	Bangladesh	10	0	0	Severe	Ogawa	LT+STh	LT+ST	Negative	CFnegative	Negative	B1	108	L26	4	19
GVE0141	SAMN42182259	SRR29657869	Bangladesh 2022-2023 (This study)	2023	Bangladesh	0	9	14	Severe	Negative	STh	ST	CFA/I+CS21	CFA/I+CS21	eatA,etpABC	B1	-	L3	4	12
GVE0142	SAMN42182260	SRR29657868	Bangladesh 2022-2023 (This study)	2023	Bangladesh	0	5	25	Severe	Negative	LT	LT	Negative	CFnegative	Negative	B1	-	L18	4	16
GVE0143	SAMN42182261	SRR29657867	Bangladesh 2022-2023 (This study)	2022	Bangladesh	4	4	0	Severe	Negative	STh	ST	Negative	CS21	etpBC	A	-	L4	6	28
GVE0144	SAMN42182262	SRR29657866	Bangladesh 2022-2023 (This study)	2022	Bangladesh	62	0	0	Severe	Negative	LT+STh	LT+ST	Negative	CFnegative	Negative	B1	19	L20	4	14
GVE0145	SAMN42182263	SRR29657862	Bangladesh 2022-2023 (This study)	2022	Bangladesh	42	0	0	Severe	Negative	LT+STh	LT+ST	Negative	CFnegative	Negative	B1	632	L27	4	20
GVE0146	SAMN42182264	SRR29657861	Bangladesh 2022-2023 (This study)	2022	Bangladesh	42	0	0	None	Negative	LT+STh	LT+ST	CS5+CS6	CS5+CS6	eatA	B1	5	L5	3	8
GVE0147	SAMN42182265	SRR29657860	Bangladesh 2022-2023 (This study)	2022	Bangladesh	37	0	0	Severe	Ogawa	LT+STh	LT+ST	Negative	CFnegative	Negative	A	-	L11+L13	2	6
GVE0148	SAMN42182266	SRR29657859	Bangladesh 2022-2023 (This study)	2022	Bangladesh	6	0	0	Some	Ogawa	LT+STh	LT+ST	CS1+CS3+CS21	CS1+CS3+CS21	eatA,etpBC	A	215	L1	6	26
GVE0149	SAMN42182267	SRR29657858	Bangladesh 2022-2023 (This study)	2022	Bangladesh	26	0	0	Severe	Negative	LT+STh	LT+ST	Negative	CFnegative	Negative	B1	-	L19	4	15
GVE0150	SAMN42182268	SRR29657857	Bangladesh 2022-2023 (This study)	2022	Bangladesh	35	0	0	Severe	Inaba	LT+STh	LT+ST	Negative	CFnegative	Negative	B1	21	L19	4	15
GVE0151	SAMN42182269	SRR29657856	Bangladesh 2022-2023 (This study)	2022	Bangladesh	42	0	0	Severe	Negative	STh	ST	Negative	CS21	etpBC	A	-	L4	6	28
GVE0152	SAMN42182270	SRR29657855	Bangladesh 2022-2023 (This study)	2022	Bangladesh	0	4	5	None	Inaba	LT+STh	LT+ST	CS2+CS3+CS21	CS2+CS3+CS21	eatA,etpABC	A	215	L2	6	27
GVE0153	SAMN42182271	SRR29657854	Bangladesh 2022-2023 (This study)	2022	Bangladesh	0	11	0	None	Negative	STp	ST	CS6	CS6	Negative	B1	86	L18	4	16
GVE0154	SAMN42182272	SRR29657853	Bangladesh 2022-2023 (This study)	2022	Bangladesh	26	0	0	Severe	Ogawa	LT+STh	LT+ST	Negative	CS23	Negative	B1	21	L19	4	15
GVE0155	SAMN42182273	SRR29657851	Bangladesh 2022-2023 (This study)	2022	Bangladesh	30	0	0	Severe	Negative	STh	ST	Negative	CFnegative	Negative	B1	-	L20	4	14
GVE0156	SAMN42182274	SRR29657850	Bangladesh 2022-2023 (This study)	2022	Bangladesh	1	6	26	None	Negative	STh	ST	CS5+CS6	CS5+CS6	eatA	B1	5	L5	3	8
GVE0157	SAMN42182275	SRR29657849	Bangladesh 2022-2023 (This study)	2022	Bangladesh	50	0	0	Severe	Negative	STh	ST	Negative	CFnegative	Negative	B1	-	L20	4	14
GVE0158	SAMN42182276	SRR29657848	Bangladesh 2022-2023 (This study)	2022	Bangladesh	28	0	0	Severe	Inaba	LT+STh	LT+ST	Negative	CS23	Negative	B1	21	L19	4	15
GVE0159	SAMN42182277	SRR29657847	Bangladesh 2022-2023 (This study)	2022	Bangladesh	22	0	0	Severe	Negative	LT+STh	LT+ST	Negative	CS23	Negative	D	77	L28	8	36
GVE0160	SAMN42182278	SRR29657846	Bangladesh 2022-2023 (This study)	2022	Bangladesh	35	0	0	Severe	Negative	LT+STh	LT+ST	Negative	CFnegative	Negative	B1	108	L26	4	19
GVE0161	SAMN42182279	SRR29657845	Bangladesh 2022-2023 (This study)	2022	Bangladesh	25	0	0	Severe	Negative	LT+STh	LT+ST	Negative	CFnegative	Negative	A	-	NA	6	30
GVE0162	SAMN42182280	SRR29657844	Bangladesh 2022-2023 (This study)	2022	Bangladesh	1	2	0	Severe	Negative	STh	ST	Negative	CFnegative	Negative	B1	19	L20	4	14
GVE0163	SAMN42182281	SRR29657843	Bangladesh 2022-2023 (This study)	2022	Bangladesh	14	0	0	Severe	Inaba	STh	ST	Negative	CFnegative	Negative	B1	-	L20	4	14
GVE0164	SAMN42182282	SRR29657842	Bangladesh 2022-2023 (This study)	2022	Bangladesh	65	0	0	Severe	Negative	STh	ST	CS4	CS6	eatA	A	-	NA	2	5
GVE0165	SAMN42182283	SRR29657840	Bangladesh 2022-2023 (This study)	2022	Bangladesh	0	7	0	Severe	Negative	LT	LT	Negative	CFnegative	Negative	B1	290	L20	4	14
GVE0166	SAMN42182284	SRR29657839	Bangladesh 2022-2023 (This study)	2022	Bangladesh	35	0	0	Severe	Ogawa	LT+STh	LT+ST	Negative	CS23	Negative	B1	-	L19	4	15
GVE0167	SAMN42182285	SRR29657838	Bangladesh 2022-2023 (This study)	2022	Bangladesh	0	6	0	Severe	Negative	LT+STp	LT+ST	Negative	CS12	Negative	D	535	L29	9	41
GVE0168	SAMN42182286	SRR29657837	Bangladesh 2022-2023 (This study)	2022	Bangladesh	35	0	0	Severe	Inaba	LT+STh	LT+ST	Negative	CS23	Negative	A	-	L25	6	29
GVE0169	SAMN42182287	SRR29657836	Bangladesh 2022-2023 (This study)	2022	Bangladesh	72	0	0	Severe	Inaba	STh	ST	Negative	CS23	Negative	B1	21	L19	4	15
GVE0170	SAMN42182288	SRR29657835	Bangladesh 2022-2023 (This study)	2023	Bangladesh	0	10	18	Severe	Negative	LT+STp	LT+ST	Negative	CS28B	Negative	B1	-	L18	4	16
GVE0171	SAMN42182289	SRR29657834	Bangladesh 2022-2023 (This study)	2022	Bangladesh	1	1	0	Severe	Negative	STh	ST	Negative	CFnegative	Negative	B1	632	L27	4	20
GVE0172	SAMN42182290	SRR29657833	Bangladesh 2022-2023 (This study)	2022	Bangladesh	38	0	0	Severe	Inaba	LT	LT	Negative	CS6+CS21	eatA	A	155	L4	6	28
GVE0173	SAMN42182291	SRR29657736	Bangladesh 2022-2023 (This study)	2022	Bangladesh	40	0	0	Severe	Inaba	LT+STh	LT+ST	Negative	CFnegative	Negative	F	-	NA	7	35
GVE0174	SAMN42182292	SRR29657735	Bangladesh 2022-2023 (This study)	2022	Bangladesh	50	0	0	Severe	Ogawa	LT+STh	LT+ST	Negative	CS5+CS6	eatA	B1	5	L5	3	8
GVE0175	SAMN42182293	SRR29657733	Bangladesh 2022-2023 (This study)	2022	Bangladesh	0	4	0	Severe	Negative	STh	ST	CFA/I+CS21	CFA/I+CS21	eatA,etpABC	A	-	L6	5	22
GVE0176	SAMN42182294	SRR29657732	Bangladesh 2022-2023 (This study)	2022	Bangladesh	2	2	0	Severe	Negative	LT	LT	Negative	CS13+CS23	eatA	B1	21	L19	4	15
GVE0177	SAMN42182295	SRR29657731	Bangladesh 2022-2023 (This study)	2022	Bangladesh	1	1	0	Severe	Ogawa	LT+STh	LT+ST	Negative	CS2+CS3	Negative	B1	537	NA	3	10
GVE0178	SAMN42182296	SRR29657730	Bangladesh 2022-2023 (This study)	2022	Bangladesh	52	0	0	Severe	Negative	STh	ST	Negative	CFnegative	Negative	B1	-	L20	4	14
GVE0179	SAMN42182297	SRR29657729	Bangladesh 2022-2023 (This study)	2022	Bangladesh	30	0	0	Severe	Negative	LT+STh	LT+ST	Negative	CFnegative	Negative	B1	108	L26	4	19
GVE0180	SAMN42182298	SRR29657728	Bangladesh 2022-2023 (This study)	2022	Bangladesh	50	0	0	Severe	Inaba	LT+STh	LT+ST	Negative	CS23	Negative	A	-	NA	6	30
GVE0181	SAMN42182299	SRR29657727	Bangladesh 2022-2023 (This study)	2022	Bangladesh	52	0	0	Severe	Negative	LT+STh	LT+ST	Negative	CFnegative	Negative	B1	479	L20	4	14
GVE0182	SAMN42182300	SRR29657726	Bangladesh 2022-2023 (This study)	2022	Bangladesh	28	0	0	Severe	Inaba	LT+STh	LT+ST	Negative	CFnegative	Negative	B1	108	L26	4	19
GVE0183	SAMN42182301	SRR29657725	Bangladesh 2022-2023 (This study)	2022	Bangladesh	35	0	0	None	Negative	LT+STh	LT+ST	CS5+CS6	CS5+CS6	eatA	B1	5	L5	3	8
GVE0184	SAMN42182302	SRR29657724	Bangladesh 2022-2023 (This study)	2022	Bangladesh	25	0	0	Severe	Inaba	LT+STh	LT+ST	Negative	CS23	Negative	B1	21	L19	4	15
GVE0185	SAMN42182303	SRR29657722	Bangladesh 2022-2023 (This study)	2022	Bangladesh	1	0	0	None	Ogawa	LT+STh	LT+ST	CS5+CS6	CS5+CS6	eatA	B1	5	L5	3	8
GVE0186	SAMN42182304	SRR29657721	Bangladesh 2022-2023 (This study)	2022	Bangladesh	55	0	0	Severe	Ogawa	LT+STh	LT+ST	Negative	CS23	Negative	B1	-	L19	4	15
GVE0187	SAMN42182305	SRR29657720	Bangladesh 2022-2023 (This study)	2022	Bangladesh	1	7	0	Severe	Negative	LT+STp	LT+ST	Negative	CS27A	Negative	B1	21	L19	4	15
GVE0188	SAMN42182306	SRR29657719	Bangladesh 2022-2023 (This study)	2022	Bangladesh	50	0	0	Severe	Inaba	LT+STh	LT+ST	Negative	CS23	Negative	B1	-	L19	4	15
GVE0189	SAMN42182307	SRR29657718	Bangladesh 2022-2023 (This study)	2022	Bangladesh	51	0	0	Severe	Negative	STh	ST	CFA/I+CS21	CFA/I+CS21	eatA,etpBC	A	-	L6	5	22
GVE0190	SAMN42182308	SRR29657717	Bangladesh 2022-2023 (This study)	2022	Bangladesh	1	8	0	Severe	Negative	LT+STh	LT+ST	Negative	CFnegative	Negative	B1	21	L19	4	15
GVE0191	SAMN42182309	SRR29657716	Bangladesh 2022-2023 (This study)	2022	Bangladesh	1	6	0	Severe	Negative	LT	LT	Negative	CFnegative	Negative	B1	-	L5	3	8
GVE0192	SAMN42182310	SRR29657715	Bangladesh 2022-2023 (This study)	2022	Bangladesh	0	10	0	Severe	Negative	LT+STp	LT+ST	Negative	CS20	Negative	F	-	NA	7	33
GVE0193	SAMN42182311	SRR29657714	Bangladesh 2022-2023 (This study)	2022	Bangladesh	25	0	0	Severe	Negative	LT+STh	LT+ST	Negative	CS5+CS6	eatA	B1	5	L5	3	8
GVE0194	SAMN42182312	SRR29657713	Bangladesh 2022-2023 (This study)	2022	Bangladesh	38	0	0	Severe	Ogawa	LT+STh	LT+ST	Negative	CS23	Negative	A	-	L25	6	29
GVE0195	SAMN42182313	SRR29657711	Bangladesh 2022-2023 (This study)	2022	Bangladesh	45	0	0	Some	Negative	LT+STh	LT+ST	CS1+CS3+CS21	CS1+CS3+CS21	eatA,etpBC	A	215	L1	6	26
GVE0196	SAMN42182314	SRR29657710	Bangladesh 2022-2023 (This study)	2022	Bangladesh	35	0	0	None	Negative	STh	ST	CS5+CS6	CS5+CS6	eatA	B1	5	L5	3	8
GVE0197	SAMN42182315	SRR29657709	Bangladesh 2022-2023 (This study)	2022	Bangladesh	40	0	0	Severe	Inaba	STh	ST	Negative	CS23	Negative	B1	21	L19	4	15
GVE0198	SAMN42182316	SRR29657708	Bangladesh 2022-2023 (This study)	2022	Bangladesh	30	0	0	Severe	Ogawa	LT	LT	Negative	CS23	Negative	A	-	L11+L13	2	6
GVE0199	SAMN42182317	SRR29657707	Bangladesh 2022-2023 (This study)	2022	Bangladesh	0	8	0	Some	Negative	STh	ST	CS14	CS14	eatA,etpBC	B1	87	L19	4	15
GVE0200	SAMN42182318	SRR29657706	Bangladesh 2022-2023 (This study)	2022	Bangladesh	55	0	0	Severe	Negative	LT+STh	LT+ST	Negative	CS23	Negative	A	-	NA	6	30
GVE0201	SAMN42182319	SRR29657705	Bangladesh 2022-2023 (This study)	2022	Bangladesh	35	0	0	None	Inaba	LT+STh	LT+ST	CS5+CS6	CS5+CS6	eatA	B1	5	L5	3	8
GVE0202	SAMN42182320	SRR29657704	Bangladesh 2022-2023 (This study)	2022	Bangladesh	43	0	0	Severe	Inaba	STh	ST	Negative	CS23	Negative	A	-	L25	6	29
GVE0203	SAMN42182321	SRR29657703	Bangladesh 2022-2023 (This study)	2022	Bangladesh	65	0	0	None	Negative	LT+STh	LT+ST	CS5+CS6	CS5+CS6	eatA	B1	5	L5	3	8
GVE0204	SAMN42182322	SRR29657702	Bangladesh 2022-2023 (This study)	2022	Bangladesh	28	0	0	Severe	Inaba	LT+STh	LT+ST	Negative	CFnegative	Negative	B1	21	L19	4	15
GVE0205	SAMN42182323	SRR29657700	Bangladesh 2022-2023 (This study)	2022	Bangladesh	45	0	0	Severe	Inaba	STh	ST	Negative	CFnegative	Negative	B1	632	L27	4	20
GVE0206	SAMN42182324	SRR29657699	Bangladesh 2022-2023 (This study)	2022	Bangladesh	40	0	0	Severe	Negative	STh	ST	Negative	CFnegative	Negative	B1	-	L20	4	14
GVE0207	SAMN42182325	SRR29657698	Bangladesh 2022-2023 (This study)	2022	Bangladesh	0	6	19	None	Negative	STh	ST	CFA/I	CFA/I+CS21	eatA,etpABC	B1	-	L3	4	12
GVE0208	SAMN42182326	SRR29657697	Bangladesh 2022-2023 (This study)	2022	Bangladesh	36	0	0	Severe	Negative	LT	LT	Negative	CS23	Negative	B1	632	L27	4	20
GVE0209	SAMN42182327	SRR29657696	Bangladesh 2022-2023 (This study)	2022	Bangladesh	1	1	0	None	Negative	STh	ST	CFA/I	CFA/I+CS21	eatA,etpABC	B1	-	L3	4	12
GVE0210	SAMN42182328	SRR29657695	Bangladesh 2022-2023 (This study)	2022	Bangladesh	0	5	23	Some	Negative	LT+STh	LT+ST	CS1+CS3+CS21	CS1+CS3+CS21	eatA,etpBC	A	215	L1	6	26
GVE0211	SAMN42182329	SRR29657694	Bangladesh 2022-2023 (This study)	2022	Bangladesh	1	3	0	None	Negative	STh	ST	CFA/I	CFA/I	eatA,etpABC	B1	-	L3	4	12
GVE0212	SAMN42182330	SRR29657693	Bangladesh 2022-2023 (This study)	2022	Bangladesh	35	0	0	Severe	Negative	STp	ST	Negative	CS6	Negative	NA	722	NA	9	42
GVE0213	SAMN42182331	SRR29657692	Bangladesh 2022-2023 (This study)	2022	Bangladesh	1	8	0	Severe	Negative	STh	ST	Negative	CS2+CS3+CS21	eatA,etpBC	A	215	L2	6	27
GVE0214	SAMN42182332	SRR29657691	Bangladesh 2022-2023 (This study)	2022	Bangladesh	12	0	0	Severe	Inaba	STh	ST	Negative	CFnegative	Negative	B1	-	L20	4	14
GVE0215	SAMN42182333	SRR29657689	Bangladesh 2022-2023 (This study)	2022	Bangladesh	60	0	0	Severe	Negative	STh	ST	Negative	CS2+CS3+CS21	eatA,etpBC	A	215	L2	6	27
GVE0216	SAMN42182334	SRR29657688	Bangladesh 2022-2023 (This study)	2022	Bangladesh	1	6	0	None	Negative	STp	ST	CS6	CS6	Negative	B1	86	L18	4	16
GVE0217	SAMN42182335	SRR29657687	Bangladesh 2022-2023 (This study)	2022	Bangladesh	0	7	6	None	Negative	STp	ST	CS6	CS6	Negative	B1	357	L17	4	11
GVE0218	SAMN42182336	SRR29657686	Bangladesh 2022-2023 (This study)	2022	Bangladesh	48	0	0	Severe	Ogawa	LT+STh	LT+ST	Negative	CS23	Negative	D	77	L28	8	36
GVE0219	SAMN42182337	SRR29657685	Bangladesh 2022-2023 (This study)	2022	Bangladesh	19	0	0	Severe	Negative	STh	ST	Negative	CS23	Negative	B1	21	L19	4	15
GVE0220	SAMN42182338	SRR29657684	Bangladesh 2022-2023 (This study)	2022	Bangladesh	50	0	0	Severe	Negative	STh	ST	Negative	CFnegative	Negative	A	-	L11+L13	2	6
GVE0221	SAMN42182339	SRR29657683	Bangladesh 2022-2023 (This study)	2022	Bangladesh	30	0	0	Severe	Inaba	STh	ST	Negative	CFnegative	Negative	B1	-	L20	4	14
GVE0222	SAMN42182340	SRR29657682	Bangladesh 2022-2023 (This study)	2022	Bangladesh	1	5	0	Severe	Negative	STh	ST	Negative	CS21	etpBC	A	843	NA	6	31
GVE0223	SAMN42182341	SRR29657681	Bangladesh 2022-2023 (This study)	2022	Bangladesh	35	0	0	Severe	Inaba	STh	ST	Negative	CFnegative	Negative	B1	-	L20	4	14
GVE0224	SAMN42182342	SRR29657680	Bangladesh 2022-2023 (This study)	2022	Bangladesh	50	0	0	None	Negative	STp	ST	CS5+CS6	CS5+CS6	eatA	B1	5	L5	3	8
GVE0225	SAMN42182343	SRR29657678	Bangladesh 2022-2023 (This study)	2022	Bangladesh	45	0	0	Severe	Negative	STh	ST	Negative	CFnegative	etpC	A	-	L11+L15	2	4
GVE0226	SAMN42182344	SRR29657677	Bangladesh 2022-2023 (This study)	2022	Bangladesh	50	0	0	Severe	Negative	LT	LT	Negative	CS23	Negative	B1	632	L27	4	20
GVE0227	SAMN42182345	SRR29657676	Bangladesh 2022-2023 (This study)	2022	Bangladesh	18	0	0	Severe	Negative	LT+STh	LT+ST	Negative	CFnegative	Negative	A	-	L25	6	29
GVE0248	SAMN42182346	SRR29657675	Bangladesh 2022-2023 (This study)	2022	Bangladesh	11	3	0	Severe	Negative	LT+STh	LT+ST	Negative	CFnegative	Negative	B1	108	L26	4	19
GVE0249	SAMN42182347	SRR29657674	Bangladesh 2022-2023 (This study)	2022	Bangladesh	0	10	29	None	Negative	LT	LT	CS17	CS17	Negative	B1	5	L5	3	8
GVE0250	SAMN42182348	SRR29657673	Bangladesh 2022-2023 (This study)	2022	Bangladesh	65	0	0	Severe	Negative	STh	ST	Negative	CFnegative	Negative	B1	-	L20	4	14
GVE0251	SAMN42182349	SRR29657832	Bangladesh 2022-2023 (This study)	2022	Bangladesh	40	0	0	Severe	Negative	STh	ST	Negative	CS23	Negative	A	-	L25	6	29
GVE0252	SAMN42182350	SRR29657831	Bangladesh 2022-2023 (This study)	2022	Bangladesh	26	0	0	Severe	Negative	LT+STh	LT+ST	Negative	CFnegative	Negative	B1	108	L26	4	19
GVE0253	SAMN42182351	SRR29657830	Bangladesh 2022-2023 (This study)	2022	Bangladesh	1	0	0	Severe	Negative	LT+STp	LT+ST	Negative	CS27A	Negative	A	2	L11+L15	2	4
GVE0254	SAMN42182352	SRR29657829	Bangladesh 2022-2023 (This study)	2022	Bangladesh	17	0	0	Severe	Inaba	STh	ST	Negative	CFnegative	Negative	B1	632	L27	4	20
GVE0255	SAMN42182353	SRR29657827	Bangladesh 2022-2023 (This study)	2022	Bangladesh	0	6	0	Severe	Negative	LT	LT	Negative	CFnegative	eatA,etpBC	A	-	L25	6	29
GVE0256	SAMN42182354	SRR29657826	Bangladesh 2022-2023 (This study)	2022	Bangladesh	40	0	0	Severe	Negative	LT+STh	LT+ST	CS5+CS6	CS5+CS6	eatA	B1	5	L5	3	8
GVE0257	SAMN42182355	SRR29657825	Bangladesh 2022-2023 (This study)	2022	Bangladesh	0	10	0	None	Negative	LT	LT	CS6	CS6+CS21	eatA	A	155	L4	6	28
GVE0258	SAMN42182356	SRR29657824	Bangladesh 2022-2023 (This study)	2022	Bangladesh	1	11	0	None	Negative	STh	ST	CS6	CS6+CS21	Negative	B1	58	L8	4	13
GVE0259	SAMN42182357	SRR29657823	Bangladesh 2022-2023 (This study)	2022	Bangladesh	55	0	0	Severe	Negative	STh	ST	Negative	CFnegative	Negative	B1	-	L20	4	14
GVE0260	SAMN42182358	SRR29657822	Bangladesh 2022-2023 (This study)	2022	Bangladesh	60	0	0	Severe	Negative	LT	LT	Negative	CFnegative	Negative	B1	108	L26	4	19
GVE0261	SAMN42182359	SRR29657821	Bangladesh 2022-2023 (This study)	2022	Bangladesh	26	0	0	Severe	Negative	LT+STh	LT+ST	Negative	CFnegative	Negative	B1	-	L18	4	16
GVE0262	SAMN42182360	SRR29657820	Bangladesh 2022-2023 (This study)	2022	Bangladesh	61	0	0	Severe	Negative	LT+STh	LT+ST	CS8	CFnegative	Negative	B1	108	L26	4	19
GVE0263	SAMN42182361	SRR29657819	Bangladesh 2022-2023 (This study)	2022	Bangladesh	0	8	13	Severe	Negative	LT	LT	Negative	CFnegative	Negative	B1	87	L19	4	15

GVE0264	SAMN42182362	SRR29657818	Bangladesh 2022-2023 (This study)	2022	Bangladesh	0	7	0	Severe	Negative	LT	LT	Negative	CFnegative	etpBC	B1	537	NA	3	10
GVE0265	SAMN42182363	SRR29657815	Bangladesh 2022-2023 (This study)	2022	Bangladesh	3	0	0	None	Negative	STh	ST	CS7	CFnegative	eatA,etpBC	B1	-	L20	4	14
GVE0266	SAMN42182364	SRR29657814	Bangladesh 2022-2023 (This study)	2022	Bangladesh	0	7	0	Severe	Negative	LT	LT	Negative	CFnegative	etpBC	A	-	L25	6	29
GVE0267	SAMN42182365	SRR29657813	Bangladesh 2022-2023 (This study)	2022	Bangladesh	0	11	0	None	Ogawa	LT+STp	LT+ST	PCFO159	CS12	Negative	D	535	L29	9	41
GVE0268	SAMN42182366	SRR29657812	Bangladesh 2022-2023 (This study)	2022	Bangladesh	0	10	28	Severe	Negative	STh	ST	Negative	CFA/I+CS21	eatA,etpBC	A	-	L6	5	22
GVE0269	SAMN42182367	SRR29657811	Bangladesh 2022-2023 (This study)	2022	Bangladesh	1	10	0	None	Negative	STh	ST	CFA/I	CFA/I+CS21	eatA,etpBC	B1	-	L3	4	12
GVE0270	SAMN42182368	SRR29657810	Bangladesh 2022-2023 (This study)	2022	Bangladesh	0	8	15	Severe	Negative	LT	LT	Negative	CS28A	Negative	B1	-	L18	4	16
GVE0271	SAMN42182369	SRR29657809	Bangladesh 2022-2023 (This study)	2022	Bangladesh	25	0	0	None	Negative	LT	LT	CS6	CS6+CS21	eatA	A	155	L4	6	28
GVE0272	SAMN42182370	SRR29657808	Bangladesh 2022-2023 (This study)	2022	Bangladesh	1	0	0	Some	Negative	STh	ST	CS14	CS14	etpBC	B1	-	L19	4	15
GVE0273	SAMN42182371	SRR29657807	Bangladesh 2022-2023 (This study)	2022	Bangladesh	0	5	18	None	Negative	STh	ST	CFA/I	CFA/I+CS21	eatA,etpBC	B1	-	L3	4	12
GVE0274	SAMN42182372	SRR29657806	Bangladesh 2022-2023 (This study)	2022	Bangladesh	1	0	0	None	Negative	STh	ST	CS2+CS3	CFnegative	eatA,etpBC	B1	-	L20	4	14
GVE0275	SAMN42182373	SRR29657804	Bangladesh 2022-2023 (This study)	2022	Bangladesh	35	0	0	Severe	Negative	STh	ST	CS8	CS23	Negative	A	-	L25	6	29
GVE0276	SAMN42182374	SRR29657803	Bangladesh 2022-2023 (This study)	2022	Bangladesh	3	0	0	None	Ogawa	LT	LT	CS6	CS6+CS21	eatA	A	155	L4	6	28
GVE0277	SAMN42182375	SRR29657802	Bangladesh 2022-2023 (This study)	2022	Bangladesh	1	9	0	Severe	Negative	STh	ST	CFA/I+CS21	CFA/I+CS21	eatA,etpBC	A	-	L6	5	22
GVE0278	SAMN42182376	SRR29657801	Bangladesh 2022-2023 (This study)	2022	Bangladesh	0	9	0	Severe	Negative	LT	LT	Negative	CS27B	Negative	B1	21	L19	4	15
GVE0279	SAMN42182377	SRR29657544	Bangladesh 2022-2023 (This study)	2022	Bangladesh	34	0	0	Severe	Negative	STh	ST	Negative	CS27B	Negative	B1	-	L20	4	14
GVE0280	SAMN42182378	SRR29657543	Bangladesh 2022-2023 (This study)	2022	Bangladesh	20	0	0	Severe	Negative	LT+STh	LT+ST	Negative	CS23	Negative	B1	632	L27	4	20
GVE0281	SAMN42182379	SRR29657542	Bangladesh 2022-2023 (This study)	2022	Bangladesh	40	0	0	Severe	Inaba	STh	ST	Negative	CFA/I+CS21	eatA,etpBC	A	-	L6	5	22
GVE0282	SAMN42182380	SRR29657541	Bangladesh 2022-2023 (This study)	2022	Bangladesh	55	0	0	Severe	Negative	STh	ST	Negative	CS21	eatA,etpBC	A	843	NA	6	31
GVE0283	SAMN42182381	SRR29657540	Bangladesh 2022-2023 (This study)	2022	Bangladesh	0	8	0	Severe	Negative	LT+STh	LT+ST	Negative	CFnegative	Negative	B1	108	L26	4	19
GVE0284	SAMN42182382	SRR29657539	Bangladesh 2022-2023 (This study)	2022	Bangladesh	1	7	0	Severe	Negative	STp	ST	CS6	CS6	Negative	B1	-	L19	4	15
GVE0285	SAMN42182383	SRR29657537	Bangladesh 2022-2023 (This study)	2022	Bangladesh	1	1	0	Severe	Negative	STh	ST	Negative	CFnegative	Negative	B1	-	L20	4	14
GVE0286	SAMN42182384	SRR29657536	Bangladesh 2022-2023 (This study)	2022	Bangladesh	0	1	15	None	Negative	LT+STh	LT+ST	CS5+CS6	CFnegative	Negative	B1	108	L26	4	19
GVE0287	SAMN42182385	SRR29657535	Bangladesh 2022-2023 (This study)	2022	Bangladesh	27	0	0	Some	Negative	STh	ST	CS14	CS14	etpBC	B1	-	L19	4	15
GVE0288	SAMN42182386	SRR29657534	Bangladesh 2022-2023 (This study)	2022	Bangladesh	30	0	0	Severe	Negative	LT+STh	LT+ST	Negative	CFnegative	Negative	B1	108	L26	4	19
GVE0289	SAMN42182387	SRR29657533	Bangladesh 2022-2023 (This study)	2022	Bangladesh	19	0	0	Severe	Ogawa	STp	ST	Negative	CS6	Negative	B1	357	L17	4	11
GVE0294	SAMN42182388	SRR29657532	Bangladesh 2022-2023 (This study)	2022	Bangladesh	1	0	0	Severe	Negative	LT	LT	Negative	CS13	Negative	A	398	L9+L10+L2	2	3
GVE0301	SAMN42182389	SRR29657531	Bangladesh 2022-2023 (This study)	2022	Bangladesh	0	11	23	Severe	Ogawa	LT+STp	LT+ST	Negative	CS20	Negative	F	-	NA	7	33
GVE0302	SAMN42182390	SRR29657530	Bangladesh 2022-2023 (This study)	2022	Bangladesh	39	0	0	Severe	Negative	LT+STh	LT+ST	Negative	CFnegative	Negative	B1	108	L26	4	19
GVE0304	SAMN42182391	SRR29658004	Bangladesh 2022-2023 (This study)	2022	Bangladesh	1	1	0	Severe	Negative	LT	LT	Negative	CFnegative	Negative	B1	87	L19	4	15
GVE0305	SAMN42182392	SRR29658003	Bangladesh 2022-2023 (This study)	2022	Bangladesh	44	0	0	Severe	Negative	LT+STh	LT+ST	Negative	CFnegative	Negative	B1	108	L26	4	19
GVE0306	SAMN42182393	SRR29658001	Bangladesh 2022-2023 (This study)	2022	Bangladesh	2	0	2	None	Negative	STh	ST	CS21	CS21	etpBC	A	843	NA	6	31
GVE0307	SAMN42182394	SRR29658000	Bangladesh 2022-2023 (This study)	2022	Bangladesh	1	4	0	Severe	Negative	LT+STp	LT+ST	Negative	CS13+CS27A	Negative	B1	21	L19	4	15
GVE0309	SAMN42182395	SRR29657999	Bangladesh 2012-2021 (This study)	2013	Bangladesh	NA	NA	NA	Severe	Negative	STh	ST	Negative	CFnegative	Negative	B1	632	L27	4	20
GVE0310	SAMN42182396	SRR29657998	Bangladesh 2012-2021 (This study)	2015	Bangladesh	NA	NA	NA	Severe	Negative	STh	ST	Negative	CFnegative	Negative	B1	471	L16	5	23
GVE0311	SAMN42182397	SRR29657997	Bangladesh 2022-2023 (This study)	2022	Bangladesh	0	6	0	None	Negative	LT+STh	LT+ST	Negative	CFnegative	Negative	A	-	L11+L13	2	6
GVE0312	SAMN42182398	SRR29657996	Bangladesh 2022-2023 (This study)	2022	Bangladesh	45	0	0	Some	Negative	LT+STh	LT+ST	CS1+CS3+CS21	CS1+CS3+CS21	eatA,etpBC	A	-	L1	6	26
GVE0313	SAMN42182399	SRR29657995	Bangladesh 2012-2021 (This study)	2016	Bangladesh	NA	NA	NA	None	Negative	LT+STh	LT+ST	Negative	CFnegative	Negative	B1	730	L20	4	14
GVE0314	SAMN42182400	SRR29657994	Bangladesh 2022-2023 (This study)	2022	Bangladesh	30	0	0	Some	Ogawa	STp	ST	Negative	F41a	Negative	B1	86	L18	4	16
GVE0315	SAMN42182401	SRR29657993	Bangladesh 2022-2023 (This study)	2022	Bangladesh	0	11	0	Some	Negative	LT	LT	Negative	CS6	Negative	B1	-	NA	3	9
GVE0316	SAMN42182402	SRR29657992	Bangladesh 2022-2023 (This study)	2022	Bangladesh	0	10	0	None	Negative	LT	LT	CS7	CS7	eatA,etpBC	B1	-	L3	4	12
GVE0317	SAMN42182403	SRR29657990	Bangladesh 2022-2023 (This study)	2022	Bangladesh	60	0	0	Severe	Negative	LT	LT	CS6	CS6+CS21	eatA	A	155	L4	6	28
GVE0318	SAMN42182404	SRR29657989	Bangladesh 2022-2023 (This study)	2022	Bangladesh	60	0	0	None	Negative	LT+STp	LT+ST	PCFO159	CS12	Negative	D	535	L29	9	41
GVE0319	SAMN42182405	SRR29657988	Bangladesh 2022-2023 (This study)	2022	Bangladesh	0	9	4	Some	Negative	STh	ST	CS14	CS14	eatA,etpBC	B1	546	L20	4	14
GVE0320	SAMN42182406	SRR29657672	Bangladesh 2022-2023 (This study)	2022	Bangladesh	0	7	0	Some	Negative	LT	LT	CS6+CS8	CS6+CS8	eatA	A	155	L4	6	28
GVE0321	SAMN42182407	SRR29657671	Bangladesh 2012-2021 (This study)	2016	Bangladesh	NA	NA	NA	Some	Ogawa	LT+STh	LT+ST	CS5+CS6	CFnegative	Negative	B1	19	L20	4	14
GVE0322	SAMN42182408	SRR29657670	Bangladesh 2022-2023 (This study)	2022	Bangladesh	0	9	12	Severe	Negative	STh	ST	CFA/I+CS21	CFA/I+CS21	eatA,etpBC	A	-	L6	5	22
GVE0323	SAMN42182409	SRR29657669	Bangladesh 2012-2021 (This study)	2017	Bangladesh	NA	NA	NA	Some	Negative	STh	ST	Negative	CFnegative	Negative	A	637	L11+L13	2	6
GVE0324	SAMN42182410	SRR29657668	Bangladesh 2022-2023 (This study)	2022	Bangladesh	0	10	2	None	Negative	LT	LT	CS7	CS7	eatA,etpBC	B1	-	L3	4	12
GVE0325	SAMN42182411	SRR29657667	Bangladesh 2022-2023 (This study)	2022	Bangladesh	1	1	0	None	Negative	LT+STp	LT+ST	CS1+CS3 , PCFO159	CS1+CS3+CS12+CS21	eatA,etpBC	D	-	L29	9	41
GVE0326	SAMN42182412	SRR29657666	Bangladesh 2012-2021 (This study)	2013	Bangladesh	NA	NA	NA	None	Negative	LT+STh	LT+ST	Negative	CFnegative	Negative	B1	730	L20	4	14
GVE0327	SAMN42182413	SRR29657664	Bangladesh 2022-2023 (This study)	2022	Bangladesh	45	0	0	Severe	Ogawa	LT+STh	LT+ST	Negative	CS23	Negative	D	535	L29	9	41
GVE0328	SAMN42182414	SRR29657663	Bangladesh 2022-2023 (This study)	2022	Bangladesh	72	0	0	Severe	Ogawa	STh	ST	Negative	CS23	Negative	B1	21	L19	4	15
GVE0329	SAMN42182415	SRR29657662	Bangladesh 2022-2023 (This study)	2022	Bangladesh	3	0	0	Severe	Negative	LT	LT	Negative	CS6+CS8	eatA	A	155	L4	6	28
GVE0330	SAMN42182416	SRR29657661	Bangladesh 2022-2023 (This study)	2022	Bangladesh	48	0	0	Severe	Ogawa	LT+STh	LT+ST	Negative	CS23	Negative	B1	-	L19	4	15
GVE0331	SAMN42182417	SRR29657660	Bangladesh 2022-2023 (This study)	2022	Bangladesh	55	0	0	None	Negative	STh	ST	CFA/I	CS21	eatA,etpBC	D	3	NA	8	37
GVE0332	SAMN42182418	SRR29657659	Bangladesh 2022-2023 (This study)	2022	Bangladesh	1	7	0	Severe	Negative	LT+STp	LT+ST	Negative	CS20	Negative	F	-	NA	7	33
GVE0333	SAMN42182419	SRR29657658	Bangladesh 2022-2023 (This study)	2022	Bangladesh	1	0	0	Some	Negative	LT	LT	CS5+CS6	CS5+CS6	eatA	B1	5	L5	3	8
GVE0334	SAMN42182420	SRR29657657	Bangladesh 2022-2023 (This study)	2022	Bangladesh	30	0	0	Severe	Negative	STh	ST	Negative	CS5+CS6	eatA	B1	5	L5	3	8
GVE0335	SAMN42182421	SRR29657656	Bangladesh 2022-2023 (This study)	2022	Bangladesh	65	0	0	Severe	Negative	LT	LT	Negative	CS23	Negative	B1	108	L26	4	19
GVE0336	SAMN42182422	SRR29657655	Bangladesh 2022-2023 (This study)	2022	Bangladesh	1	3	0	None	Negative	STp	ST	CS6	CS6	Negative	B1	86	L18	4	16
GVE0337	SAMN42182423	SRR29657653	Bangladesh 2012-2021 (This study)	2017	Bangladesh	NA	NA	NA	Some	Negative	LT+STh	LT+ST	Negative	CFnegative	Negative	A	-	NA	6	30
GVE0338	SAMN42182424	SRR29657652	Bangladesh 2022-2023 (This study)	2022	Bangladesh	1	1	0	Some	Negative	LT+STh	LT+ST	CS1+CS3+CS21	CS1+CS3+CS21	eatA,etpBC	A	215	L1	6	26
GVE0340	SAMN42182425	SRR29657651	Bangladesh 2022-2023 (This study)	2022	Bangladesh	26	0	0	Severe	Ogawa	LT+STh	LT+ST	Negative	CS23	Negative	B1	-	L19	4	15
GVE0341	SAMN42182426	SRR29657650	Bangladesh 2022-2023 (This study)	2022	Bangladesh	30	0	0	Severe	Inaba	STh	ST	Negative	CS23	Negative	A	-	L11+L13	2	6
GVE0342	SAMN42182427	SRR29657649	Bangladesh 2022-2023 (This study)	2022	Bangladesh	1	6	0	None	Negative	STp	ST	CS6	CS6	Negative	B1	357	L17	4	11
GVE0343	SAMN42182428	SRR29657648	Bangladesh 2022-2023 (This study)	2022	Bangladesh	23	0	0	Severe	Negative	LT	LT	CS6	CS6+CS21	eatA	A	155	L4	6	28
GVE0344	SAMN42182429	SRR29657647	Bangladesh 2022-2023 (This study)	2022	Bangladesh	4	0	0	Some	Negative	LT	LT	CS1+CS3+CS21	CS2+CS3+CS21	eatA,etpBC	A	215	L2	6	27
GVE0345	SAMN42182430	SRR29657646	Bangladesh 2022-2023 (This study)	2022	Bangladesh	0	10	0	None	Negative	LT+STp	LT+ST	PCFO159	CS12	Negative	D	535	L29	9	41
GVE0346	SAMN42182431	SRR29657645	Bangladesh 2012-2021 (This study)	2012	Bangladesh	NA	NA	NA	Severe	Negative	LT+ST	LT+ST	CFA/I	CFnegative	Negative	B1	-	L18	4	16
GVE0347	SAMN42182432	SRR29657644	Bangladesh 2022-2023 (This study)	2022	Bangladesh	1	0	0	None	Negative	LT	LT	CS5+CS6	CS17	eatA,etpBC	B1	69	NA	5	24
GVE0348	SAMN42182433	SRR29657642	Bangladesh 2022-2023 (This study)	2022	Bangladesh	1	0	0	None	Negative	LT+STp	LT+ST	CS17	CS12	Negative	D	535	L29	9	41
GVE0349	SAMN42182434	SRR29657641	Bangladesh 2012-2021 (This study)	2017	Bangladesh	NA	NA	NA	None	Negative	LT+STh	LT+ST	Negative	CFnegative	Negative	B1	108	L26	4	19
GVE0350	SAMN42182435	SRR29657800	Bangladesh 2012-2021 (This study)	2015	Bangladesh	NA	NA	NA	Some	Negative	LT	LT	CS2+CS3+CS21	CFnegative	Negative	A	-	L11+L15	2	4
GVE0351	SAMN42182436	SRR29657799	Bangladesh 2022-2023 (This study)	2022	Bangladesh	0	6	27	Severe	Negative	STh	ST	CFA/I+CS21	CFA/I+CS21	eatA,etpBC	NA	-	NA	9	42
GVE0352	SAMN42182437	SRR29657798	Bangladesh 2022-2023 (This study)	2022	Bangladesh	17	0	0	Severe	Ogawa	LT+STp	LT+ST	Negative	CS12	Negative	D	535	L29	9	41
GVE0353	SAMN42182438	SRR29657797	Bangladesh 2012-2021 (This study)	2017	Bangladesh	NA	NA	NA	Severe	Negative	STh	ST	Negative	CFnegative	Negative	A	-	L25	6	29
GVE0354	SAMN42182439	SRR29657796	Bangladesh 2022-2023 (This study)	2022	Bangladesh	0	8	10	None	Negative	STh	ST	CFA/I	CFA/I+CS21	eatA,etpBC	B1	-	L3	4	12
GVE0355	SAMN42182440	SRR29657795	Bangladesh 2012-2021 (This study)	2013	Bangladesh	NA	NA	NA	None	Negative	LT	LT	Negative	CFnegative	Negative	B1	-	L17	4	11
GVE0356	SAMN42182441	SRR29657794	Bangladesh 2022-2023 (This study)	2022	Bangladesh	2	6	0	Severe	Negative	STp	ST	Negative	CS6	Negative	E	1010	L7	1	1
GVE0357	SAMN42182442	SRR29657793	Bangladesh 2012-2021 (This study)	2012	Bangladesh	NA	NA	NA	Some	Negative	STh	ST	Negative	CFnegative	Negative	B1	-	L20	4	14
GVE0358	SAMN42182443	SRR29657791	Bangladesh 2022-2023 (This study)	2022	Bangladesh	4	4	0	None	Negative	STp	ST	CS7	CS6	Negative	D	-	L29	9	41
GVE0359	SAMN42182444	SRR29657790	Bangladesh 2012-2021 (This study)	2013	Bangladesh	NA	NA	NA	None	Negative	LT	LT	Negative	CFnegative	Negative	B1	537	NA	3	10
GVE0360	SAMN42182445	SRR29657789	Bangladesh 2022-2023 (This study)	2022	Bangladesh	37	0	0	Severe	Inaba	LT+STh	LT+ST	Negative	CFnegative	Negative	B1	479	L20	4	14
GVE0361	SAMN42182446	SRR29657788	Bangladesh 2022-2023 (This study)	2022	Bangladesh	45	0	0	Severe	Inaba	STp	ST	Negative	CS6	Negative	B1	-	L19	4	15
GVE0363	SAMN42182447	SRR29657787	Bangladesh 2022-2023 (This study)	2022	Bangladesh	21	0	0	None	Ogawa	STh	ST	CFA/I	CFA/I+CS21	eatA,etpBC	B1	-	L3	4	12
GVE0364	SAMN42182448	SRR29657786	Bangladesh 2022-2023 (This study)	2022	Bangladesh	26	0	0	Severe	Negative	LT+STh	LT+ST	CS5+CS6	CS5+CS6	eatA	B1	5	L5	3	8
GVE0365	SAMN42182449	SRR29657785	Bangladesh 2022-2023 (This study)	2022	Bangladesh	0	8	15	Severe	Negative	LT+STp	LT+ST	Negative	CS13+CS27A	Negative	B1	21	L19	4	15
GVE0366	SAMN42182450	SRR29657784	Bangladesh 2022-2023 (This study)	2022	Bangladesh	12	0	0	Severe	Negative	STh	ST	Negative	CS23	Negative	B1	-	L19	4	15
GVE0367	SAMN42182451	SRR29657783	Bangladesh 2022-2023 (This study)	2022	Bangladesh	0	10	6	None	Negative	LT	LT	Negative	CS28A	Negative	B2	-	NA	9	40
GVE0368	SAMN42182452	SRR29657782	Bangladesh 2022-2023 (This study)	2022	Bangladesh	2	3	0	None	Inaba	STh	ST	CS2+CS3	CS2+CS3+CS21	eatA,etpBC	A	215	L2	6	27
GVE0369	SAMN42182453	SRR29657780	Bangladesh 2022-2023 (This study)	2022	Bangladesh	30	0	0	None	Inaba	STh	ST	CS5+CS6	CFnegative	Negative	F	-	NA	9	39
GVE0370	SAMN42182454	SRR29657779	Bangladesh 2012-2021 (This study)	2020	Bangladesh	NA	NA	NA	Severe	Inaba	LT	LT	Negative	CFnegative	etpBC	F	-	NA	9	39
GVE0398	SAMN42182455	SRR29657778	Bangladesh 2022-2023 (This study)	2023	Bangladesh	0	4	10	Some	Negative	LT	LT	Negative	CS12	Negative	B1	-	L20	4	14
GVE0400	SAMN42182456	SRR29657777	Bangladesh 2022-2023 (This study)	2023	Bangladesh	54	0	0	Severe	Negative	STh	ST	Negative	CFnegative	Negative	B1	-	L20	4	14
GVE0401	SAMN42182457	SRR29657776	Bangladesh 2022-2023 (This study)	2023	Bangladesh	0	6	19	None	Negative	LT+STh	LT+ST	CS2+CS3+CS21	CS2+CS3+CS21	eatA,etpBC	D	-	L28	8	36
GVE0402	SAMN42182458	SRR29657775	Bangladesh 2022-2023 (This study)	2023	Bangladesh	2	6	0	None	Negative	LT+STh	LT+ST	CS2+CS3+CS21	CS2+CS3+CS21	eatA,etpBC	A	215	L2	6	27
GVE0403	SAMN42182459	SRR29657774	Bangladesh 2022-2023 (This study)	2023	Bangladesh	0	6	19	Some	Negative	LT+STh	LT+ST	CS1+CS3+CS21	CS1+CS3+CS21	eatA,etpBC	A	215	L1	6	26
GVE0404	SAMN42182460	SRR29657773	Bangladesh 2022-2023 (This study)	2023	Bangladesh	65	0	0	Severe	Negative	STh	ST	Negative	CFnegative	etpBC	A	342	L11+L13	2	6
GVE0405	SAMN42182461	SRR29657772	Bangladesh 2022-2023 (This study)	2023	Bangladesh	28	0	0	None	Negative	STp	ST	CS6	CS6	Negative	E	1010	L7	1	1
GVE0407	SAMN42182462	SRR29657771	Bangladesh 2022-2023 (This study)	2023	Bangladesh	17	0	0	Severe	Negative	LT	LT	Negative	CS23	Negative	B1	21	L19	4	15
GVE0425	SAMN42182463	SRR29657955	Bangladesh 2022-2023 (This study)	2023	Bangladesh	0	2	7	None	Negative	LT	LT	CS5+CS6	CS5+CS6	eatA	B1	5	L5	3	8
GVE0426	SAMN42182464	SRR29657954	Bangladesh 2022-2023 (This study)	2023	Bangladesh	0	8	0	None	Negative	LT	LT	CS17	CS17	Negative	B1	-	L5	3	8
GVE0427	SAMN42182465	SRR29657953	Bangladesh 2022-2023 (This study)	2023	Bangladesh	2	9	0	Severe	Negative	STh	ST	Negative	CFA/I+CS21	eatA,etpBC	B1	-	L3	4	12
GVE0429	SAMN42182466	SRR29657952	Bangladesh 2022-2023 (This study)	2023	Bangladesh	4	0	0	Severe	Negative	LT+STh	LT+ST	Negative	CS1+CS3+CS21	eatA,etpBC	B1	-	L20	4	14
GVE0433	SAMN42182467	SRR29657951	Bangladesh 2022-2023 (This study)	2023	Bangladesh	60	0	0	None	Negative	LT+STh	LT+ST	CS5+CS6	CS5+CS6	eatA	B1	5	L5	3	8
GVE0434	SAMN42182468	SRR29657950	Bangladesh 2022-2023 (This study)	2023	Bangladesh	0	8	1	Severe	Negative	STh	ST	CFA/I+CS21	CFA/I+CS21	eatA,etpBC	A	-	L6	5	22
GVE0436	SAMN42182469	SRR29657949	Bangladesh 2022-2023 (This study)	2023	Bangladesh	0	10	0	Severe	Negative	LT	LT	Negative	CFnegative	etpC	B1	537	NA	3	10
GVE0437	SAMN42182470	SRR29657948	Bangladesh 2022-2023 (This study)	2023	Bangladesh	1	6	0	None	Ogawa	STh	ST	CS6	CS6	eatA	A	-	L11+L13	2	6
GVE0441	SAMN42182471	SRR29657947	Bangladesh 2012-2021 (This study)	2021	Bangladesh	NA	NA	NA	None	Negative	STh	ST	Negative	CS23	Negative	B1	21	L19	4	15
GVE0443	SAMN42182472	SRR29657946	Bangladesh 2012-2021 (This study)	2021	Bangladesh	NA	NA	NA	None	Negative	LT	LT	Negative	CFnegative	Negative	A	165	L11+L15	2	4
GVE0445	SAMN42182473	SRR29657944	Bangladesh 2012-2021 (This study)	2021	Bangladesh	NA	NA	NA	None	Negative	STh	ST	CS14	CS14	etpBC	B1	87	L19	4	15
GVE0446	SAMN42182474	SRR29657943	Bangladesh 2012-2021 (This study)	2021	Bangladesh	NA	NA	NA	Some	Inaba	LT+STh	LT+ST	Negative	CS23	Negative	A	-	L25	6	29
GVE0448	SAMN42182475	SRR29657942	Bangladesh 2012-2021 (This study)	2021	Bangladesh	NA	NA	NA	None	Negative	LT	LT	Negative	CFnegative	etpBC	B1	-	L23	4	17
GVE0450	SAMN42182476	SRR29657941	Bangladesh 2012-2021 (This study)	2021	Bangladesh	NA	NA	NA	None	Negative	LT	LT	Negative	CFnegative	Negative	B1	87	L19	4	15
GVE0451	SAMN42182477	SRR29657940	Bangladesh 2012-2021 (This study)	2021	Bangladesh	NA	NA	NA	severe	Negative	LT+STh	LT+ST	CS5+CS6	CS5+CS6	eatA	B1	5	L5	3	8
GVE0452	SAMN42182478	SRR29657939	Bangladesh 2012-2021 (This study)	2021	Bangladesh	NA	NA	NA	none	Negative	LT	LT	Negative	CS6	Negative	B1	-	NA	3	9
GVE0453	SAMN42182479	SRR29657938	Bangladesh 2012-2021 (This study)	2020	Bangladesh	NA	NA	NA	Severe	Negative	LT+STh	LT+ST	CS5+CS6	CS5+CS6	eatA	B1	5	L5	3	8
GVE0454	SAMN42182480	SRR29657937	Bangladesh 2012-2021 (This study)	2020	Bangladesh	NA	NA	NA	None	Negative	LT	LT	Negative	CFnegative	etpC	B1	537	NA	3	10
GVE0455	SAMN42182481	SRR29657936	Bangladesh 2012-2021 (This study)	2021	Bangladesh	NA	NA	NA	Some	Negative	LT	LT	CS5+CS6	CS26	Negative	B1	469	L23	4	17
GVE0456	SAMN42182482	SRR29657935	Bangladesh 2012-2021 (This study)	2021	Bangladesh	NA	NA	NA	None	Inaba	LT+STp	LT+ST	Negative	CS20	Negative	D	-	L28	8	36
GVE0466	SAMN42182483	SRR29657933	Bangladesh 2012-2021 (This study)	2020	Bangladesh	NA	NA	NA	Some	Negative	LT+STh	LT+ST	CS1+CS3+CS21	CFnegative	Negative	B1	471	L16	5	23
GVE0476	SAMN42182484	SRR29657932	Bangladesh 2012-2021 (This study)	2018	Bangladesh	NA	NA	NA	Severe	Negative	LT+STh	LT+ST	Negative	CFnegative	Negative	B1	471	L16	5	23
GVE0477	SAMN42182485	SRR29657931	Bangladesh 2012-2021 (This study)	2018	Bangladesh	NA	NA	NA	Severe	Negative	LT	LT	Negative	CFnegative	Negative	B1	479	L20	4	14
GVE0480	SAMN42182486	SRR29657930	Bangladesh 2012-2021 (This study)	2018	Bangladesh	NA	NA	NA	Some	Negative	LT	LT	Negative	CFnegative	Negative	A	-	L25	6	29
GVE0481	SAMN42182487	SRR29657929	Bangladesh 2012-2021 (This study)	2018	Bangladesh	NA	NA	NA	Some	Negative	LT+STh	LT+ST	Negative	CFnegative	Negative	B1	471	L16	5	23
GVE0482	SAMN42182488	SRR29657928	Bangladesh 2012-2021 (This study)	2018	Bangladesh	NA	NA	NA	Some	Negative	LT+STh	LT+ST	Negative	CFnegative	Negative	A	-	L25	6	29
GVE0483	SAMN42182489	SRR29657927	Bangladesh 2012-2021 (This study)	2018	Bangladesh	NA	NA	NA	Severe	Negative	STh	ST	Negative	CFnegative	Negative	B1	-	L27	4	20
GVE0484	SAMN42182490	SRR29657926	Bangladesh 2012-2021 (This study)	2018	Bangladesh	NA	NA	NA	Severe	Ogawa	STh	ST	Negative	CFnegative	Negative	B1	-	L20	4	14
GVE0485	SAMN42182491	SRR29657925	Bangladesh 2012-2021 (This study)	2018	Bangladesh	NA	NA	NA	Some	Negative	STp	ST	CS6	CS6	Negative	B1	357	L17	4	11
GVE0487	SAMN42182492	SRR29657924	Bangladesh 2012-2021 (This study)	2018	Bangladesh	NA	NA	NA	Severe	Negative	STh	ST	CS4	CFnegative	etpBC	A	-	L11+L13	2	6
GVE0488	SAMN42182493	SRR29657986	Bangladesh 2012-2021 (This study)	2018	Bangladesh	NA	NA	NA	Severe	Negative	STh	ST	Negative	CFnegative	etpBC	A	-	L11+L13	2	6
GVE0489	SAMN42182494	SRR29657985	Bangladesh 2012-2021 (This study)	2018	Bangladesh	NA	NA	NA	Severe	Ogawa	LT	LT	CS6+CS8	CS6+CS8	eatA	A	155	L4	6	28
GVE0491	SAMN42182495	SRR29657984	Bangladesh 2012-2021 (This study)	2018	Bangladesh	NA	NA	NA	None	Negative	STh	ST	Negative	CS21	etpBC	A	843	NA	6	31
GVE0492	SAMN42182496	SRR29657983	Bangladesh 2012-2021 (This study)	2021	Bangladesh	NA	NA	NA	Some	Inaba	STh	ST	Negative	CS23	Negative	A	-	L25	6	29
GVE0493	SAMN42182497	SRR29657982	Bangladesh 2012-2021 (This study)	2018	Bangladesh	NA	NA	NA	Severe	Negative	LT	LT	CS17	CS17	eatA+etpBC	B1	69	NA	5	24
GVE0494	SAMN42182498	SRR29657981	Bangladesh 2012-2021 (This study)	2018	Bangladesh	NA	NA	NA	Severe	Negative	STh	ST	Negative	CS21	eatA+etpBC	D	3	NA	8	37
GVE0495	SAMN42182499	SRR29657980	Bangladesh 2012-2021 (This study)	2018	Bangladesh	NA	NA	NA	Some	Negative	STh	ST	Negative	CFnegative	Negative	B1	-	L20	4	14
GVE0498	SAMN42182500	SRR29657979	Bangladesh 2012-2021 (This study)	2019	Bangladesh	NA	NA	NA	Severe	Negative	LT+STp	LT+ST	Negative	CS19	eatA+etpBC	B1	69	NA	5	24
GVE0499	SAMN42182501	SRR29657978	Bangladesh 2012-2021 (This study)	2019	Bangladesh	NA	NA	NA	Some	Negative	STh	ST	Negative	CFnegative	Negative	B1	632	L27	4	20
GVE0500	SAMN42182502	SRR29657977	Bangladesh 2012-2021 (This study)	2019	Bangladesh	NA	NA	NA	Severe	Negative	LT	LT	CS5+CS6	CS5+CS6	eatA	B1	5	L5	3	8
GVE0501	SAMN42182503	SRR29657975	Bangladesh 2012-2021 (This study)	2019	Bangladesh	NA	NA	NA	Severe	Negative	LT	LT	CS7	CS7	eatA,etpBC	B1	-	L3	4	12
GVE0502	SAMN42182504	SRR29657974	Bangladesh 2022-2023 (This study)	2023	Bangladesh	45	0	0	Some	Negative	LT	LT	Negative	CS23	eatA	B1	-	L27	4	20
GVE0503	SAMN42182505	SRR29657973	Bangladesh 2022-2023 (This study)	2023	Bangladesh	60	0	0	Severe	Negative	LT	LT	Negative	CS23	Negative	D	77	L28	8	36
GVE0504	SAMN42182506	SRR29657972	Bangladesh 2022-2023 (This study)	2023	Bangladesh	22	0	0	Severe	Negative	LT	LT	Negative	CS23	Negative	D	77	L28	8	36
GVE0505	SAMN42182507	SRR29657971	Bangladesh 2012-2021 (This study)	2019	Bangladesh	NA	NA	NA	Some	Negative	STp	ST	CS6	CS6	Negative	B1	357	L17	4	11
GVE0506	SAMN42182508	SRR29657970	Bangladesh 2012-2021 (This study)	2019	Bangladesh	NA	NA	NA	Severe	Negative	LT+STh	LT+ST	CFA/I	CS14	etpBC	B1	357	L17	4	11
GVE0507	SAMN42182509	SRR29657969	Bangladesh 2012-2021 (This study)	2019	Bangladesh	NA	NA	NA	Severe	Negative	LT+STh	LT+ST	CS5+CS6	CS5+CS6	eatA	B1	-	L5	3	8
GVE0508	SAMN42182510	SRR29657968	Bangladesh 2012-2021 (This study)	2019	Bangladesh	NA	NA	NA	Severe	Negative	LT+STh	LT+ST	Negative	CFnegative	Negative	B1	479	L20	4	14
GVE0509	SAMN42182511	SRR29657967	Bangladesh 2012-2021 (This study)	2019	Bangladesh	NA	NA	NA	Some	Negative	LT	LT	CS17	CS17	eatA+etpBC	B1	5	L5	3	8
GVE0543	SAMN42182512	SRR29657966	Bangladesh 2012-2021 (This study)	2017	Bangladesh	NA	NA	NA	Severe	Negative	STh	ST	Negative	CFnegative	Negative	A	-	L25	6	29
GVE0546	SAMN42182513	SRR29657964	Bangladesh 2012-2021 (This study)	2016	Bangladesh	NA	NA	NA	None	Negative	STh	ST	CS2+CS3+CS21	CS14	eatA,etpBC	B1	-	L19	4	15
GVE0549	SAMN42182514	SRR29657963	Bangladesh 2012-2021 (This study)	2016	Bangladesh	NA	NA	NA	Some	Negative	LT+STh	LT+ST	CS1+CS3+CS21	CS1+CS3+CS21	eatA+etpBC	A	215	L1	6	26
GVE0551	SAMN42182515	SRR29657962	Bangladesh 2012-2021 (This study)	2017	Bangladesh	NA	NA	NA	Some	Negative	LT	LT	CS5+CS6	CS5+CS6	eatA	B1	5	L5	3	8
GVE0556	SAMN42182516	SRR29657961	Bangladesh 2012-2021 (This study)	2016	Bangladesh	NA	NA	NA	None	Negative	LT	LT	Negative	CS6	etpBC	B1	-	L20	4	14
GVE0562	SAMN42182517	SRR29657960	Bangladesh 2022-2023 (This study)	2023	Bangladesh	62	0	0	Some	Negative	STh	ST	Negative	CS23	Negative	B1	21	L19	4	15
GVE0569	SAMN42182518	SRR29657959	Bangladesh 2012-2021 (This study)	2014	Bangladesh	NA	NA	NA	Some	Negative	LT	LT	CS1+CS3+CS21	CS1+CS3+CS21	eatA+etpBC	A	215	L1	6	26
GVE0588	SAMN42182519	SRR29657958	Bangladesh 2012-2021 (This study)	2014	Bangladesh	NA	NA	NA	Severe	Negative	LT+STp	LT+ST	Negative	CS12	Negative	B1	86	L18	4	16
GVE0627	SAMN42182520	SRR29657957	Bangladesh 2022-2023 (This study)	2023	Bangladesh	1	0	21	Some	Negative	LT	LT	Negative	CFnegative	Negative	B1	108	L26	4	19
GVE0628	SAMN42182521	SRR29657956	Bangladesh 2022-2023 (This study)	2023	Bangladesh	0	8	0	Severe	Negative	STh	ST	CFA/I+CS21	CFnegative	Negative	NA	-	NA	6	32
GVE0631	SAMN42182522	SRR29657640	Bangladesh 2012-2021 (This study)	2014	Bangladesh	NA	NA	NA	None	Negative	LT+STh	LT+ST	CS14	CS14	etpBC	B1	357	L17	4	11
GVE0632	SAMN42182523	SRR29657638	Bangladesh 2012-2021 (This study)	2014	Bangladesh	NA	NA	NA	None	Negative	STp	ST	CS6	CS6	Negative	E	-	NA	1	2
GVE0633	SAMN42182524	SRR29657637	Bangladesh 2012-2021 (This study)	2014	Bangladesh	NA	NA	NA	Severe	Negative	LT+STh	LT+ST	Negative	CS21	etpBC	A	155	L4	6	28
GVE0634	SAMN42182525	SRR29657636	Bangladesh 2012-2021 (This study)	2014	Bangladesh	NA	NA	NA	Some	Negative	LT+STh	LT+ST	CS5+CS6	CFnegative	Negative	B1	19	L20	4	14
GVE0635	SAMN42182526	SRR29657635	Bangladesh 2012-2021 (This study)	2014	Bangladesh	NA	NA	NA	None	Negative	LT+STh	LT+ST	CS2+CS3	CS2+CS3+CS21	eatA+etpBC	A	215	L2	6	27
GVE0636	SAMN42182527	SRR29657634	Bangladesh 2022-2023 (This study)	2023	Bangladesh	23	0	0	Severe	Ogawa	LT+STh	LT+ST	Negative	CFnegative	Negative	B1	-	L20	4	14
GVE0637	SAMN42182528	SRR29657633	Bangladesh 2022-2023 (This study)	2023	Bangladesh	48	0	0	None	Negative	STp	ST	CS6	CS6	Negative	A	-	L11+L15	2	4
GVE0638	SAMN42182529	SRR29657632	Bangladesh 2012-2021 (This study)	2017	Bangladesh	NA	NA	NA	Some	Negative	STh	ST	Negative	CFnegative	Negative	B1	21	L19	4	15
GVE0639	SAMN42182530	SRR29657631	Bangladesh 2022-2023 (This study)	2023	Bangladesh	20	0	0	Severe	Ogawa	LT+STh	LT+ST	Negative	CS23	Negative	A	-	L11+L13	2	6
GVE0640	SAMN42182531	SRR29657630	Bangladesh 2022-2023 (This study)	2023	Bangladesh	30	0	0	Severe	Negative	LT	LT	Negative	CS23	Negative	D	77	L28	8	36
GVE0641	SAMN42182532	SRR29657629	Bangladesh 2012-2021 (This study)	2014	Bangladesh	NA	NA	NA	Severe	Negative	STp	ST	Negative	CS6	Negative	E	-	NA	1	2
GVE0642	SAMN42182533	SRR29657627	Bangladesh 2012-2021 (This study)	2017	Bangladesh	NA	NA	NA	Severe	Negative	LT+STh	LT+ST	Negative	CFnegative	Negative	B1	108	L26	4	19
GVE0643	SAMN42182534	SRR29657626	Bangladesh 2022-2023 (This study)	2023	Bangladesh	0	8	0	None	Negative	LT	LT	CS21	CFnegative	etpBC	B1	479	L20	4	14
GVE0644	SAMN42182535	SRR29657625	Bangladesh 2022-2023 (This study)	2023	Bangladesh	0	7	0	Severe	Negative	LT+STh	LT+ST	Negative	CS2+CS3+CS21	eatA,etpBC	A	-	L2	6	27
GVE0645	SAMN42182536	SRR29657624	Bangladesh 2012-2021 (This study)	2014	Bangladesh	NA	NA	NA	Severe	Negative	LT	LT	Negative	CFnegative	Negative	B1	-	L17	4	11
GVE0646	SAMN42182537	SRR29657623	Bangladesh 2012-2021 (This study)	2015	Bangladesh	NA	NA	NA	Severe	Ogawa	STh	ST	Negative	CFnegative	Negative	B1	471	L16	5	23
GVE0647	SAMN42182538	SRR29657622	Bangladesh 2022-2023 (This study)	2023	Bangladesh	26	0	0	Severe	Negative	LT+STh	LT+ST	Negative	CS23	Negative	B1	632	L27	4	20
GVE0648	SAMN42182539	SRR29657621	Bangladesh 2022-2023 (This study)	2023	Bangladesh	34	0	0	Some	Negative	LT	LT	CS6+CS8	CS6+CS8	eatA	A	-	L4	6	28
GVE0649	SAMN42182540	SRR29657620	Bangladesh 2012-2021 (This study)	2015	Bangladesh	NA	NA	NA	Some	Negative	LT+STp	LT+ST	CS4+CS6	CS6	eatA	A	-	NA	2	5
GVE0650	SAMN42182541	SRR29657619	Bangladesh 2012-2021 (This study)	2014	Bangladesh	NA	NA	NA	Severe	Negative	LT+STh	LT+ST	Negative	CFnegative	Negative	B1	632	L27	4	20
GVE0651	SAMN42182542	SRR29657618	Bangladesh 2012-2021 (This study)	2014	Bangladesh	NA	NA	NA	None	Ogawa	LT+STh	LT+ST	Negative	CFnegative	Negative	B1	87	L19	4	15
GVE0653	SAMN42182543	SRR29657616	Bangladesh 2022-2023 (This study)	2023	Bangladesh	1	1	0	None	Negative	STp	ST	CS6	CS6	Negative	E	1010	L7	1	1
GVE0654	SAMN42182544	SRR29657615	Bangladesh 2022-2023 (This study)	2023	Bangladesh	1	6	0	Some	Negative	LT+STp	LT+ST	Negative	CS20	eatA	F	-	NA	7	33
GVE0655	SAMN42182545	SRR29657614	Bangladesh 2022-2023 (This study)	2023	Bangladesh	1	0	0	None	Negative	LT	LT	Negative	CFnegative	eatA	A	-	NA	6	30
GVE0656	SAMN42182546	SRR29657613	Bangladesh 2022-2023 (This study)	2023	Bangladesh	0	10	0	None	Negative	LT	LT	CS2+CS3+CS21	CS2+CS3+CS21	eatA,etpBC	A	215	L2	6	27
GVE0657	SAMN42182547	SRR29657612	Bangladesh 2012-2021 (This study)	2015	Bangladesh	NA	NA	NA	Some	Negative	LT	LT	Negative	CS14	etpBC	B1	357	L17	4	11
GVE0658	SAMN42182548	SRR29657611	Bangladesh 2012-2021 (This study)	2015	Bangladesh	NA	NA	NA	Some	Negative	LT+STp	LT+ST	Negative	CS20	Negative	D	-	L28	8	36
GVE0659	SAMN42182549	SRR29657610	Bangladesh 2012-2021 (This study)	2015	Bangladesh	NA	NA	NA	Severe	Negative	LT+STh	LT+ST	Negative	CFnegative	Negative	B1	632	L27	4	20
GVE0660	SAMN42182550	SRR29657609	Bangladesh 2012-2021 (This study)	2015	Bangladesh	NA	NA	NA	Severe	Ogawa	LT+STh	LT+ST	Negative	CFnegative	Negative	B1	632	L27	4	20
GVE0661	SAMN42182551	SRR29657768	Bangladesh 2012-2021 (This study)	2015	Bangladesh	NA	NA	NA	None	Negative	LT	LT	CFA/I	CFnegative	Negative	B1	537	NA	3	10
GVE0662	SAMN42182552	SRR29657767	Bangladesh 2012-2021 (This study)	2015	Bangladesh	NA	NA	NA	Some	Negative	STh	ST	Negative	CFnegative	Negative	B1	471	L16	5	23
GVE0663	SAMN42182553	SRR29657765	Bangladesh 2022-2023 (This study)	2023	Bangladesh	60	0	0	Some	Negative	LT+STh	LT+ST	Negative	CS23	Negative	A	637	L11+L13	2	6
GVE0664	SAMN42182554	SRR29657764	Bangladesh 2012-2021 (This study)	2014	Bangladesh	NA	NA	NA	Severe	Negative	LT+STh	LT+ST	CS6	CFnegative	Negative	B1	19	L20	4	14
GVE0665	SAMN42182555	SRR29657763	Bangladesh 2022-2023 (This study)	2023	Bangladesh	23	0	0	Severe	Negative	STh	ST	Negative	CS23	Negative	B1	471	L16	5	23
GVE0666	SAMN42182556	SRR29657762	Bangladesh 2012-2021 (This study)	2020	Bangladesh	NA	NA	NA	Severe	Negative	LT+STh	LT+ST	Negative	CS23	Negative	D	77	L28	8	36
GVE0667	SAMN42182557	SRR29657761	Bangladesh 2012-2021 (This study)	2015	Bangladesh	NA	NA	NA	Severe	Negative	LT	LT	Negative	CS6	Negative	B1	956	L18	4	16
GVE0668	SAMN42182558	SRR29657760	Bangladesh 2012-2021 (This study)	2014	Bangladesh	NA	NA	NA	Some	Negative	LT+STh	LT+ST	Negative	CFnegative	Negative	A	-	L25	6	29
GVE0669	SAMN42182559	SRR29657759	Bangladesh 2012-2021 (This study)	2014	Bangladesh	NA	NA	NA	Some	Negative	LT	LT	Negative	CFnegative	etpBC	B1	537	NA	3	10
GVE0670	SAMN42182560	SRR29657758	Bangladesh 2012-2021 (This study)	2014	Bangladesh	NA	NA	NA	Some	Negative	LT+STh	LT+ST	Negative	CFnegative	Negative	B1	19	L20	4	14
GVE0696	SAMN42182561	SRR29657757	Bangladesh 2012-2021 (This study)	2016	Bangladesh	NA	NA	NA	Severe	Negative	LT+STh	LT+ST	CS5+CS6	CS5+CS6	eatA	B1	5	L5	3	8
GVE0697	SAMN42182562	SRR29657756	Bangladesh 2012-2021 (This study)	2016	Bangladesh	NA	NA	NA	Some	Negative	STh	ST	CS14	CS14	etpBC	B1	-	L19	4	15
GVE0698	SAMN42182563	SRR29657753	Bangladesh 2012-2021 (This study)	2017	Bangladesh	NA	NA	NA	Severe	Negative	LT	LT	CS17	CS17	Negative	B1	-	L5	3	8
GVE0699	SAMN42182564	SRR29657752	Bangladesh 2022-2023 (This study)	2023	Bangladesh	23	0	0	Severe	Inaba	LT+STh	LT+ST	Negative	CS23	Negative	D	77	L28	8	36

GVE0700	SAMN42182565	SRR29657751	Bangladesh 2022-2023 (This study)	2023	Bangladesh	15	0	0	Severe	Inaba	LT+STh	LT+ST	Negative	CS23	Negative	D	77	L28	8	36
GVE0701	SAMN42182566	SRR29657750	Bangladesh 2022-2023 (This study)	2023	Bangladesh	30	0	0	Severe	Negative	LT+STh	LT+ST	Negative	CS23	Negative	B1	471	L16	5	23
GVE0702	SAMN42182567	SRR29657749	Bangladesh 2012-2021 (This study)	2017	Bangladesh	NA	NA	NA	Severe	Negative	LT	LT	CS5+CS6	CS5+CS6	eatA	B1	5	L5	3	8
GVE0703	SAMN42182568	SRR29657748	Bangladesh 2012-2021 (This study)	2016	Bangladesh	NA	NA	NA	Severe	Negative	LT	LT	Negative	CFnegative	Negative	B1	471	L16	5	23
GVE0704	SAMN42182569	SRR29657747	Bangladesh 2012-2021 (This study)	2020	Bangladesh	NA	NA	NA	Some	Negative	LT+STh	LT+ST	Negative	CFnegative	Negative	B1	471	L16	5	23
GVE0705	SAMN42182570	SRR29657746	Bangladesh 2012-2021 (This study)	2016	Bangladesh	NA	NA	NA	Some	Negative	LT+STh	LT+ST	CS5+CS6	CS5+CS6	eatA	B1	5	L5	3	8
GVE0706	SAMN42182571	SRR29657745	Bangladesh 2012-2021 (This study)	2017	Bangladesh	NA	NA	NA	Severe	Negative	LT	LT	CS7	CS17	eatA,etpBC	B1	69	NA	5	24
GVE0707	SAMN42182572	SRR29657744	Bangladesh 2022-2023 (This study)	2023	Bangladesh	0	7	0	Severe	Negative	LT	LT	Negative	CS23	Negative	B1	632	L27	4	20
GVE0708	SAMN42182573	SRR29657742	Bangladesh 2012-2021 (This study)	2017	Bangladesh	NA	NA	NA	Severe	Negative	LT+STh	LT+ST	CS1+CS3+CS21	CS1+CS3+CS21	eatA+etpBC	A	215	L1	6	26
GVE0709	SAMN42182574	SRR29657741	Bangladesh 2022-2023 (This study)	2023	Bangladesh	30	0	0	Severe	Negative	LT+STh	LT+ST	Negative	CS23	Negative	B1	-	L20	4	14
GVE0710	SAMN42182575	SRR29657740	Bangladesh 2012-2021 (This study)	2017	Bangladesh	NA	NA	NA	Some	Negative	LT	LT	CS7	CS7	eatA,etpBC	B1	-	L3	4	12
GVE0711	SAMN42182576	SRR29657739	Bangladesh 2012-2021 (This study)	2013	Bangladesh	NA	NA	NA	Severe	Negative	LT+STh	LT+ST	CS14	CS14	etpBC	B1	357	L17	4	11
GVE0712	SAMN42182577	SRR29657738	Bangladesh 2012-2021 (This study)	2016	Bangladesh	NA	NA	NA	Severe	Negative	LT+STh	LT+ST	CS2+CS3	CS2+CS3+CS21	eatA,etpBC	NA	-	NA	9	42
GVE0713	SAMN42182578	SRR29657737	Bangladesh 2012-2021 (This study)	2016	Bangladesh	NA	NA	NA	None	Negative	LT+STp	LT+ST	Negative	CS12	Negative	B1	86	L18	4	16
GVE0714	SAMN42182579	SRR29657608	Bangladesh 2012-2021 (This study)	2016	Bangladesh	NA	NA	NA	Some	Negative	LT	LT	Negative	CFnegative	etpBC	B1	537	NA	3	10
GVE0715	SAMN42182580	SRR29657607	Bangladesh 2012-2021 (This study)	2016	Bangladesh	NA	NA	NA	Severe	Negative	LT	LT	CS17	CS17	eatA,etpBC	B1	69	NA	5	24
GVE0716	SAMN42182581	SRR29657606	Bangladesh 2012-2021 (This study)	2016	Bangladesh	NA	NA	NA	Some	Inaba	LT+STp	LT+ST	Negative	CS12	Negative	B1	86	L18	4	16
GVE0717	SAMN42182582	SRR29657605	Bangladesh 2022-2023 (This study)	2023	Bangladesh	24	0	0	Severe	Ogawa	STh	ST	Negative	CS23	Negative	B1	632	L27	4	20
GVE0718	SAMN42182583	SRR29657603	Bangladesh 2012-2021 (This study)	2013	Bangladesh	NA	NA	NA	None	Negative	LT	LT	CS1+CS3+CS21	CS1+CS3+CS21	eatA,etpBC	A	215	L1	6	26
GVE0719	SAMN42182584	SRR29657602	Bangladesh 2012-2021 (This study)	2013	Bangladesh	NA	NA	NA	Severe	Negative	LT+STp	LT+ST	CS4+CS6	CS6	eatA	A	-	NA	2	5
GVE0720	SAMN42182585	SRR29657601	Bangladesh 2022-2023 (This study)	2023	Bangladesh	17	0	0	Severe	Negative	LT	LT	Negative	CS23	Negative	B1	108	L26	4	19
GVE0721	SAMN42182586	SRR29657600	Bangladesh 2012-2021 (This study)	2013	Bangladesh	NA	NA	NA	Some	Negative	LT+STh	LT+ST	Negative	CFnegative	Negative	A	-	L25	6	29
GVE0722	SAMN42182587	SRR29657599	Bangladesh 2012-2021 (This study)	2013	Bangladesh	NA	NA	NA	None	Negative	LT	LT	Negative	CS6+CS8	eatA	A	155	L4	6	28
GVE0723	SAMN42182588	SRR29657598	Bangladesh 2012-2021 (This study)	2013	Bangladesh	NA	NA	NA	None	Ogawa	LT+STh	LT+ST	Negative	CFnegative	Negative	B1	632	L27	4	20
GVE0724	SAMN42182589	SRR29657597	Bangladesh 2012-2021 (This study)	2015	Bangladesh	NA	NA	NA	Some	Negative	STh	ST	Negative	CFnegative	eatA	A	681	L11+L13	2	6
GVE0725	SAMN42182590	SRR29657596	Bangladesh 2012-2021 (This study)	2013	Bangladesh	NA	NA	NA	None	Negative	STh	ST	CS5+CS6	CS5+CS6	eatA	B1	5	L5	3	8
GVE0726	SAMN42182591	SRR29657595	Bangladesh 2012-2021 (This study)	2013	Bangladesh	NA	NA	NA	Some	Negative	LT+STh	LT+ST	Negative	CFnegative	Negative	A	-	L25	6	29
GVE0727	SAMN42182592	SRR29657594	Bangladesh 2022-2023 (This study)	2023	Bangladesh	1	1	0	Severe	Negative	LT	LT	Negative	CS23	Negative	A	637	L11+L13	2	6
GVE0728	SAMN42182593	SRR29657592	Bangladesh 2012-2021 (This study)	2013	Bangladesh	NA	NA	NA	None	Negative	LT	LT	CS7	CS7	eatA,etpBC	B1	-	L3	4	12
GVE0729	SAMN42182594	SRR29657591	Bangladesh 2012-2021 (This study)	2013	Bangladesh	NA	NA	NA	Some	Negative	LT	LT	Negative	CS6	Negative	B1	956	L18	4	16
GVE0730	SAMN42182595	SRR29657590	Bangladesh 2012-2021 (This study)	2013	Bangladesh	NA	NA	NA	Some	Negative	LT+STh	LT+ST	Negative	CFnegative	Negative	B1	632	L27	4	20
GVE0731	SAMN42182596	SRR29657589	Bangladesh 2022-2023 (This study)	2023	Bangladesh	0	6	9	Severe	Ogawa	LT	LT	Negative	CS23	Negative	B1	632	L27	4	20
GVE0736	SAMN42182597	SRR29657588	Bangladesh 2012-2021 (This study)	2017	Bangladesh	NA	NA	NA	Severe	Negative	LT+STh	LT+ST	CS5+CS6	CS5+CS6	eatA	B1	5	L5	3	8
GVE0737	SAMN42182598	SRR29657587	Bangladesh 2012-2021 (This study)	2017	Bangladesh	NA	NA	NA	Severe	Negative	STp	ST	CS6	CS6	Negative	E	-	NA	1	2
GVE0738	SAMN42182599	SRR29657586	Bangladesh 2012-2021 (This study)	2019	Bangladesh	NA	NA	NA	Severe	Ogawa	LT+STh	LT+ST	Negative	CFnegative	Negative	B1	87	L19	4	15
GVE0739	SAMN42182600	SRR29657585	Bangladesh 2012-2021 (This study)	2019	Bangladesh	NA	NA	NA	None	Ogawa	LT+STh	LT+ST	CS1+CS3+CS21	CS1+CS3+CS21	eatA,etpBC	A	215	L1	6	26
GVE0740	SAMN42182601	SRR29657584	Bangladesh 2012-2021 (This study)	2020	Bangladesh	NA	NA	NA	Severe	Negative	LT+STh	LT+ST	Negative	CS23	Negative	B1	632	L27	4	20
GVE0741	SAMN42182602	SRR29657583	Bangladesh 2012-2021 (This study)	2020	Bangladesh	NA	NA	NA	Some	Negative	LT+STh	LT+ST	Negative	CS23	Negative	A	-	L25	6	29
GVE0742	SAMN42182603	SRR29657581	Bangladesh 2012-2021 (This study)	2020	Bangladesh	NA	NA	NA	None	Negative	LT	LT	Negative	CFnegative	etpBC	B1	-	NA	5	25
GVE0743	SAMN42182604	SRR29657580	Bangladesh 2012-2021 (This study)	2020	Bangladesh	NA	NA	NA	None	Negative	LT	LT	Negative	CS23	Negative	B1	632	L27	4	20
GVE0744	SAMN42182605	SRR29657579	Bangladesh 2012-2021 (This study)	2021	Bangladesh	NA	NA	NA	Some	Inaba	STp	ST	CFA/I	CS6	Negative	E	-	L7	1	1
GVE0745	SAMN42182606	SRR29657578	Bangladesh 2012-2021 (This study)	2021	Bangladesh	NA	NA	NA	Some	Inaba	LT+STh	LT+ST	Negative	CS23	Negative	B1	108	L26	4	19
GVE0746	SAMN42182607	SRR29657577	Bangladesh 2012-2021 (This study)	2020	Bangladesh	NA	NA	NA	Severe	Negative	STh	ST	CS14	CS14	etpBC	B1	87	L19	4	15
GVE0747	SAMN42182608	SRR29657576	Bangladesh 2022-2023 (This study)	2023	Bangladesh	55	0	0	Severe	Inaba	STh	ST	Negative	CS23	Negative	B1	108	L26	4	19
GVE0748	SAMN42182609	SRR29657575	Bangladesh 2012-2021 (This study)	2020	Bangladesh	NA	NA	NA	Some	Negative	LT	LT	Negative	CS23	Negative	B1	632	L27	4	20
GVE0749	SAMN42182610	SRR29657574	Bangladesh 2012-2021 (This study)	2021	Bangladesh	NA	NA	NA	Severe	Negative	STh	ST	Negative	CS23	Negative	B1	21	L19	4	15
GVE0751	SAMN42182611	SRR29657573	Bangladesh 2012-2021 (This study)	2016	Bangladesh	NA	NA	NA	Some	Negative	LT+STh	LT+ST	Negative	CFnegative	Negative	A	-	L25	6	29
GVE0752	SAMN42182612	SRR29657572	Bangladesh 2012-2021 (This study)	2015	Bangladesh	NA	NA	NA	Some	Negative	LT+STh	LT+ST	Negative	CFnegative	Negative	B1	471	L16	5	23
GVE0753	SAMN42182613	SRR29657570	Bangladesh 2012-2021 (This study)	2020	Bangladesh	NA	NA	NA	Severe	Negative	LT	LT	CS17	CS17	eatA,etpBC	B1	5	L5	3	8
GVE0754	SAMN42182614	SRR29657569	Bangladesh 2012-2021 (This study)	2015	Bangladesh	NA	NA	NA	Some	Negative	LT+STh	LT+ST	Negative	CFnegative	Negative	B1	471	L16	5	23
GVE0757	SAMN42182615	SRR29657568	Bangladesh 2012-2021 (This study)	2019	Bangladesh	NA	NA	NA	Severe	Ogawa	LT+STh	LT+ST	Negative	CFnegative	Negative	B1	-	L20	4	14
GVE0758	SAMN42182616	SRR29657567	Bangladesh 2012-2021 (This study)	2019	Bangladesh	NA	NA	NA	Severe	Ogawa	LT	LT	CS6,CS14	CS14	etpBC	B1	357	L17	4	11
GVE0759	SAMN42182617	SRR29657566	Bangladesh 2012-2021 (This study)	2019	Bangladesh	NA	NA	NA	Severe	Negative	LT	LT	CS5+CS6	CS5+CS6	eatA	B1	5	L5	3	8
GVE0760	SAMN42182618	SRR29657565	Bangladesh 2012-2021 (This study)	2017	Bangladesh	NA	NA	NA	None	Negative	LT	LT	CS1+CS3+CS21	CS1+CS3+CS21	eatA,etpBC	A	215	L1	6	26
GVE0761	SAMN42182619	SRR29657564	Bangladesh 2012-2021 (This study)	2021	Bangladesh	NA	NA	NA	Some	Negative	LT+STh	LT+ST	Negative	CS23	Negative	B1	632	L27	4	20
GVE0762	SAMN42182620	SRR29657563	Bangladesh 2012-2021 (This study)	2015	Bangladesh	NA	NA	NA	Some	Inaba	STh	ST	Negative	CFnegative	Negative	B1	632	L27	4	20
GVE0763	SAMN42182621	SRR29657562	Bangladesh 2012-2021 (This study)	2020	Bangladesh	NA	NA	NA	Some	Negative	LT	LT	Negative	CS23	Negative	D	77	L28	8	36
GVE0764	SAMN42182622	SRR29657561	Bangladesh 2012-2021 (This study)	2020	Bangladesh	NA	NA	NA	Severe	Inaba	LT+STh	LT+ST	Negative	CS23	Negative	D	77	L28	8	36
GVE0770	SAMN42182623	SRR29657559	Bangladesh 2012-2021 (This study)	2012	Bangladesh	NA	NA	NA	Severe	Ogawa	LT+STh	LT+ST	Negative	CFnegative	Negative	A	-	L25	6	29
GVE0771	SAMN42182624	SRR29657558	Bangladesh 2012-2021 (This study)	2012	Bangladesh	NA	NA	NA	None	Negative	LT+STp	LT+ST	CS12	CS12	Negative	D	535	L29	9	41
GVE0772	SAMN42182625	SRR29657557	Bangladesh 2012-2021 (This study)	2012	Bangladesh	NA	NA	NA	Some	Negative	LT+STh	LT+ST	Negative	CFnegative	Negative	A	-	L25	6	29
GVE0773	SAMN42182626	SRR29657556	Bangladesh 2012-2021 (This study)	2012	Bangladesh	NA	NA	NA	Some	Negative	LT+STh	LT+ST	CS5+CS6	CS5+CS6	eatA	B1	5	L5	3	8
GVE0774	SAMN42182627	SRR29657555	Bangladesh 2012-2021 (This study)	2012	Bangladesh	NA	NA	NA	Severe	Negative	LT+STh	LT+ST	CS1+CS3+CS21	CS1+CS3+CS21	eatA,etpBC	A	215	L1	6	26
GVE0775	SAMN42182628	SRR29657554	Bangladesh 2012-2021 (This study)	2012	Bangladesh	NA	NA	NA	Some	Negative	LT	LT	CS17	CS17	eatA,etpBC	B1	69	NA	5	24
GVE0776	SAMN42182629	SRR29657553	Bangladesh 2022-2023 (This study)	2023	Bangladesh	50	0	0	Severe	Ogawa	LT+STh	LT+ST	Negative	CS23	Negative	A	637	L11+L13	2	6
GVE0777	SAMN42182630	SRR29657552	Bangladesh 2012-2021 (This study)	2012	Bangladesh	NA	NA	NA	Severe	Negative	LT	LT	Negative	CFnegative	Negative	B1	537	NA	3	10
GVE0778	SAMN42182631	SRR29657551	Bangladesh 2022-2023 (This study)	2023	Bangladesh	62	0	0	Severe	Negative	STh	ST	Negative	CS23	Negative	A	-	L11+L13	2	6
GVE0779	SAMN42182632	SRR29657550	Bangladesh 2012-2021 (This study)	2012	Bangladesh	NA	NA	NA	None	Negative	LT+STh	LT+ST	CS1+CS3+CS21	CS1+CS3+CS21	eatA,etpBC	A	215	L1	6	26
GVE0780	SAMN42182633	SRR29657548	Bangladesh 2012-2021 (This study)	2012	Bangladesh	NA	NA	NA	Some	Negative	LT+STp	LT+ST	CS12	CS12	Negative	D	535	L29	9	41
GVE0781	SAMN42182634	SRR29657547	Bangladesh 2012-2021 (This study)	2012	Bangladesh	NA	NA	NA	None	Ogawa	STh	ST	Negative	CFnegative	Negative	B1	-	L20	4	14
GVE0782	SAMN42182635	SRR29657546	Bangladesh 2012-2021 (This study)	2012	Bangladesh	NA	NA	NA	Severe	Negative	STh	ST	CS4	CFnegative	etpBC	A	843	NA	6	31
GVE0783	SAMN42182636	SRR29657545	Bangladesh 2012-2021 (This study)	2012	Bangladesh	NA	NA	NA	Some	Negative	LT	LT	CS7	CS7	eatA,etpBC	B1	-	L3	4	12
GVE0784	SAMN42182637	SRR29657923	Bangladesh 2012-2021 (This study)	2012	Bangladesh	NA	NA	NA	Some	Negative	LT+STh	LT+ST	Negative	CFnegative	Negative	A	-	L25	6	29
E110	NA	NA	Bangladesh 1980-2011 (Mentzer et al)	1985	Bangladesh	NA	NA	NA	NA	NA	STh	ST	NA	CS5+CS6	eatA	B1	5	L5	3	8
E1712	NA	NA	Bangladesh 1980-2011 (Mentzer et al)	2001	Bangladesh	NA	NA	NA	NA	NA	LT	LT	NA	CS14	Negative	B1	357	L17	4	11
E1716	NA	NA	Bangladesh 1980-2011 (Mentzer et al)	2001	Bangladesh	NA	NA	NA	NA	NA	STh	ST	NA	CFA/I+CS21	etpBC	B1	-	L3	4	12
E1724	NA	NA	Bangladesh 1980-2011 (Mentzer et al)	2001	Bangladesh	NA	NA	NA	NA	NA	LT+STh	LT+ST	NA	CS5+CS6	eatA	B1	5	L5	3	8
E1735	NA	NA	Bangladesh 1980-2011 (Mentzer et al)	2002	Bangladesh	NA	NA	NA	NA	NA	LT+STh	LT+ST	NA	CS14	Negative	B1	357	L17	4	11
E1736	NA	NA	Bangladesh 1980-2011 (Mentzer et al)	2002	Bangladesh	NA	NA	NA	NA	NA	LT+STh	LT+ST	NA	CS14	eatA,etpBC	B1	357	L17	4	11
E1739	NA	NA	Bangladesh 1980-2011 (Mentzer et al)	2002	Bangladesh	NA	NA	NA	NA	NA	LT+STh	LT+ST	NA	CS1+CS3+CS21	Negative	A	215	L1	6	26
E1741	NA	NA	Bangladesh 1980-2011 (Mentzer et al)	2002	Bangladesh	NA	NA	NA	NA	NA	LT+ST	LT+ST	NA	CS17	eatA	B1	5	L5	3	8
E1744	NA	NA	Bangladesh 1980-2011 (Mentzer et al)	2002	Bangladesh	NA	NA	NA	NA	NA	LT	LT	NA	CFnegative	eatA	B1	-	L17	4	11
E1750	NA	NA	Bangladesh 1980-2011 (Mentzer et al)	2002	Bangladesh	NA	NA	NA	NA	NA	LT+STh	LT+ST	NA	CS5+CS6	eatA,etpBC	B1	5	L5	3	8
E1752	NA	NA	Bangladesh 1980-2011 (Mentzer et al)	2002	Bangladesh	NA	NA	NA	NA	NA	STh	ST	NA	CS6	eatA	A	155	L4	6	28
E1760	NA	NA	Bangladesh 1980-2011 (Mentzer et al)	2002	Bangladesh	NA	NA	NA	NA	NA	LT+STh	LT+ST	NA	CS5+CS6	eatA,etpBC	B1	5	L5	3	8
E1779	NA	NA	Bangladesh 1980-2011 (Mentzer et al)	2005	Bangladesh	NA	NA	NA	NA	NA	LT+STh	LT+ST	NA	CS5+CS6	etpBC	B1	5	L5	3	8
E1784	NA	NA	Bangladesh 1980-2011 (Mentzer et al)	2005	Bangladesh	NA	NA	NA	NA	NA	STh	ST	NA	CS6	Negative	B1	-	L20	4	14
E1795	NA	NA	Bangladesh 1980-2011 (Mentzer et al)	2007	Bangladesh	NA	NA	NA	NA	NA	STh	ST	NA	CFA/I+CS21	eatA,etpABC	A	2	L11+L15	2	4
E1797	NA	NA	Bangladesh 1980-2011 (Mentzer et al)	2007	Bangladesh	NA	NA	NA	NA	NA	LT+STh	LT+ST	NA	CS1+CS3+CS21	Negative	A	215	L1	6	26
E1841	NA	NA	Bangladesh 1980-2011 (Mentzer et al)	2007	Bangladesh	NA	NA	NA	NA	NA	STh	ST	NA	CFA/I+CS21	Negative	B1	-	L3	4	12
E1871	NA	NA	Bangladesh 1980-2011 (Mentzer et al)	2007	Bangladesh	NA	NA	NA	NA	NA	LT+STh	LT+ST	NA	CS14	eatA	B1	357	L17	4	11
E1883	NA	NA	Bangladesh 1980-2011 (Mentzer et al)	2007	Bangladesh	NA	NA	NA	NA	NA	LT+STh	LT+ST	NA	CS2+CS3+CS21	eatA	A	215	L2	6	27
E1918	NA	NA	Bangladesh 1980-2011 (Mentzer et al)	2007	Bangladesh	NA	NA	NA	NA	NA	LT+STh	LT+ST	NA	CS5+CS6	Negative	B1	5	L5	3	8
E1939	NA	NA	Bangladesh 1980-2011 (Mentzer et al)	2007	Bangladesh	NA	NA	NA	NA	NA	LT	LT	NA	CS7	Negative	B1	-	L3	4	12
E1947	NA	NA	Bangladesh 1980-2011 (Mentzer et al)	2007	Bangladesh	NA	NA	NA	NA	NA	STh	ST	NA	CFA/I+CS21	eatA,etpABC	B1	-	L3	4	12
E1961	NA	NA	Bangladesh 1980-2011 (Mentzer et al)	2007	Bangladesh	NA	NA	NA	NA	NA	LT+STp	LT+ST	NA	CS19	Negative	B1	69	NA	5	24
E1994	NA	NA	Bangladesh 1980-2011 (Mentzer et al)	2007	Bangladesh	NA	NA	NA	NA	NA	LT	LT	NA	CS17	eatA,etpABC	B1	5	L5	3	8
E2088	NA	NA	Bangladesh 1980-2011 (Mentzer et al)	2007	Bangladesh	NA	NA	NA	NA	NA	LT+STh	LT+ST	NA	CFA/I+CS21	eatA,etpBC	B1	-	L3	4	12
E2092	NA	NA	Bangladesh 1980-2011 (Mentzer et al)	2007	Bangladesh	NA	NA	NA	NA	NA	LT+STh	LT+ST	NA	CS5+CS6	eatA,etpBC	B1	5	L5	3	8
E21	NA	NA	Bangladesh 1980-2011 (Mentzer et al)	1980	Bangladesh	NA	NA	NA	NA	NA	STh	ST	NA	CS5+CS6	eatA,etpBC	B1	5	L5	3	8
E2108	NA	NA	Bangladesh 1980-2011 (Mentzer et al)	2007	Bangladesh	NA	NA	NA	NA	NA	LT+STp	LT+ST	NA	CS6+CS12+CS21	Negative	B1	58	L8	4	13
E2110	NA	NA	Bangladesh 1980-2011 (Mentzer et al)	2007	Bangladesh	NA	NA	NA	NA	NA	LT+STp	LT+ST	NA	CS6+CS21	eatA,etpBC	B1	58	L8	4	13
E2118	NA	NA	Bangladesh 1980-2011 (Mentzer et al)	2007	Bangladesh	NA	NA	NA	NA	NA	LT	LT	NA	CS17	eatA,etpBC	B1	-	L5	3	8
E2121	NA	NA	Bangladesh 1980-2011 (Mentzer et al)	2007	Bangladesh	NA	NA	NA	NA	NA	LT+STp	LT+ST	NA	CS6+CS21	Negative	B1	58	L8	4	13
E2131	NA	NA	Bangladesh 1980-2011 (Mentzer et al)	2007	Bangladesh	NA	NA	NA	NA	NA	ST	ST	NA	CS21	Negative	B1	-	L3	4	12
E28	NA	NA	Bangladesh 1980-2011 (Mentzer et al)	1980	Bangladesh	NA	NA	NA	NA	NA	ST	ST	NA	CFnegative	eatA,etpBC	A	2	L11+L15	2	4
E2980	NA	NA	Bangladesh 1980-2011 (Mentzer et al)	2010	Bangladesh	NA	NA	NA	NA	NA	LT	LT	NA	CS7	eatA	B1	-	L3	4	12
E2981sec	NA	NA	Bangladesh 1980-2011 (Mentzer et al)	2010	Bangladesh	NA	NA	NA	NA	NA	LT	LT	NA	CFnegative	Negative	A	679	NA	9	39
E36	NA	NA	Bangladesh 1980-2011 (Mentzer et al)	1980	Bangladesh	NA	NA	NA	NA	NA	LT+STh	LT+ST	NA	CFA/I+CS21	eatA,etpBC	B1	-	L3	4	12
E45	NA	NA	Bangladesh 1980-2011 (Mentzer et al)	1980	Bangladesh	NA	NA	NA	NA	NA	LT+STh	LT+ST	NA	CFA/I+CS21	eatA	B1	-	L3	4	12
E5080	NA	NA	Bangladesh 1980-2011 (Mentzer et al)	2011	Bangladesh	NA	NA	NA	NA	NA	LT+STh	LT+ST	NA	CS5+CS6	eatA,etpBC	B1	5	L5	3	8
E5081	NA	NA	Bangladesh 1980-2011 (Mentzer et al)	2011	Bangladesh	NA	NA	NA	NA	NA	LT+STh	LT+ST	NA	CS5+CS6	eatA,etpBC	B1	5	L5	3	8
E5082	NA	NA	Bangladesh 1980-2011 (Mentzer et al)	2011	Bangladesh	NA	NA	NA	NA	NA	LT+STh	LT+ST	NA	CS5+CS6	Negative	B1	5	L5	3	8
E5084	NA	NA	Bangladesh 1980-2011 (Mentzer et al)	2011	Bangladesh	NA	NA	NA	NA	NA	STp	ST	NA	CS6	eatA,etpBC	E	1010	L7	1	1
E5085	NA	NA	Bangladesh 1980-2011 (Mentzer et al)	2011	Bangladesh	NA	NA	NA	NA	NA	STp	ST	NA	CS6	eatA	B1	86	L18	4	16
E5086	NA	NA	Bangladesh 1980-2011 (Mentzer et al)	2011	Bangladesh	NA	NA	NA	NA	NA	STh	ST	NA	CFA/I+CS21	Negative	B1	-	L3	4	12
E5087	NA	NA	Bangladesh 1980-2011 (Mentzer et al)	2011	Bangladesh	NA	NA	NA	NA	NA	ST	ST	NA	CFnegative	eatA,etpBC	D	44	NA	9	39
E5088	NA	NA	Bangladesh 1980-2011 (Mentzer et al)	2011	Bangladesh	NA	NA	NA	NA	NA	STp	ST	NA	CS6	eatA,etpBC	B1	86	L18	4	16
E5089	NA	NA	Bangladesh 1980-2011 (Mentzer et al)	2011	Bangladesh	NA	NA	NA	NA	NA	LT+ST	LT+ST	NA	CFnegative	Negative	A	-	L11+L13	2	6
E54	NA	NA	Bangladesh 1980-2011 (Mentzer et al)	1980	Bangladesh	NA	NA	NA	NA	NA	LT+STh	LT+ST	NA	CS1+CS3+CS21	Negative	A	215	L1	6	26
E66	NA	NA	Bangladesh 1980-2011 (Mentzer et al)	1988	Bangladesh	NA	NA	NA	NA	NA	LT+STh	LT+ST	NA	CS2+CS3+CS21	etpBC	A	215	L2	6	27
E70	NA	NA	Bangladesh 1980-2011 (Mentzer et al)	1988	Bangladesh	NA	NA	NA	NA	NA	LT+STh	LT+ST	NA	CS2+CS3+CS21	eatA,etpBC	A	215	L2	6	27
E71	NA	NA	Bangladesh 1980-2011 (Mentzer et al)	1988	Bangladesh	NA	NA	NA	NA	NA	LT+STh	LT+ST	NA	CS2+CS3+CS21	eatA,etpBC	A	215	L2	6	27
E74	NA	NA	Bangladesh 1980-2011 (Mentzer et al)	1988	Bangladesh	NA	NA	NA	NA	NA	LT+ST	LT+ST	NA	CS21	eatA,etpBC	B1	-	L3	4	12
E79	NA	NA	Bangladesh 1980-2011 (Mentzer et al)	1986	Bangladesh	NA	NA	NA	NA	NA	LT+STh	LT+ST	NA	CS3+CS21	Negative	B1	-	L16	5	23
E8	NA	NA	Bangladesh 1980-2011 (Mentzer et al)	1980	Bangladesh	NA	NA	NA	NA	NA	LT+STh	LT+ST	NA	CS1+CS3+CS21	eatA	A	215	L1	6	26
E85	NA	NA	Bangladesh 1980-2011 (Mentzer et al)	1987	Bangladesh	NA	NA	NA	NA	NA	LT	LT	NA	CS3+CS21	eatA,etpBC	B1	7	L16	5	23
E87	NA	NA	Bangladesh 1980-2011 (Mentzer et al)	1988	Bangladesh	NA	NA	NA	NA	NA	LT+STh	LT+ST	NA	CS2+CS3+CS21	eatA,etpBC	A	215	L2	6	27
E88	NA	NA	Bangladesh 1980-2011 (Mentzer et al)	1988	Bangladesh	NA	NA	NA	NA	NA	LT+STh	LT+ST	NA	CS5+CS6	eatA	B1	5	L5	3	8
E97	NA	NA	Bangladesh 1980-2011 (Mentzer et al)	1988	Bangladesh	NA	NA	NA	NA	NA	LT+STp	LT+ST	NA	CS6	eatA	A	-	NA	2	5
E99	NA	NA	Bangladesh 1980-2011 (Mentzer et al)	1988	Bangladesh	NA	NA	NA	NA	NA	LT+STh	LT+ST	NA	CS2+CS21	eatA,etpBC	A	215	L2	6	27
E1003	NA	NA	Global 1983-2008 (Mentzer et al)	1999	Egypt	NA	NA	NA	NA	NA	LT+STh	LT+ST	NA	CS5+CS6	eatA	B1	5	L5	3	8
E1009	NA	NA	Global 1983-2008 (Mentzer et al)	1999	Egypt	NA	NA	NA	NA	NA	LT	LT	NA	CS7	eatA,etpBC	B1	-	L3	4	12
E1018	NA	NA	Global 1983-2008 (Mentzer et al)	1999	Egypt	NA	NA	NA	NA	NA	LT+STh	LT+ST	NA	CS2+CS3+CS21	eatA,etpBC	A	215	L2	6	27
E1034	NA	NA	Global 1983-2008 (Mentzer et al)	1999	Egypt	NA	NA	NA	NA	NA	LT+STh	LT+ST	NA	CS5+CS6	eatA	B1	5	L5	3	8
E1057	NA	NA	Global 1983-2008 (Mentzer et al)	2000	Egypt	NA	NA	NA	NA	NA	LT+STp	LT+ST	NA	CS27A	Negative	A	-	L11+L15	2	4
E106	NA	NA	Global 1983-2008 (Mentzer et al)	1985	unknown	NA	NA	NA	NA	NA	LT+STp	LT+ST	NA	CS6	eatA	A	-	NA	2	5
E1072	NA	NA	Global 1983-2008 (Mentzer et al)	2000	Egypt	NA	NA	NA	NA	NA	LT+STp	LT+ST	NA	CS20	eatA	F	675	NA	7	33
E1074	NA	NA	Global 1983-2008 (Mentzer et al)	2000	Egypt	NA	NA	NA	NA	NA	LT+STh	LT+ST	NA	CS1+CS3+CS21	eatA	A	215	L1	6	26
E1085	NA	NA	Global 1983-2008 (Mentzer et al)	2000	Egypt	NA	NA	NA	NA	NA	LT+STh	LT+ST	NA	CS5+CS6	Negative	B1	5	L5	3	8
E1091	NA	NA	Global 1983-2008 (Mentzer et al)	2000	Egypt	NA	NA	NA	NA	NA	STh	ST	NA	CFA/I+CS21	eatA	A	2	L11+L15	2	4
E1101	NA	NA	Global 1983-2008 (Mentzer et al)	2000	Egypt	NA	NA	NA	NA	NA	LT+STp	LT+ST	NA	CS30	eatA	A	2	L11+L15	2	4
E1102	NA	NA	Global 1983-2008 (Mentzer et al)	2000	Egypt	NA	NA	NA	NA	NA	ST	ST	NA	CFnegative	Negative	B1	-	NA	5	24
E1111	NA	NA	Global 1983-2008 (Mentzer et al)	2000	Egypt	NA	NA	NA	NA	NA	LT+STh	LT+ST	NA	CS5+CS6	eatA,etpBC	B1	5	L5	3	8
E1114	NA	NA	Global 1983-2008 (Mentzer et al)	2001	Kenya	NA	NA	NA	NA	NA	LT	LT	NA	CS6+CS8	eatA	A	155	L4	6	28
E1115	NA	NA	Global 1983-2008 (Mentzer et al)	2001	Kenya	NA	NA	NA	NA	NA	LT	LT	NA	CS6+CS8	eatA	A	155	L4	6	28
E1116	NA	NA	Global 1983-2008 (Mentzer et al)	2001	Kenya	NA	NA	NA	NA	NA	LT	LT	NA	CS6+CS8	eatA	A	155	L4	6	28
E1117	NA	NA	Global 1983-2008 (Mentzer et al)	2001	Kenya	NA	NA	NA	NA	NA	LT	LT	NA	CS6+CS8	eatA	A	155	L4	6	28
E1118	NA	NA	Global 1983-2008 (Mentzer et al)	2001	Kenya	NA	NA	NA	NA	NA	LT	LT	NA	CS6+CS8	eatA	A	155	L4	6	28
E1119	NA	NA	Global 1983-2008 (Mentzer et al)	2001	Kenya	NA	NA	NA	NA	NA	LT	LT	NA	CS6+CS21	eatA	A	155	L4	6	28
E1120	NA	NA	Global 1983-2008 (Mentzer et al)	2001	Kenya	NA	NA	NA	NA	NA	LT	LT	NA	CS6+CS21	eatA	A	155	L4	6	28
E1121	NA	NA	Global 1983-2008 (Mentzer et al)	2001	Kenya	NA	NA	NA	NA	NA	LT	LT	NA	CS6+CS21	eatA	A	155	L4	6	28
E1122	NA	NA	Global 1983-2008 (Mentzer et al)	2001	Kenya	NA	NA	NA	NA	NA	LT	LT	NA	CS6+CS21	eatA	A	155	L4	6	28
E1167	NA	NA	Global 1983-2008 (Mentzer et al)	2001	Egypt	NA	NA	NA	NA	NA	LT+STp	LT+ST	NA	CS19	eatA,etpBC	A	-	L9+L10+L2	2	3
E1169	NA	NA	Global 1983-2008 (Mentzer et al)	2001	Egypt	NA	NA	NA	NA	NA	LT+STp	LT+ST	NA	CS20	Negative	F	675	NA	7	33
E1189	NA	NA	Global 1983-2008 (Mentzer et al)	2001	Egypt	NA	NA	NA	NA	NA	STh	ST	NA	CS6+CS21	Negative	B1	58	L8	4	13
E1193	NA	NA	Global 1983-2008 (Mentzer et al)	2001	Egypt	NA	NA	NA	NA	NA	ST	ST	NA	CFnegative	Negative	A	532	L12+L14	2	7
E1242	NA	NA	Global 1983-2008 (Mentzer et al)	2001	Egypt	NA	NA	NA	NA	NA	LT+STp	LT+ST	NA	CS18	eatA	B1	-	NA	4	18
E1245	NA	NA	Global 1983-2008 (Mentzer et al)	2001	Egypt	NA	NA	NA	NA	NA	STp	ST	NA	CS6	Negative	E	1010	L7	1	1
E1248	NA	NA	Global 1983-2008 (Mentzer et al)	2001	Egypt	NA	NA	NA	NA	NA	LT+STp	LT+ST	NA	CS19	eatA,etpBC	A	-	L9+L10+L2	2	3
E1258	NA	NA	Global 1983-2008 (Mentzer et al)	2001	Egypt	NA	NA	NA	NA	NA	LT+STp	LT+ST	NA	CS19	eatA,etpBC	A	-	L9+L10+L2	2	3
E126	NA	NA	Global 1983-2008 (Mentzer et al)	1989	unknown	NA	NA	NA	NA	NA	STh	ST	NA	CS14	etpBC	B1	87	L19	4	15
E1264	NA	NA	Global 1983-2008 (Mentzer et al)	2001	Egypt	NA	NA	NA	NA	NA	LT+STp	LT+ST	NA	CS19	eatA,etpBC	A	-	L9+L10+L2	2	3
E1281	NA	NA	Global 1983-2008 (Mentzer et al)	2001	Egypt	NA	NA	NA	NA	NA	LT+STp	LT+ST	NA	CS19	eatA,etpBC	A	-	L9+L10+L2	2	3
E1282	NA	NA	Global 1983-2008 (Mentzer et al)	2001	Egypt	NA	NA	NA	NA	NA	ST	ST	NA	CFnegative	Negative	B1	1101	L24	5	21
E1285	NA	NA	Global 1983-2008 (Mentzer et al)	2001	Egypt	NA	NA	NA	NA	NA	LT+STp	LT+ST	NA	CS19	eatA,etpBC	A	-	L9+L10+L2	2	3
E1287	NA	NA	Global 1983-2008 (Mentzer et al)	2001	Egypt	NA	NA	NA	NA	NA	STh	ST	NA	CFA/I+CS21	eatA,etpBC	A	2	L11+L15	2	4
E129	NA	NA	Global 1983-2008 (Mentzer et al)	1989	DR Congo (Zaire)	NA	NA	NA	NA	NA	LT	LT	NA	CS17	eatA,etpBC	B1	-	L17	4	11
E1298	NA	NA	Global 1983-2008 (Mentzer et al)	2001	Egypt	NA	NA	NA	NA	NA	LT+STh	LT+ST	NA	CS5+CS6	eatA	B1	5	L5	3	8
E1316	NA	NA	Global 1983-2008 (Mentzer et al)	1996	Nepal	NA	NA	NA	NA	NA	LT+STh	LT+ST	NA	CS1+CS3+CS21	eatA,etpBC	A	215	L1	6	26
E1328	NA	NA	Global 1983-2008 (Mentzer et al)	1996	Kenya	NA	NA	NA	NA	NA	STp	ST	NA	CS6	Negative	E	1010	L7	1	1
E1329	NA	NA	Global 1983-2008 (Mentzer et al)	1996	Kenya	NA	NA	NA	NA	NA	STp	ST	NA	CS6	Negative	E	1010	L7	1	1
E133	NA	NA	Global 1983-2008 (Mentzer et al)	1989	unknown	NA	NA	NA	NA	NA	LT+STp	LT+ST	NA	CS12	Negative	A	-	L11+L15	2	4
E1334	NA	NA	Global 1983-2008 (Mentzer et al)	1996	China	NA	NA	NA	NA	NA	STp	ST	NA	CS6	Negative	E	-	L7	1	1
E135	NA	NA	Global 1983-2008 (Mentzer et al)	1989	unknown	NA	NA	NA	NA	NA	LT	LT	NA	CS6+CS8	eatA	A	-	L4	6	28
E1352	NA	NA	Global 1983-2008 (Mentzer et al)	1996	Egypt	NA	NA	NA	NA	NA	STh	ST	NA	CS6+CS21	Negative	B1	58	L8	4	13
E1355	NA	NA	Global 1983-2008 (Mentzer et al)	1996	Egypt	NA	NA	NA	NA	NA	STh	ST	NA	CS6+CS21	Negative	B1	58	L8	4	13
E1356	NA	NA	Global 1983-2008 (Mentzer et al)	1996	Egypt	NA	NA	NA	NA	NA	STh	ST	NA	CS6+CS21	Negative	B1	58	L8	4	13
E1360sec	NA	NA	Global 1983-2008 (Mentzer et al)	1996	Tunisia	NA	NA	NA	NA	NA	ST	ST	NA	F41a	Negative	B1	21	L19	4	15
E1362	NA	NA	Global 1983-2008 (Mentzer et al)	1996	Egypt	NA	NA	NA	NA	NA	STh	ST	NA	CS21	etpBC	A	155	L4	6	28
E1363	NA	NA	Global 1983-2008 (Mentzer et al)	1996	Egypt	NA	NA	NA	NA	NA	STh	ST	NA	CS6+CS21	etpBC	A	155	L4	6	28
E1365	NA	NA	Global 1983-2008 (Mentzer et al)	1996	Egypt	NA	NA	NA	NA	NA	STh	ST	NA	CS21	etpBC	A	155	L4	6	28
E1373	NA	NA	Global 1983-2008 (Mentzer et al)	1996	Indonesia	NA	NA	NA	NA	NA	STp	ST	NA	CS6	Negative	E	1010	L7	1	1
E1392	NA	NA	Global 1983-2008 (Mentzer et al)	1996	Indonesia	NA	NA	NA	NA	NA	STp	ST	NA	CS6	Negative	B1	357	L17	4	11
E1398	NA	NA	Global 1983-2008 (Mentzer et al)	1996	Indonesia	NA	NA	NA	NA	NA	STp	ST	NA	CS6	Negative	B1	357	L17	4	11
E1407	NA	NA	Global 1983-2008 (Mentzer et al)	1996	Mexico	NA	NA	NA	NA	NA	LT	LT	NA	CS6	etpBC	B1	-	L20	4	14
E141	NA	NA	Global 1983-2008 (Mentzer et al)	1989	Mayanmar (Burma)	NA	NA	NA	NA	NA	LT	LT	NA	CS7	Negative	B1	-	L3	4	12
E1429tiny	NA	NA	Global 1983-2008 (Mentzer et al)	1997	Venezuela	NA	NA	NA	NA	NA	LT	LT	NA	CS6+CS21	eatA	A	155	L4	6	28
E143	NA	NA	Global 1983-2008 (Mentzer et al)	1983	Japan	NA	NA	NA	NA	NA	LT+STh	LT+ST	NA	CS2+CS3+CS21	Negative	A	215	L2	6	27
E1432G	NA	NA	Global 1983-2008 (Mentzer et al)	1997	Venezuela	NA	NA	NA	NA	NA	LT	LT	NA	CFnegative	Negative	A	-	L11+L15	2	4
E1432w	NA	NA	Global 1983-2008 (Mentzer et al)	1997	Venezuela	NA	NA	NA	NA	NA	LT	LT	NA	CFnegative	Negative	D	77	L28	8	36
E1433	NA	NA	Global 1983-2008 (Mentzer et al)	1997	Morocco	NA	NA	NA	NA	NA	LT+STh	LT+ST	NA	CS1+CS3+CS21	eatA,etpBC	A	215	L1	6	26
E1441	NA	NA	Global 1983-2008 (Mentzer et al)	1997	Kenya	NA	NA	NA	NA	NA	LT	LT	NA	CS6+CS21	eatA	A	155	L4	6	28
E1442	NA	NA	Global 1983-2008 (Mentzer et al)	1997	Kenya	NA	NA	NA	NA	NA	LT	LT	NA	CS6+CS21	eatA	A	155	L4	6	28
E1460	NA	NA	Global 1983-2008 (Mentzer et al)	1997	Indonesia	NA	NA	NA	NA	NA	ST	ST	NA	CFnegative	Negative	G	641	NA	9	39
E1484	NA	NA	Global 1983-2008 (Mentzer et al)	2003	Guinea Bissau	NA	NA	NA	NA	NA	STp	ST	NA	CS6	Negative	E	1010	L7	1	1
E151	NA	NA	Global 1983-2008 (Mentzer et al)	1983	Japan	NA	NA	NA	NA	NA	LT	LT	NA	CS12	Negative	F	675	NA	7	33
E1512	NA	NA	Global 1983-2008 (Mentzer et al)	2003	Guinea Bissau	NA	NA	NA	NA	NA	STp	ST	NA	CS6	Negative	B1	357	L17	4	11
E1516	NA	NA	Global 1983-2008 (Mentzer et al)	2003	Guinea Bissau	NA	NA	NA	NA	NA	LT	LT	NA	CS6	eatA	A	155	L4	6	28
E1523	NA	NA	Global 1983-2008 (Mentzer et al)	2003	Guinea Bissau	NA	NA	NA	NA	NA	STp	ST	NA	CS6	Negative	B1	357	L17	4	11
E1524	NA	NA	Global 1983-2008 (Mentzer et al)	1989	Argentina	NA	NA	NA	NA	NA	STh	ST	NA	CS5+CS6+CS14	eatA,etpBC	B1	-	L16	5	23
E1525	NA	NA	Global 1983-2008 (Mentzer et al)	1989	Argentina	NA	NA	NA	NA	NA	LT+STh	LT+ST	NA	CS27B	Negative	A	-	L11+L13	2	6
E1526	NA	NA	Global 1983-2008 (Mentzer et al)	1989	Argentina	NA	NA	NA	NA	NA	ST	ST	NA	CFnegative	Negative	B1	7	L16	5	23

E1527	NA	NA	Global 1983-2008 (Mentzer et al)	1989	Argentina	NA	NA	NA	NA	NA	STh	ST	NA	CS5+CS6	eatA	B1	-	L17	4	11
E1532	NA	NA	Global 1983-2008 (Mentzer et al)	1989	Argentina	NA	NA	NA	NA	NA	LT	LT	NA	CFnegative	Negative	A	-	L11+L15	2	4
E1533	NA	NA	Global 1983-2008 (Mentzer et al)	1989	Argentina	NA	NA	NA	NA	NA	LT+STp	LT+ST	NA	CS30	eatA	B1	-	NA	4	18
E1534	NA	NA	Global 1983-2008 (Mentzer et al)	1989	Argentina	NA	NA	NA	NA	NA	ST	ST	NA	CFnegative	Negative	B1	469	L23	4	17
E1535	NA	NA	Global 1983-2008 (Mentzer et al)	1989	Argentina	NA	NA	NA	NA	NA	LT+STh	LT+ST	NA	CS1+CS3+CS21	eatA,etpBC	A	215	L1	6	26
E1541	NA	NA	Global 1983-2008 (Mentzer et al)	1989	Argentina	NA	NA	NA	NA	NA	STh	ST	NA	CFA/I+CS21	eatA,etpBC	B1	-	L3	4	12
E1542	NA	NA	Global 1983-2008 (Mentzer et al)	1989	Argentina	NA	NA	NA	NA	NA	LT	LT	NA	CFnegative	Negative	A	718	L11+L15	2	4
E1543	NA	NA	Global 1983-2008 (Mentzer et al)	1989	Argentina	NA	NA	NA	NA	NA	STh	ST	NA	CFnegative	etpBC	A	-	NA	5	25
E1544	NA	NA	Global 1983-2008 (Mentzer et al)	1989	Argentina	NA	NA	NA	NA	NA	STh	ST	NA	CFnegative	etpBC	A	-	NA	5	25
E1548	NA	NA	Global 1983-2008 (Mentzer et al)	1989	Argentina	NA	NA	NA	NA	NA	STh	ST	NA	CFA/I+CS21	etpBC	A	-	L6	5	22
E1556	NA	NA	Global 1983-2008 (Mentzer et al)	1989	Argentina	NA	NA	NA	NA	NA	LT	LT	NA	CS28B	Negative	A	987	L12+L14	2	7
E1561	NA	NA	Global 1983-2008 (Mentzer et al)	1989	Argentina	NA	NA	NA	NA	NA	LT	LT	NA	CS28B	Negative	A	987	L12+L14	2	7
E1564	NA	NA	Global 1983-2008 (Mentzer et al)	1989	Argentina	NA	NA	NA	NA	NA	LT	LT	NA	CFnegative	Negative	A	679	L9+L10+L2	2	3
E157	NA	NA	Global 1983-2008 (Mentzer et al)	1983	Japan	NA	NA	NA	NA	NA	LT	LT	NA	CS6	Negative	B1	537	NA	3	9
E1573	NA	NA	Global 1983-2008 (Mentzer et al)	1989	Argentina	NA	NA	NA	NA	NA	LT	LT	NA	CFnegative	Negative	A	835	L11+L15	2	4
E1574	NA	NA	Global 1983-2008 (Mentzer et al)	1989	Argentina	NA	NA	NA	NA	NA	LT	LT	NA	CS27B	Negative	A	-	L11+L13	2	6
E1576	NA	NA	Global 1983-2008 (Mentzer et al)	1989	Argentina	NA	NA	NA	NA	NA	LT	LT	NA	CS13	Negative	B1	866	L20	4	14
E1580	NA	NA	Global 1983-2008 (Mentzer et al)	1989	Argentina	NA	NA	NA	NA	NA	LT+STp	LT+ST	NA	CS27A	Negative	A	-	L11+L13	2	6
E1581	NA	NA	Global 1983-2008 (Mentzer et al)	1989	Argentina	NA	NA	NA	NA	NA	LT	LT	NA	CS13+CS26	Negative	A	-	L11+L13	2	6
E1582	NA	NA	Global 1983-2008 (Mentzer et al)	1989	Argentina	NA	NA	NA	NA	NA	LT	LT	NA	CS27B	Negative	A	-	L11+L13	2	6
E1585	NA	NA	Global 1983-2008 (Mentzer et al)	1989	Argentina	NA	NA	NA	NA	NA	LT	LT	NA	CFnegative	Negative	A	865	L11+L13	2	6
E1586	NA	NA	Global 1983-2008 (Mentzer et al)	1989	Argentina	NA	NA	NA	NA	NA	LT+STp	LT+ST	NA	CS30	eatA	B1	1001	NA	4	18
E1587	NA	NA	Global 1983-2008 (Mentzer et al)	1989	Argentina	NA	NA	NA	NA	NA	STh	ST	NA	CFA/I+CS21	eatA,etpBC	B1	-	L3	4	12
E159	NA	NA	Global 1983-2008 (Mentzer et al)	1983	Japan	NA	NA	NA	NA	NA	LT	LT	NA	CFnegative	Negative	A	342	L11+L13	2	6
E1592	NA	NA	Global 1983-2008 (Mentzer et al)	1989	Argentina	NA	NA	NA	NA	NA	LT	LT	NA	CS7+CS28B	eatA,etpBC	B1	-	L3	4	12
E1593	NA	NA	Global 1983-2008 (Mentzer et al)	1989	Argentina	NA	NA	NA	NA	NA	STh	ST	NA	CS5+CS6	eatA	B1	-	L17	4	11
E1594	NA	NA	Global 1983-2008 (Mentzer et al)	1989	Argentina	NA	NA	NA	NA	NA	ST	ST	NA	CFnegative	eatA	A	-	L11+L15	2	4
E1596	NA	NA	Global 1983-2008 (Mentzer et al)	1989	Argentina	NA	NA	NA	NA	NA	LT	LT	NA	CS28B	eatA	A	987	L12+L14	2	7
E1597	NA	NA	Global 1983-2008 (Mentzer et al)	1989	Argentina	NA	NA	NA	NA	NA	LT+STp	LT+ST	NA	CS20	Negative	D	-	L29	9	41
E1599	NA	NA	Global 1983-2008 (Mentzer et al)	1989	Argentina	NA	NA	NA	NA	NA	ST	ST	NA	CFnegative	Negative	B1	469	L23	4	17
E160	NA	NA	Global 1983-2008 (Mentzer et al)	1983	Japan	NA	NA	NA	NA	NA	LT	LT	NA	CFnegative	Negative	A	-	NA	6	30
E1600	NA	NA	Global 1983-2008 (Mentzer et al)	1989	Argentina	NA	NA	NA	NA	NA	LT	LT	NA	CS13	Negative	A	-	L11+L13	2	6
E1604	NA	NA	Global 1983-2008 (Mentzer et al)	1989	Argentina	NA	NA	NA	NA	NA	STh	ST	NA	CFA/I+CS21	eatA,etpBC	A	-	L6	5	22
E1607	NA	NA	Global 1983-2008 (Mentzer et al)	1989	Argentina	NA	NA	NA	NA	NA	LT	LT	NA	CS14	etpBC	B1	7	L16	5	23
E1609	NA	NA	Global 1983-2008 (Mentzer et al)	1989	Argentina	NA	NA	NA	NA	NA	LT	LT	NA	CS12	eatA,etpBC	A	-	L9+L10+L2	2	3
E1611	NA	NA	Global 1983-2008 (Mentzer et al)	1989	Argentina	NA	NA	NA	NA	NA	LT	LT	NA	CS6	etpBC	B1	66	NA	5	24
E1615	NA	NA	Global 1983-2008 (Mentzer et al)	1989	Argentina	NA	NA	NA	NA	NA	LT	LT	NA	CS13	Negative	A	-	L11+L13	2	6
E1616	NA	NA	Global 1983-2008 (Mentzer et al)	1989	Argentina	NA	NA	NA	NA	NA	LT	LT	NA	CFnegative	Negative	B1	88	L20	4	14
E1617	NA	NA	Global 1983-2008 (Mentzer et al)	1989	Argentina	NA	NA	NA	NA	NA	LT+STp	LT+ST	NA	CS20	Negative	D	-	L29	9	41
E1620	NA	NA	Global 1983-2008 (Mentzer et al)	1989	Argentina	NA	NA	NA	NA	NA	LT+STh	LT+ST	NA	CS1+CS3+CS21	eatA,etpBC	A	215	L1	6	26
E1623	NA	NA	Global 1983-2008 (Mentzer et al)	1990	Indonesia	NA	NA	NA	NA	NA	ST	ST	NA	CFnegative	Negative	B1	7	L16	5	23
E1624	NA	NA	Global 1983-2008 (Mentzer et al)	1990	Indonesia	NA	NA	NA	NA	NA	LT+STh	LT+ST	NA	CS2+CS3+CS21	eatA,etpBC	A	215	L2	6	27
E1625	NA	NA	Global 1983-2008 (Mentzer et al)	1990	Indonesia	NA	NA	NA	NA	NA	LT	LT	NA	CS6	Negative	B1	-	NA	3	9
E1628	NA	NA	Global 1983-2008 (Mentzer et al)	1990	Indonesia	NA	NA	NA	NA	NA	LT	LT	NA	CS6+CS21	eatA	A	155	L4	6	28
E1634	NA	NA	Global 1983-2008 (Mentzer et al)	1990	Indonesia	NA	NA	NA	NA	NA	LT	LT	NA	CFnegative	Negative	A	-	L11+L13	2	6
E1635	NA	NA	Global 1983-2008 (Mentzer et al)	1997	Indonesia	NA	NA	NA	NA	NA	LT	LT	NA	CS17	Negative	B1	5	L5	3	8
E1637	NA	NA	Global 1983-2008 (Mentzer et al)	1990	Indonesia	NA	NA	NA	NA	NA	ST	ST	NA	CFnegative	Negative	B1	7	L16	5	23
E1638	NA	NA	Global 1983-2008 (Mentzer et al)	1990	Indonesia	NA	NA	NA	NA	NA	ST	ST	NA	CFnegative	Negative	B1	-	L20	4	14
E1640	NA	NA	Global 1983-2008 (Mentzer et al)	1990	Indonesia	NA	NA	NA	NA	NA	LT+ST	LT+ST	NA	CS28A	Negative	B1	86	L18	4	16
E1641	NA	NA	Global 1983-2008 (Mentzer et al)	1990	Indonesia	NA	NA	NA	NA	NA	LT	LT	NA	CS23	eatA	D	535	L29	9	41
E1642	NA	NA	Global 1983-2008 (Mentzer et al)	1990	Indonesia	NA	NA	NA	NA	NA	LT+STp	LT+ST	NA	CS20	Negative	F	-	NA	7	34
E1646	NA	NA	Global 1983-2008 (Mentzer et al)	1990	Indonesia	NA	NA	NA	NA	NA	STh	ST	NA	CS5+CS6	eatA	B1	5	L5	3	8
E1647	NA	NA	Global 1983-2008 (Mentzer et al)	1990	Indonesia	NA	NA	NA	NA	NA	LT	LT	NA	CS6	eatA	B1	-	L20	4	14
E1648	NA	NA	Global 1983-2008 (Mentzer et al)	1990	Indonesia	NA	NA	NA	NA	NA	LT+STh	LT+ST	NA	CS1+CS3+CS21	eatA,etpBC	A	215	L1	6	26
E1649	NA	NA	Global 1983-2008 (Mentzer et al)	1997	Indonesia	NA	NA	NA	NA	NA	LT+STh	LT+ST	NA	CS2+CS3+CS21	eatA,etpBC	A	215	L2	6	27
E1650	NA	NA	Global 1983-2008 (Mentzer et al)	1990	Indonesia	NA	NA	NA	NA	NA	LT	LT	NA	CFnegative	Negative	B1	-	L20	4	14
E1654	NA	NA	Global 1983-2008 (Mentzer et al)	1990	Indonesia	NA	NA	NA	NA	NA	LT+STp	LT+ST	NA	CS1+CS3+CS21	eatA,etpBC	A	215	L1	6	26
E1657	NA	NA	Global 1983-2008 (Mentzer et al)	1997	Indonesia	NA	NA	NA	NA	NA	LT+STp	LT+ST	NA	CS12	Negative	B1	956	L18	4	16
E1659	NA	NA	Global 1983-2008 (Mentzer et al)	1990	Indonesia	NA	NA	NA	NA	NA	LT	LT	NA	CFnegative	Negative	B1	-	L20	4	14
E1661	NA	NA	Global 1983-2008 (Mentzer et al)	1990	Indonesia	NA	NA	NA	NA	NA	LT+STp	LT+ST	NA	CS28B	Negative	D	77	L28	8	36
E1666	NA	NA	Global 1983-2008 (Mentzer et al)	1990	Indonesia	NA	NA	NA	NA	NA	STh	ST	NA	CFA/I+CS21	etpBC	B1	-	L3	4	12
E1667	NA	NA	Global 1983-2008 (Mentzer et al)	1997	Indonesia	NA	NA	NA	NA	NA	LT+STh	LT+ST	NA	CS2+CS3+CS21	eatA,etpBC	A	215	L2	6	27
E167	NA	NA	Global 1983-2008 (Mentzer et al)	1983	Japan	NA	NA	NA	NA	NA	LT	LT	NA	CFnegative	Negative	A	-	L11+L13	2	6
E1673	NA	NA	Global 1983-2008 (Mentzer et al)	1990	Indonesia	NA	NA	NA	NA	NA	ST	ST	NA	CFnegative	Negative	B1	21	L19	4	15
E1674	NA	NA	Global 1983-2008 (Mentzer et al)	1990	Indonesia	NA	NA	NA	NA	NA	ST	ST	NA	CFnegative	Negative	B1	7	L16	5	23
E1679sec	NA	NA	Global 1983-2008 (Mentzer et al)	1990	Indonesia	NA	NA	NA	NA	NA	LT+STp	LT+ST	NA	CS12	Negative	A	-	L11+L15	2	4
E1682	NA	NA	Global 1983-2008 (Mentzer et al)	1990	Indonesia	NA	NA	NA	NA	NA	LT	LT	NA	CS27B	Negative	B1	21	L19	4	15
E1684	NA	NA	Global 1983-2008 (Mentzer et al)	1990	Indonesia	NA	NA	NA	NA	NA	STp	ST	NA	CS6	Negative	B1	357	L17	4	11
E1690	NA	NA	Global 1983-2008 (Mentzer et al)	1990	Indonesia	NA	NA	NA	NA	NA	ST	ST	NA	CS14	eatA,etpB	A	486	L11+L13	2	6
E2185	NA	NA	Global 1983-2008 (Mentzer et al)	2007	Bolivia	NA	NA	NA	NA	NA	LT	LT	NA	CS17	eatA,etpBC	B1	69	NA	5	24
E220	NA	NA	Global 1983-2008 (Mentzer et al)	1987	Japan	NA	NA	NA	NA	NA	STp	ST	NA	CS6	Negative	A	-	L9+L10+L2	2	3
E2219	NA	NA	Global 1983-2008 (Mentzer et al)	2007	Bolivia	NA	NA	NA	NA	NA	LT+STh	LT+ST	NA	CS1+CS3+CS21	eatA,etpBC	A	-	L1	6	26
E222	NA	NA	Global 1983-2008 (Mentzer et al)	1987	Japan	NA	NA	NA	NA	NA	ST	ST	NA	CS21	Negative	B1	58	L8	4	13
E223	NA	NA	Global 1983-2008 (Mentzer et al)	1987	Japan	NA	NA	NA	NA	NA	ST	ST	NA	CFnegative	Negative	A	-	L9+L10+L2	2	3
E224	NA	NA	Global 1983-2008 (Mentzer et al)	1987	Japan	NA	NA	NA	NA	NA	STh	ST	NA	CS6+CS21	Negative	B1	-	L8	4	13
E2256	NA	NA	Global 1983-2008 (Mentzer et al)	2007	Thailand	NA	NA	NA	NA	NA	STp	ST	NA	CS6	Negative	E	1010	L7	1	1
E2339	NA	NA	Global 1983-2008 (Mentzer et al)	2007	Bolivia	NA	NA	NA	NA	NA	LT+STh	LT+ST	NA	CS5+CS6	eatA	B1	5	L5	3	8
E2347	NA	NA	Global 1983-2008 (Mentzer et al)	2007	Bolivia	NA	NA	NA	NA	NA	LT	LT	NA	CFnegative	etpBC	B1	-	L3	4	12
E2348	NA	NA	Global 1983-2008 (Mentzer et al)	2007	Bolivia	NA	NA	NA	NA	NA	LT+ST	LT+ST	NA	CFnegative	Negative	B1	-	L16	5	23
E2362sec	NA	NA	Global 1983-2008 (Mentzer et al)	2008	Bolivia	NA	NA	NA	NA	NA	ST	ST	NA	CS27A	Negative	NA	-	NA	9	42
E2367	NA	NA	Global 1983-2008 (Mentzer et al)	2008	Bolivia	NA	NA	NA	NA	NA	LT	LT	NA	CFnegative	Negative	A	398	L9+L10+L2	2	3
E237	NA	NA	Global 1983-2008 (Mentzer et al)	1987	Japan	NA	NA	NA	NA	NA	STp	ST	NA	CS6	Negative	A	-	L9+L10+L2	2	3
E2370sec	NA	NA	Global 1983-2008 (Mentzer et al)	2008	Japan	NA	NA	NA	NA	NA	LT+ST	LT+ST	NA	CFnegative	Negative	A	342	L11+L13	2	6
E2371	NA	NA	Global 1983-2008 (Mentzer et al)	2008	Bolivia	NA	NA	NA	NA	NA	LT	LT	NA	CFnegative	Negative	A	342	L11+L13	2	6
E2377	NA	NA	Global 1983-2008 (Mentzer et al)	2007	Bolivia	NA	NA	NA	NA	NA	LT	LT	NA	CS6	Negative	A	-	L11+L15	2	4
E2386	NA	NA	Global 1983-2008 (Mentzer et al)	2007	Bolivia	NA	NA	NA	NA	NA	LT+STh	LT+ST	NA	CS1+CS3+CS21	eatA,etpBC	A	-	L1	6	26
E2388	NA	NA	Global 1983-2008 (Mentzer et al)	2007	Bolivia	NA	NA	NA	NA	NA	LT	LT	NA	CS27A	Negative	NA	-	NA	9	42
E239	NA	NA	Global 1983-2008 (Mentzer et al)	1987	Japan	NA	NA	NA	NA	NA	LT+STp	LT+ST	NA	CS20	Negative	D	77	L28	8	36
E2392	NA	NA	Global 1983-2008 (Mentzer et al)	2007	Bolivia	NA	NA	NA	NA	NA	LT	LT	NA	CFnegative	Negative	A	165	L11+L15	2	4
E2393	NA	NA	Global 1983-2008 (Mentzer et al)	2007	Bolivia	NA	NA	NA	NA	NA	LT	LT	NA	PCFO71	eatA,etpBC	B1	7	L16	5	23
E2395	NA	NA	Global 1983-2008 (Mentzer et al)	2007	Bolivia	NA	NA	NA	NA	NA	STh	ST	NA	CS14	eatA,etpBC	B1	-	L20	4	14
E2397	NA	NA	Global 1983-2008 (Mentzer et al)	2007	Bolivia	NA	NA	NA	NA	NA	LT+STh	LT+ST	NA	CS5+CS6	eatA	B1	5	L5	3	8
E2404	NA	NA	Global 1983-2008 (Mentzer et al)	2008	Bolivia	NA	NA	NA	NA	NA	LT	LT	NA	CFnegative	Negative	B1	-	L17	4	11
E2405	NA	NA	Global 1983-2008 (Mentzer et al)	2008	Bolivia	NA	NA	NA	NA	NA	LT	LT	NA	CFnegative	Negative	B1	-	L17	4	11
E2439	NA	NA	Global 1983-2008 (Mentzer et al)	2008	Bolivia	NA	NA	NA	NA	NA	ST	ST	NA	CFnegative	Negative	B2	43	NA	9	40
E251	NA	NA	Global 1983-2008 (Mentzer et al)	1987	Japan	NA	NA	NA	NA	NA	STh	ST	NA	CFnegative	Negative	A	-	L9+L10+L2	2	3
E263	NA	NA	Global 1983-2008 (Mentzer et al)	1987	Japan	NA	NA	NA	NA	NA	LT+ST	LT+ST	NA	CS1+CS21	eatA	A	-	L1	6	26
E272	NA	NA	Global 1983-2008 (Mentzer et al)	1987	Japan	NA	NA	NA	NA	NA	LT+STh	LT+ST	NA	CS2+CS3	eatA,etpBC	A	215	L2	6	27
E3015	NA	NA	Global 1983-2008 (Mentzer et al)	1998	Egypt	NA	NA	NA	NA	NA	STp	ST	NA	CS6	Negative	B1	357	L17	4	11
E329	NA	NA	Global 1983-2008 (Mentzer et al)	1998	Mexico	NA	NA	NA	NA	NA	LT	LT	NA	CFnegative	eatA,etpBC	A	478	L11+L15	2	4
E330	NA	NA	Global 1983-2008 (Mentzer et al)	1998	Mexico	NA	NA	NA	NA	NA	LT	LT	NA	CS28B	Negative	A	987	L12+L14	2	7
E333	NA	NA	Global 1983-2008 (Mentzer et al)	1998	Mexico	NA	NA	NA	NA	NA	STp	ST	NA	CS6	eatA	B1	357	L17	4	11
E335	NA	NA	Global 1983-2008 (Mentzer et al)	1998	Mexico	NA	NA	NA	NA	NA	LT+STp	LT+ST	NA	CS6+CS21	Negative	B1	-	L8	4	13
E336	NA	NA	Global 1983-2008 (Mentzer et al)	1998	Mexico	NA	NA	NA	NA	NA	LT+STp	LT+ST	NA	CS6+CS21	Negative	B1	-	L8	4	13
E340	NA	NA	Global 1983-2008 (Mentzer et al)	1998	Mexico	NA	NA	NA	NA	NA	STh	ST	NA	CS21	etpBC	A	155	L4	6	28
E343	NA	NA	Global 1983-2008 (Mentzer et al)	1998	Mexico	NA	NA	NA	NA	NA	STh	ST	NA	CS21	etpBC	A	155	L4	6	28
E344	NA	NA	Global 1983-2008 (Mentzer et al)	1998	Mexico	NA	NA	NA	NA	NA	STp	ST	NA	CS6	Negative	E	1010	L7	1	1
E351	NA	NA	Global 1983-2008 (Mentzer et al)	1998	Mexico	NA	NA	NA	NA	NA	LT	LT	NA	CFnegative	Negative	B1	7	L16	5	23
E354	NA	NA	Global 1983-2008 (Mentzer et al)	1998	Mexico	NA	NA	NA	NA	NA	ST	ST	NA	CFnegative	Negative	A	2	L11+L15	2	4
E356	NA	NA	Global 1983-2008 (Mentzer et al)	1998	Mexico	NA	NA	NA	NA	NA	ST	ST	NA	PCFO71+CS21	Negative	A	2	L11+L15	2	4
E360	NA	NA	Global 1983-2008 (Mentzer et al)	1998	Mexico	NA	NA	NA	NA	NA	LT	LT	NA	PCFO71+CS21	Negative	B1	7	L16	5	23
E361	NA	NA	Global 1983-2008 (Mentzer et al)	1998	Mexico	NA	NA	NA	NA	NA	LT+STp	LT+ST	NA	CS13+CS30	Negative	B1	866	L20	4	14
E370	NA	NA	Global 1983-2008 (Mentzer et al)	1998	Guatemala	NA	NA	NA	NA	NA	STp	ST	NA	CS6	Negative	E	1010	L7	1	1
E390	NA	NA	Global 1983-2008 (Mentzer et al)	1999	Guatemala	NA	NA	NA	NA	NA	ST	ST	NA	CFnegative	Negative	B1	-	L24	5	21
E391	NA	NA	Global 1983-2008 (Mentzer et al)	1999	Guatemala	NA	NA	NA	NA	NA	LT	LT	NA	CFnegative	Negative	B1	86	L18	4	16
E399	NA	NA	Global 1983-2008 (Mentzer et al)	1999	Guatemala	NA	NA	NA	NA	NA	LT	LT	NA	CFnegative	Negative	A	-	L11+L13	2	6
E405	NA	NA	Global 1983-2008 (Mentzer et al)	1999	Guatemala	NA	NA	NA	NA	NA	LT	LT	NA	CS28B	Negative	A	987	L12+L14	2	7
E4092	NA	NA	Global 1983-2008 (Mentzer et al)	2005	Israel	NA	NA	NA	NA	NA	LT	LT	NA	CS6+CS8	eatA	A	155	L4	6	28
E415	NA	NA	Global 1983-2008 (Mentzer et al)	1999	Guatemala	NA	NA	NA	NA	NA	LT	LT	NA	CS6	Negative	A	-	L11+L15	2	4
E416	NA	NA	Global 1983-2008 (Mentzer et al)	1999	Guatemala	NA	NA	NA	NA	NA	STp	ST	NA	CS6	eatA	B1	357	L17	4	11
E425	NA	NA	Global 1983-2008 (Mentzer et al)	1999	Guatemala	NA	NA	NA	NA	NA	LT	LT	NA	CS6	Negative	A	-	L11+L15	2	4
E445	NA	NA	Global 1983-2008 (Mentzer et al)	2000	Guatemala	NA	NA	NA	NA	NA	LT	LT	NA	PCFO71	Negative	B1	7	L16	5	23
E451	NA	NA	Global 1983-2008 (Mentzer et al)	2000	Guatemala	NA	NA	NA	NA	NA	LT	LT	NA	CS27B	Negative	A	-	L11+L15	2	4
E471	NA	NA	Global 1983-2008 (Mentzer et al)	2000	Guatemala	NA	NA	NA	NA	NA	LT+STh	LT+ST	NA	CS1+CS3+CS21	eatA,etpBC	A	-	L1	6	26
E5049	NA	NA	Global 1983-2008 (Mentzer et al)	unkown	India	NA	NA	NA	NA	NA	LT	LT	NA	CS6+CS13	etpBC	B1	-	L23	4	17
E5051	NA	NA	Global 1983-2008 (Mentzer et al)	unkown	India	NA	NA	NA	NA	NA	LT	LT	NA	CS6	eatA,etpBC	B1	469	L23	4	17
E5052	NA	NA	Global 1983-2008 (Mentzer et al)	unkown	India	NA	NA	NA	NA	NA	LT	LT	NA	CS6	etpBC	A	-	L11+L13	2	6
E509	NA	NA	Global 1983-2008 (Mentzer et al)	2000	Mexico	NA	NA	NA	NA	NA	ST	ST	NA	CFnegative	Negative	B1	469	L23	4	17
E513	NA	NA	Global 1983-2008 (Mentzer et al)	2000	Mexico	NA	NA	NA	NA	NA	ST	ST	NA	CFnegative	Negative	A	-	L11+L15	2	4
E517	NA	NA	Global 1983-2008 (Mentzer et al)	2000	Mexico	NA	NA	NA	NA	NA	LT+STh	LT+ST	NA	CS1+CS3+CS21	eatA,etpBC	A	215	L1	6	26
E519	NA	NA	Global 1983-2008 (Mentzer et al)	2000	Mexico	NA	NA	NA	NA	NA	LT	LT	NA	CFnegative	Negative	B1	-	L18	4	16
E520	NA	NA	Global 1983-2008 (Mentzer et al)	2000	Mexico	NA	NA	NA	NA	NA	STp	ST	NA	CS6	Negative	E	1010	L7	1	1
E523	NA	NA	Global 1983-2008 (Mentzer et al)	2000	Mexico	NA	NA	NA	NA	NA	ST	ST	NA	CFnegative	Negative	B2	-	NA	9	40
E527	NA	NA	Global 1983-2008 (Mentzer et al)	2000	Mexico	NA	NA	NA	NA	NA	LT	LT	NA	CFnegative	eatA	B1	-	L18	4	16
E528	NA	NA	Global 1983-2008 (Mentzer et al)	2000	Mexico	NA	NA	NA	NA	NA	LT	LT	NA	CS23+CS26	Negative	B1	-	L18	4	16
E529	NA	NA	Global 1983-2008 (Mentzer et al)	2000	Mexico	NA	NA	NA	NA	NA	LT	LT	NA	CS13	Negative	A	398	L9+L10+L2	2	3
E554	NA	NA	Global 1983-2008 (Mentzer et al)	2000	Mexico	NA	NA	NA	NA	NA	STp	ST	NA	CS6	eatA,etpBC	E	1010	L7	1	1
E562	NA	NA	Global 1983-2008 (Mentzer et al)	2000	Mexico	NA	NA	NA	NA	NA	STh	ST	NA	CFA/I+CS21	eatA,etpBC	A	-	L6	5	22
E563	NA	NA	Global 1983-2008 (Mentzer et al)	2000	Mexico	NA	NA	NA	NA	NA	STh	ST	NA	CFA/I+CS21	eatA,etpBC	A	-	L6	5	22
E604	NA	NA	Global 1983-2008 (Mentzer et al)	2000	Guatemala	NA	NA	NA	NA	NA	LT+STp	LT+ST	NA	CS27A	eatA,etpBC	A	-	L11+L13	2	6
E616	NA	NA	Global 1983-2008 (Mentzer et al)	2000	Guatemala	NA	NA	NA	NA	NA	LT+STp	LT+ST	NA	CS27A	Negative	A	-	L11+L13	2	6
E618	NA	NA	Global 1983-2008 (Mentzer et al)	2000	Guatemala	NA	NA	NA	NA	NA	ST	ST	NA	CFnegative	Negative	B1	469	L23	4	17
E620	NA	NA	Global 1983-2008 (Mentzer et al)	2000	Guatemala	NA	NA	NA	NA	NA	ST	ST	NA	CFnegative	Negative	A	532	L12+L14	2	7
E621	NA	NA	Global 1983-2008 (Mentzer et al)	2000	Guatemala	NA	NA	NA	NA	NA	ST	ST	NA	CFnegative	Negative	B1	-	L20	4	14
E622	NA	NA	Global 1983-2008 (Mentzer et al)	2000	Guatemala	NA	NA	NA	NA	NA	LT	LT	NA	CFnegative	Negative	B1	87	L19	4	15
E626	NA	NA	Global 1983-2008 (Mentzer et al)	2000	Guatemala	NA	NA	NA	NA	NA	ST	ST	NA	CFnegative	eatA,etpBC	A	-	L12+L14	2	7
E628	NA	NA	Global 1983-2008 (Mentzer et al)	2000	Guatemala	NA	NA	NA	NA	NA	LT+STp	LT+ST	NA	CS27A	Negative	A	-	L11+L13	2	6
E632	NA	NA	Global 1983-2008 (Mentzer et al)	2000	Guatemala	NA	NA	NA	NA	NA	LT+STh	LT+ST	NA	CS1+CS3+CS21	eatA,etpBC	A	-	L1	6	26
E636	NA	NA	Global 1983-2008 (Mentzer et al)	2000	Guatemala	NA	NA	NA	NA	NA	STh	ST	NA	CFA/I+CS21	eatA	A	-	L6	5	22
E645	NA	NA	Global 1983-2008 (Mentzer et al)	2000	Guatemala	NA	NA	NA	NA	NA	LT	LT	NA	CS27B	Negative	B1	21	L19	4	15
E655	NA	NA	Global 1983-2008 (Mentzer et al)	2000	Guatemala	NA	NA	NA	NA	NA	STh	ST	NA	CS5+CS6	etpBC	B1	5	L5	3	8
E658	NA	NA	Global 1983-2008 (Mentzer et al)	2001	Guatemala	NA	NA	NA	NA	NA	LT	LT	NA	CFA/I+CS21	eatA,etpBC	B1	-	L3	4	12
E659	NA	NA	Global 1983-2008 (Mentzer et al)	2001	Guatemala	NA	NA	NA	NA	NA	LT	LT	NA	CFnegative	Negative	B1	86	L18	4	16
E662	NA	NA	Global 1983-2008 (Mentzer et al)	2001	Guatemala	NA	NA	NA	NA	NA	LT	LT	NA	CFnegative	Negative	A	-	L12+L14	2	7
E703	NA	NA	Global 1983-2008 (Mentzer et al)	2001	Guatemala	NA	NA	NA	NA	NA	ST	ST	NA	CFnegative	Negative	B1	469	L23	4	17
E704	NA	NA	Global 1983-2008 (Mentzer et al)	2001	Guatemala	NA	NA	NA	NA	NA	ST	ST	NA	CFnegative	Negative	A	-	L12+L14	2	7
E705	NA	NA	Global 1983-2008 (Mentzer et al)	2001	Guatemala	NA	NA	NA	NA	NA	ST	ST	NA	CFnegative	Negative	A	-	L12+L14	2	7
E710	NA	NA	Global 1983-2008 (Mentzer et al)	2001	Guatemala	NA	NA	NA	NA	NA	ST	ST	NA	CFnegative	Negative	B1	-	L20	4	14
E806	NA	NA	Global 1983-2008 (Mentzer et al)	2002	Guatemala	NA	NA	NA	NA	NA	STp	ST	NA	CS6	Negative	A	-	L9+L10+L2	2	3
E810	NA	NA	Global 1983-2008 (Mentzer et al)	2002	Guatemala	NA	NA	NA	NA	NA	LT+STh	LT+ST	NA	CFA/I+CS21	eatA,etpBC	B1	-	L3	4	12
E811	NA	NA	Global 1983-2008 (Mentzer et al)	2002	Guatemala	NA	NA	NA	NA	NA	ST	ST	NA	CFnegative	Negative	A	532	L12+L14	2	7
E812	NA	NA	Global 1983-2008 (Mentzer et al)	2002	Guatemala	NA	NA	NA	NA	NA	STh	ST	NA	CS1+CS3+CS21	eatA,etpBC	A	215	L1	6	26
E816	NA	NA	Global 1983-2008 (Mentzer et al)	2002	Guatemala	NA	NA	NA	NA	NA	LT+STh	LT+ST	NA	CS3+CS21	etpBC	B1	-	L16	5	23
E818	NA	NA	Global 1983-2008 (Mentzer et al)	2002	Guatemala	NA	NA	NA	NA	NA	LT	LT	NA	CS13	Negative	B1	-	L18	4	16
E819	NA	NA	Global 1983-2008 (Mentzer et al)	2002	Guatemala	NA	NA	NA	NA	NA	LT	LT	NA	CFnegative	Negative	B1	-	L18	4	16
E821	NA	NA	Global 1983-2008 (Mentzer et al)	2002	Guatemala	NA	NA	NA	NA	NA	LT	LT	NA	CFnegative	Negative	B1	-	L18	4	16
E822	NA	NA	Global 1983-2008 (Mentzer et al)	2002	Guatemala	NA	NA	NA	NA	NA	LT+STh	LT+ST	NA	CS2+CS3+CS21	eatA,etpBC	A	-	L2	6	27
E828	NA	NA	Global 1983-2008 (Mentzer et al)	2002	Guatemala	NA	NA	NA	NA	NA	ST	ST	NA	CFnegative	Negative	A	-	L11+L15	2	4
E833	NA	NA	Global 1983-2008 (Mentzer et al)	2003	Guatemala	NA	NA	NA	NA	NA	LT	LT	NA	CS28A	Negative	B1	86	L18	4	16
E841	NA	NA	Global 1983-2008 (Mentzer et al)	2003	Guatemala	NA	NA	NA	NA	NA	LT	LT	NA	CFnegative	Negative	B1	-	NA	5	24
E842	NA	NA	Global 1983-2008 (Mentzer et al)	2003	Guatemala	NA	NA	NA	NA	NA	STp	ST	NA	CS6	Negative	B1	357	L17	4	11
E855	NA	NA	Global 1983-2008 (Mentzer et al)	2003	Guatemala	NA	NA	NA	NA	NA	LT	LT	NA	CFnegative	Negative	A	-	L11+L13	2	6
E856	NA	NA	Global 1983-2008 (Mentzer et al)	2003	Guatemala	NA	NA	NA	NA	NA	STp	ST	NA	CS6	Negative	A	-	L9+L10+L2	2	3
E858	NA	NA	Global 1983-2008 (Mentzer et al)	2003	Guatemala	NA	NA	NA	NA	NA	LT+STh	LT+ST	NA	CFA/I+CS21	eatA,etpBC	B1	-	L3	4	12
E860	NA	NA	Global 1983-2008 (Mentzer et al)	2003	Guatemala	NA	NA	NA	NA	NA	STp	ST	NA	CS6	Negative	A	-	L9+L10+L2	2	3
E863	NA	NA	Global 1983-2008 (Mentzer et al)	2003	Guatemala	NA	NA	NA	NA	NA	LT+STh	LT+ST	NA	CS1+CS3+CS21	eatA,etpBC	A	215	L1	6	26
E865	NA	NA	Global 1983-2008 (Mentzer et al)	2003	Guatemala	NA	NA	NA	NA	NA	STh	ST	NA	CS5+CS6	Negative	B1	5	L5	3	8
E867	NA	NA	Global 1983-2008 (Mentzer et al)	2003	Guatemala	NA	NA	NA	NA	NA	STh	ST	NA	CS5+CS6	eatA	B1	5	L5	3	8
E871	NA	NA	Global 1983-2008 (Mentzer et al)	2003	Guatemala	NA	NA	NA	NA	NA	STp	ST	NA	CS6	Negative	A	-	L9+L10+L2	2	3
E873	NA	NA	Global 1983-2008 (Mentzer et al)	2003	Guatemala	NA	NA	NA	NA	NA	LT+STp	LT+ST	NA	CS30	Negative	B1	866	L20	4	14
E876	NA	NA	Global 1983-2008 (Mentzer et al)	2003	Guatemala	NA	NA	NA	NA	NA	LT	LT	NA	CS20	Negative	A	398	L9+L10+L2	2	3
E877	NA	NA	Global 1983-2008 (Mentzer et al)	2003	Guatemala	NA	NA	NA	NA	NA	STh	ST	NA	CS5+CS6	eatA	B1	5	L5	3	8
E879	NA	NA	Global 1983-2008 (Mentzer et al)	2003	Guatemala	NA	NA	NA	NA	NA	STp	ST	NA	CS6	Negative	A	-	L9+L10+L2	2	3
E881	NA	NA	Global 1983-2008 (Mentzer et al)	2003	Guatemala	NA	NA	NA	NA	NA	LT+STp	LT+ST	NA	CS13+CS30	Negative	B1	866	L20	4	14
E882	NA	NA	Global 1983-2008 (Mentzer et al)	2003	Guatemala	NA	NA	NA	NA	NA	LT+STp	LT+ST	NA	CS12	Negative	A	1102	L11+L15	2	4
E883	NA	NA	Global 1983-2008 (Mentzer et al)	2003	Guatemala	NA	NA	NA	NA	NA	LT+ST	LT+ST	NA	CFnegative	Negative	A	999	L11+L13	2	6
E884	NA	NA	Global 1983-2008 (Mentzer et al)	2003	Guatemala	NA	NA	NA	NA	NA	LT	LT	NA	CFnegative	Negative	B1	87	L19	4	15
E885	NA	NA	Global 1983-2008 (Mentzer et al)	2003	Guatemala	NA	NA	NA	NA	NA	LT	LT	NA	CFnegative	Negative	B1	86	L18	4	16
E887	NA	NA	Global 1983-2008 (Mentzer et al)	2003	Guatemala	NA	NA	NA	NA	NA	ST	ST	NA	CFnegative	Negative	B1	21	L19	4	15
E888	NA	NA	Global 1983-2008 (Mentzer et al)	2003	Guatemala	NA	NA	NA	NA	NA	ST	ST	NA	CFnegative	Negative	B1	88	L20	4	14
E890	NA	NA	Global 1983-2008 (Mentzer et al)	2003	Guatemala	NA	NA	NA	NA	NA	ST	ST	NA	CFnegative	Negative	B1	-	L24	5	21
E891	NA	NA	Global 1983-2008 (Mentzer et al)	2003	Guatemala	NA	NA	NA	NA	NA	ST	ST	NA	CFnegative	Negative	B1	21	L19	4	15
E892	NA	NA	Global 1983-2008 (Mentzer et al)	2003	Guatemala	NA	NA	NA	NA	NA	ST	ST	NA	CFnegative	Negative	B1	21	L19	4	15
E895	NA	NA	Global 1983-2008 (Mentzer et al)	2003	Guatemala	NA	NA	NA	NA	NA	STp	ST	NA	CS6	Negative	B1	357	L17	4	11
E897	NA	NA	Global 1983-2008 (Mentzer et al)	2003	Guatemala	NA	NA	NA	NA	NA	STh	ST	NA	CFA/I+CS21	eatA,etpBC	A	-	L6	5	22
E898	NA	NA	Global 1983-2008 (Mentzer et al)	2003	Guatemala	NA	NA	NA	NA	NA	LT	LT	NA	CS6	Negative	A	-	L11+L15	2	4
E899	NA	NA	Global 1983-2008 (Mentzer et al)	2003	Guatemala	NA	NA	NA	NA	NA	LT	LT	NA	CFnegative	Negative	B1	86	L18	4	16
E900	NA	NA	Global 1983-2008 (Mentzer et al)	2003	Guatemala	NA	NA	NA	NA	NA	LT	LT	NA	CS17	Negative	B1	5	L5	3	8
E901	NA	NA	Global 1983-2008 (Mentzer et al)	2003	Guatemala	NA	NA	NA	NA	NA	LT	LT	NA	CS8	Negative	D	77	L28	8	36
E903	NA	NA	Global 1983-2008 (Mentzer et al)	2003	Guatemala	NA	NA	NA	NA	NA	LT+STh	LT+ST	NA	CS2+CS3+CS21	eatA,etpBC	A	215	L2	6	27
E907	NA	NA	Global 1983-2008 (Mentzer et al)	2004	Guatemala	NA	NA	NA	NA	NA	STh	ST	NA	CS14	eatA,etpBC	B1	87	L19	4	15
E908	NA	NA	Global 1983-2008 (Mentzer et al)	2004	Guatemala	NA	NA	NA	NA	NA	ST	ST	NA	CFnegative	Negative	B1	-	L17	4	11
E916	NA	NA	Global 1983-2008 (Mentzer et al)	2003	Guatemala	NA	NA	NA	NA	NA	STh	ST	NA	CS5+CS6	eatA	B1	5	L5	3	8

E917	NA	NA	Global 1983-2008 (Mentzer et al)	2003	Guatemala	NA	NA	NA	NA	NA	STh	ST	NA	CFA/I+CS21	eatA,etpBC	B1	-	L3	4	12
E920	NA	NA	Global 1983-2008 (Mentzer et al)	2003	Guatemala	NA	NA	NA	NA	NA	STh	ST	NA	CS14	eatA,etpBC	B1	87	L19	4	15
E924	NA	NA	Global 1983-2008 (Mentzer et al)	2003	Guatemala	NA	NA	NA	NA	NA	STh	ST	NA	CS21	etpBC	A	155	L4	6	28
E925	NA	NA	Global 1983-2008 (Mentzer et al)	2003	Guatemala	NA	NA	NA	NA	NA	LT+STh	LT+ST	NA	CS1+CS3+CS21	eatA,etpBC	A	215	L1	6	26
E927	NA	NA	Global 1983-2008 (Mentzer et al)	1997	Egypt	NA	NA	NA	NA	NA	STp	ST	NA	CS6	Negative	A	-	L9+L10+L2	2	3
E928	NA	NA	Global 1983-2008 (Mentzer et al)	1997	Egypt	NA	NA	NA	NA	NA	STh	ST	NA	CS14	etpBC	B1	366	L20	4	14
E934	NA	NA	Global 1983-2008 (Mentzer et al)	1997	Egypt	NA	NA	NA	NA	NA	LT+STp	LT+ST	NA	CS19	eatA,etpBC	A	-	L9+L10+L2	2	3
E935	NA	NA	Global 1983-2008 (Mentzer et al)	1997	Egypt	NA	NA	NA	NA	NA	LT+STp	LT+ST	NA	CS19	eatA,etpBC	A	-	L9+L10+L2	2	3
E936	NA	NA	Global 1983-2008 (Mentzer et al)	1997	Egypt	NA	NA	NA	NA	NA	LT+ST	LT+ST	NA	CFnegative	Negative	B1	-	L23	4	17
E938	NA	NA	Global 1983-2008 (Mentzer et al)	1997	Egypt	NA	NA	NA	NA	NA	LT+STh	LT+ST	NA	CS1+CS3+CS21	eatA,etpBC	A	215	L1	6	26
E939	NA	NA	Global 1983-2008 (Mentzer et al)	1997	Egypt	NA	NA	NA	NA	NA	ST	ST	NA	CS27A	Negative	B1	-	L20	4	14
E940	NA	NA	Global 1983-2008 (Mentzer et al)	1997	Egypt	NA	NA	NA	NA	NA	STp	ST	NA	CS6	Negative	A	-	L9+L10+L2	2	3
E941	NA	NA	Global 1983-2008 (Mentzer et al)	1997	Egypt	NA	NA	NA	NA	NA	LT	LT	NA	CFnegative	Negative	A	-	L11+L15	2	4
E943	NA	NA	Global 1983-2008 (Mentzer et al)	1997	Egypt	NA	NA	NA	NA	NA	STp	ST	NA	CS6	Negative	A	-	L9+L10+L2	2	3
E944	NA	NA	Global 1983-2008 (Mentzer et al)	1997	Egypt	NA	NA	NA	NA	NA	LT+STp	LT+ST	NA	CS19	eatA,etpBC	A	-	L9+L10+L2	2	3
E945	NA	NA	Global 1983-2008 (Mentzer et al)	1997	Egypt	NA	NA	NA	NA	NA	LT+STp	LT+ST	NA	CS19	eatA,etpBC	A	-	L9+L10+L2	2	3
E947	NA	NA	Global 1983-2008 (Mentzer et al)	1997	Egypt	NA	NA	NA	NA	NA	LT+STp	LT+ST	NA	CS27A	Negative	A	-	L11+L15	2	4
E949	NA	NA	Global 1983-2008 (Mentzer et al)	1997	Egypt	NA	NA	NA	NA	NA	LT	LT	NA	CFnegative	etpBC	A	-	L25	6	29
E952	NA	NA	Global 1983-2008 (Mentzer et al)	1997	Egypt	NA	NA	NA	NA	NA	LT	LT	NA	CS13+CS26	Negative	A	-	L11+L13	2	6
E953	NA	NA	Global 1983-2008 (Mentzer et al)	1997	Egypt	NA	NA	NA	NA	NA	LT+STh	LT+ST	NA	CS1+CS3+CS21	eatA,etpBC	A	215	L1	6	26
E955	NA	NA	Global 1983-2008 (Mentzer et al)	1997	Egypt	NA	NA	NA	NA	NA	LT+STp	LT+ST	NA	CS19	eatA,etpBC	A	-	L9+L10+L2	2	3
E956	NA	NA	Global 1983-2008 (Mentzer et al)	1997	Egypt	NA	NA	NA	NA	NA	LT	LT	NA	CFnegative	etpBC	A	-	L25	6	29
E957	NA	NA	Global 1983-2008 (Mentzer et al)	1997	Egypt	NA	NA	NA	NA	NA	LT	LT	NA	CFnegative	etpBC	A	-	L25	6	29
E978	NA	NA	Global 1983-2008 (Mentzer et al)	1997	Egypt	NA	NA	NA	NA	NA	LT+STp	LT+ST	NA	CS19	eatA,etpBC	A	-	L9+L10+L2	2	3
E986	NA	NA	Global 1983-2008 (Mentzer et al)	1997	Egypt	NA	NA	NA	NA	NA	STh	ST	NA	CFA/I+CS21	eatA,etpBC	A	-	L6	5	22
E995	NA	NA	Global 1983-2008 (Mentzer et al)	1997	Egypt	NA	NA	NA	NA	NA	LT+STh	LT+ST	NA	CS1+CS3+CS21	eatA,etpBC	A	215	L1	6	26
E996	NA	NA	Global 1983-2008 (Mentzer et al)	1997	Egypt	NA	NA	NA	NA	NA	STh	ST	NA	CS6	Negative	NA	-	NA	9	39
E998	NA	NA	Global 1983-2008 (Mentzer et al)	1997	Egypt	NA	NA	NA	NA	NA	ST	ST	NA	CS27A	Negative	B1	469	L23	4	17
