# Supplementary material for: Genomic insights into a diarrheal outbreak in Bangladesh reveal novel ETEC lineages and expansion of CS23 colonization factor
Source: Microbiol Spectr. 2025 Sep 10;13(10):e03315-24. doi: 10.1128/spectrum.03315-24 (PMC12502627; doi:10.1128/spectrum.03315-24)
Supplement: Table S2 — Data set of plasmid profile for a total of 836 ETEC isolates, including 475 Bangladeshi ETEC isolates of this study. [file spectrum.03315-24-s0004.pdf]

Table S2: Dataset of plasmid profile for total 836 ETEC isolates including 475 Bangladeshi ETEC isolates of this study.

Strain ID	Study dataset	lineage	CF	year	Col156	Col440I	Col8282	ColE10	ColRNAI	Col_BS512	Col_IRGK	Col_MG828	Col_pHAD28	ColpEC648	IncB/O/K/Z	IncFI	IncFIA	IncFIB	IncFIB_AP001918	IncFIB_H89_PhagePlasmid	IncFIB(K)	IncFIB(pHCM2)	IncFIB(pLF82 PhagePlasmid)	IncFII(p)	IncFII_pEH01	IncHI1A	IncHI1B	IncI	IncI2	IncR	IncX1	IncX4	IncX4-1	IncY	p0111
E1003	Global 1983-2008 (Mentzer et al)	L5	CS5+CS6	1999	0	0	0	0	0	0	0	1	0	0	0	1	0	0	0	1	0	0	0	0	0	0	0	0	0	0	0	0	1	0	0
E1009	Global 1983-2008 (Mentzer et al)	L3	CS7	1999	0	0	0	0	1	0	0	0	1	0	1	1	0	0	0	0	0	0	0	0	0	0	0	1	0	0	0	0	0	0	0
E1018	Global 1983-2008 (Mentzer et al)	L2	CS2+CS3+CS21	1999	0	0	0	0	0	0	0	0	1	0	0	1	0	0	1	0	0	0	0	0	0	0	0	0	0	0	0	0	0	0	0
E1034	Global 1983-2008 (Mentzer et al)	L5	CS5+CS6	1999	0	0	0	0	0	0	0	0	0	0	0	1	0	0	0	0	0	0	0	0	0	0	0	0	0	0	0	0	0	0	0
E1057	Global 1983-2008 (Mentzer et al)	L11	CS27A	2000	0	0	0	0	0	0	0	0	0	0	0	1	0	0	1	0	0	0	0	0	0	0	0	0	0	0	0	0	0	0	0
E106	Global 1983-2008 (Mentzer et al)	Not defined	CS6	1985	0	0	0	0	0	0	0	0	0	0	1	1	0	0	0	0	0	0	0	1	0	0	0	0	0	0	0	0	0	0	0
E1072	Global 1983-2008 (Mentzer et al)	Not defined	CS20	2000	0	0	0	0	0	0	0	0	0	0	0	1	0	0	0	0	0	0	0	0	0	0	0	0	0	0	0	0	0	0	0
E1074	Global 1983-2008 (Mentzer et al)	L1	CS1+CS3+CS21	2000	0	0	0	0	1	0	0	0	1	0	0	1	0	0	1	0	0	0	0	0	0	0	0	1	0	0	0	0	0	0	0
E1085	Global 1983-2008 (Mentzer et al)	L5	CS5+CS6	2000	0	0	0	0	0	0	0	0	0	0	0	1	0	0	0	0	0	0	0	0	0	0	0	0	0	0	0	0	0	0	0
E1091	Global 1983-2008 (Mentzer et al)	L15	CFA/I+CS21	2000	0	0	0	0	0	0	0	0	0	0	1	1	0	0	1	0	0	0	0	0	0	0	0	0	0	0	1	0	0	0	0
E110	Bangladesh 1980-2011 (Mentzer et al)	L5	CS5+CS6	1985	0	0	0	0	0	0	0	0	0	0	1	0	0	0	0	0	0	0	0	0	0	0	0	0	0	0	0	0	0	0	0
E1101	Global 1983-2008 (Mentzer et al)	L15	CS30	2000	0	0	0	0	0	0	0	0	0	0	0	1	0	0	0	0	0	0	0	0	0	0	0	0	0	0	1	0	0	0	0
E1102	Global 1983-2008 (Mentzer et al)	L16	CFnegative	2000	0	0	0	0	0	0	0	0	0	0	0	1	0	0	0	0	0	0	0	0	0	0	0	1	0	0	0	0	1	0	0
E1111	Global 1983-2008 (Mentzer et al)	L5	CS5+CS6	2000	0	0	0	0	0	0	0	0	0	0	0	1	0	0	0	0	0	0	0	0	0	0	0	0	0	0	1	0	0	0	0
E1114	Global 1983-2008 (Mentzer et al)	L4	CS6+CS8	2001	0	0	0	0	0	0	0	1	0	1	0	1	1	0	0	0	0	0	0	0	0	0	0	0	0	0	1	0	1	1	0
E1115	Global 1983-2008 (Mentzer et al)	L4	CS6+CS8	2001	0	0	0	0	0	0	0	0	0	0	0	1	0	0	0	0	0	0	0	0	0	0	0	0	0	0	0	0	0	0	0
E1116	Global 1983-2008 (Mentzer et al)	L4	CS6+CS8	2001	0	0	0	0	0	0	0	0	0	0	0	1	0	0	0	0	0	0	0	0	0	0	0	0	0	0	0	0	0	0	0
E1117	Global 1983-2008 (Mentzer et al)	L4	CS6+CS8	2001	0	0	0	0	0	0	0	0	0	0	0	1	0	0	0	0	0	0	0	0	0	0	0	0	0	0	0	0	0	0	0
E1118	Global 1983-2008 (Mentzer et al)	L4	CS6+CS8	2001	0	0	0	0	0	0	0	1	1	0	0	1	0	0	0	0	1	0	0	0	0	1	1	0	0	0	0	0	0	0	0
E1119	Global 1983-2008 (Mentzer et al)	L4	CS6+CS21	2001	0	0	0	0	0	0	0	0	0	0	0	1	0	0	1	0	0	0	0	0	0	0	0	0	0	0	0	0	0	0	0
E1120	Global 1983-2008 (Mentzer et al)	L4	CS6+CS21	2001	0	0	0	0	0	0	0	0	0	0	0	1	0	0	1	0	0	0	0	0	0	0	0	0	0	0	0	0	0	0	0
E1121	Global 1983-2008 (Mentzer et al)	L4	CS6+CS21	2001	0	0	0	0	0	0	0	0	0	0	0	1	0	0	1	0	0	0	0	0	0	0	0	0	0	0	0	0	0	0	0
E1122	Global 1983-2008 (Mentzer et al)	L4	CS6+CS21	2001	0	0	0	0	0	0	0	0	0	0	0	1	0	0	1	0	0	0	0	0	0	0	0	0	0	0	0	0	0	0	0
E1167	Global 1983-2008 (Mentzer et al)	L10	CS19	2001	0	0	0	0	1	0	0	0	1	0	0	1	0	0	0	0	0	0	0	0	1	0	0	0	0	0	0	0	0	0	0
E1169	Global 1983-2008 (Mentzer et al)	Not defined	CS20	2001	0	0	0	0	0	0	0	0	0	0	0	1	0	0	0	0	0	0	0	0	0	0	0	0	0	0	0	0	0	0	0
E1189	Global 1983-2008 (Mentzer et al)	L8	CS6+CS21	2001	0	0	0	0	0	0	0	0	0	0	0	1	0	0	1	0	0	0	0	1	0	0	0	0	0	0	0	0	0	0	0
E1193	Global 1983-2008 (Mentzer et al)	L14	CFnegative	2001	0	0	0	0	0	0	0	0	0	0	0	0	0	0	0	0	0	0	0	0	0	0	0	0	0	0	0	0	0	0	0
E1242	Global 1983-2008 (Mentzer et al)	Not defined	CS18	2001	0	0	0	0	0	1	0	0	0	0	0	1	0	1	0	0	0	0	0	0	0	0	0	0	0	0	0	0	0	0	0
E1245	Global 1983-2008 (Mentzer et al)	L7	CS6	2001	0	0	0	0	1	0	0	0	0	0	0	1	0	0	1	0	0	0	0	0	0	0	0	0	0	0	0	0	0	0	0
E1248	Global 1983-2008 (Mentzer et al)	L10	CS19	2001	0	0	0	0	1	0	0	0	1	0	0	1	0	0	0	0	0	0	0	1	0	0	0	0	0	0	0	0	0	0	0
E1258	Global 1983-2008 (Mentzer et al)	L10	CS19	2001	0	0	0	0	1	0	0	0	1	0	0	1	0	0	0	0	0	0	0	0	0	0	0	0	0	0	0	0	0	0	0
E126	Global 1983-2008 (Mentzer et al)	L19	CS14	1989	0	0	0	0	0	0	0	0	0	0	0	1	0	0	1	0	0	0	0	0	0	0	0	0	0	0	0	0	0	0	0
E1264	Global 1983-2008 (Mentzer et al)	L10	CS19	2001	0	0	0	0	1	0	0	0	1	0	0	1	0	0	0	0	0	0	0	0	0	0	0	0	0	0	0	0	0	0	0
E1281	Global 1983-2008 (Mentzer et al)	L10	CS19	2001	0	0	0	0	1	0	0	0	1	0	0	1	0	0	0	0	0	0	0	0	0	0	0	0	0	0	0	0	0	0	0
E1282	Global 1983-2008 (Mentzer et al)	L24	CFnegative	2001	0	0	0	0	0	0	0	0	0	0	0	0	0	0	0	0	0	0	0	0	0	0	0	0	0	0	0	0	0	0	0
E1285	Global 1983-2008 (Mentzer et al)	L10	CS19	2001	0	0	0	0	1	0	0	0	1	0	0	1	0	0	0	0	0	0	0	0	0	0	0	0	0	0	0	0	0	0	0
E1287	Global 1983-2008 (Mentzer et al)	L15	CFA/I+CS21	2001	1	0	0	0	0	0	0	0	0	1	0	0	1	0	1	0	0	0	0	1	0	0	0	0	0	0	0	0	0	0	0
E129	Global 1983-2008 (Mentzer et al)	L17	CS17	1989	0	0	0	0	0	0	0	1	0	0	0	1	0	0	0	0	0	0	0	1	0	0	0	0	0	0	0	0	0	0	0
E1298	Global 1983-2008 (Mentzer et al)	L5	CS5+CS6	2001	0	0	0	0	0	0	0	0	1	0	0	1	0	0	0	0	0	0	0	0	0	0	0	0	0	0	1	0	0	0	0
E1316	Global 1983-2008 (Mentzer et al)	L1	CS1+CS3+CS21	1996	0	0	0	0	1	0	0	0	0	0	0	1	0	0	1	0	0	0	0	1	0	0	0	1	0	0	0	0	0	0	0
E1328	Global 1983-2008 (Mentzer et al)	L7	CS6	1996	0	0	0	0	0	0	0	1	0	0	0	1	0	0	1	0	0	0	0	0	0	0	0	0	0	0	0	0	0	0	0
E1329	Global 1983-2008 (Mentzer et al)	L7	CS6	1996	0	0	0	0	0	0	0	0	0	0	0	1	0	0	1	0	0	0	0	0	0	0	0	0	0	0	0	0	0	0	0
E133	Global 1983-2008 (Mentzer et al)	L15	CS12	1989	0	0	0	0	0	0	0	0	0	0	0	1	0	0	0	0	0	0	0	0	0	0	0	0	0	0	0	0	0	0	0
E1334	Global 1983-2008 (Mentzer et al)	L7	CS6	1996	0	0	0	0	0	0	0	0	0	0	0	1	0	0	1	0	0	0	0	0	0	0	0	0	0	0	0	0	0	0	0

E855	Global 1983-2008 (Mentzer et al)	L13	CFnegative	2003	0	0	0	0	0	1	0
E856	Global 1983-2008 (Mentzer et al)	L9	CS6	2003	0	0	0	0	0	0	0
E858	Global 1983-2008 (Mentzer et al)	L3	CFA/I+CS21	2003	0	0	0	0	0	0	0
E860	Global 1983-2008 (Mentzer et al)	L9	CS6	2003	0	0	0	0	0	0	0
E863	Global 1983-2008 (Mentzer et al)	L1	CS1+CS3+CS21	2003	0	0	0	0	1	0	0
E865	Global 1983-2008 (Mentzer et al)	L5	CS5+CS6	2003	0	0	0	0	1	0	0
E867	Global 1983-2008 (Mentzer et al)	L5	CS5+CS6	2003	0	0	0	0	0	0	0
E87	Bangladesh 1980-2011 (Mentzer et al)	L2	CS2+CS3+CS21	1988	0	0	0	0	0	0	0
E871	Global 1983-2008 (Mentzer et al)	L9	CS6	2003	0	0	0	0	0	0	0
E873	Global 1983-2008 (Mentzer et al)	L20	CS30	2003	0	0	0	0	0	0	0
E876	Global 1983-2008 (Mentzer et al)	L21	CS20	2003	0	0	0	0	0	0	0
E877	Global 1983-2008 (Mentzer et al)	L5	CS5+CS6	2003	0	0	0	0	0	0	0
E879	Global 1983-2008 (Mentzer et al)	L9	CS6	2003	0	0	0	0	0	0	0
E88	Bangladesh 1980-2011 (Mentzer et al)	L5	CS5+CS6	1988	0	0	0	0	0	0	0
E881	Global 1983-2008 (Mentzer et al)	L20	CS13+CS30	2003	0	0	0	0	0	0	0
E882	Global 1983-2008 (Mentzer et al)	L15	CS12	2003	0	0	0	0	0	0	0
E883	Global 1983-2008 (Mentzer et al)	L11	CFnegative	2003	0	0	0	0	0	0	0
E884	Global 1983-2008 (Mentzer et al)	L19	CFnegative	2003	0	0	0	0	0	0	0
E885	Global 1983-2008 (Mentzer et al)	L18	CFnegative	2003	0	0	0	0	0	0	0
E887	Global 1983-2008 (Mentzer et al)	L19	CFnegative	2003	0	0	0	0	1	0	0
E888	Global 1983-2008 (Mentzer et al)	L5	CFnegative	2003	0	0	0	0	0	0	0
E890	Global 1983-2008 (Mentzer et al)	L24	CFnegative	2003	0	0	0	0	0	1	0
E891	Global 1983-2008 (Mentzer et al)	L19	CFnegative	2003	0	0	0	0	0	1	0
E892	Global 1983-2008 (Mentzer et al)	L19	CFnegative	2003	0	0	0	0	0	0	0
E895	Global 1983-2008 (Mentzer et al)	L17	CS6	2003	0	0	0	0	0	0	0
E897	Global 1983-2008 (Mentzer et al)	L6	CFA/I+CS21	2003	0	0	0	0	0	0	0
E898	Global 1983-2008 (Mentzer et al)	L15	CS6	2003	0	0	0	0	1	0	0
E899	Global 1983-2008 (Mentzer et al)	L18	CFnegative	2003	0	0	0	0	0	0	0
E900	Global 1983-2008 (Mentzer et al)	L5	CS17	2003	0	0	0	0	0	0	0
E901	Global 1983-2008 (Mentzer et al)	Not defined	CS8	2003	0	0	0	0	0	0	0
E903	Global 1983-2008 (Mentzer et al)	L2	CS2+CS3+CS21	2003	0	0	0	0	0	0	0
E907	Global 1983-2008 (Mentzer et al)	L19	CS14	2004	0	0	0	0	0	0	0
E908	Global 1983-2008 (Mentzer et al)	L17	CFnegative	2004	0	0	0	0	0	1	0

GVE0312	Bangladesh 2022-2023 (This study)	L1	CS1+CS3+CS21	2022	0	0	0	0	1	0	0	0	0	0	0	1	0	0	1	0	0	0	0	1	0	0	0	1	0	0	0	0	0	0	0
GVE0313	Bangladesh 2012-2021 (This study)	L26	CFnegative	2016	0	0	0	0	0	0	0	0	0	0	0	1	1	0	1	0	0	0	0	0	0	0	0	1	0	0	0	0	0	0	0
GVE0314	Bangladesh 2022-2023 (This study)	L18	F41a	2022	0	0	0	0	1	0	0	0	1	0	1	1	0	0	1	0	0	0	0	0	0	0	0	0	0	0	0	0	0	0	0
GVE0315	Bangladesh 2022-2023 (This study)	L5	CS6	2022	0	0	0	0	1	0	0	0	0	0	0	1	0	0	1	0	0	0	0	0	0	1	1	0	0	0	0	0	0	0	0
GVE0316	Bangladesh 2022-2023 (This study)	L3	CS7	2022	0	0	0	0	1	0	0	0	0	0	0	1	0	0	0	0	0	0	0	0	0	0	0	1	0	0	0	0	0	0	0
GVE0317	Bangladesh 2022-2023 (This study)	L4	CS6+CS21	2022	0	0	0	0	0	0	0	0	0	0	1	1	0	0	1	0	0	0	0	0	0	0	0	0	0	0	0	0	0	0	0
GVE0318	Bangladesh 2022-2023 (This study)	Not defined	CS12	2022	0	0	0	0	1	0	0	0	0	0	0	1	0	0	0	0	0	0	0	0	0	0	0	0	0	0	0	0	0	0	0
GVE0319	Bangladesh 2022-2023 (This study)	L26	CS14	2022	0	0	0	0	0	0	0	0	0	0	1	1	0	0	1	0	0	0	0	0	0	0	0	0	0	0	0	0	0	0	0
GVE0320	Bangladesh 2022-2023 (This study)	L4	CS6+CS8	2022	0	0	0	0	0	0	0	0	0	0	0	1	0	0	0	0	0	0	0	0	0	0	0	0	0	0	0	0	0	0	0
GVE0321	Bangladesh 2012-2021 (This study)	L26	CFnegative	2016	0	0	0	0	0	0	0	0	0	0	0	1	1	0	1	0	0	0	0	0	0	0	0	0	0	0	0	0	0	0	0
GVE0322	Bangladesh 2022-2023 (This study)	L6	CFA/I+CS21	2022	0	0	0	0	0	0	0	0	1	0	1	1	0	0	1	0	0	0	0	0	0	0	0	0	0	0	0	0	0	0	0
GVE0323	Bangladesh 2012-2021 (This study)	L11	CFnegative	2017	0	0	0	0	0	0	0	0	0	0	0	1	1	0	0	0	0	0	0	0	0	0	0	1	0	0	0	1	0	0	0
GVE0324	Bangladesh 2022-2023 (This study)	L3	CS7	2022	0	0	0	0	1	0	0	0	0	0	1	1	0	0	0	0	0	0	0	0	0	0	0	1	0	0	0	0	0	0	0
GVE0325	Bangladesh 2022-2023 (This study)	Not defined	S1+CS3+CS12+C	2022	0	0	0	0	1	0	0	0	0	0	0	1	0	0	1	0	0	0	0	0	1	0	0	1	0	0	0	0	0	0	0
GVE0326	Bangladesh 2012-2021 (This study)	L26	CFnegative	2013	0	0	0	0	1	0	0	0	1	0	0	1	1	0	1	0	0	0	0	0	0	0	0	1	0	0	0	0	0	0	0
GVE0327	Bangladesh 2022-2023 (This study)	Not defined	CS23	2022	0	0	0	0	1	0	0	0	0	0	0	1	1	0	0	1	0	0	0	0	0	0	0	1	0	0	0	1	0	0	0
GVE0328	Bangladesh 2022-2023 (This study)	L19	CS23	2022	0	0	0	0	1	0	0	0	0	0	0	1	1	0	1	0	0	0	0	0	0	0	0	1	0	0	0	0	0	0	0
GVE0329	Bangladesh 2022-2023 (This study)	L4	CS6+CS8	2022	0	0	0	0	0	0	0	0	0	0	0	1	0	0	0	0	0	0	0	0	0	0	0	0	0	0	0	0	0	0	0
GVE0330	Bangladesh 2022-2023 (This study)	L19	CS23	2022	1	0	0	0	1	0	0	1	0	1	0	1	1	0	0	0	0	0	0	0	0	0	0	1	0	0	0	1	0	0	0
GVE0331	Bangladesh 2022-2023 (This study)	Not defined	CS21	2022	0	0	0	0	0	0	0	0	0	0	0	1	0	0	1	0	0	0	0	0	0	0	0	0	0	0	0	0	0	0	0
GVE0332	Bangladesh 2022-2023 (This study)	Not defined	CS20	2022	0	0	0	1	0	0	0	0	0	0	0	1	0	0	0	0	0	0	0	0	0	0	0	0	0	0	0	0	0	0	0
GVE0333	Bangladesh 2022-2023 (This study)	L5	CS5+CS6	2022	0	0	0	0	0	0	0	0	0	0	0	1	0	0	0	0	0	0	0	0	0	0	0	0	0	0	0	0	0	0	0
GVE0334	Bangladesh 2022-2023 (This study)	L5	CS5+CS6	2022	0	0	0	0	0	0	0	0	0	0	0	1	0	0	0	1	0	0	0	0	0	0	0	0	0	0	0	0	0	0	0
GVE0335	Bangladesh 2022-2023 (This study)	L5	CS23	2022	0	0	0	0	1	0	0	0	0	0	0	1	1	0	1	0	0	0	0	0	0	0	0	1	0	0	0	0	0	0	0
GVE0336	Bangladesh 2022-2023 (This study)	L18	CS6	2022	0	0	0	0	0	0	0	0	0	0	0	1	0	0	1	0	0	0	0	0	0	0	0	0	0	0	0	0	0	0	0
GVE0337	Bangladesh 2012-2021 (This study)	L15	CFnegative	2017	1	0	0	0	1	0	0	1	0	0	0	1	1	0	0	0	0	0	0	0	0	0	0	1	0	0	1	0	0	0	0
GVE0338	Bangladesh 2022-2023 (This study)	L1	CS1+CS3+CS21	2022	0	0	0	0	1	0	0	0	0	0	1	1	0	0	1	0	0	0	0	1	0	0	0	1	0	0	0	0	0	0	0
GVE0340	Bangladesh 2022-2023 (This study)	L19	CS23	2022	1	0	0	0	1	0	0	1	1	1	0	1	1	0	0	0	0	0	0	0	0	0	0	1	0	0	0	1	0	0	0
GVE0341	Bangladesh 2022-2023 (This study)	L13	CS23	2022	0	0	0	0	0	0	0	0	0	0	0	1	1	0	1	0	0	0	0	0	0	0	0	1	0	0	0	1	0	0	0
GVE0342	Bangladesh 2022-2023 (This study)	L17	CS6	2022	0	0	0	0	1	0	0	0	1	0	0	1	0	0	1	0	0	0	0	0	0	0	0	1	0	0	0	0	0	0	0
GVE0343	Bangladesh 2022-2023 (This study)	L4	CS6+CS21	2022	0	0	0	0	0	0	0	0	0	0	0	1	0	0	1	0	0	0	0	1	0	0	0	0	0	0	0	0	0	0	0
GVE0344	Bangladesh 2022-2023 (This study)	L2	CS2+CS3+CS21	2022	0	0	0	0	1	0	0	0	0	0	0	1	0	0	1	0	0	0	0	1	0	0	0	1	0	0	0	0	0	0	0
GVE0345	Bangladesh 2022-2023 (This study)	Not defined	CS12	2022	0	0	0	0	1	0	0	0	0	0	0	1	0	0	0	0	0	0	0	0	0	0	0	0	0	0	0	0	0	0	0
GVE0346	Bangladesh 2012-2021 (This study)	L18	CFnegative	2012	0	0	0	0	0	0	0	0	0	0	0	0	0	1	0	0	0	0	0	1	0	0	0	0	0	0	0	0	0	1	0
GVE0347	Bangladesh 2022-2023 (This study)	L16	CS17	2022	0	0	0	0	0	0	0	0	0	0	1	1	0	0	0	0	0	0	0	0	0	0	0	0	1	0	0	0	0	0	0
GVE0348	Bangladesh 2022-2023 (This study)	Not defined	CS12	2022	0	0	0	0	1	0	0	0	0	0	0	1	0	0	0	0	0	0	0	0	0	0	0	0	0	0	0	0	0	0	0
GVE0349	Bangladesh 2012-2021 (This study)	L5	CFnegative	2017	0	0	0	0	1	0	0	0	0	0	0	1	1	0	1	0	0	0	0	0	0	0	0	1	0	0	0	0	0	0	0
GVE0350	Bangladesh 2012-2021 (This study)	L11	CFnegative	2015	0	0	0	0	0	0	0	0	1	0	0	1	0	0	0	0	0	0	0	0	0	0	0	0	0	0	0	0	0	0	0
GVE0351	Bangladesh 2022-2023 (This study)	Not defined	CFA/I+CS21	2022	0	0	0	0	0	0	0	0	0	0	0	1	0	0	1	0	0	0	0	1	0	0	0	0	0	0	0	0	0	0	0
GVE0352	Bangladesh 2022-2023 (This study)	Not defined	CS12	2022	0	0	0	0	1	0	0	0	0	0	0	1	0	0	1	0	0	0	0	0	0	0	0	0	0	0	0	0	0	0	0
GVE0353	Bangladesh 2012-2021 (This study)	L25	CFnegative	2017	0	0	0	0	0	0	0	0	0	0	0	1	1	0	0	0	0	0	0	0	0	0	0	1	0	0	0	0	0	0	0
GVE0354	Bangladesh 2022-2023 (This study)	L3	CFA/I+CS21	2022	0	0	0	0	0	0	0	0	0	0	1	1	0	0	1	0	0	0	0	0	0	0	0	0	0	0	0	0	0	0	0
GVE0355	Bangladesh 2012-2021 (This study)	L17	CFnegative	2013	0	0	0	0	1	0	0	0	0	0	0	1	0	0	1	0	0	0	0	0	0	0	0	0	0	0	0	0	0	0	0
GVE0356	Bangladesh 2022-2023 (This study)	L7	CS6	2022	0	0	0	0	0	0	0	0	0	0	0	1	0	0	1	0	0	0	0	0	0	0	0	0	0	0	0	0	0	0	0
GVE0357	Bangladesh 2012-2021 (This study)	L26	CFnegative	2012	0	0	0	0	1	0	0	0	0	0	0	1	1	1	0	0	0	0	0	0	0	0	0	1	0	0	0	0	0	0	0
GVE0358	Bangladesh 2022-2023 (This study)	Not defined	CS6	2022	0	0	0	0	0	0	0	1	0	0	1	1	0	0	1	0	0	0	0	1	0	0	0	0	1	0	0	0	0	0	0
GVE0359	Bangladesh 2012-2021 (This study)	L5	CFnegative	2013	0	0	0	0	0	0	0	0	0	0	0	1	0	0	1	0	0	0	0	0	0	0	0	0	0	0	0	0	0	0	0
GVE0360	Bangladesh 2022-2023 (This study)	L26	CFnegative	2022	0	0	0	0	1	0	0	0	0	0	0	1	1	0	0	0	0	0	0	0	0	0	0	1	0	0	0	0	0	0	0
GVE0361	Bangladesh 2022-2023 (This study)	L19	CS6	2022	0	0	0	0	0	0	0	1	0	0	0	1	0	0	1	0	0	0	0	0	0	0	0	0	1	0	0	0	0	0	0
GVE0363	Bangladesh 2022-2023 (This study)	L3	CFA/I+CS21	2022	0	0	0	0	0	0	0	0	0	0	1	1	0	0	1	0	0	0	0	1	0	0	0	0	0	0	0	0	0	0	0
GVE0364	Bangladesh 2022-2023 (This study)	L5	CS5+CS6	2022	0	0	0	0	0	0	0	0	0	0	0	1	0	0	0	1	0	0	0	0	0	0	0	0	0	0	0	0	0	0	0
GVE0365	Bangladesh 2022-2023 (This study)	L19	CS13+CS27A	2022	0	0	0	0	0	0	0	0	0	0	0	1	0	0	1	0	0	0	0	0	0	0	0	0	0	0	0	0	0	0	0
GVE0366	Bangladesh 2022-2023 (This study)	L19	CS23	2022	0	0	0	0	1	0	0	0	0	0	0	1	1	0	1	0	0	0	0	1	0	1	1	1	0	0	0	0	0	0	0
GVE0367	Bangladesh 2022-2023 (This study)	Not defined	CS28A	2022	1	0	0	0	0	1	0	0	1	0	0	1	1	0	1	0	1	0	0	0	0	0	0	0	0	1	0	0	0	1	0
GVE0368	Bangladesh 2022-2023 (This study)	L2	CS2+CS3+CS21	2022	0	0	0	0	1	0	0	0	0	0	0	1	0	0	1	0	0	0	0	0	0	0	0	0	0	0	1	0	0	0	0
GVE0369	Bangladesh 2022-2023 (This study)	Not defined	CFnegative	2022	1	1	0	0	1	0	0	1	0	0	0	1	1	0	1	0	0	0	0	1	0	0	0	1	0	0	0	0	0	0	1
GVE0370	Bangladesh 2012-2021 (This study)	Not defined	CFnegative	2020	0	0	0	0	0	0	0	0	0	0	0	1	0	0	1	1	0	0	0	0	0	0	0	0	0	0	0	0	0	0	0
GVE0388	Bangladesh 2022-2023 (This study)	L22	CS12	2023	0	0	0	0	0	0	0	0	0	0	0	1	0	0	0	0	0	0	0	0	0	0	0	0	0	0	0	0	0	0	0
GVE0400	Bangladesh 2022-2023 (This study)	L17	CFnegative	2023	0	0	0	0	0	0	0	0	0	0	0	1	1	0	0	0	0	0	0	0	0	0	0	1	0	0	0	0	0	0	1
GVE0401	Bangladesh 2022-2023 (This study)	Not defined	CS2+CS3+CS21	2023	0	0	0	0	1	0	0	0	0	0	1	1	0	0	1	0	0	0	0	1	0	0	0	0	0	0	0	0	0	0	0
GVE0402	Bangladesh 2022-2023 (This study)	L2	CS2+CS3+CS21	2023	0	0	0	0	1	0	0	0	0	0	0	1	0	0	1	0	0	0	0	1	0	0	0	0	0	0	0	1	0	0	0
GVE0403	Bangladesh 2022-2023 (This study)	L1	CS1+CS3+CS21	2023	0	0	0	0	1	0	0	0	0	0	0	1	0	0	1	0	0	0	0	1	0	0	0	1	0	0	0	0	0	0	0
GVE0404	Bangladesh 2022-2023 (This study)	L11	CFnegative	2023	0	0	0	0	0	0	0	0	0	0	0	1	0	0	0	0	0	0	0	1	0	0	0	1	0	0	0	0	0	0	0
GVE0405	Bangladesh 2022-2023 (This study)	L7	CS6	2023	0	0	0	0	0	0	0	0	0	0	0	1	0	0	1	1	0	0	0	1	0	0	0	0	0	0	0	0	0	0	0
GVE0407	Bangladesh 2022-2023 (This study)	L19	CS23	2023	0	0	0	0	1	0	0	0	0	0	0	1	1	0	1	0	0	0	0	0	0	0	0	1	0	0	0	0	0	0	0
GVE0425	Bangladesh 2022-2023 (This study)	L5	CS5+CS6	2023	0	0	0	0	0	0	0	0	0	0	0	1	0	0	0	1	0	0	0	0	0	0	0	0	0	0	0	0	0	0	0
GVE0426	Bangladesh 2022-2023 (This study)	L5	CS17	2023	0	0	0	0	0	0	0	0	1	0	0	0	0	0	0	0	0	0	0	1	0	0	0	0	1	0	0	0	0	0	0
GVE0427	Bangladesh 2022-2023 (This study)	L3	CFA/I+CS21	2023	0	0	0	0	1	0	0	0	0	0	1	1	0	0	1	0	0	0	0	0	0	0	0	1	0	0	0	0	0	0	0
GVE0429	Bangladesh 2022-2023 (This study)	L26	CS1+CS3+CS21	2023	0	0	0	0	1	0	0	0	0	0	0	0	0	0	1	0	0	0	0	1	0	0	0	1	0	0	0	0	0	0	0
GVE0433	Bangladesh 2022-2023 (This study)	L5	CS5+CS6	2023	0	0	0	0	0	0	0	0	0	0	0	1	0	0	0	1	0	0	0	0	0	0	0	0	0	0	0	0	0	0	0
GVE0434	Bangladesh 2022-2023 (This study)	L6	CFA/I+CS21	2023	0	0	0	0	0	0	0	0	0	0	0	1	0	0	1	0	0	0	0	1	0	0	0	0	0	0	0	0	0	0	0
GVE0436	Bangladesh 2022-2023 (This study)	L5	CFnegative	2023	0	0	0	0	0	0	0	0	0	0	0	1	0	0	1	0	0	0	0	0	0	0	0	0	0	0	0	0	0	0	0
GVE0437	Bangladesh 2022-2023 (This study)	L11	CS6	2023	0	0	0	0	0	0	0	0	0	0	1	1	0	0	0	1	0	0	0	0	0	0	0	0	1	0	0	0	0	0	0
GVE0441	Bangladesh 2012-2021 (This study)	L19	CS23	2021	0	0	0	0	1	0	0	1	0	0	0	1	1	0	0	0	0	0	0	0	0	0	0	1	0	0	0	0	0	0	0
GVE0443	Bangladesh 2012-2021 (This study)	L15	CFnegative	2021	0	0	0	0	1	0	0	0	0	0	0	1	0	0	1	0	0	0	0	0	0	0	0	0	0	0	0	0	0	0	0
GVE0445	Bangladesh 2012-2021 (This study)	L19	CS14	2021	0	0	0	0	0	0	0	0	0	0	0	1	0	0	0	0	0	0	0	0	0	0	0	1	0	0	0	0	0	0	0
GVE0446	Bangladesh 2012-2021 (This study)	L25	CS23	2021	1	0	0	0	1	0	0	1	1	1	0	1	1	0	0	0	0	0	0	0	0	0	0	1	0	0	1	1	0	0	0
GVE0448	Bangladesh 2012-2021 (This study)	L22	CFnegative	2021	0	0	0	0	0	0	0	0	0	0	0	1	0	0	1	0	0	0	0	0	0	0	0	1	0	0	0	0	0	0	0
GVE0450	Bangladesh 2012-2021 (This study)	L19	CFnegative	2021	0	0	0	0	0	0	0	0	0	0	0	1	0	0	0	0	0	0	0	0	0	0	0	1	0	0	0	0	0	1	0
GVE0451	Bangladesh 2012-2021 (This study)	L5	CS5+CS6	2021	0	0	0	0	0	0	0	0	0	0	0	1	1	0	0	1	0	0	0	0	0	0	0	0	0	0	0	0	0	0	0
GVE0452	Bangladesh 2012-2021 (This study)	L5	CS6	2021	0	0	0	0	0	0	0	0	0	0	0	1	0	0	1	0	0	0	0	0	0	0	0	0	0	0	0	0	0	0	0
GVE0453	Bangladesh 2012-2021 (This study)	L5	CS5+CS6	2020	0	0	0	0	0	0	0	0	0	0	0	1	0	0	0	0	0	0	0	0	0	0	0	0	0	0	0	0	0	0	0
GVE0454	Bangladesh 2012-2021 (This study)	L5	CFnegative	2020	0	0	0	0	0	1	0	0	0	0	0	1	0	0	1	0	0	0	0	0	0	0	0	0	0	0	0	0	0	0	0
GVE0455	Bangladesh 2012-2021 (This study)	L22	CS26	2021	0	0	0	0	0	0	0	0	0	0	1	1	0	0	0	0	0	0	0	0	0	0	0	0	0	0	0	0	0	0	0
GVE0456	Bangladesh 2012-2021 (This study)	Not defined	CS20	2021	0	0	0	0	0	0	0	0	0	0	0	1	0	0	0	0	0	0	0	0	0	0	0	1	0	0	0	0	0	0	0
GVE0466	Bangladesh 2012-2021 (This study)	L16	CFnegative	2020	0	0	0	0	0	0	0	0	0	0	0	1	1	0	0	0	0	0	0	0	0	0	0	1	0	0	0	0	0	0	0
GVE0476	Bangladesh 2012-2021 (This study)	L16	CFnegative	2018	0	0	0	0	0	0	0	0	1	0	0	1	1	0	0	0	0	0	0	0	0	0	0	1	1	0	0	1	0	1	0
GVE0477	Bangladesh 2012-2021 (This study)	L26	CFnegative	2018	0	0	0	0	0	0	0	0	0	0	0	1	1	0	0	0	0	0	0	0	0	0	0	1	0	0	0	0	0	0	0
GVE0480	Bangladesh 2012-2021 (This study)	L25	CFnegative	2018	1	0	0	0	0	0	0	0	0	0	0	1	1	0	0	0	0	0	0	0	0	0	0	1	0	0	0	0	0	0	0
GVE0481	Bangladesh 2012-2021 (This study)	L16	CFnegative	2018	0	0	0	0	0	0	0	0	0	0	0	1	1	0	0	0	0	0	0	0	0	0	0	1	0	0	0	0	0	0	0
GVE0482	Bangladesh 2012-2021 (This study)	L25	CFnegative	2018	1	0	0	0	0	0	0	0	0	0	0	1	1	0	0	0	0	0	0	0	0	0	0	1	0	0	0	0	0	0	0
GVE0483	Bangladesh 2012-2021 (This study)	L26	CFnegative	2018	0	1	0	0	1	0	0	0	1	0	0	1	1	0	0	0	0	0	0	0	0	0	0	1	0	0	0	0	0	0	0
GVE0484	Bangladesh 2012-2021 (This study)	L17	CFnegative	2018	0	0	0	0	1	0	0	0	1	1	0	1	1	0	0	0	0	0	0	0	0	0	0	1	0	0	0	0	0	0	0
GVE0485	Bangladesh 2012-2021 (This study)	L17	CS6	2018	0	0	0	0	1	0	0	0	0	0	0	1	0	0	1	0	0	0	0	0	0	0	0	0	0	0	0	0	0	0	0
GVE0487	Bangladesh 2012-2021 (This study)	L11	CFnegative	2018	0	0	0	0	0	0	0	0	1	0	0	1	0	0	0	0	0	1	0	0	0	0	0	0	0	0	0	0	0	0	0
GVE0488	Bangladesh 2012-2021 (This study)	L11	CFnegative	2018	0	0	0	0	0	0	0	0	0	0	0	1	0	0	0	0	0	0	0	0	0	1	0	0	0	0	0	0	0	0	0
GVE0489	Bangladesh 2012-2021 (This study)	L4	CS6+CS8	2018	1	0	0	0	0	0	0	0	0	0	0	1	1	0	1	0	0	0	0	0	0	0	0	0	0	0	0	0	0	1	0
GVE0491	Bangladesh 2012-2021 (This study)	Not defined	CS21	2018	0	0	0	0	0	0	0	0	0	0	0	1	1	0	1	0	0	0	0	0	0	0	0	1	0	0	0	0	0	0	0
GVE0492	Bangladesh 2012-2021 (This study)	L25	CS23	2021	0	0	0	0	1	0	0	0	0	0	0	1	0	0	0	0	0	0	0	0	0	0	0	1	0	1	0	1	0	0	0
GVE0493	Bangladesh 2012-2021 (This study)	L16	CS17	2018	0	0	0	0	0	0	0	0	0	0	1	1	0	0	0	0	0	0	0	0	0	0	0	0	0	0	0	0	0	0	0
GVE0494	Bangladesh 2012-2021 (This study)	Not defined	CS21	2018	0	0	0	0	0	0	0	0	0	0	0	1	0	0	1	0	0	0	0	0	0	0	0	0	0	0	0	0	0	0	0
GVE0495	Bangladesh 2012-2021 (This study)	L17	CFnegative	2018	0	0	0	0	1	0	0	0	0	0	0	1	1	0	0	0	0	0	0	0	0	0	0	1	0	0	0	0	0	0	0
GVE0498	Bangladesh 2012-2021 (This study)	L16	CS19	2019	0	0	0	0	0	0	0	0	0	0	0	1	0	0	1	0	0	0	0	0	0	0	0	0	0	0	0	0	0	0	0
GVE0499	Bangladesh 2012-2021 (This study)	L26	CFnegative	2019	0	0	0	0	1	0	0	0	0	0	0	1	1	0	0	0	0	0	0	0	0	0	0	1	0	0	0	0	0	0	0
GVE0500	Bangladesh 2012-2021 (This study)	L5	CS5+CS6	2019	0	0	0	0	0	0	0	0	0	0	0	1	0	0	0	0	0	0	0	0	0	0	0	0	0	0	1	0	0	0	0
GVE0501	Bangladesh 2012-2021 (This study)	L3	CS7	2019	0	0	0	0	1	0	0	0	0	0	0	1	0	0	0	0	0	0	0	0	0	0	0	1	0	0	0	0	0	0	0
GVE0502	Bangladesh 2022-2023 (This study)	L26	CS23	2023	0	0	0	0	1	1	0	0	1	0	0	1	1	0	0	1	0	0	0	1	1	0	0	1	1	0	0	0	0	0	0
GVE0503	Bangladesh 2022-2023 (This study)	Not defined	CS23	2023	0	0	0	0	0	0	0	1	0	1	0	1	1	0	0	0	0	0	0	0	0	0	0	1	0	0	0	0	0	0	0
GVE0504	Bangladesh 2022-2023 (This study)	Not defined	CS23	2023	0	0	0	0	0	0	0	1	0	1	0	1	1	0	0	0	0	0	0	0	0	0	0	1	0	0	0	0	0	0	0
GVE0505	Bangladesh 2012-2021 (This study)	L17	CS6	2019	0	0	0	0	1	0	0	0	0	0	0	1	0	0	1	0	0	0	0	0	0	0	0	0	0	0	0	0	0	0	0
GVE0506	Bangladesh 2012-2021 (This study)	L17	CS14	2019	0	0	0	0	0	0	0	0	0	0	0	1	0	0	1	0	0	0	0	0	0	0	0	0	0	0	0	0	0	0	0
GVE0507	Bangladesh 2012-2021 (This study)	L5	CS5+CS6	2019	0	0	0	0	0	0	0	0	0	0	0	1	0	0	0	0	0	0	0	0	0	0	0	0	0	0	0	0	0	0	0
GVE0508	Bangladesh 2012-2021 (This study)	L26	CFnegative	2019	0	0	0	0	0	0	0	0	0	0	0	1	1	0	0	0	0	0	0	0	0	0	0	1	0	0	0	0	0	0	0
GVE0509	Bangladesh 2012-2021 (This study)	L5	CS17	2019	0	0	0	0	0	1	0	0	0	0	0	1	0	0	0	1	0	0	0	0	0	0	0	0	0	0	0	0	0	0	0
GVE0543	Bangladesh 2012-2021 (This study)	L25	CFnegative	2017	0	0	0	0	1	0	0	0	0	0	0	1	1	0	0	0	0	0	0	0	0	0	0	1	0	0	0	1	0	0	1
GVE0546	Bangladesh 2012-2021 (This study)	L19	CS14	2016	0	0	0	0	0	0	0	0	0	0	1	1	0	0	1	0	0	0	0	0	0	0	0	0	0	0	0	0	1	0	0
GVE0549	Bangladesh 2012-2021 (This study)	L1	CS1+CS3+CS21	2016	0	0	0	0	1	0	0	0	0	0	0	1	0	0	1	0	0	0	0	1	0	0	0	1	0	0	0	0	0	0	0
GVE0551	Bangladesh 2012-2021 (This study)	L5	CS5+CS6	2017	0	0	0	0	0	0	0	0	0	0	0	1	0	0	0	0	0	0	0	0	0	0	0	0	0	0	0	0	0	0	0
GVE0556	Bangladesh 2012-2021 (This study)	L26	CS6	2016	0	0	0	0	1	1	0	0	0	0	0	1	0	0	1	0	0	0	0	0	0	0	0	0	0	0	0	0	0	0	0
GVE0562	Bangladesh 2022-2023 (This study)	L19	CS23	2023	0	0	0	0	0	0	0	0	0	0	0	1	1	0	0	0	0	0	0	0	0	0	0	1	0	0	1	1	0	0	0
GVE0569	Bangladesh 2012-2021 (This study)	L1	CS1+CS3+CS21	2014	0	0	0	0	1	0	0	0	0	0	0	1	0	0	1	0	0	0	0	1	0	0	0	1	0	0	0	0	0	0	0
GVE0588	Bangladesh 2012-2021 (This study)	L18	CS12	2014	0	0	0	0	0	0	0	0	0	0	0	1	0	0	0	0	0	0	0	0	0	0	0	0	0	0	0	0	0	0	0
GVE0627	Bangladesh 2022-2023 (This study)	L5	CFnegative	2023	0	0	0	0	0	0	0	1	0	0	0	1	1	0	0	0	0	0	0	0	0	0	0	0	0	0	0	0	0	0	0
GVE0628	Bangladesh 2022-2023 (This study)	Not defined	CFnegative	2023	0	0	0	0	0	0	0	0	0	0	0	0	0	0	0	0	1	0	0	0	0	0	0	0	0	1	0	0	0	0	0
GVE0631	Bangladesh 2012-2021 (This study)	L17	CS14	2014	0	0	0	0	0	0	0	0	1	0	0	1	0	0	1	0	0	0	0	0	0	0	0	1	0	0	0	0	0	0	0
GVE0632	Bangladesh 2012-2021 (This study)	L7	CS6	2014	0	0	0	0	0	0	0	0	0	0	1	1	0	0	1	0	0	0	0	0	0	0	0	0	0	0	0	0	0	1	0
GVE0633	Bangladesh 2012-2021 (This study)	L4	CS21	2014	0	0	0	0	1	0	0	0	0	0	0	1	1	0	1	0	0	0	0	0	0	0	0	1	0	0	1	0	0	0	0
GVE0634	Bangladesh 2012-2021 (This study)	L26	CFnegative	2014	0	0	0	0	0	0	0	0	0	0	0	1	1	0	0	0	0	0	0	0	0	0	0	1	0	0	1	0	0	0	0
GVE0635	Bangladesh 2012-2021 (This study)	L2	CS2+CS3+CS21	2014	0	0	0	0	1	0	0	0	0	0	0	1	0	0	1	0	0	0	0	1	0	0	0	0	0	0	0	0	0	0	0
GVE0636	Bangladesh 2022-2023 (This study)	L5	CFnegative	2023	0	0	0	0	0	0	0	0	0	1	0	1	1	0	0	0	0	0	0	0	0	0	0	1	0	0	1	0	0	0	0
GVE0637	Bangladesh 2022-2023 (This study)	L15	CS6	2023	0	0	0	1	1	0	0	1	1	0	0	1	0	0	1	0	0	0	0	1	0	0	0	0	0	0	0	0	0	0	0
GVE0638	Bangladesh 2012-2021 (This study)	L19	CFnegative	2017	0	0	0	0	0	0	0	0	0	0	0	1	1	0	0	0	0	0	0	0	0	0	0	1	0	0	0	0	0	0	1
GVE0639	Bangladesh 2022-2023 (This study)	L11	CS23	2023	0	0	0	0	1	0	0	1	0	1	0	1	1	0	0	0	0	0	0	0	0	0	0	1	0	0	0	0	0	0	0
GVE0640	Bangladesh 2022-2023 (This study)	Not defined	CS23	2023	0	0	0	0	0	0	0	0	1	0	0	1	1	0	0	0	0	0	0	0	0	0	0	1	0	0	0	0	0	0	0
GVE0641	Bangladesh 2012-2021 (This study)	L7	CS6	2014	0	0	0	0	0	0	0	0	0	0	1	1	0	0	1	0	0	0	0	0	0	0	0	0	0	0	0	0	0	1	0
GVE0642	Bangladesh 2012-2021 (This study)	L5	CFnegative	2017	0	0	0	0	0	0	0	1	0	0	0	1	1	0	0	0	0	0	0	0	0	0	0	0	0	0	0	0	0	0	0
GVE0643	Bangladesh 2022-2023 (This study)	L26	CFnegative	2023	0	0	0	0	0	0	0	0	0	0	0	1	0	0	1	0	0	0	0	0	0	0	0	0	0	0	0	0	0	0	0
GVE0644	Bangladesh 2022-2023 (This study)	L2	CS2+CS3+CS21	2023	0	0	0	0	1	0	0	0	0	0	0	1	0	0	1	0	0	0	0	1	0	0	0	0	0	0	0	0	0	0	0
GVE0645	Bangladesh 2012-2021 (This study)	L17	CFnegative	2014	0	0	0	0	1	0	0	0	0	0	0	1	0	0	1	0	0	0	0	0	0	0	0	0	0	0	0	0	0	0	0
GVE0646	Bangladesh 2012-2021 (This study)	L16	CFnegative	2015	0	0	0	0	0	0	0	0	0	0	0	1	1	0	1	0	0	0	0	0	0	0	0	1	0	0	0	0	0	0	0
GVE0647	Bangladesh 2022-2023 (This study)	L26	CS23	2023	0	0	0	0	1	0	0	0	0	0	0	1	1	0	0	0	0	0	0	0	0	0	0	1	0	0	0	1	0	0	0
GVE0648	Bangladesh 2022-2023 (This study)	L4	CS6+CS8	2023	1	0	0	0	0	0	0	1	0	0	1	1	0	0	0	0	0	0	0	0	0	0	0	0	0	0	0	0	0	0	0
GVE0649	Bangladesh 2012-2021 (This study)	Not defined	CS6	2015	1	1	0	0	1	0	0	1	0	0	1	1	1	0	0	0	0	0	0	1	0	0	0	1	0	0	1	0	0	0	0
GVE0650	Bangladesh 2012-2021 (This study)	L26	CFnegative	2014	0	0	0	0	0	0	0	0	0	0	0	1	1	0	0	0	0	0	0	0	1	0	0	1	0	0	0	0	0	0	0
GVE0651	Bangladesh 2012-2021 (This study)	L19	CFnegative	2014	1	0	0	0	0	0	0	1	0	0	0	1	1	0	0	0	0	0	0	0	0	0	0	1	0	0	0	0	0	0	0
GVE0653	Bangladesh 2022-2023 (This study)	L7	CS6	2023	0	0	0	0	0	0	0	0	0	0	0	1	0	0	1	0	0	0	0	0	0	0	0	0	0	0	0	0	0	0	0
GVE0654	Bangladesh 2022-2023 (This study)	Not defined	CS20	2023	0	0	0	0	1	1	0	0	0	0	0	1	0	1	0	0	0	0	0	0	0	0	0	0	0	0	0	0	0	0	0
GVE0655	Bangladesh 2022-2023 (This study)	L15	CFnegative	2023	0	0	0	0	0	0	0	0	0	0	0	1	0	0	0	0	0	0	0	0	0	0	0	0	0	0	0	0	0	1	0
GVE0656	Bangladesh 2022-2023 (This study)	L2	CS2+CS3+CS21	2023	0	0	0	0	0	0	0	0	0	0	0	1	0	0	1	0	0	0	0	1	0	0	0	0	0	0	0	0	0	0	0
GVE0657	Bangladesh 2012-2021 (This study)	L17	CS14	2015	0	0	0	0	0	0	0	0	1	0	0	1	0	0	1	0	0	0	0	0	0	0	0	1	0	0	0	0	0	0	0
GVE0658	Bangladesh 2012-2021 (This study)	Not defined	CS20	2015	0	0	0	0	0	0	0	0	0	0	1	1	0	0	0	0	0	0	0	0	0	0	0	0	1	0	0	0	0	0	0
GVE0659	Bangladesh 2012-2021 (This study)	L26	CFnegative	2015	0	0	0	0	0	0	0	0	0	1	0	1	1	0	0	0	0	0	0	0	0	0	0	1	0	0	1	0	0	0	0
GVE0660	Bangladesh 2012-2021 (This study)	L26	CFnegative	2015	0	0	0	0	0	0	0	1	0	1	0	1	1	0	0	0	0	0	0	0	0	0	0	1	0	0	1	0	0	0	0
GVE0661	Bangladesh 2012-2021 (This study)	L5	CFnegative	2015	0	0	0	0	0	0	0	0	0	0	0	1	0	0	1	0	0	0	0	0	0	0	0	0	0	0	0	0	0	0	0
GVE0662	Bangladesh 2012-2021 (This study)	L16	CFnegative	2015	0	0	0	0	0	0	0	0	0	0	0	1	1	0	0	0	0	0	0	0	0	0	0	1	0	0	1	0	0	0	0
GVE0663	Bangladesh 2022-2023 (This study)	L11	CS23	2023	0	0	0	0	0	0	0	0	0	0	0	1	1	0	1	0	0	0	0	0	0	0	0	1	0	0	1	0	0	0	0
GVE0664	Bangladesh 2012-2021 (This study)	L26	CFnegative	2014	0	0	0	0	0	0	0	0	0	0	0	1	1	0	0	0	0	0	0	0	0	0	0	1	0	0	1	0	0	0	0
GVE0665	Bangladesh 2022-2023 (This study)	L16	CS23	2023	0	0	0	0	0	0	0	0	0	0	0	1	0	0	0	0	0	0	0	0	0	0	0	1	0	0	1	0	0	0	0
GVE0666	Bangladesh 2012-2021 (This study)	Not defined	CS23	2020	0	0	0	0	0	0	0	0	1	0	0	1	1	0	1	0	0	0	0	0	0	0	0	1	0	0	0	0	0	0	0
GVE0667	Bangladesh 2012-2021 (This study)	L18	CS6	2015	0	0	0	0	0	0	0	0	0	0	1	1	0	0	1	0	0	0	0	1	0	0	0	0	0	0	0	0	0	0	0
GVE0668	Bangladesh 2012-2021 (This study)	L25	CFnegative	2014	0	0	0	0	0	0	0	0	0	1	0	1	1	0	0	0	0	0	0	0	0	0	0	1	0	0	1	0	0	1	0
GVE0669	Bangladesh 2012-2021 (This study)	L5	CFnegative	2014	0	0	0	0	0	0	0	0	0	0	0	1	0	0	1	0	0	0	0	0	0	0	0	0	0	0	0	0	0	0	0
GVE0670	Bangladesh 2012-2021 (This study)	L26	CFnegative	2014	0	0	0	0	0	0	0	0	0	0	0	1	1	0	0	0	0	0	0	0	0	0	0	1	0	0	1	0	0	0	0
GVE0696	Bangladesh 2012-2021 (This study)	L5	CS5+CS6	2016	0	0	0	0	0	0	0	0	0	0	0	1	0	0	0	0	0	0	0	0	0	0	0	0	0	0	0	0	0	0	0
GVE0697	Bangladesh 2012-2021 (This study)	L19	CS14	2016	0	0	0	0	0	0	0	0	0	0	1	1	0	0	1	0	0	0	0	0	0	0	0	0	0	0	0	0	0	0	0
GVE0698	Bangladesh 2012-2021 (This study)	L5	CS17	2017	0	0	0	0	0	0	0	0	1	0	0	1	0	0	0	0	0	0	0	1	0	0	0	0	0	0	0	0	0	0	0
GVE0699	Bangladesh 2022-2023 (This study)	Not defined	CS23	2023	0	0	0	0	0	0	0	0	1	0	0	1	1	0	0	0	0	0	0	0	0	0	0	1	0	0	0	0	0	0	0
GVE0700	Bangladesh 2022-2023 (This study)	Not defined	CS23	2023	0	0	0	0	0	0	0	0	1	0	0	1	0	0	0	0	0	0	0	0	0	0	0	1	0	0	0	0	0	0	0
GVE0701	Bangladesh 2022-2023 (This study)	L16	CS23	2023	1	0	0	0	0	0	0	1	0	0	0	1	1	0	0	0	0	0	0	0	0	0	0	1	0	0	0	0	0	0	0
GVE0702	Bangladesh 2012-2021 (This study)	L5	CS5+CS6	2017	0	0	0	0	0	0	0	0	0	0	0	1	0	0	0	0	0	0	0	0	0	0	0	0	0	0	1	0	0	0	0
GVE0703	Bangladesh 2012-2021 (This study)	L16	CFnegative	2016	1	0	0	0	1	0	0	1	0	1	0	1	1	0	1	0	0	0	0	0	0	0	0	1	0	0	0	0	0	0	0
GVE0704	Bangladesh 2012-2021 (This study)	L16	CFnegative	2020	0	0	0	0	0	0	0	0	1	0	0	1	1	0	0	0	0	0	0	0	0	0	0	1	1	0	0	0	0	0	0
GVE0705	Bangladesh 2012-2021 (This study)	L5	CS5+CS6	2016	0	0	0	0	0	0	0	0	1	0	0	1	0	0	0	0	0	0	0	0	0	0	0	0	0	0	0	0	0	0	0
GVE0706	Bangladesh 2012-2021 (This study)	L16	CS17	2017	0	0	0	0	0	0	0	0	0	0	1	1	0	0	0	0	0	0	0	0	0	0	0	0	0	0	0	0	0	0	0
GVE0707	Bangladesh 2022-2023 (This study)	L26	CS23	2023	0	0	0	0	1	0	0	1	0	1	0	1	1	0	0	0	0	0	0	0	0	0	0	0	0	0	1	1	0	0	0
GVE0708	Bangladesh 2012-2021 (This study)	L1	CS1+CS3+CS21	2017	0	0	0	0	1	0	0	0	0	0	0	1	0	0	1	0	0	0	0	0	0	0	0	1	0	0	0	0	0	0	0
GVE0709	Bangladesh 2022-2023 (This study)	L26	CS23	2023	0	0	0	0	1	0	0	0	0	0	0	1	1	0	1	0	0	0	0	0	0	0	0	1	0	0	0	0	0	0	0
GVE0710	Bangladesh 2012-2021 (This study)	L3	CS7	2017	0	0	0	0	1	0	0	0	0	0	0	1	0	0	0	0	0	0	0	0	0	0	0	1	0	0	0	0	0	0	0
GVE0711	Bangladesh 2012-2021 (This study)	L17	CS14	2013	0	0	0	0	0	0	0	0	1	0	0	1	0	0	1	0	0	0	0	0	0	0	0	1	0	0	0	0	0	0	0
GVE0712	Bangladesh 2012-2021 (This study)	Not defined	CS2+CS3+CS21	2016	0	0	0	0	1	0	0	0	0	0	0	1	0	0	1	0	0	0	0	0	0	0	0	0	0	0	0	0	0	0	0
GVE0713	Bangladesh 2012-2021 (This study)	L18	CS12	2016	0	0	0	0	0	0	0	0	0	0	0	1	0	0	0	0	0	0	0	0	0	0	0	0	0	0	0	0	0	0	0
GVE0714	Bangladesh 2012-2021 (This study)	L5	CFnegative	2016	0	0	0	0	0	0	0	0	0	0	0	1	0	0	1	0	0	0	0	0	0	0	0	0	0	0	0	0	0	0	0
GVE0715	Bangladesh 2012-2021 (This study)	L16	CS17	2016	0	0	0	0	0	0	0	0	0	0	1	1	0	0	0	0	0	0	0	0	0	0	0	0	0	0	0	0	0	0	0
GVE0716	Bangladesh 2012-2021 (This study)	L18	CS12	2016	0	0	0	0	0	0	0	0	0	0	0	1	0	0	0	0	0	0	0	0	0	0	0	0	0	0	0	0	0	0	0
GVE0717	Bangladesh 2022-2023 (This study)	L26	CS23	2023	0	0	0	0	0	0	0	0	0	0	0	1	0	0	0	0	0	0	0	0	0	0	0	1	0	0	0	1	0	0	0
GVE0718	Bangladesh 2012-2021 (This study)	L1	CS1+CS3+CS21	2013	0	0	0	0	1	0	0	0	0	0	1	1	0	0	1	0	0	0	0	1	0	0	0	1	0	0	0	0	0	0	0
GVE0719	Bangladesh 2012-2021 (This study)	Not defined	CS6	2013	0	0	0	0	0	0	0	1	0	0	1	1	0	0	0	0	0	0	0	1	0	0	0	0	0	0	0	0	0	0	0
GVE0720	Bangladesh 2022-2023 (This study)	L5	CS23	2023	0	0	0	0	0	0	0	0	0	0	0	1	1	0	0	0	0	0	0	0	0	0	0	0	0	0	0	0	0	0	0
GVE0721	Bangladesh 2012-2021 (This study)	L25	CFnegative	2013	1	0	0	0	1	0	0	1	0	1	0	1	1	0	0	0	0	0	0	0	0	0	0	1	0	0	1	0	0	0	0
GVE0722	Bangladesh 2012-2021 (This study)	L4	CS6+CS8	2013	0	0	0	0	0	0	0	0	0	0	0	1	0	0	0	0	0	0	0	0	0	0	0	0	0	0	0	0	0	0	0
GVE0723	Bangladesh 2012-2021 (This study)	L26	CFnegative	2013	1	0	0	0	1	0	0	1	1	0	0	1	1	0	0	0	0	0	0	0	0	0	0	1	1	1	0	1	0	0	0
GVE0724	Bangladesh 2012-2021 (This study)	L11	CFnegative	2015	0	0	0	0	0	0	0	1	0	0	0	1	0	0	0	0	0	0	0	0	0	0	0	1	0	0	0	0	0	0	0
GVE0725	Bangladesh 2012-2021 (This study)	L5	CS5+CS6	2013	0	0	0	0	0	0	0	0	0	0	0	1	0	0	0	0	0	0	0	0	0	0	0	0	0	0	0	0	0	0	0
GVE0726	Bangladesh 2012-2021 (This study)	L25	CFnegative	2013	1	0	0	0	0	0	0	0	0	1	0	1	1	0	0	0	0	0	0	0	0	0	0	1	0	0	1	1	0	1	0
GVE0727	Bangladesh 2022-2023 (This study)	L11	CS23	2023	0	0	0	0	1	0	0	0	1	0	0	1	1	0	1	0	0	0	0	0	0	0	0	0	0	0	0	1	0	0	0
GVE0728	Bangladesh 2012-2021 (This study)	L3	CS7	2013	0	1	0	0	1	0	0	0	0	0	0	1	0	0	0	0	0	0	0	0	0	0	0	1	0	0	0	0	0	0	0
GVE0729	Bangladesh 2012-2021 (This study)	L18	CS6	2013	0	0	0	0	0	0	0	0	0	0	1	1	0	0	1	0	0	0	0	0	0	0	0	0	0	0	0	0	0	0	0
GVE0730	Bangladesh 2012-2021 (This study)	L26	CFnegative	2013	0	0	0	0	1	0	0	0	1	0	0	1	1	0	0	0	0	0	0	0	0	0	0	1	0	1	1	0	0	0	0
GVE0731	Bangladesh 2022-2023 (This study)	L26	CS23	2023	0	0	0	0	1	0	0	0	0	0	0	1	1	0	0	0	0	0	0	0	0	0	0	0	0	0	1	1	0	0	0
GVE0736	Bangladesh 2012-2021 (This study)	L5	CS5+CS6	2017	0	0	0	0	0	0	0	0	0	0	0	1	0	0	0	0	0	0	0	0	0	0	0	0	0	0	0	1	0	0	0
GVE0737	Bangladesh 2012-2021 (This study)	L7	CS6	2017	0	0	0	0	0	0	0	0	0	0	0	1	0	0	1	0	0	0	0	0	0	0	0	0	0	0	1	0	0	0	0
GVE0738	Bangladesh 2012-2021 (This study)	L19	CFnegative	2019	1	0	0	0	1	0	0	1	0	0	0	1	1	0	0	0	0	0	0	0	0	0	0	1	0	0	0	0	0	0	0
GVE0739	Bangladesh 2012-2021 (This study)	L1	CS1+CS3+CS21	2019	0	0	0	0	1	0	0	0	0	0	0	1	0	0	1	0	0	0	0	0	0	0	0	1	0	0	0	0	0	0	0
GVE0740	Bangladesh 2012-2021 (This study)	L26	CS23	2020	0	0	0	0	0	0	0	0	0	1	0	1	1	0	0	0	0	0	0	0	0	0	0	1	0	0	1	1	0	0	0
GVE0741	Bangladesh 2012-2021 (This study)	L25	CS23	2020	0	0	0	0	1	0	0	0	0	1	0	1	1	0	0	0	0	0	0	0	0	0	0	1	0	0	0	1	0	0	1
GVE0742	Bangladesh 2012-2021 (This study)	Not defined	CFnegative	2020	0	0	0	0	0	0	0	0	0	0	0	1	0	0	1	0	0	0	0	0	0	0	0	0	0	0	0	0	0	0	0
GVE0743	Bangladesh 2012-2021 (This study)	L26	CS23	2020	0	0	0	0	0	0	0	0	0	0	0	1	1	0	0	0	0	0	0	0	0	0	0	0	0	0	0	0	0	0	0
GVE0744	Bangladesh 2012-2021 (This study)	L7	CS6	2021	0	0	0	0	0	0	0	0	0	0	0	1	0	0	1	0	0	0	0	1	0	0	0	0	0	0	0	0	0	0	0
GVE0745	Bangladesh 2012-2021 (This study)	L5	CS23	2021	0	0	0	0	1	0	0	1	0	0	0	1	1	0	0	0	0	0	0	0	0	0	0	1	0	0	1	0	0	0	0
GVE0746	Bangladesh 2012-2021 (This study)	L19	CS14	2020	0	0	0	0	0	0	0	0	0	0	0	1	0	0	0	0	0	0	0	0	0	0	0	0	0	0	0	0	0	0	0
GVE0747	Bangladesh 2022-2023 (This study)	L5	CS23	2023	0	0	0	0	0	0	0	0	0	0	0	1	0	0	0	0	0	0	0	0	0	0	0	1	0	0	1	1	0	0	0
GVE0748	Bangladesh 2012-2021 (This study)	L26	CS23	2020	0	0	0	0	0	0	0	0	0	0	0	1	1	0	0	0	0	0	0	0	0	0	0	0	0	0	1	0	0	0	0
GVE0749	Bangladesh 2012-2021 (This study)	L19	CS23	2021	0	0	0	0	0	0	0	0	0	0	0	1	0	0	0	0	0	0	0	0	0	0	0	1	0	0	0	1	0	0	0
GVE0751	Bangladesh 2012-2021 (This study)	L25	CFnegative	2016	0	0	0	0	1	0	0	0	0	1	0	1	1	0	0	0	0	0	0	0	0	0	0	1	0	0	1	1	0	0	0
GVE0752	Bangladesh 2012-2021 (This study)	L16	CFnegative	2015	0	0	0	0	0	0	0	0	0	0	0	1	1	0	0	0	0	0	0	0	0	0	0	1	0	0	0	0	0	1	0
GVE0753	Bangladesh 2012-2021 (This study)	L5	CS17	2020	0	0	0	0	1	1	0	0	0	0	0	1	0	0	0	0	0	0	0	0	0	0	0	0	0	0	0	0	0	0	0
GVE0754	Bangladesh 2012-2021 (This study)	L16	CFnegative	2015	0	0	0	0	1	0	0	0	0	0	0	1	1	0	0	0	0	0	0	0	0	0	0	1	0	0	0	0	0	1	0
GVE0757	Bangladesh 2012-2021 (This study)	L5	CFnegative	2019	0	0	0	0	0	0	0	0	0	0	0	0	1	1	1	0	0	0	0	1	0	0	0	0	1	0	0	0	0	1	0
GVE0758	Bangladesh 2012-2021 (This study)	L17	CS14	2019	0	0	0	0	0	0	0	0	0	0	0	1	0	0	1	0	0	0	0	0	0	0	0	1	0	0	0	0	0	0	0
GVE0759	Bangladesh 2012-2021 (This study)	L5	CS5+CS6	2019	0	0	0	0	0	0	0	0	0	0	0	1	0	0	0	0	0	0	0	0	0	0	0	0	0	0	1	0	0	0	0
GVE0760	Bangladesh 2012-2021 (This study)	L1	CS1+CS3+CS21	2017	0	0	0	0	1	0	0	0	0	0	0	1	0	0	1	0	0	0	0	0	0	0	0	1	0	0	0	0	0	0	0
GVE0761	Bangladesh 2012-2021 (This study)	L26	CS23	2021	0	0	0	0	1	0	0	0	0	1	0	1	1	0	0	0	0	0	0	0	0	0	0	1	0	0	1	1	0	0	0
GVE0762	Bangladesh 2012-2021 (This study)	L26	CFnegative	2015	0	0	0	0	1	0	0	0	1	0	0	1	1	0	0	0	0	0	0	0	0	0	0	1	0	0	0	0	0	0	0
GVE0763	Bangladesh 2012-2021 (This study)	Not defined	CS23	2020	0	0	0	0	0	0	0	0	1	0	0	1	1	0	0	0	0	0	0	0	0	0	0	1	0	0	0	0	0	0	0
GVE0764	Bangladesh 2012-2021 (This study)	Not defined	CS23	2020	0	0	0	0	0	0	0	1	1	1	0	1	1	0	0	0	0	0	0	0	0	0	0	1	0	0	0	0	0	0	0
GVE0770	Bangladesh 2012-2021 (This study)	L25	CFnegative	2012	0	0	0	0	1	0	0	0	0	1	0	1	1	0	1	0	0	0	0	0	0	0	0	1	0	0	1	0	0	1	0
GVE0771	Bangladesh 2012-2021 (This study)	Not defined	CS12	2012	0	0	0	0	0	0	0	0	1	0	0	1	0	0	0	0	0	0	0	0	0	0	0	0	1	0	0	0	0	0	0
GVE0772	Bangladesh 2012-2021 (This study)	L25	CFnegative	2012	1	0	0	0	0	0	0	0	0	1	0	1	1	0	0	0	0	0	0	0	0	0	0	1	0	0	1	1	0	0	0
GVE0773	Bangladesh 2012-2021 (This study)	L5	CS5+CS6	2012	0	0	0	0	0	0	0	0	1	0	0	1	0	0	0	0	0	0	0	0	0	0	0	0	0	0	0	0	0	0	0
GVE0774	Bangladesh 2012-2021 (This study)	L1	CS1+CS3+CS21	2012	0	0	0	0	1	0	0	0	1	0	0	1	0	0	1	0	0	0	0	1	0	0	0	1	0	0	0	0	0	0	0
GVE0775	Bangladesh 2012-2021 (This study)	L16	CS17	2012	0	0	0	0	0	0	0	0	0	0	1	1	0	0	0	0	0	0	0	0	0	0	0	0	0	0	0	0	0	0	0
GVE0776	Bangladesh 2022-2023 (This study)	L11	CS23	2023	0	0	0	0	0	0	0	0	0	0	0	1	1	0	0	0	0	0	0	0	0	0	0	1	0	0	1	1	0	0	0
GVE0777	Bangladesh 2012-2021 (This study)	L5	CFnegative	2012	0	0	0	0	0	0	0	0	0	0	0	1	0	0	1	0	0	0	0	0	0	0	0	0	1	0	0	0	0	0	0
GVE0778	Bangladesh 2022-2023 (This study)	L11	CS23	2023	0	0	0	0	1	0	0	1	0	0	0	1	1	0	0	0	0	0	0	0	0	0	0	1	0	0	0	0	0	0	0
GVE0779	Bangladesh 2012-2021 (This study)	L1	CS1+CS3+CS21	2012	0	0	0	0	1	0	0	0	0	0	0	1	0	0	1	0	0	0	0	1	0	0	0	1	0	0	0	0	0	0	0
GVE0780	Bangladesh 2012-2021 (This study)	Not defined	CS12	2012	0	0	0	0	0	0	0	0	0	0	0	1	0	0	0	0	0	0	0	0	0	0	0	1	0	0	0	0	0	1	0
GVE0781	Bangladesh 2012-2021 (This study)	L26	CFnegative	2012	0	0	0	0	0	0	0	0	0	0	0	1	1	0	1	0	0	0	0	0	1	0	0	1	0	1	0	0	0	0	0
GVE0782	Bangladesh 2012-2021 (This study)	Not defined	CFnegative	2012	0	0	0	0	1	0	0	0	0	0	0	1	0	0	0	0	0	0	0	1	0	0	0	1	0	0	0	0	0	0	0
GVE0783	Bangladesh 2012-2021 (This study)	L3	CS7	2012	1	0	0	0	1	0	0	0	0	0	0	1	0	0	0	0	0	0	0	0	0	0	0	1	0	0	0	0	0	0	0
GVE0784	Bangladesh 2012-2021 (This study)	L25	CFnegative	2012	0	1	1	0	0	0	0	0	0	1	0	1	1	0	0	0	0	0	0	0	0	0	0	1	0	0	1	0	0	0	0
