# Supplementary material for: Genomic insights into a diarrheal outbreak in Bangladesh reveal novel ETEC lineages and expansion of CS23 colonization factor
Source: Microbiol Spectr. 2025 Sep 10;13(10):e03315-24. doi: 10.1128/spectrum.03315-24 (PMC12502627; doi:10.1128/spectrum.03315-24)
Supplement: Table S3 — Data set of AMR genes for a total of 836 ETEC isolates, including 475 Bangladeshi ETEC isolates of this study. [file spectrum.03315-24-s0005.pdf]

Table S3: Dataset of AMR genes for total 836 ETEC isolates including 475 Bangladeshi ETEC isolates of this study

StrainID	Study Dataset	ETEC_Lineage	CFs	Year	ampicillin.TEM	macrolide.mrx	macrolide.mphA.E.G.	macrolide.msrE	ESBL.CTXM	ESBL.DHA1	ESBL.Carbapenum	Fluoroquinolone.QnrB	Fluoroquinolone.QnrS	Fluoroquinolone.gyrB	Fluoroquiolone.parC	strA.APH6	strB.APH3	Trimethoprim.dfr	sulphonamide.sul1	sulphonamide.sul2	sulphonamide.sul3	chloramphenicol.CAT	tetracyclin.tetA	tetracyclin.tetB
E1003	Global 1983-2008 (Mentzer et al)	L5	CS5+CS6	1999	1	0	0	0	0	0	0	0	0	0	0	1	0	0	0	1	0	0	0	0
E1009	Global 1983-2008 (Mentzer et al)	L3	CS7	1999	1	0	0	0	0	0	0	0	0	0	0	1	0	1	0	1	0	0	0	0
E1018	Global 1983-2008 (Mentzer et al)	L2	CS2+CS3+CS21	1999	0	0	0	0	0	0	0	0	0	0	0	0	0	0	0	0	0	0	0	1
E1034	Global 1983-2008 (Mentzer et al)	L5	CS5+CS6	1999	0	0	0	0	0	0	0	0	0	0	0	0	0	0	0	0	0	0	0	0
E1057	Global 1983-2008 (Mentzer et al)	L11+L15	CS27A	2000	0	0	0	0	0	0	0	0	0	0	0	0	0	0	0	0	0	0	0	0
E106	Global 1983-2008 (Mentzer et al)	NA	CS6	1985	0	0	0	0	0	0	0	0	0	0	0	0	0	0	0	0	0	0	0	0
E1072	Global 1983-2008 (Mentzer et al)	NA	CS20	2000	1	0	0	0	0	0	0	0	0	0	0	0	0	0	0	0	0	0	0	0
E1074	Global 1983-2008 (Mentzer et al)	L1	CS1+CS3+CS21	2000	0	0	0	0	0	0	0	0	0	0	0	0	0	0	0	0	0	0	0	0
E1085	Global 1983-2008 (Mentzer et al)	L5	CS5+CS6	2000	0	0	0	0	0	0	0	0	0	0	0	1	0	1	0	1	0	0	1	0
E1091	Global 1983-2008 (Mentzer et al)	L11+L15	CFA/I+CS21	2000	1	0	0	0	0	0	0	0	0	0	0	1	0	1	0	1	0	0	0	0
E110	Bangladesh 1980-2011 (Mentzer et al)	L5	CS5+CS6	1985	0	0	0	0	0	0	0	0	0	0	0	1	0	0	0	1	0	0	0	0
E1101	Global 1983-2008 (Mentzer et al)	L11+L15	CS30	2000	1	0	0	0	0	0	0	0	0	0	0	1	0	1	0	1	0	0	0	1
E1102	Global 1983-2008 (Mentzer et al)	NA	CFnegative	2000	1	0	0	0	0	0	0	0	0	0	0	1	0	1	1	1	0	0	1	0
E1111	Global 1983-2008 (Mentzer et al)	L5	CS5+CS6	2000	0	0	0	0	0	0	0	0	0	0	0	0	0	1	0	0	0	0	0	0
E1114	Global 1983-2008 (Mentzer et al)	L4	CS6+CS8	2001	1	0	0	0	1	0	0	0	0	0	0	1	0	1	0	1	0	0	1	0
E1115	Global 1983-2008 (Mentzer et al)	L4	CS6+CS8	2001	1	0	0	0	0	0	0	0	0	0	0	1	0	1	0	1	0	0	0	0
E1116	Global 1983-2008 (Mentzer et al)	L4	CS6+CS8	2001	1	0	0	0	0	0	0	0	0	0	0	1	0	1	0	1	0	0	0	0
E1117	Global 1983-2008 (Mentzer et al)	L4	CS6+CS8	2001	1	0	0	0	0	0	0	0	0	0	0	1	0	1	0	1	0	0	0	0
E1118	Global 1983-2008 (Mentzer et al)	L4	CS6+CS8	2001	1	0	0	0	0	0	0	0	0	0	0	1	0	1	0	1	0	0	0	0
E1119	Global 1983-2008 (Mentzer et al)	L4	CS6+CS21	2001	0	0	0	0	0	0	0	0	0	0	0	0	0	1	1	0	0	0	1	0
E1120	Global 1983-2008 (Mentzer et al)	L4	CS6+CS21	2001	0	0	0	0	0	0	0	0	0	0	0	0	0	1	1	0	0	0	1	0
E1121	Global 1983-2008 (Mentzer et al)	L4	CS6+CS21	2001	0	0	0	0	0	0	0	0	0	0	0	0	0	1	1	0	0	0	1	0
E1122	Global 1983-2008 (Mentzer et al)	L4	CS6+CS21	2001	0	0	0	0	0	0	0	0	0	0	0	0	0	1	1	0	0	0	1	0
E1167	Global 1983-2008 (Mentzer et al)	L9+L10+L21	CS19	2001	0	0	0	0	0	0	0	0	0	0	0	0	0	0	0	0	0	0	0	0
E1169	Global 1983-2008 (Mentzer et al)	NA	CS20	2001	1	0	0	0	0	0	0	0	0	0	0	0	0	0	0	0	0	0	0	0
E1189	Global 1983-2008 (Mentzer et al)	L8	CS6+CS21	2001	1	0	0	0	0	0	0	0	0	0	0	1	0	1	0	1	0	0	0	0
E1193	Global 1983-2008 (Mentzer et al)	L12+L14	CFnegative	2001	0	0	0	0	0	0	0	0	0	0	0	0	0	0	0	0	0	0	0	0
E1242	Global 1983-2008 (Mentzer et al)	NA	CS18	2001	0	0	0	0	0	0	0	0	0	0	0	1	0	0	0	1	0	0	0	0
E1245	Global 1983-2008 (Mentzer et al)	L7	CS6	2001	0	0	0	0	0	0	0	0	0	0	0	0	0	0	0	0	0	0	0	1
E1248	Global 1983-2008 (Mentzer et al)	L9+L10+L21	CS19	2001	1	0	0	0	0	0	0	0	0	0	0	1	0	1	0	1	0	0	0	0
E1258	Global 1983-2008 (Mentzer et al)	L9+L10+L21	CS19	2001	0	0	0	0	0	0	0	0	0	0	0	1	0	0	0	1	0	0	0	0
E126	Global 1983-2008 (Mentzer et al)	L19	CS14	1989	0	0	0	0	0	0	0	0	0	0	0	0	0	0	0	0	0	0	0	0
E1264	Global 1983-2008 (Mentzer et al)	L9+L10+L21	CS19	2001	1	0	0	0	0	0	0	0	0	0	0	1	0	1	1	1	0	0	0	0
E1281	Global 1983-2008 (Mentzer et al)	L9+L10+L21	CS19	2001	0	0	0	0	0	0	0	0	0	0	0	1	0	0	0	1	0	0	0	0
E1282	Global 1983-2008 (Mentzer et al)	L24	CFnegative	2001	1	0	0	0	0	0	0	0	0	0	0	1	0	1	0	1	0	0	0	1
E1285	Global 1983-2008 (Mentzer et al)	L9+L10+L21	CS19	2001	0	0	0	0	0	0	0	0	0	0	0	1	0	0	0	1	0	0	0	0
E1287	Global 1983-2008 (Mentzer et al)	L11+L15	CFA/I+CS21	2001	1	1	1	0	0	0	0	0	0	0	0	1	0	1	0	1	0	0	0	1
E129	Global 1983-2008 (Mentzer et al)	L17	CS17	1989	0	0	0	0	0	0	0	0	0	0	0	1	0	0	0	0	0	0	0	0
E1298	Global 1983-2008 (Mentzer et al)	L5	CS5+CS6	2001	0	0	0	0	0	0	0	0	0	0	0	1	0	1	0	1	0	0	0	0
E1316	Global 1983-2008 (Mentzer et al)	L1	CS1+CS3+CS21	1996	0	0	0	0	0	0	0	0	0	0	0	0	0	0	0	0	0	0	0	0
E1328	Global 1983-2008 (Mentzer et al)	L7	CS6	1996	1	0	0	0	0	0	0	0	0	0	0	1	0	1	0	1	0	0	0	1
E1329	Global 1983-2008 (Mentzer et al)	L7	CS6	1996	1	0	0	0	0	0	0	0	0	0	0	1	0	1	0	1	0	0	0	1
E133	Global 1983-2008 (Mentzer et al)	L11+L15	CS12	1989	0	0	0	0	0	0	0	0	0	0	0	0	0	0	0	0	0	0	0	0
E1334	Global 1983-2008 (Mentzer et al)	L7	CS6	1996	0	0	0	0	0	0	0	0	0	0	0	0	0	0	0	0	0	0	0	1
E135	Global 1983-2008 (Mentzer et al)	L4	CS6+CS8	1989	0	0	0	0	0	0	0	0	0	0	0	0	0	0	0	0	0	0	0	0
E1352	Global 1983-2008 (Mentzer et al)	L8	CS6+CS21	1996	0	0	0	0	0	0	0	0	0	0	0	0	0	0	0	0	0	0	0	0
E1355	Global 1983-2008 (Mentzer et al)	L8	CS6+CS21	1996	0	0	0	0	0	0	0	0	0	0	0	0	0	0	0	0	0	0	0	0
E1356	Global 1983-2008 (Mentzer et al)	L8	CS6+CS21	1996	0	0	0	0	0	0	0	0	0	0	0	0	0	0	0	0	0	0	0	0
E1360sec	Global 1983-2008 (Mentzer et al)	L19	F41a	1996	0	0	0	0	0	0	0	0	0	0	0	0	0	0	0	0	0	0	0	0
E1362	Global 1983-2008 (Mentzer et al)	L4	CS21	1996	0	0	0	0	0	0	0	0	0	0	0	0	0	0	0	0	0	0	0	0
E1363	Global 1983-2008 (Mentzer et al)	L4	CS6+CS21	1996	0	0	0	0	0	0	0	0	0	0	0	0	0	0	0	0	0	0	0	1
E1365	Global 1983-2008 (Mentzer et al)	L4	CS21	1996	0	0	0	0	0	0	0	0	0	0	0	0	0	0	0	0	0	0	0	0
E1373	Global 1983-2008 (Mentzer et al)	L7	CS6	1996	0	0	0	0	0	0	0	0	0	0	0	0	0	0	0	0	0	0	0	1
E1392	Global 1983-2008 (Mentzer et al)	L17	CS6	1996	0	0	0	0	0	0	0	0	0	0	0	0	0	0	0	0	0	0	0	0
E1398	Global 1983-2008 (Mentzer et al)	L17	CS6	1996	0	0	0	0	0	0	0	0	0	0	0	1	0	0	0	1	0	0	0	0
E1407	Global 1983-2008 (Mentzer et al)	L20	CS6	1996	0	0	0	0	0	0	0	0	0	0	0	0	0	1	0	0	0	0	0	0
E141	Global 1983-2008 (Mentzer et al)	L3	CS7	1989	0	0	0	0	0	0	0	0	0	0	0	0	0	0	0	0	0	0	0	0
E1429tiny	Global 1983-2008 (Mentzer et al)	L4	CS6+CS21	1997	0	0	0	0	0	0	0	0	0	0	0	0	0	0	0	0	0	0	0	0
E143	Global 1983-2008 (Mentzer et al)	L2	CS2+CS3+CS21	1983	0	0	0	0	0	0	0	0	0	0	0	0	0	0	0	0	0	0	0	0
E1432G	Global 1983-2008 (Mentzer et al)	L11+L15	CFnegative	1997	1	0	0	0	0	0	0	0	0	0	0	1	0	1	0	1	0	0	0	1
E1432w	Global 1983-2008 (Mentzer et al)	L28	CFnegative	1997	0	0	0	0	0	0	0	0	0	0	0	0	0	0	0	0	0	0	0	0
E1433	Global 1983-2008 (Mentzer et al)	L1	CS1+CS3+CS21	1997	0	0	0	0	0	0	0	0	0	0	0	0	0	0	0	0	0	0	0	0
E1441	Global 1983-2008 (Mentzer et al)	L4	CS6+CS21	1997	0	0	0	0	0	0	0	0	0	0	0	0	0	1	1	0	0	0	1	0
E1442	Global 1983-2008 (Mentzer et al)	L4	CS6+CS21	1997	0	0	0	0	0	0	0	0	0	0	0	0	0	1	1	0	0	0	1	0
E1460	Global 1983-2008 (Mentzer et al)	NA	CFnegative	1997	0	0	0	0	0	0	0	0	0	0	0	1	0	0	0	0	0	0	0	1
E1484	Global 1983-2008 (Mentzer et al)	L7	CS6	2003	0	0	0	0	0	0	0	0	0	0	0	0	0	0	0	0	0	0	0	1
E151	Global 1983-2008 (Mentzer et al)	NA	CS12	1983	1	0	0	0	0	0	0	0	0	0	0	0	0	0	0	0	0	0	0	0
E1512	Global 1983-2008 (Mentzer et al)	L17	CS6	2003	0	0	0	0	0	0	0	0	0	0	0	0	0	0	0	0	0	0	0	0
E1516	Global 1983-2008 (Mentzer et al)	L4	CS6	2003	0	0	0	0	0	0	0	0	0	0	0	0	0	1	1	0	0	0	1	0
E1523	Global 1983-2008 (Mentzer et al)	L17	CS6	2003	0	0	0	0	0	0	0	0	0	0	0	0	0	0	0	0	0	0	0	0
E1524	Global 1983-2008 (Mentzer et al)	L16	CS5+CS6+CS14	1989	0	0	0	0	0	0	0	0	0	0	0	0	0	0	0	0	0	0	0	0
E1525	Global 1983-2008 (Mentzer et al)	L11+L13	CS27B	1989	0	0	0	0	0	0	0	0	0	0	0	1	0	0	0	1	0	0	0	0
E1526	Global 1983-2008 (Mentzer et al)	L16	CFnegative	1989	1	0	0	0	0	0	0	0	0	0	0	0	0	0	1	0	0	1	0	0
E1527	Global 1983-2008 (Mentzer et al)	L17	CS5+CS6	1989	0	0	0	0	0	0	0	0	0	0	0	0	0	0	0	0	0	0	0	0
E1532	Global 1983-2008 (Mentzer et al)	L11+L15	CFnegative	1989	0	0	0	0	0	0	0	0	0	0	0	0	0	0	0	0	0	0	0	0
E1533	Global 1983-2008 (Mentzer et al)	NA	CS30	1989	0	0	0	0	0	0	0	0	0	0	0	0	0	0	0	0	0	0	0	0
E1534	Global 1983-2008 (Mentzer et al)	L23	CFnegative	1989	0	0	0	0	0	0	0	0	0	0	0	0	0	0	0	0	0	0	0	0
E1535	Global 1983-2008 (Mentzer et al)	L1	CS1+CS3+CS21	1989	0	0	0	0	0	0	0	0	0	0	0	0	0	0	0	0	0	0	0	0
E1541	Global 1983-2008 (Mentzer et al)	L3	CFA/I+CS21	1989	1	0	0	0	0	0	0	0	0	0	0	1	0	0	0	1	0	0	0	0
E1542	Global 1983-2008 (Mentzer et al)	L11+L15	CFnegative	1989	1	0	0	0	0	0	0	0	0	0	0	0	0	0	0	0	0	0	0	0
E1543	Global 1983-2008 (Mentzer et al)	NA	CFnegative	1989	0	0	0	0	0	0	0	0	0	0	0	0	0	0	0	0	0	0	0	0
E1544	Global 1983-2008 (Mentzer et al)	NA	CFnegative	1989	0	0	0	0	0	0	0	0	0	0	0	0	0	0	0	0	0	0	0	0
E1548	Global 1983-2008 (Mentzer et al)	L6	CFA/I+CS21	1989	1	0	0	0	0	0	0	0	0	0	0	1	0	0	0	1	0	0	1	0
E1556	Global 1983-2008 (Mentzer et al)	L12+L14	CS28B	1989	1	0	0	0	0	0	0	0	0	0	0	1	0	1	0	1	0	0	0	0
E1561	Global 1983-2008 (Mentzer et al)	L12+L14	CS28B	1989	1	0	0	0	0	0	0	0	0	0	0	0	0	1	0	1	0	0	0	1

StrainID	Study Dataset	ETEC_Lineage	CFs	Year	ampicillin.TEM	macrolide.mrx	macrolide.mphA.E.G.	macrolide.msrE	ESBL.CTXM	ESBL.DHA1	ESBL.Carbapenum	Fluoroquinolone.QnrB	Fluoroquinolone.QnrS	Fluoroquinolone.gyrB	Fluoroquiolone.parC	strA.APH6	strB.APH3	Trimethoprim.dfr	sulphonamide.sul1	sulphonamide.sul2	sulphonamide.sul3	chloramphenicol.CAT	tetracyclin.tetA	tetracyclin.tetB
E1564	Global 1983-2008 (Mentzer et al)	L9+L10+L21	CFnegative	1989	0	0	0	0	0	0	0	0	0	0	0	0	0	0	0	0	0	0	0	0
E157	Global 1983-2008 (Mentzer et al)	NA	CS6	1983	0	0	0	0	0	0	0	0	0	0	0	1	0	0	0	1	0	0	0	0
E1573	Global 1983-2008 (Mentzer et al)	L11+L15	CFnegative	1989	1	0	0	0	0	0	0	0	0	0	0	1	0	1	0	1	0	0	0	0
E1574	Global 1983-2008 (Mentzer et al)	L11+L13	CS27B	1989	1	0	0	0	0	0	0	0	0	0	0	1	0	1	0	1	0	0	0	0
E1576	Global 1983-2008 (Mentzer et al)	L20	CS13	1989	1	0	0	0	0	0	0	0	0	0	0	0	0	1	0	1	0	0	0	1
E1580	Global 1983-2008 (Mentzer et al)	L11+L13	CS27A	1989	1	0	0	0	0	0	0	0	0	0	0	1	0	0	0	1	0	0	1	0
E1581	Global 1983-2008 (Mentzer et al)	L11+L13	CS13+CS26	1989	1	0	0	0	0	0	0	0	0	0	0	1	0	1	0	1	0	0	0	0
E1582	Global 1983-2008 (Mentzer et al)	L11+L13	CS27B	1989	0	0	0	0	0	0	0	0	0	0	0	0	0	1	0	0	0	0	0	0
E1585	Global 1983-2008 (Mentzer et al)	L11+L13	CFnegative	1989	0	0	0	0	0	0	0	0	0	0	0	1	0	0	0	0	0	0	0	1
E1586	Global 1983-2008 (Mentzer et al)	NA	CS30	1989	0	0	0	0	0	0	0	0	0	0	0	0	0	0	0	0	0	0	0	0
E1587	Global 1983-2008 (Mentzer et al)	L3	CFA/I+CS21	1989	0	0	0	0	0	0	0	0	0	0	0	1	0	0	0	1	0	0	0	0
E159	Global 1983-2008 (Mentzer et al)	L11+L13	CFnegative	1983	0	0	0	0	0	0	0	0	0	0	0	0	0	0	0	0	0	0	0	0
E1592	Global 1983-2008 (Mentzer et al)	L3	CS7+CS28B	1989	1	0	0	0	0	0	0	0	0	0	0	1	0	1	0	1	0	0	0	0
E1593	Global 1983-2008 (Mentzer et al)	L17	CS5+CS6	1989	0	0	0	0	0	0	0	0	0	0	0	0	0	0	1	0	0	1	0	1
E1594	Global 1983-2008 (Mentzer et al)	L11+L15	CFnegative	1989	1	0	0	0	0	0	0	0	0	0	0	0	0	0	0	0	0	0	0	0
E1596	Global 1983-2008 (Mentzer et al)	L12+L14	CS28B	1989	1	0	0	0	0	0	0	0	0	0	0	0	0	1	0	1	0	0	0	0
E1597	Global 1983-2008 (Mentzer et al)	L29	CS20	1989	1	0	0	0	0	0	0	0	0	0	0	0	0	1	0	1	0	0	0	0
E1599	Global 1983-2008 (Mentzer et al)	L23	CFnegative	1989	0	0	0	0	0	0	0	0	0	0	0	0	0	0	0	0	0	0	0	0
E160	Global 1983-2008 (Mentzer et al)	NA	CFnegative	1983	1	0	0	0	0	0	0	0	0	0	0	0	0	0	1	0	0	1	0	0
E1600	Global 1983-2008 (Mentzer et al)	L11+L13	CS13	1989	1	0	0	0	0	0	0	0	0	0	0	1	0	1	0	1	0	0	0	0
E1604	Global 1983-2008 (Mentzer et al)	L6	CFA/I+CS21	1989	1	0	0	0	0	0	0	0	0	0	0	1	0	0	0	1	0	0	1	0
E1607	Global 1983-2008 (Mentzer et al)	L16	CS14	1989	0	0	0	0	0	0	0	0	0	0	0	0	0	1	0	0	0	0	0	0
E1609	Global 1983-2008 (Mentzer et al)	L9+L10+L21	CS12	1989	1	0	0	0	0	0	0	0	0	0	0	1	0	0	0	1	0	0	0	0
E1611	Global 1983-2008 (Mentzer et al)	NA	CS6	1989	1	0	0	0	0	0	0	0	0	0	0	0	0	0	0	0	0	0	0	0
E1615	Global 1983-2008 (Mentzer et al)	L11+L13	CS13	1989	1	0	1	0	0	0	0	0	0	0	0	1	0	1	0	1	0	0	0	0
E1616	Global 1983-2008 (Mentzer et al)	L20	CFnegative	1989	1	0	0	0	0	0	0	0	0	0	0	1	0	1	0	1	0	0	0	0
E1617	Global 1983-2008 (Mentzer et al)	L29	CS20	1989	1	0	0	0	0	0	0	0	0	0	0	0	0	0	0	0	0	0	0	0
E1620	Global 1983-2008 (Mentzer et al)	L1	CS1+CS3+CS21	1989	1	0	0	0	0	0	0	0	0	0	0	1	0	1	0	1	0	0	0	0
E1623	Global 1983-2008 (Mentzer et al)	L16	CFnegative	1990	0	0	0	0	0	0	1	0	0	0	0	0	0	0	1	0	0	1	0	1
E1624	Global 1983-2008 (Mentzer et al)	L2	CS2+CS3+CS21	1990	0	0	0	0	0	0	0	0	0	0	0	0	0	0	0	0	0	0	0	0
E1625	Global 1983-2008 (Mentzer et al)	NA	CS6	1990	0	0	0	0	0	0	0	0	0	0	0	0	0	0	0	0	0	0	0	0
E1628	Global 1983-2008 (Mentzer et al)	L4	CS6+CS21	1990	1	0	0	0	0	0	0	0	0	0	0	0	0	0	0	0	0	0	0	0
E1634	Global 1983-2008 (Mentzer et al)	L11+L13	CFnegative	1990	0	0	0	0	0	0	0	0	0	0	0	1	0	0	0	1	0	0	1	0
E1635	Global 1983-2008 (Mentzer et al)	L5	CS17	1997	0	0	0	0	0	0	0	0	0	0	0	0	0	0	0	0	0	0	0	0
E1637	Global 1983-2008 (Mentzer et al)	L16	CFnegative	1990	0	0	0	0	0	0	1	0	0	0	0	0	0	0	1	0	0	1	0	1
E1638	Global 1983-2008 (Mentzer et al)	L20	CFnegative	1990	0	0	0	0	0	0	0	0	0	0	0	0	0	0	0	0	0	0	0	0
E1640	Global 1983-2008 (Mentzer et al)	L18	CS28A	1990	0	0	0	0	0	0	0	0	0	0	0	0	0	0	0	0	0	0	0	0
E1641	Global 1983-2008 (Mentzer et al)	L29	CS23	1990	1	0	0	0	0	0	0	0	0	0	0	1	0	0	0	1	0	1	0	1
E1642	Global 1983-2008 (Mentzer et al)	NA	CS20	1990	0	0	0	0	0	0	0	0	0	0	0	0	0	0	0	0	0	0	0	0
E1646	Global 1983-2008 (Mentzer et al)	L5	CS5+CS6	1990	0	0	0	0	0	0	0	0	0	0	0	0	0	0	0	0	0	0	0	0
E1647	Global 1983-2008 (Mentzer et al)	L20	CS6	1990	0	0	0	0	0	0	0	0	0	0	0	0	0	0	0	0	0	0	0	0
E1648	Global 1983-2008 (Mentzer et al)	L1	CS1+CS3+CS21	1990	0	0	0	0	0	0	0	0	0	0	0	0	0	0	0	0	0	0	0	0
E1649	Global 1983-2008 (Mentzer et al)	L2	CS2+CS3+CS21	1997	0	0	0	0	0	0	0	0	0	0	0	0	0	0	0	0	0	0	0	0
E1650	Global 1983-2008 (Mentzer et al)	L20	CFnegative	1990	0	0	0	0	0	0	0	0	0	0	0	0	0	0	0	0	0	0	0	0
E1654	Global 1983-2008 (Mentzer et al)	L1	CS1+CS3+CS21	1990	0	0	0	0	0	0	0	0	0	0	0	0	0	0	0	0	0	0	0	0
E1657	Global 1983-2008 (Mentzer et al)	L18	CS12	1997	0	0	0	0	0	0	0	0	0	0	0	0	0	0	0	0	0	0	0	0
E1659	Global 1983-2008 (Mentzer et al)	L20	CFnegative	1990	0	0	0	0	0	0	0	0	0	0	0	0	0	0	0	0	0	0	0	1
E1661	Global 1983-2008 (Mentzer et al)	L28	CS28B	1990	0	0	0	0	0	0	0	0	0	0	0	0	0	0	0	0	0	0	0	0
E1666	Global 1983-2008 (Mentzer et al)	L3	CFA/I+CS21	1990	0	0	0	0	0	0	0	0	0	0	0	1	0	0	1	1	0	1	1	1
E1667	Global 1983-2008 (Mentzer et al)	L2	CS2+CS3+CS21	1997	0	0	0	0	0	0	0	0	0	0	0	0	0	0	0	0	0	0	0	0
E167	Global 1983-2008 (Mentzer et al)	L11+L13	CFnegative	1983	0	0	0	0	0	0	0	0	0	0	0	0	0	0	0	0	0	0	0	0
E1673	Global 1983-2008 (Mentzer et al)	L19	CFnegative	1990	0	0	0	0	0	0	0	0	0	0	0	0	0	0	0	0	0	0	0	0
E1674	Global 1983-2008 (Mentzer et al)	L16	CFnegative	1990	0	0	0	0	0	0	1	0	0	0	0	0	0	0	1	0	0	1	0	1
E1679sec	Global 1983-2008 (Mentzer et al)	L11+L15	CS12	1990	0	0	0	0	0	0	0	0	0	0	0	0	0	0	0	0	0	0	0	0
E1682	Global 1983-2008 (Mentzer et al)	L19	CS27B	1990	0	0	0	0	0	0	0	0	0	0	0	0	0	0	0	0	0	0	0	0
E1684	Global 1983-2008 (Mentzer et al)	L17	CS6	1990	0	0	0	0	0	0	0	0	0	0	0	0	0	0	0	0	0	0	0	0
E1690	Global 1983-2008 (Mentzer et al)	L11+L13	CS14	1990	1	0	0	0	0	0	0	0	0	0	0	1	0	0	0	1	0	0	1	0
E1712	Bangladesh 1980-2011 (Mentzer et al)	L17	CS14	2001	1	0	0	0	0	0	0	0	0	0	0	0	0	1	0	1	0	0	0	0
E1716	Bangladesh 1980-2011 (Mentzer et al)	L3	CFA/I+CS21	2001	1	0	0	0	0	0	0	0	0	0	0	1	0	1	0	1	0	0	0	1
E1724	Bangladesh 1980-2011 (Mentzer et al)	L5	CS5+CS6	2001	0	0	0	0	0	0	0	0	0	0	0	0	0	0	0	0	0	0	0	0
E1735	Bangladesh 1980-2011 (Mentzer et al)	L17	CS14	2002	1	0	0	0	0	0	0	0	0	0	0	0	0	0	0	0	0	0	0	0
E1736	Bangladesh 1980-2011 (Mentzer et al)	L17	CS14	2002	0	0	0	0	0	0	0	0	0	0	0	0	0	0	0	0	0	0	0	0
E1739	Bangladesh 1980-2011 (Mentzer et al)	L1	CS1+CS3+CS21	2002	0	0	0	0	0	0	0	0	0	0	0	0	0	0	0	0	0	0	0	0
E1741	Bangladesh 1980-2011 (Mentzer et al)	L5	CS17	2002	0	0	0	0	0	0	0	0	0	0	0	0	0	0	0	0	0	0	0	0

StrainID	Study Dataset	ETEC_Lineage	CFs	Year	ampicillin.TEM	macrolide.mrx	macrolide.mphA.E.G.	macrolide.msrE	ESBL.CTXM	ESBL.DHA1	ESBL.Carbapenum	Fluoroquinolone.QnrB	Fluoroquinolone.QnrS	Fluoroquinolone.gyrB	Fluoroquiolone.parC	strA.APH6	strB.APH3	Trimethoprim.dfr	sulphonamide.sul1	sulphonamide.sul2	sulphonamide.sul3	chloramphenicol.CAT	tetracyclin.tetA	tetracyclin.tetB
E1744	Bangladesh 1980-2011 (Mentzer et al)	L17	CFnegative	2002	1	0	0	0	0	0	0	0	0	0	0	0	0	0	0	0	0	0	0	0
E1750	Bangladesh 1980-2011 (Mentzer et al)	L5	CS5+CS6	2002	0	0	0	0	0	0	0	0	0	0	0	0	0	0	0	0	0	0	0	0
E1752	Bangladesh 1980-2011 (Mentzer et al)	L4	CS6	2002	0	0	0	0	0	0	0	0	0	0	0	0	0	1	1	0	0	1	0	1
E1760	Bangladesh 1980-2011 (Mentzer et al)	L5	CS5+CS6	2002	1	0	0	0	0	0	0	0	0	0	0	1	0	1	0	1	0	0	0	0
E1779	Bangladesh 1980-2011 (Mentzer et al)	L5	CS5+CS6	2005	0	0	0	0	0	0	0	0	0	0	0	0	0	0	0	0	0	0	0	0
E1784	Bangladesh 1980-2011 (Mentzer et al)	L20	CS6	2005	0	0	0	0	0	0	0	0	0	0	0	0	0	0	0	0	0	0	0	0
E1795	Bangladesh 1980-2011 (Mentzer et al)	L11+L15	CFA/I+CS21	2007	1	0	0	0	0	0	0	0	0	0	0	1	0	1	0	1	0	0	0	0
E1797	Bangladesh 1980-2011 (Mentzer et al)	L1	CS1+CS3+CS21	2007	1	0	0	0	0	0	0	0	0	0	0	1	0	1	0	1	0	0	0	1
E1841	Bangladesh 1980-2011 (Mentzer et al)	L3	CFA/I+CS21	2007	0	0	0	0	0	0	0	0	0	0	0	0	0	1	1	0	0	0	0	1
E1871	Bangladesh 1980-2011 (Mentzer et al)	L17	CS14	2007	0	0	0	0	0	0	0	0	0	0	0	0	0	0	0	0	0	0	0	0
E1883	Bangladesh 1980-2011 (Mentzer et al)	L2	CS2+CS3+CS21	2007	0	0	0	0	0	0	0	0	0	0	0	0	0	0	0	0	0	0	0	0
E1918	Bangladesh 1980-2011 (Mentzer et al)	L5	CS5+CS6	2007	0	0	0	0	0	0	0	0	0	0	0	0	0	0	0	0	0	0	0	0
E1939	Bangladesh 1980-2011 (Mentzer et al)	L3	CS7	2007	1	0	0	0	0	0	0	0	0	0	0	1	0	1	0	1	0	0	0	1
E1947	Bangladesh 1980-2011 (Mentzer et al)	L3	CFA/I+CS21	2007	1	0	0	0	0	0	0	0	0	0	0	1	0	1	0	1	0	0	0	1
E1961	Bangladesh 1980-2011 (Mentzer et al)	NA	CS19	2007	0	0	0	0	0	0	0	0	0	0	0	0	0	0	0	0	0	0	0	0
E1994	Bangladesh 1980-2011 (Mentzer et al)	L5	CS17	2007	0	0	0	0	0	0	0	0	0	0	0	0	0	0	0	0	0	0	0	0
E2088	Bangladesh 1980-2011 (Mentzer et al)	L3	CFA/I+CS21	2007	0	0	0	0	0	0	0	0	0	0	0	0	0	0	1	0	0	0	0	1
E2092	Bangladesh 1980-2011 (Mentzer et al)	L5	CS5+CS6	2007	0	0	0	0	0	0	0	0	0	0	0	0	0	0	0	0	0	0	0	0
E21	Bangladesh 1980-2011 (Mentzer et al)	L5	CS5+CS6	1980	0	0	0	0	0	0	0	0	0	0	0	0	0	0	0	0	0	0	0	0
E2108	Bangladesh 1980-2011 (Mentzer et al)	L8	CS6+CS12+CS21	2007	0	0	0	0	0	0	0	0	0	0	0	0	0	0	0	0	0	0	0	0
E2110	Bangladesh 1980-2011 (Mentzer et al)	L8	CS6+CS21	2007	0	0	0	0	0	0	0	0	0	0	0	0	0	0	0	0	0	0	0	0
E2118	Bangladesh 1980-2011 (Mentzer et al)	L5	CS17	2007	0	0	0	0	0	0	0	0	0	0	0	0	0	0	0	0	0	0	0	0
E2121	Bangladesh 1980-2011 (Mentzer et al)	L8	CS6+CS21	2007	0	0	0	0	0	0	0	0	0	0	0	0	0	0	0	0	0	0	0	0
E2131	Bangladesh 1980-2011 (Mentzer et al)	L3	CS21	2007	0	0	0	0	0	0	0	0	0	0	0	0	0	0	0	0	0	0	0	0
E2185	Global 1983-2008 (Mentzer et al)	NA	CS17	2007	0	0	0	0	0	0	0	0	0	0	0	0	0	0	0	0	0	1	0	0
E220	Global 1983-2008 (Mentzer et al)	L9+L10+L21	CS6	1987	0	0	0	0	0	0	0	0	0	0	0	0	0	0	0	0	0	0	0	0
E2219	Global 1983-2008 (Mentzer et al)	L1	CS1+CS3+CS21	2007	1	0	0	0	0	0	0	0	0	0	0	1	0	1	0	1	0	0	0	0
E222	Global 1983-2008 (Mentzer et al)	L8	CS21	1987	0	0	0	0	0	0	0	0	0	0	0	0	0	0	0	0	0	0	0	0
E223	Global 1983-2008 (Mentzer et al)	L9+L10+L21	CFnegative	1987	0	0	0	0	0	0	0	0	0	0	0	0	0	0	0	0	0	0	0	0
E224	Global 1983-2008 (Mentzer et al)	L8	CS6+CS21	1987	0	0	0	0	0	0	0	0	0	0	0	0	0	0	0	0	0	0	0	0
E2256	Global 1983-2008 (Mentzer et al)	L7	CS6	2007	1	0	0	0	0	0	0	0	0	0	0	1	0	1	1	1	0	0	1	1
E2339	Global 1983-2008 (Mentzer et al)	L5	CS5+CS6	2007	0	0	0	0	0	0	0	0	0	0	0	1	0	1	0	1	0	0	1	0
E2347	Global 1983-2008 (Mentzer et al)	L3	CFnegative	2007	0	0	0	0	0	0	0	0	0	0	0	0	0	0	0	0	0	0	0	0
E2348	Global 1983-2008 (Mentzer et al)	L16	CFnegative	2007	1	0	0	0	0	0	0	0	0	0	0	1	0	1	0	1	0	0	0	1
E2362sec	Global 1983-2008 (Mentzer et al)	NA	CS27A	2008	1	0	0	0	0	0	0	0	0	0	0	1	0	1	1	1	0	0	1	0
E2367	Global 1983-2008 (Mentzer et al)	L9+L10+L21	CFnegative	2008	1	0	0	0	0	0	0	0	0	0	0	1	0	1	0	1	0	0	0	1
E237	Global 1983-2008 (Mentzer et al)	L9+L10+L21	CS6	1987	0	0	0	0	0	0	0	0	0	0	0	0	0	0	0	0	0	0	0	0
E2370sec	Global 1983-2008 (Mentzer et al)	L11+L13	CFnegative	2008	0	0	0	0	0	0	0	0	0	0	0	0	0	0	0	0	0	0	0	0
E2371	Global 1983-2008 (Mentzer et al)	L11+L13	CFnegative	2008	1	0	0	0	0	0	0	0	0	0	0	1	0	1	0	1	0	0	0	0
E2377	Global 1983-2008 (Mentzer et al)	L11+L15	CS6	2007	1	0	0	0	0	0	0	0	0	0	0	1	0	1	0	1	0	0	0	0
E2386	Global 1983-2008 (Mentzer et al)	L1	CS1+CS3+CS21	2007	1	0	0	0	0	0	0	0	0	0	0	1	0	1	0	1	0	0	0	0
E2388	Global 1983-2008 (Mentzer et al)	NA	CS27A	2007	1	0	0	0	0	0	0	0	0	0	0	1	0	1	1	1	0	0	1	0
E239	Global 1983-2008 (Mentzer et al)	L28	CS20	1987	0	0	0	0	0	0	0	0	0	0	0	0	0	0	0	0	0	0	0	0
E2392	Global 1983-2008 (Mentzer et al)	L11+L15	CFnegative	2007	1	0	0	0	0	0	0	0	0	0	0	1	0	1	0	1	0	0	0	1
E2393	Global 1983-2008 (Mentzer et al)	L16	PCFO71	2007	0	0	0	0	0	0	0	0	0	0	0	0	0	0	0	0	0	0	0	0
E2395	Global 1983-2008 (Mentzer et al)	L20	CS14	2007	1	0	0	0	0	0	0	0	0	0	0	0	0	1	1	1	0	0	0	0
E2397	Global 1983-2008 (Mentzer et al)	L5	CS5+CS6	2007	1	0	0	0	0	0	0	0	0	0	0	1	0	1	0	1	0	0	0	0
E2404	Global 1983-2008 (Mentzer et al)	L17	CFnegative	2008	1	0	0	0	0	0	0	0	0	0	0	1	0	1	0	1	0	0	0	0
E2405	Global 1983-2008 (Mentzer et al)	L17	CFnegative	2008	1	0	0	0	0	0	0	0	0	0	0	1	0	1	0	1	0	0	0	0
E2439	Global 1983-2008 (Mentzer et al)	NA	CFnegative	2008	1	0	0	0	0	0	0	0	0	0	0	0	0	1	1	0	0	0	0	0
E251	Global 1983-2008 (Mentzer et al)	L9+L10+L21	CFnegative	1987	0	0	0	0	0	0	0	0	0	0	0	0	0	0	0	0	0	0	0	0
E263	Global 1983-2008 (Mentzer et al)	L1	CS1+CS21	1987	0	0	0	0	0	0	0	0	0	0	0	0	0	0	0	0	0	0	0	0
E272	Global 1983-2008 (Mentzer et al)	L2	CS2+CS3	1987	0	0	0	0	0	0	0	0	0	0	0	0	0	0	0	0	0	1	0	1
E28	Bangladesh 1980-2011 (Mentzer et al)	L11+L15	CFnegative	1980	0	0	0	0	0	0	0	0	0	0	0	0	0	0	0	0	0	0	0	0
E2980	Bangladesh 1980-2011 (Mentzer et al)	L3	CS7	2010	1	0	0	0	0	0	0	0	0	0	0	1	0	0	0	1	0	0	0	0
E2981sec	Bangladesh 1980-2011 (Mentzer et al)	NA	CFnegative	2010	0	0	0	0	1	0	0	0	0	0	0	0	0	0	0	0	0	0	1	0
E3015	Global 1983-2008 (Mentzer et al)	L17	CS6	1998	1	0	0	0	0	0	0	0	0	0	0	1	0	1	1	1	0	1	1	0
E329	Global 1983-2008 (Mentzer et al)	L11+L15	CFnegative	1998	0	0	0	0	0	0	0	0	0	0	0	0	0	0	0	0	0	0	0	1
E330	Global 1983-2008 (Mentzer et al)	L12+L14	CS28B	1998	1	0	0	0	0	0	0	0	0	0	0	0	0	0	0	1	0	0	0	0
E333	Global 1983-2008 (Mentzer et al)	L17	CS6	1998	0	0	0	0	0	0	0	0	0	0	0	0	0	0	0	1	0	0	0	1
E335	Global 1983-2008 (Mentzer et al)	L8	CS6+CS21	1998	0	0	0	0	0	0	0	0	0	0	0	0	0	0	0	0	0	0	0	0
E336	Global 1983-2008 (Mentzer et al)	L8	CS6+CS21	1998	0	0	0	0	0	0	0	0	0	0	0	0	0	0	0	0	0	0	0	0
E340	Global 1983-2008 (Mentzer et al)	L4	CS21	1998	0	0	0	0	0	0	0	0	0	0	0	0	0	0	0	0	0	0	0	0
E343	Global 1983-2008 (Mentzer et al)	L4	CS21	1998	0	0	0	0	0	0	0	0	0	0	0	0	0	0	0	0	0	0	0	0
E344	Global 1983-2008 (Mentzer et al)	L7	CS6	1998	0	0	0	0	0	0	0	0	0	0	0	0	0	0	0	0	0	0	0	1
E351	Global 1983-2008 (Mentzer et al)	L16	CFnegative	1998	1	0	0	0	0	0	0	0	0	0	0	1	0	1	0	1	0	0	0	1
E354	Global 1983-2008 (Mentzer et al)	L11+L15	CFnegative	1998	0	0	0	0	0	0	0	1	0	0	0	1	0	0	0	0	0	0	0	0
E356	Global 1983-2008 (Mentzer et al)	L11+L15	PCFO71+CS21	1998	0	0	0	0	0	0	0	0	0	0	0	1	0	0	0	0	0	0	1	0
E36	Bangladesh 1980-2011 (Mentzer et al)	L3	CFA/I+CS21	1980	0	0	0	0	0	0	0	0	0	0	0	0	0	0	0	0	0	0	0	1
E360	Global 1983-2008 (Mentzer et al)	L16	PCFO71+CS21	1998	0	0	0	0	0	0	0	0	0	0	0	0	0	0	0	0	0	0	0	0
E361	Global 1983-2008 (Mentzer et al)	L20	CS13+CS30	1998	1	1	1	0	0	0	0	0	0	0	0	0	0	0	0	0	0	0	0	0
E370	Global 1983-2008 (Mentzer et al)	L7	CS6	1998	1	0	0	0	0	0	0	0	0	0	0	1	0	0	0	1	0	0	0	1
E390	Global 1983-2008 (Mentzer et al)	L24	CFnegative	1999	1	0	0	0	0	0	0	0	0	0	0	1	0	1	0	1	0	1	0	1
E391	Global 1983-2008 (Mentzer et al)	L18	CFnegative	1999	1	0	0	0	0	0	0	0	0	0	0	1	0	1	0	1	0	0	0	0
E399	Global 1983-2008 (Mentzer et al)	L11+L13	CFnegative	1999	0	0	0	0	0	0	0	0	0	0	0	0	0	0	0	0	0	0	1	0
E405	Global 1983-2008 (Mentzer et al)	L12+L14	CS28B	1999	1	0	0	0	0	0	0	0	0	0	0	0	0	0	0	1	0	0	0	0
E4092	Global 1983-2008 (Mentzer et al)	L4	CS6+CS8	2005	0	0	0	0	0	0	0	0	0	0	0	0	0	0	0	0	0	0	0	0
E415	Global 1983-2008 (Mentzer et al)	L11+L15	CS6	1999	1	0	0	0	0	0	0	0	0	0	0	1	0	1	0	1	0	0	1	0
E416	Global 1983-2008 (Mentzer et al)	L17	CS6	1999	0	0	0	0	0	0	0	0	0	0	0	0	0	0	0	1	0	0	0	1
E425	Global 1983-2008 (Mentzer et al)	L11+L15	CS6	1999	1	0	0	0	0	0	0	0	0	0	0	1	0	1	0	1	0	0	0	1
E445	Global 1983-2008 (Mentzer et al)	L16	PCFO71	2000	0	0	0	0	0	0	0	0	0	0	0	0	0	0	0	0	0	0	0	0
E45	Bangladesh 1980-2011 (Mentzer et al)	L3	CFA/I+CS21	1980	1	0	0	0	0	0	0	0	0	0	0	1	0	0	0	1	0	0	0	1
E451	Global 1983-2008 (Mentzer et al)	L11+L15	CS27B	2000	0	0	0	0	0	0	0	0	0	0	0	0	0	0	0	0	0	0	0	0
E471	Global 1983-2008 (Mentzer et al)	L1	CS1+CS3+CS21	2000	0	0	0	0	0	0	0	0	0	0	0	0	0	0	0	0	0	0	0	0
E5049	Global 1983-2008 (Mentzer et al)	L23	CS6+CS13	unkown	0	0	0	0	0	0	0	0	0	0	0	0	0	0	0	0	0	0	1	0
E5051	Global 1983-2008 (Mentzer et al)	L23	CS6	unkown	1	0	0	0	0	0	0	0	0	0	0	0	0	1	0	1	0	0	1	0

StrainID	Study Dataset	ETEC_Lineage	CFs	Year	ampicillin.TEM	macrolide.mrx	macrolide.mphA.E.G.	macrolide.msrE	ESBL.CTXM	ESBL.DHA1	ESBL.Carbapenum	Fluoroquinolone.QnrB	Fluoroquinolone.QnrS	Fluoroquinolone.gyrB	Fluoroquiolone.parC	strA.APH6	strB.APH3	Trimethoprim.dfr	sulphonamide.sul1	sulphonamide.sul2	sulphonamide.sul3	chloramphenicol.CAT	tetracyclin.tetA	tetracyclin.tetB
E5052	Global 1983-2008 (Mentzer et al)	L11+L13	CS6	unkown	1	0	0	0	0	0	0	0	0	0	0	1	0	1	1	1	0	1	1	0
E5080	Bangladesh 1980-2011 (Mentzer et al)	L5	CS5+CS6	2011	0	0	0	0	0	0	0	0	0	0	0	0	0	0	0	0	0	0	0	0
E5081	Bangladesh 1980-2011 (Mentzer et al)	L5	CS5+CS6	2011	0	0	0	0	0	0	0	0	0	0	0	0	0	0	0	0	0	0	0	0
E5082	Bangladesh 1980-2011 (Mentzer et al)	L5	CS5+CS6	2011	0	0	0	0	0	0	0	0	0	0	0	0	0	0	0	0	0	0	0	0
E5084	Bangladesh 1980-2011 (Mentzer et al)	L7	CS6	2011	1	1	1	0	0	0	0	0	0	0	0	1	0	1	0	1	0	0	0	1
E5085	Bangladesh 1980-2011 (Mentzer et al)	L18	CS6	2011	1	1	1	0	0	0	0	0	0	0	0	0	0	0	0	0	0	0	0	0
E5086	Bangladesh 1980-2011 (Mentzer et al)	L3	CFA/I+CS21	2011	1	1	1	0	0	0	0	0	0	0	0	0	0	1	0	0	0	0	0	1
E5087	Bangladesh 1980-2011 (Mentzer et al)	NA	CFnegative	2011	1	1	1	0	1	0	0	0	0	0	1	1	0	1	1	1	0	0	0	0
E5088	Bangladesh 1980-2011 (Mentzer et al)	L18	CS6	2011	1	1	1	0	0	0	0	0	0	0	0	0	0	0	0	0	0	0	0	0
E5089	Bangladesh 1980-2011 (Mentzer et al)	L11+L13	CFnegative	2011	1	0	0	0	0	0	0	0	0	0	0	1	0	0	0	0	0	0	0	0
E509	Global 1983-2008 (Mentzer et al)	L23	CFnegative	2000	0	0	0	0	0	0	0	0	0	0	0	0	0	0	0	0	0	0	0	0
E513	Global 1983-2008 (Mentzer et al)	L11+L15	CFnegative	2000	1	0	0	0	0	0	0	0	0	0	0	1	0	0	0	0	0	0	1	0
E517	Global 1983-2008 (Mentzer et al)	L1	CS1+CS3+CS21	2000	0	0	0	0	0	0	0	0	0	0	0	0	0	0	0	0	0	0	0	0
E519	Global 1983-2008 (Mentzer et al)	L18	CFnegative	2000	0	0	0	0	0	0	0	0	0	0	0	0	0	0	0	0	0	0	0	0
E520	Global 1983-2008 (Mentzer et al)	L7	CS6	2000	0	0	0	0	0	0	0	0	0	0	0	0	0	0	0	0	0	0	0	1
E523	Global 1983-2008 (Mentzer et al)	NA	CFnegative	2000	0	0	0	0	0	0	0	0	0	0	0	0	0	0	1	0	0	0	1	0
E527	Global 1983-2008 (Mentzer et al)	L18	CFnegative	2000	0	0	0	0	0	0	0	0	0	0	0	0	0	0	0	0	0	0	0	0
E528	Global 1983-2008 (Mentzer et al)	L18	CS23+CS26	2000	0	0	0	0	0	0	0	0	0	0	0	0	0	0	0	0	0	0	0	0
E529	Global 1983-2008 (Mentzer et al)	L9+L10+L21	CS13	2000	0	0	0	0	0	0	0	0	0	0	0	0	0	0	0	0	0	0	0	0
E54	Bangladesh 1980-2011 (Mentzer et al)	L1	CS1+CS3+CS21	1980	0	0	0	0	0	0	0	0	0	0	0	0	0	0	0	0	0	0	0	0
E554	Global 1983-2008 (Mentzer et al)	L7	CS6	2000	0	0	0	0	0	0	0	0	0	0	0	0	0	0	0	0	0	0	0	1
E562	Global 1983-2008 (Mentzer et al)	L6	CFA/I+CS21	2000	1	0	0	0	0	0	0	0	0	0	0	0	0	0	0	0	0	0	0	0
E563	Global 1983-2008 (Mentzer et al)	L6	CFA/I+CS21	2000	1	0	0	0	0	0	0	0	0	0	0	0	0	0	0	0	0	0	0	0
E604	Global 1983-2008 (Mentzer et al)	L11+L13	CS27A	2000	1	0	0	0	0	0	0	0	0	0	0	1	0	1	0	1	0	0	0	1
E616	Global 1983-2008 (Mentzer et al)	L11+L13	CS27A	2000	1	0	0	0	0	0	0	0	0	0	0	0	0	1	0	1	0	0	0	1
E618	Global 1983-2008 (Mentzer et al)	L23	CFnegative	2000	0	0	0	0	0	0	0	0	0	0	0	0	0	0	0	0	0	0	0	0
E620	Global 1983-2008 (Mentzer et al)	L12+L14	CFnegative	2000	0	0	0	0	0	0	0	0	0	0	0	0	0	0	0	0	0	0	0	0
E621	Global 1983-2008 (Mentzer et al)	L20	CFnegative	2000	0	0	0	0	0	0	0	0	0	0	0	0	0	0	0	0	0	0	0	0
E622	Global 1983-2008 (Mentzer et al)	L19	CFnegative	2000	1	0	0	0	0	0	0	0	0	0	0	0	0	1	1	0	0	0	1	0
E626	Global 1983-2008 (Mentzer et al)	L12+L14	CFnegative	2000	0	0	0	0	0	0	0	0	0	0	0	0	0	0	0	0	0	0	0	0
E628	Global 1983-2008 (Mentzer et al)	L11+L13	CS27A	2000	1	0	0	0	0	0	0	0	0	0	0	0	0	1	0	1	0	0	0	1
E632	Global 1983-2008 (Mentzer et al)	L1	CS1+CS3+CS21	2000	0	0	0	0	0	0	0	0	0	0	0	0	0	0	0	0	0	0	0	0
E636	Global 1983-2008 (Mentzer et al)	L6	CFA/I+CS21	2000	1	0	0	0	0	0	0	0	0	0	0	0	0	1	0	0	0	0	0	0
E645	Global 1983-2008 (Mentzer et al)	L19	CS27B	2000	0	0	0	0	0	0	0	0	0	0	0	0	0	1	0	0	0	0	1	0
E655	Global 1983-2008 (Mentzer et al)	L5	CS5+CS6	2000	0	0	0	0	0	0	0	0	0	0	0	0	0	0	0	0	0	0	0	0
E658	Global 1983-2008 (Mentzer et al)	L3	CFA/I+CS21	2001	0	0	0	0	0	0	0	0	0	0	0	0	0	0	1	0	0	0	0	1
E659	Global 1983-2008 (Mentzer et al)	L18	CFnegative	2001	1	0	0	0	0	0	0	0	0	0	0	0	0	1	1	0	0	0	1	0
E66	Bangladesh 1980-2011 (Mentzer et al)	L2	CS2+CS3+CS21	1988	0	0	0	0	0	0	0	0	0	0	0	0	0	0	0	0	0	0	0	0
E662	Global 1983-2008 (Mentzer et al)	L12+L14	CFnegative	2001	0	0	0	0	0	0	0	0	0	0	0	0	0	0	0	0	0	0	0	0
E70	Bangladesh 1980-2011 (Mentzer et al)	L2	CS2+CS3+CS21	1988	0	0	0	0	0	0	0	0	0	0	0	0	0	0	0	0	0	0	0	0
E703	Global 1983-2008 (Mentzer et al)	L23	CFnegative	2001	0	0	0	0	0	0	0	0	0	0	0	0	0	0	0	0	0	0	1	0
E704	Global 1983-2008 (Mentzer et al)	L12+L14	CFnegative	2001	0	0	0	0	0	0	0	0	0	0	0	0	0	0	0	0	0	0	0	0
E705	Global 1983-2008 (Mentzer et al)	L12+L14	CFnegative	2001	0	0	0	0	0	0	0	0	0	0	0	0	0	0	0	0	0	0	0	0
E71	Bangladesh 1980-2011 (Mentzer et al)	L2	CS2+CS3+CS21	1988	0	0	0	0	0	0	0	0	0	0	0	0	0	0	0	0	0	0	0	0
E710	Global 1983-2008 (Mentzer et al)	L20	CFnegative	2001	0	0	0	0	0	0	0	0	0	0	0	0	0	0	0	0	0	0	0	0
E74	Bangladesh 1980-2011 (Mentzer et al)	L3	CS21	1988	0	0	0	0	0	0	0	0	0	0	0	1	0	0	0	1	0	0	0	1
E79	Bangladesh 1980-2011 (Mentzer et al)	L16	CS3+CS21	1986	0	0	0	0	0	0	0	0	0	0	0	0	0	0	0	0	0	0	0	0
E8	Bangladesh 1980-2011 (Mentzer et al)	L1	CS1+CS3+CS21	1980	0	0	0	0	0	0	0	0	0	0	0	0	0	0	0	0	0	0	0	0
E806	Global 1983-2008 (Mentzer et al)	L9+L10+L21	CS6	2002	1	0	0	0	0	0	0	0	0	0	0	1	0	1	0	1	0	0	0	1
E810	Global 1983-2008 (Mentzer et al)	L3	CFA/I+CS21	2002	0	0	0	0	0	0	0	0	0	0	0	0	0	0	0	0	0	0	0	0
E811	Global 1983-2008 (Mentzer et al)	L12+L14	CFnegative	2002	0	0	0	0	0	0	0	0	0	0	0	0	0	0	0	0	0	0	1	0
E812	Global 1983-2008 (Mentzer et al)	L1	CS1+CS3+CS21	2002	0	0	0	0	0	0	0	0	0	0	0	0	0	0	0	0	0	0	0	0
E816	Global 1983-2008 (Mentzer et al)	L16	CS3+CS21	2002	1	0	0	0	0	0	0	0	0	0	0	0	0	1	1	0	0	0	1	0
E818	Global 1983-2008 (Mentzer et al)	L18	CS13	2002	1	0	0	0	0	0	0	0	0	0	0	1	0	1	0	1	0	0	0	1
E819	Global 1983-2008 (Mentzer et al)	L18	CFnegative	2002	1	0	0	0	0	0	0	0	0	0	0	0	0	1	1	0	0	0	1	0
E821	Global 1983-2008 (Mentzer et al)	L18	CFnegative	2002	1	0	0	0	0	0	0	0	0	0	0	0	0	1	1	0	0	0	1	0
E822	Global 1983-2008 (Mentzer et al)	L2	CS2+CS3+CS21	2002	0	0	0	0	0	0	0	0	0	0	0	1	0	0	0	0	0	0	0	1
E828	Global 1983-2008 (Mentzer et al)	L11+L15	CFnegative	2002	0	0	0	0	0	0	0	0	0	0	1	0	0	0	0	0	0	1	0	0
E833	Global 1983-2008 (Mentzer et al)	L18	CS28A	2003	1	0	0	0	0	0	0	0	0	0	0	1	0	1	0	1	0	0	0	1
E841	Global 1983-2008 (Mentzer et al)	NA	CFnegative	2003	0	0	0	0	0	0	0	0	0	0	0	0	0	0	0	0	0	0	0	0
E842	Global 1983-2008 (Mentzer et al)	L17	CS6	2003	0	0	0	0	0	0	0	0	0	0	0	0	0	0	0	0	0	0	0	0
E85	Bangladesh 1980-2011 (Mentzer et al)	L16	CS3+CS21	1987	0	0	0	0	0	0	0	0	0	0	0	0	0	0	0	0	0	0	0	0
E855	Global 1983-2008 (Mentzer et al)	L11+L13	CFnegative	2003	0	0	0	0	0	0	0	0	0	0	0	0	0	0	0	0	0	0	0	0
E856	Global 1983-2008 (Mentzer et al)	L9+L10+L21	CS6	2003	0	0	0	0	0	0	0	0	0	0	0	0	0	0	0	0	0	0	0	1
E858	Global 1983-2008 (Mentzer et al)	L3	CFA/I+CS21	2003	1	0	0	0	0	0	0	0	0	0	0	1	0	1	0	1	0	0	1	0
E860	Global 1983-2008 (Mentzer et al)	L9+L10+L21	CS6	2003	0	0	0	0	0	0	0	0	0	0	0	0	0	0	0	0	0	0	0	1
E863	Global 1983-2008 (Mentzer et al)	L1	CS1+CS3+CS21	2003	0	0	0	0	0	0	0	0	0	0	0	0	0	0	0	0	0	0	0	0
E865	Global 1983-2008 (Mentzer et al)	L5	CS5+CS6	2003	1	0	0	0	0	0	0	0	0	0	0	1	0	1	0	1	0	0	0	1
E867	Global 1983-2008 (Mentzer et al)	L5	CS5+CS6	2003	0	0	0	0	0	0	0	0	0	0	0	0	0	0	0	0	0	0	0	1
E87	Bangladesh 1980-2011 (Mentzer et al)	L2	CS2+CS3+CS21	1988	0	0	0	0	0	0	0	0	0	0	0	0	0	0	0	0	0	0	0	0
E871	Global 1983-2008 (Mentzer et al)	L9+L10+L21	CS6	2003	0	0	0	0	0	0	0	0	0	0	0	0	0	0	0	0	0	0	0	1
E873	Global 1983-2008 (Mentzer et al)	L20	CS30	2003	1	0	0	0	0	0	0	0	0	0	0	0	0	1	1	0	0	0	1	0
E876	Global 1983-2008 (Mentzer et al)	L9+L10+L21	CS20	2003	1	0	0	0	0	0	0	0	0	0	0	0	0	0	0	1	0	0	0	1
E877	Global 1983-2008 (Mentzer et al)	L5	CS5+CS6	2003	1	0	0	0	0	0	0	0	0	0	0	1	0	1	0	1	0	0	0	1
E879	Global 1983-2008 (Mentzer et al)	L9+L10+L21	CS6	2003	1	0	0	0	0	0	0	0	0	0	0	1	0	1	0	1	0	0	1	0
E88	Bangladesh 1980-2011 (Mentzer et al)	L5	CS5+CS6	1988	0	0	0	0	0	0	0	0	0	0	0	0	0	0	0	0	0	0	0	1
E881	Global 1983-2008 (Mentzer et al)	L20	CS13+CS30	2003	1	0	0	0	0	0	0	0	0	0	0	0	0	1	1	0	0	0	1	0
E882	Global 1983-2008 (Mentzer et al)	L11+L15	CS12	2003	1	0	0	0	0	0	0	0	0	0	0	1	0	1	0	1	0	0	0	1
E883	Global 1983-2008 (Mentzer et al)	L11+L13	CFnegative	2003	1	1	1	0	0	0	0	0	0	0	0	1	0	1	1	0	0	0	0	1
E884	Global 1983-2008 (Mentzer et al)	L19	CFnegative	2003	1	0	0	0	0	0	0	0	0	0	0	0	0	1	1	0	0	0	1	0
E885	Global 1983-2008 (Mentzer et al)	L18	CFnegative	2003	1	0	0	0	0	0	0	0	0	0	0	1	0	1	0	1	0	0	1	0
E887	Global 1983-2008 (Mentzer et al)	L19	CFnegative	2003	0	0	0	0	0	0	0	0	0	0	0	0	0	0	0	0	0	0	0	0
E888	Global 1983-2008 (Mentzer et al)	L20	CFnegative	2003	1	0	0	0	0	0	0	0	0	0	0	0	0	0	1	0	0	1	1	0
E890	Global 1983-2008 (Mentzer et al)	L24	CFnegative	2003	1	0	0	0	0	0	0	0	0	0	0	1	0	1	0	1	0	1	0	1
E891	Global 1983-2008 (Mentzer et al)	L19	CFnegative	2003	1	0	0	0	0	0	0	0	0	0	0	1	0	1	1	1	0	0	0	1
E892	Global 1983-2008 (Mentzer et al)	L19	CFnegative	2003	0	0	0	0	0	0	0	0	0	0	0	0	0	0	0	0	0	0	0	0

StrainID	Study Dataset	ETEC_Lineage	CFs	Year	ampicillin.TEM	macrolide.mrx	macrolide.mphA.E.G.	macrolide.msrE	ESBL.CTXM	ESBL.DHA1	ESBL.Carbapenum	Fluoroquinolone.QnrB	Fluoroquinolone.QnrS	Fluoroquinolone.gyrB	Fluoroquiolone.parC	strA.APH6	strB.APH3	Trimethoprim.dfr	sulphonamide.sul1	sulphonamide.sul2	sulphonamide.sul3	chloramphenicol.CAT	tetracyclin.tetA	tetracyclin.tetB
E895	Global 1983-2008 (Mentzer et al)	L17	CS6	2003	0	0	0	0	0	0	0	0	0	0	0	0	0	0	0	0	0	0	0	0
E897	Global 1983-2008 (Mentzer et al)	L6	CFA/I+CS21	2003	1	0	0	0	0	0	0	0	0	0	0	1	0	1	0	1	0	0	1	0
E898	Global 1983-2008 (Mentzer et al)	L11+L15	CS6	2003	0	0	0	0	0	0	0	0	0	0	0	1	0	0	0	0	0	0	1	0
E899	Global 1983-2008 (Mentzer et al)	L18	CFnegative	2003	1	0	0	0	0	0	0	0	0	0	0	1	1	1	0	1	0	0	0	1
E900	Global 1983-2008 (Mentzer et al)	L5	CS17	2003	1	0	0	0	0	0	0	0	0	0	0	1	0	1	0	1	0	0	0	1
E901	Global 1983-2008 (Mentzer et al)	L28	CS8	2003	1	0	0	0	0	0	0	0	0	0	0	1	0	1	0	1	0	0	0	1
E903	Global 1983-2008 (Mentzer et al)	L2	CS2+CS3+CS21	2003	1	0	0	0	0	0	0	0	0	0	0	1	0	1	0	1	0	0	0	1
E907	Global 1983-2008 (Mentzer et al)	L19	CS14	2004	0	0	0	0	0	0	0	0	0	0	0	0	0	0	0	0	0	0	0	1
E908	Global 1983-2008 (Mentzer et al)	L17	CFnegative	2004	1	0	1	0	0	0	0	0	0	0	0	1	0	1	1	1	0	0	0	1
E916	Global 1983-2008 (Mentzer et al)	L5	CS5+CS6	2003	1	0	0	0	0	0	0	0	0	0	0	1	0	0	0	1	0	0	0	1
E917	Global 1983-2008 (Mentzer et al)	L3	CFA/I+CS21	2003	1	0	0	0	0	0	0	0	0	0	0	1	0	1	0	1	0	0	0	1
E920	Global 1983-2008 (Mentzer et al)	L19	CS14	2003	0	0	0	0	0	0	0	0	0	0	0	1	0	1	0	1	0	0	0	1
E924	Global 1983-2008 (Mentzer et al)	L4	CS21	2003	1	0	0	0	0	0	0	0	0	0	0	0	0	0	0	0	0	0	0	0
E925	Global 1983-2008 (Mentzer et al)	L1	CS1+CS3+CS21	2003	0	0	0	0	0	0	0	0	0	0	0	0	0	0	0	0	0	0	0	0
E927	Global 1983-2008 (Mentzer et al)	L9+L10+L21	CS6	1997	1	0	0	0	0	0	0	0	0	0	0	1	0	1	0	1	0	0	0	0
E928	Global 1983-2008 (Mentzer et al)	L20	CS14	1997	1	0	0	0	0	0	0	0	0	0	0	1	0	1	1	1	0	0	1	0
E934	Global 1983-2008 (Mentzer et al)	L9+L10+L21	CS19	1997	1	0	0	0	0	0	0	0	0	0	0	1	0	1	0	1	0	0	0	1
E935	Global 1983-2008 (Mentzer et al)	L9+L10+L21	CS19	1997	0	0	0	0	0	0	0	0	0	0	0	0	0	0	0	0	0	0	0	0
E936	Global 1983-2008 (Mentzer et al)	L23	CFnegative	1997	1	0	0	0	0	0	0	0	0	0	0	1	0	0	0	1	0	0	0	1
E938	Global 1983-2008 (Mentzer et al)	L1	CS1+CS3+CS21	1997	0	0	0	0	0	0	0	0	0	0	0	1	0	0	0	1	0	0	0	0
E939	Global 1983-2008 (Mentzer et al)	L20	CS27A	1997	1	0	0	0	0	0	0	0	0	0	0	1	0	1	1	1	0	0	1	0
E940	Global 1983-2008 (Mentzer et al)	L9+L10+L21	CS6	1997	0	0	0	0	0	0	0	0	0	0	0	0	0	0	0	0	0	0	0	0
E941	Global 1983-2008 (Mentzer et al)	L11+L15	CFnegative	1997	0	0	0	0	0	0	0	0	0	0	0	1	0	1	1	1	0	1	0	0
E943	Global 1983-2008 (Mentzer et al)	L9+L10+L21	CS6	1997	1	0	0	0	0	0	0	0	0	0	0	1	0	1	0	1	0	0	0	0
E944	Global 1983-2008 (Mentzer et al)	L9+L10+L21	CS19	1997	0	0	0	0	0	0	0	0	0	0	0	1	0	0	0	1	0	0	0	0
E945	Global 1983-2008 (Mentzer et al)	L9+L10+L21	CS19	1997	0	0	0	0	0	0	0	0	0	0	0	1	0	0	0	1	0	0	0	0
E947	Global 1983-2008 (Mentzer et al)	L11+L15	CS27A	1997	0	0	0	0	0	0	0	0	0	0	0	1	0	1	0	1	0	0	0	0
E949	Global 1983-2008 (Mentzer et al)	L25	CFnegative	1997	1	0	0	0	0	0	0	0	0	0	0	1	0	1	1	1	0	0	0	0
E952	Global 1983-2008 (Mentzer et al)	L11+L13	CS13+CS26	1997	1	0	0	0	0	0	0	0	0	0	0	0	0	0	0	1	0	0	1	0
E953	Global 1983-2008 (Mentzer et al)	L1	CS1+CS3+CS21	1997	0	0	0	0	0	0	0	0	0	0	0	1	0	0	0	1	0	0	0	0
E955	Global 1983-2008 (Mentzer et al)	L9+L10+L21	CS19	1997	1	0	0	0	0	0	0	0	0	0	0	1	0	0	0	1	0	0	0	0
E956	Global 1983-2008 (Mentzer et al)	L25	CFnegative	1997	1	0	0	0	0	0	0	0	0	0	0	1	0	1	1	1	0	0	0	0
E957	Global 1983-2008 (Mentzer et al)	L25	CFnegative	1997	1	0	0	0	0	0	0	0	0	0	0	1	0	1	1	1	0	1	0	0
E97	Bangladesh 1980-2011 (Mentzer et al)	NA	CS6	1988	0	0	0	0	0	0	0	0	0	0	0	0	0	0	0	0	0	0	0	0
E978	Global 1983-2008 (Mentzer et al)	L9+L10+L21	CS19	1997	0	0	0	0	0	0	0	0	0	0	0	1	0	0	0	1	0	0	0	0
E986	Global 1983-2008 (Mentzer et al)	L6	CFA/I+CS21	1997	1	0	0	0	0	0	0	0	0	0	0	1	0	0	0	0	0	0	0	0
E99	Bangladesh 1980-2011 (Mentzer et al)	L2	CS2+CS21	1988	0	0	0	0	0	0	0	0	0	0	0	0	0	0	0	0	0	0	0	0
E995	Global 1983-2008 (Mentzer et al)	L1	CS1+CS3+CS21	1997	0	0	0	0	0	0	0	0	0	0	0	0	0	0	0	0	0	0	0	0
E996	Global 1983-2008 (Mentzer et al)	NA	CS6	1997	1	0	0	0	0	0	0	0	0	0	0	1	0	0	0	1	0	0	0	0
E998	Global 1983-2008 (Mentzer et al)	L23	CS27A	1997	0	0	0	0	0	0	0	0	0	0	0	1	0	0	0	0	0	0	0	0
GVE0025	Bangladesh 2022-2023 (This study)	L1	CS1+CS3+CS21	2023	0	1	1	0	1	0	0	0	1	0	0	0	0	0	0	0	0	0	0	0
GVE0026	Bangladesh 2022-2023 (This study)	L3	CFA/I+CS21	2023	1	1	1	0	1	0	0	0	1	0	0	0	0	1	1	0	0	0	0	1
GVE0027	Bangladesh 2022-2023 (This study)	L26	CFnegative	2023	0	1	1	0	0	1	0	1	0	0	0	0	0	1	1	0	0	0	0	0
GVE0028	Bangladesh 2022-2023 (This study)	L7	CS6	2023	1	1	1	0	0	0	0	0	0	0	0	0	0	0	0	0	0	0	0	1
GVE0029	Bangladesh 2022-2023 (This study)	L6	CFA/I+CS21	2023	0	0	0	0	1	0	0	0	1	0	0	1	0	1	1	1	0	0	1	0
GVE0030	Bangladesh 2022-2023 (This study)	L4	CS6+CS21	2023	0	0	0	0	0	0	0	0	0	0	0	0	0	0	0	0	0	0	0	0
GVE0031	Bangladesh 2022-2023 (This study)	L27	CFnegative	2023	0	0	0	0	1	0	0	0	1	0	0	0	0	0	0	0	0	0	0	0
GVE0032	Bangladesh 2022-2023 (This study)	L3	CS7	2023	1	1	1	0	0	0	0	0	0	0	0	0	0	0	0	0	0	0	0	0
GVE0033	Bangladesh 2022-2023 (This study)	L25	CFnegative	2023	1	1	1	1	0	1	0	1	0	0	0	0	0	0	1	0	0	0	0	0
GVE0034	Bangladesh 2022-2023 (This study)	L20	CFnegative	2023	1	1	1	1	1	0	0	0	1	0	0	0	0	0	0	0	0	0	1	0
GVE0035	Bangladesh 2022-2023 (This study)	NA	CFnegative	2023	1	1	1	0	0	0	0	0	0	0	0	0	0	1	0	0	0	0	0	0
GVE0036	Bangladesh 2022-2023 (This study)	L19	CS14	2023	0	0	0	0	0	0	0	0	0	0	0	0	0	0	0	0	0	0	0	0
GVE0037	Bangladesh 2022-2023 (This study)	L3	CS7	2023	1	1	1	0	0	0	0	0	0	0	0	0	0	0	0	0	0	0	0	0
GVE0038	Bangladesh 2022-2023 (This study)	L3	CFA/I+CS21	2023	1	1	1	0	1	0	0	0	1	0	0	0	0	1	0	0	0	0	0	1
GVE0039	Bangladesh 2022-2023 (This study)	L20	F41a	2023	0	1	1	0	0	1	0	1	0	0	0	0	0	0	1	0	0	0	0	0
GVE0040	Bangladesh 2022-2023 (This study)	L6	CFA/I+CS21	2023	1	1	1	0	0	0	0	0	0	0	0	0	0	0	0	0	0	0	0	0
GVE0041	Bangladesh 2022-2023 (This study)	L25	CFnegative	2023	1	1	1	1	0	0	0	0	1	0	0	0	0	0	0	0	0	0	1	0
GVE0042	Bangladesh 2022-2023 (This study)	L1	CS1+CS3+CS21	2023	0	0	0	0	0	0	0	0	0	0	0	0	0	0	0	0	0	0	0	0
GVE0043	Bangladesh 2022-2023 (This study)	L6	CFA/I+CS21	2023	1	1	1	0	0	0	0	0	0	0	0	0	0	0	0	0	0	0	0	0
GVE0044	Bangladesh 2022-2023 (This study)	NA	CS21	2023	1	1	1	0	0	0	0	0	0	0	0	0	0	0	0	0	0	0	0	0
GVE0045	Bangladesh 2022-2023 (This study)	L12+L14	CFnegative	2023	0	0	0	0	0	0	0	0	1	0	0	0	0	0	0	0	0	0	0	0
GVE0046	Bangladesh 2022-2023 (This study)	L6	CFA/I+CS21	2023	1	1	1	0	0	0	0	0	0	0	0	0	0	0	0	0	0	0	0	0
GVE0047	Bangladesh 2022-2023 (This study)	L1	CS1+CS3+CS21	2023	0	1	1	0	1	0	0	0	1	0	0	0	0	0	0	0	0	0	0	0
GVE0048	Bangladesh 2022-2023 (This study)	L3	CFA/I+CS21	2023	1	1	1	0	1	0	0	0	1	0	0	0	0	1	1	0	0	0	0	1
GVE0049	Bangladesh 2022-2023 (This study)	L5	CS5+CS6	2023	0	0	0	0	0	0	0	0	0	0	0	0	0	0	0	0	0	0	0	0
GVE0050	Bangladesh 2022-2023 (This study)	L25	CFnegative	2023	1	1	1	1	0	0	0	0	1	0	0	0	0	0	0	0	0	0	1	0
GVE0051	Bangladesh 2022-2023 (This study)	L19	CS23	2023	0	0	0	0	0	1	0	1	0	0	0	0	0	1	0	0	0	0	0	0
GVE0052	Bangladesh 2022-2023 (This study)	L26	CFnegative	2023	1	1	1	0	0	0	0	0	1	0	0	0	0	0	0	0	0	0	1	0
GVE0053	Bangladesh 2022-2023 (This study)	L29	CS12	2023	0	0	0	0	1	0	0	0	1	0	0	0	0	0	0	0	0	0	0	0
GVE0054	Bangladesh 2022-2023 (This study)	L20	CFnegative	2023	1	1	1	0	1	0	0	0	0	0	0	0	0	1	1	0	0	1	0	1
GVE0055	Bangladesh 2022-2023 (This study)	L6	CFA/I+CS21	2023	0	0	0	0	1	0	0	0	1	0	0	1	0	1	1	1	0	0	1	0
GVE0056	Bangladesh 2022-2023 (This study)	L5	CS5+CS6	2023	0	0	0	0	0	0	0	0	0	0	0	0	0	0	0	0	0	0	0	0
GVE0057	Bangladesh 2022-2023 (This study)	L7	CS6	2023	1	1	1	0	0	0	0	0	0	0	0	0	0	0	0	0	0	0	0	1
GVE0058	Bangladesh 2022-2023 (This study)	L26	CFnegative	2023	1	0	0	0	0	0	0	0	1	0	0	0	0	0	0	1	0	0	1	0
GVE0059	Bangladesh 2022-2023 (This study)	L19	CS23	2023	1	1	1	1	0	0	0	0	1	0	0	0	0	0	0	0	0	0	1	0
GVE0060	Bangladesh 2022-2023 (This study)	L6	CFA/I+CS21	2023	1	1	1	0	0	0	0	0	0	0	0	0	0	0	0	0	0	0	0	0
GVE0061	Bangladesh 2022-2023 (This study)	L11+L15	CFnegative	2023	1	1	1	0	0	0	0	0	0	0	0	0	0	0	0	0	0	0	0	0
GVE0062	Bangladesh 2022-2023 (This study)	L6	CFA/I+CS21	2023	0	0	0	0	1	0	0	0	1	0	0	1	0	1	1	1	0	0	1	0
GVE0063	Bangladesh 2022-2023 (This study)	L11+L15	CFnegative	2023	0	0	0	0	0	0	0	0	1	0	0	0	0	1	0	0	0	0	1	0
GVE0064	Bangladesh 2022-2023 (This study)	L3	CS7	2023	1	1	1	0	0	0	0	0	0	0	0	0	0	0	0	0	0	0	0	0
GVE0065	Bangladesh 2022-2023 (This study)	L18	CS6	2023	0	0	0	0	0	0	0	0	0	0	0	0	0	0	0	0	0	0	0	0
GVE0066	Bangladesh 2022-2023 (This study)	L20	CS12	2023	0	1	1	0	0	1	0	1	0	0	0	0	0	1	1	0	0	0	0	0
GVE0067	Bangladesh 2022-2023 (This study)	L4	CS6+CS8	2023	1	1	1	0	1	0	0	0	1	0	0	0	0	0	0	0	0	0	0	0
GVE0068	Bangladesh 2022-2023 (This study)	L5	CS5+CS6	2023	0	0	0	0	0	0	0	0	0	0	0	0	0	0	0	0	0	0	0	0
GVE0069	Bangladesh 2022-2023 (This study)	NA	CS21	2023	0	1	1	0	0	0	0	0	0	0	0	0	0	0	0	0	0	0	0	0
GVE0070	Bangladesh 2022-2023 (This study)	NA	CS6	2023	1	1	1	1	0	1	0	1	1	0	0	0	0	0	1	0	0	0	0	1

StrainID	Study Dataset	ETEC_Lineage	CFs	Year	ampicillin.TEM	macrolide.mrx	macrolide.mphA.E.G.	macrolide.msrE	ESBL.CTXM	ESBL.DHA1	ESBL.Carbapenum	Fluoroquinolone.QnrB	Fluoroquinolone.QnrS	Fluoroquinolone.gyrB	Fluoroquiolone.parC	strA.APH6	strB.APH3	Trimethoprim.dfr	sulphonamide.sul1	sulphonamide.sul2	sulphonamide.sul3	chloramphenicol.CAT	tetracyclin.tetA	tetracyclin.tetB
GVE0071	Bangladesh 2022-2023 (This study)	L6	CFA/I+CS21	2023	0	0	0	0	1	0	0	0	1	0	0	1	0	1	1	1	0	0	1	0
GVE0072	Bangladesh 2022-2023 (This study)	L25	CS23	2023	0	0	0	0	1	0	0	0	1	0	0	0	0	0	0	0	0	0	0	0
GVE0073	Bangladesh 2022-2023 (This study)	L5	CS5+CS6	2023	0	0	0	0	0	0	0	0	0	0	0	0	0	0	0	0	0	0	0	0
GVE0074	Bangladesh 2022-2023 (This study)	L20	CFnegative	2023	1	1	1	0	0	0	0	0	0	0	0	0	0	0	0	0	0	0	0	0
GVE0075	Bangladesh 2022-2023 (This study)	L5	CS5+CS6	2023	0	0	0	0	0	0	0	0	0	0	0	0	0	0	0	0	0	0	0	0
GVE0076	Bangladesh 2022-2023 (This study)	L5	CS5+CS6	2023	0	0	0	0	0	0	0	0	0	0	0	0	0	0	0	0	0	0	0	0
GVE0077	Bangladesh 2022-2023 (This study)	L3	CFA/I+CS21	2023	1	1	1	0	0	0	0	0	0	0	0	0	0	1	1	0	0	0	0	1
GVE0078	Bangladesh 2022-2023 (This study)	NA	CS6	2023	1	1	1	0	1	0	0	0	0	0	0	0	0	0	0	0	0	0	0	0
GVE0079	Bangladesh 2022-2023 (This study)	L11+L15	CFnegative	2023	0	0	0	0	0	0	0	0	1	0	0	0	0	1	0	0	0	0	1	0
GVE0080	Bangladesh 2022-2023 (This study)	L19	CS6+PCFO71	2023	0	1	1	0	1	1	0	1	1	0	0	0	0	1	1	0	0	0	0	0
GVE0081	Bangladesh 2022-2023 (This study)	L5	CS5+CS6	2023	0	0	0	0	0	0	0	0	0	0	0	0	0	0	0	0	0	0	0	0
GVE0082	Bangladesh 2022-2023 (This study)	L11+L15	CFnegative	2023	0	0	0	0	0	0	0	0	1	0	0	0	0	1	0	0	0	0	0	0
GVE0083	Bangladesh 2022-2023 (This study)	L6	CFA/I+CS21	2023	1	1	1	0	0	0	0	0	0	0	0	0	0	0	0	0	0	0	0	0
GVE0084	Bangladesh 2022-2023 (This study)	L3	CFA/I+CS21	2023	1	1	1	0	1	0	0	0	1	0	0	0	0	1	1	0	0	0	0	1
GVE0085	Bangladesh 2022-2023 (This study)	L26	CS23	2023	0	1	1	1	0	1	0	1	1	0	0	0	0	0	1	1	0	0	1	0
GVE0093	Bangladesh 2022-2023 (This study)	L1	CS1+CS3+CS21	2023	0	1	1	0	1	0	0	0	1	0	0	0	0	0	0	0	0	0	0	0
GVE0094	Bangladesh 2022-2023 (This study)	L11+L15	CFnegative	2023	0	0	0	0	1	0	0	0	1	0	0	0	0	0	0	0	0	1	0	1
GVE0095	Bangladesh 2022-2023 (This study)	L5	CS5+CS6	2023	0	0	0	0	0	0	0	0	0	0	0	0	0	0	0	0	0	0	0	0
GVE0096	Bangladesh 2022-2023 (This study)	L18	CS6	2023	1	1	1	0	0	0	0	0	1	0	0	0	0	1	1	0	0	0	0	0
GVE0097	Bangladesh 2022-2023 (This study)	NA	CFnegative	2023	0	1	1	0	0	1	0	1	0	0	0	0	0	1	1	0	0	0	0	0
GVE0098	Bangladesh 2022-2023 (This study)	L3	CFA/I+CS21	2023	1	1	1	0	1	0	0	0	1	0	0	0	0	1	1	0	0	0	0	1
GVE0099	Bangladesh 2022-2023 (This study)	L3	CFA/I+CS21	2023	1	1	1	0	1	0	0	0	1	0	0	0	0	1	1	0	0	0	0	1
GVE0100	Bangladesh 2022-2023 (This study)	L19	CFnegative	2023	0	0	0	0	1	0	0	0	1	0	0	0	0	0	0	0	0	0	0	0
GVE0101	Bangladesh 2022-2023 (This study)	L3	CFA/I+CS21	2023	0	1	1	0	1	0	0	0	1	0	0	0	0	0	1	0	0	0	0	1
GVE0102	Bangladesh 2022-2023 (This study)	L5	CS5+CS6	2023	0	0	0	0	0	0	0	0	0	0	0	0	0	0	0	0	0	0	0	0
GVE0103	Bangladesh 2022-2023 (This study)	L19	CS23	2023	1	1	1	1	1	0	0	0	1	0	0	0	0	0	0	0	0	0	1	0
GVE0104	Bangladesh 2022-2023 (This study)	L19	CS13	2023	0	1	1	0	0	1	0	1	0	0	0	0	0	1	1	0	0	0	0	0
GVE0105	Bangladesh 2022-2023 (This study)	L25	CS23	2023	0	0	0	0	1	0	0	0	1	0	0	0	0	0	0	0	0	0	1	0
GVE0106	Bangladesh 2022-2023 (This study)	NA	CS13	2023	1	1	1	0	0	0	0	0	0	0	0	1	0	0	0	1	0	0	0	0
GVE0107	Bangladesh 2022-2023 (This study)	NA	CS23	2023	1	1	1	0	0	0	0	0	0	0	0	0	0	1	1	0	0	1	0	0
GVE0108	Bangladesh 2022-2023 (This study)	L25	CS23	2023	0	0	0	0	1	0	0	0	1	0	0	0	0	0	0	0	0	0	0	0
GVE0109	Bangladesh 2022-2023 (This study)	L19	CFnegative	2023	0	1	1	0	0	1	0	1	0	0	0	0	0	1	1	0	0	0	0	0
GVE0110	Bangladesh 2022-2023 (This study)	L3	CS7	2023	1	1	1	0	0	0	0	0	0	0	0	0	0	0	0	0	0	0	0	0
GVE0111	Bangladesh 2022-2023 (This study)	NA	CFnegative	2023	1	1	1	1	1	1	0	1	0	0	1	1	0	1	1	1	0	0	0	1
GVE0112	Bangladesh 2022-2023 (This study)	L20	CFnegative	2023	0	1	1	0	1	0	0	0	1	0	0	0	0	0	0	0	0	0	0	0
GVE0126	Bangladesh 2022-2023 (This study)	NA	CS21	2023	0	1	1	0	0	0	0	0	0	0	0	0	0	0	0	0	0	0	0	0
GVE0127	Bangladesh 2022-2023 (This study)	L17	CS6	2023	0	1	1	0	0	1	0	1	0	0	0	0	0	1	1	0	0	0	0	0
GVE0128	Bangladesh 2022-2023 (This study)	L28	CS20	2023	0	1	1	0	0	1	0	1	0	0	0	0	0	1	1	0	0	0	0	0
GVE0129	Bangladesh 2022-2023 (This study)	L25	CFnegative	2023	0	0	0	0	0	0	0	0	0	0	0	0	0	0	0	0	0	0	0	0
GVE0130	Bangladesh 2022-2023 (This study)	L27	CFnegative	2023	0	0	0	0	1	0	0	0	1	0	0	0	0	0	0	0	0	0	0	0
GVE0131	Bangladesh 2022-2023 (This study)	L12+L14	CFnegative	2023	0	0	0	0	0	0	0	0	1	0	0	0	0	0	0	0	0	0	0	0
GVE0132	Bangladesh 2022-2023 (This study)	L5	CS5+CS6	2023	0	0	0	0	0	0	0	0	0	0	0	0	0	0	0	0	0	0	0	0
GVE0133	Bangladesh 2022-2023 (This study)	L25	CFnegative	2023	0	0	0	0	0	0	0	0	0	0	0	0	0	0	0	0	0	0	0	0
GVE0134	Bangladesh 2022-2023 (This study)	NA	CS23	2023	0	0	0	0	0	0	0	0	0	0	0	0	0	0	0	0	0	0	0	0
GVE0135	Bangladesh 2022-2023 (This study)	L28	CS20	2023	0	1	1	0	0	1	0	1	0	0	0	0	0	1	1	0	0	0	0	0
GVE0136	Bangladesh 2022-2023 (This study)	L19	CS14	2023	0	1	1	0	1	0	0	0	1	0	0	0	0	0	0	0	0	0	0	0
GVE0137	Bangladesh 2022-2023 (This study)	L4	CS6+CS21	2023	0	0	0	0	1	0	0	0	1	0	0	0	0	1	1	0	0	0	1	0
GVE0138	Bangladesh 2022-2023 (This study)	L19	CS23	2023	1	1	1	1	0	1	0	1	0	0	0	1	0	1	0	1	0	0	0	0
GVE0139	Bangladesh 2022-2023 (This study)	L5	CS17	2023	1	1	1	0	0	0	0	0	0	0	0	0	0	0	0	0	0	0	0	0
GVE0140	Bangladesh 2022-2023 (This study)	L26	CFnegative	2023	0	0	0	0	0	0	0	0	0	0	0	0	0	1	0	0	0	0	0	1
GVE0141	Bangladesh 2022-2023 (This study)	L3	CFA/I+CS21	2023	1	1	1	0	1	0	0	0	1	0	0	0	0	1	1	0	0	0	0	1
GVE0142	Bangladesh 2022-2023 (This study)	L18	CFnegative	2023	1	1	1	0	1	0	0	0	1	0	0	1	0	1	0	1	0	0	1	0
GVE0143	Bangladesh 2022-2023 (This study)	L4	CS21	2022	0	0	0	0	0	0	0	0	0	0	0	0	0	0	0	0	0	0	0	0
GVE0144	Bangladesh 2022-2023 (This study)	L20	CFnegative	2022	1	1	1	0	1	0	0	0	0	0	0	0	0	1	1	0	0	1	0	1
GVE0145	Bangladesh 2022-2023 (This study)	L27	CFnegative	2022	0	0	0	0	0	0	0	0	0	0	0	0	0	0	0	0	0	0	0	0
GVE0146	Bangladesh 2022-2023 (This study)	L5	CS5+CS6	2022	0	0	0	0	0	0	0	0	0	0	0	0	0	0	0	0	0	0	0	0
GVE0147	Bangladesh 2022-2023 (This study)	L11+L13	CFnegative	2022	0	1	1	0	1	0	0	0	1	0	0	0	0	1	0	0	0	0	1	0
GVE0148	Bangladesh 2022-2023 (This study)	L1	CS1+CS3+CS21	2022	0	0	0	0	0	0	0	0	0	0	0	0	0	0	0	0	0	0	0	0
GVE0149	Bangladesh 2022-2023 (This study)	L19	CFnegative	2022	0	0	0	0	1	0	0	0	1	0	0	0	0	0	0	0	0	0	1	0
GVE0150	Bangladesh 2022-2023 (This study)	L19	CFnegative	2022	0	0	0	0	1	0	0	0	1	0	0	0	0	0	0	0	0	0	1	0
GVE0151	Bangladesh 2022-2023 (This study)	L4	CS21	2022	0	0	0	0	0	0	0	0	0	0	0	0	0	0	0	0	0	0	0	0
GVE0152	Bangladesh 2022-2023 (This study)	L2	CS2+CS3+CS21	2022	0	1	1	0	1	0	0	0	1	0	0	0	0	1	1	0	0	0	0	0
GVE0153	Bangladesh 2022-2023 (This study)	L18	CS6	2022	1	1	1	0	0	0	0	0	0	0	0	0	0	0	0	0	0	0	0	0
GVE0154	Bangladesh 2022-2023 (This study)	L19	CS23	2022	1	0	0	0	0	0	0	0	1	0	0	0	0	1	0	1	0	0	1	0
GVE0155	Bangladesh 2022-2023 (This study)	L20	CFnegative	2022	1	1	1	1	1	0	0	0	1	0	0	0	0	0	0	0	0	0	1	0
GVE0156	Bangladesh 2022-2023 (This study)	L5	CS5+CS6	2022	0	0	0	0	0	0	0	0	0	0	0	0	0	0	0	0	0	0	0	0
GVE0157	Bangladesh 2022-2023 (This study)	L20	CFnegative	2022	1	1	1	1	0	0	0	0	1	0	0	0	0	0	0	0	0	0	1	0
GVE0158	Bangladesh 2022-2023 (This study)	L19	CS23	2022	1	0	0	0	0	0	0	0	1	0	0	0	0	1	0	1	0	0	1	0
GVE0159	Bangladesh 2022-2023 (This study)	L28	CS23	2022	1	1	1	0	0	0	0	0	0	0	0	0	0	0	0	0	0	0	0	0
GVE0160	Bangladesh 2022-2023 (This study)	L26	CFnegative	2022	1	0	0	0	1	0	0	0	1	0	0	0	0	0	0	0	0	0	1	0
GVE0161	Bangladesh 2022-2023 (This study)	NA	CFnegative	2022	1	0	0	0	0	0	0	0	1	0	0	0	0	0	0	0	0	0	1	0
GVE0162	Bangladesh 2022-2023 (This study)	L20	CFnegative	2022	1	1	1	0	0	0	0	0	1	0	0	0	0	1	1	0	0	0	1	0
GVE0163	Bangladesh 2022-2023 (This study)	L20	CFnegative	2022	0	0	0	0	1	0	0	0	1	0	0	0	0	0	0	0	0	0	0	0
GVE0164	Bangladesh 2022-2023 (This study)	NA	CS6	2022	1	1	1	0	1	0	0	0	0	0	0	0	0	0	0	0	0	0	0	0
GVE0165	Bangladesh 2022-2023 (This study)	L20	CFnegative	2022	0	1	1	0	0	1	0	1	0	0	0	0	0	1	1	0	0	0	0	0
GVE0166	Bangladesh 2022-2023 (This study)	L19	CS23	2022	0	0	0	0	1	0	0	0	1	0	0	0	0	0	0	0	0	0	0	0
GVE0167	Bangladesh 2022-2023 (This study)	L29	CS12	2022	0	0	0	0	1	0	0	0	1	0	0	0	0	0	0	0	0	0	0	0
GVE0168	Bangladesh 2022-2023 (This study)	L25	CS23	2022	0	0	0	0	1	0	0	0	1	0	0	0	0	0	0	0	0	0	1	0
GVE0169	Bangladesh 2022-2023 (This study)	L19	CS23	2022	1	1	1	1	0	1	0	1	0	0	0	1	0	1	0	1	0	0	0	0
GVE0170	Bangladesh 2022-2023 (This study)	L18	CS28B	2023	1	1	1	0	0	0	0	0	0	0	0	0	0	0	0	0	0	0	0	0
GVE0171	Bangladesh 2022-2023 (This study)	L27	CFnegative	2022	0	0	0	0	1	0	0	0	1	0	0	0	0	0	0	0	0	0	0	0
GVE0172	Bangladesh 2022-2023 (This study)	L4	CS6+CS21	2022	0	0	0	0	1	0	0	0	1	0	0	0	0	1	1	0	0	0	1	0
GVE0173	Bangladesh 2022-2023 (This study)	NA	CFnegative	2022	1	1	1	1	0	0	0	0	1	0	0	1	0	1	0	1	1	0	1	0
GVE0174	Bangladesh 2022-2023 (This study)	L5	CS5+CS6	2022	0	0	0	0	0	0	0	0	0	0	0	0	0	0	0	0	0	0	0	0
GVE0175	Bangladesh 2022-2023 (This study)	L6	CFA/I+CS21	2022	1	1	1	0	0	0	0	0	0	0	0	0	0	0	0	0	0	0	0	0
GVE0176	Bangladesh 2022-2023 (This study)	L19	CS13+CS23	2022	0	1	1	0	0	1	0	1	0	0	0	0	0	1	1	0	0	0	0	0

StrainID	Study Dataset	ETEC_Lineage	CFs	Year	ampicillin.TEM	macrolide.mrx	macrolide.mphA.E.G.	macrolide.msrE	ESBL.CTXM	ESBL.DHA1	ESBL.Carbapenum	Fluoroquinolone.QnrB	Fluoroquinolone.QnrS	Fluoroquinolone.gyrB	Fluoroquiolone.parC	strA.APH6	strB.APH3	Trimethoprim.dfr	sulphonamide.sul1	sulphonamide.sul2	sulphonamide.sul3	chloramphenicol.CAT	tetracyclin.tetA	tetracyclin.tetB
GVE0177	Bangladesh 2022-2023 (This study)	NA	CS2+CS3	2022	1	1	1	0	0	0	0	0	1	0	0	0	0	0	0	0	0	0	0	0
GVE0178	Bangladesh 2022-2023 (This study)	L20	CFnegative	2022	1	1	1	0	0	0	0	0	0	0	0	0	0	0	0	0	0	0	0	0
GVE0179	Bangladesh 2022-2023 (This study)	L26	CFnegative	2022	0	1	1	0	0	1	0	1	0	0	0	0	0	1	1	0	0	0	0	0
GVE0180	Bangladesh 2022-2023 (This study)	NA	CS23	2022	1	0	0	0	0	0	0	0	1	0	0	0	0	0	0	0	0	0	1	0
GVE0181	Bangladesh 2022-2023 (This study)	L20	CFnegative	2022	1	1	1	0	0	0	0	0	0	0	0	0	0	1	1	0	0	1	0	0
GVE0182	Bangladesh 2022-2023 (This study)	L26	CFnegative	2022	0	1	1	0	0	1	0	1	0	0	0	0	0	1	1	0	0	0	0	0
GVE0183	Bangladesh 2022-2023 (This study)	L5	CS5+CS6	2022	0	0	0	0	0	0	0	0	0	0	0	0	0	0	0	0	0	0	0	0
GVE0184	Bangladesh 2022-2023 (This study)	L19	CS23	2022	1	0	0	0	0	0	0	0	1	0	0	0	0	0	0	0	0	0	0	0
GVE0185	Bangladesh 2022-2023 (This study)	L5	CS5+CS6	2022	1	1	1	0	0	0	0	0	0	0	0	0	0	0	0	0	0	0	0	0
GVE0186	Bangladesh 2022-2023 (This study)	L19	CS23	2022	0	0	0	0	1	0	0	0	1	0	0	0	0	0	0	0	0	0	0	0
GVE0187	Bangladesh 2022-2023 (This study)	L19	CS27A	2022	0	1	1	0	0	1	0	1	0	0	0	0	0	1	1	0	0	0	0	0
GVE0188	Bangladesh 2022-2023 (This study)	L19	CS23	2022	0	0	0	0	1	0	0	0	1	0	0	0	0	0	0	0	0	0	0	0
GVE0189	Bangladesh 2022-2023 (This study)	L6	CFA/I+CS21	2022	1	1	1	0	0	0	0	0	0	0	0	0	0	0	0	0	0	0	0	0
GVE0190	Bangladesh 2022-2023 (This study)	L19	CFnegative	2022	1	0	0	0	0	0	0	0	1	0	0	0	0	1	0	0	0	0	1	0
GVE0191	Bangladesh 2022-2023 (This study)	L5	CFnegative	2022	1	1	1	0	0	0	0	0	0	0	0	0	0	0	0	0	0	0	0	0
GVE0192	Bangladesh 2022-2023 (This study)	NA	CS20	2022	1	1	1	0	0	0	0	0	0	0	0	0	0	0	0	0	0	0	0	0
GVE0193	Bangladesh 2022-2023 (This study)	L5	CS5+CS6	2022	0	0	0	0	0	0	0	0	0	0	0	0	0	0	0	0	0	0	0	0
GVE0194	Bangladesh 2022-2023 (This study)	L25	CS23	2022	0	0	0	0	1	0	0	0	1	0	0	0	0	0	0	0	0	0	1	0
GVE0195	Bangladesh 2022-2023 (This study)	L1	CS1+CS3+CS21	2022	1	1	1	0	0	0	0	0	0	0	0	0	0	0	0	0	0	0	0	0
GVE0196	Bangladesh 2022-2023 (This study)	L5	CS5+CS6	2022	0	0	0	0	0	0	0	0	0	0	0	0	0	0	0	0	0	0	0	0
GVE0197	Bangladesh 2022-2023 (This study)	L19	CS23	2022	1	0	0	0	1	0	0	0	1	0	0	0	0	0	0	0	0	0	0	0
GVE0198	Bangladesh 2022-2023 (This study)	L11+L13	CS23	2022	1	1	1	1	1	0	0	0	1	0	0	0	0	0	0	0	0	0	1	0
GVE0199	Bangladesh 2022-2023 (This study)	L19	CS14	2022	0	0	0	0	0	0	0	0	0	0	0	0	0	0	0	0	0	0	0	0
GVE0200	Bangladesh 2022-2023 (This study)	NA	CS23	2022	1	0	0	0	0	0	0	0	1	0	0	0	0	0	0	0	0	0	1	0
GVE0201	Bangladesh 2022-2023 (This study)	L5	CS5+CS6	2022	0	0	0	0	0	0	0	0	0	0	0	0	0	0	0	0	0	0	0	0
GVE0202	Bangladesh 2022-2023 (This study)	L25	CS23	2022	0	0	0	0	1	0	0	0	1	0	0	0	0	0	0	0	0	0	0	0
GVE0203	Bangladesh 2022-2023 (This study)	L5	CS5+CS6	2022	0	0	0	0	0	0	0	0	0	0	0	0	0	0	0	0	0	0	0	0
GVE0204	Bangladesh 2022-2023 (This study)	L19	CFnegative	2022	1	0	0	0	0	0	0	0	1	0	0	0	0	1	0	0	0	0	1	0
GVE0205	Bangladesh 2022-2023 (This study)	L27	CFnegative	2022	0	0	0	0	1	0	0	0	1	0	0	0	0	0	0	0	0	0	0	0
GVE0206	Bangladesh 2022-2023 (This study)	L20	CFnegative	2022	1	1	1	1	0	0	0	0	1	0	0	0	0	0	0	0	0	0	1	0
GVE0207	Bangladesh 2022-2023 (This study)	L3	CFA/I+CS21	2022	1	1	1	0	1	0	0	0	1	0	0	0	0	0	1	0	0	0	0	1
GVE0208	Bangladesh 2022-2023 (This study)	L27	CS23	2022	1	1	1	1	0	0	0	0	1	0	0	0	0	0	0	0	0	0	1	0
GVE0209	Bangladesh 2022-2023 (This study)	L3	CFA/I+CS21	2022	1	1	1	0	1	0	0	0	1	0	0	0	0	1	1	0	0	0	0	1
GVE0210	Bangladesh 2022-2023 (This study)	L1	CS1+CS3+CS21	2022	1	1	1	0	0	0	0	0	0	0	0	0	0	0	0	0	0	0	0	0
GVE0211	Bangladesh 2022-2023 (This study)	L3	CFA/I	2022	1	0	1	0	1	0	0	0	0	0	0	0	0	1	0	0	0	0	0	0
GVE0212	Bangladesh 2022-2023 (This study)	NA	CS6	2022	1	1	1	0	0	1	0	1	0	0	0	0	0	1	1	0	0	0	0	0
GVE0213	Bangladesh 2022-2023 (This study)	L2	CS2+CS3+CS21	2022	0	0	0	0	1	0	0	0	0	0	0	0	0	0	0	0	0	0	0	0
GVE0214	Bangladesh 2022-2023 (This study)	L20	CFnegative	2022	0	1	1	0	0	0	0	0	1	0	0	0	0	0	0	0	0	0	1	0
GVE0215	Bangladesh 2022-2023 (This study)	L2	CS2+CS3+CS21	2022	0	0	0	0	1	0	0	0	0	0	0	0	0	0	0	0	0	0	0	0
GVE0216	Bangladesh 2022-2023 (This study)	L18	CS6	2022	0	0	0	0	0	0	0	0	0	0	0	0	0	0	0	0	0	0	0	0
GVE0217	Bangladesh 2022-2023 (This study)	L17	CS6	2022	0	1	1	0	0	1	0	1	0	0	0	0	0	1	1	0	0	0	0	0
GVE0218	Bangladesh 2022-2023 (This study)	L28	CS23	2022	1	1	1	0	0	0	0	0	0	0	0	0	0	0	0	0	0	0	0	0
GVE0219	Bangladesh 2022-2023 (This study)	L19	CS23	2022	1	1	1	0	0	0	0	0	0	0	0	0	0	0	0	0	0	0	0	0
GVE0220	Bangladesh 2022-2023 (This study)	L11+L13	CFnegative	2022	1	1	1	1	1	0	0	0	1	0	0	0	0	0	0	0	0	0	1	0
GVE0221	Bangladesh 2022-2023 (This study)	L20	CFnegative	2022	0	0	0	0	1	1	0	0	1	1	0	0	0	0	0	0	0	1	0	0
GVE0222	Bangladesh 2022-2023 (This study)	NA	CS21	2022	1	1	1	0	0	0	0	0	0	0	0	0	0	1	1	0	0	0	0	0
GVE0223	Bangladesh 2022-2023 (This study)	L20	CFnegative	2022	1	1	1	1	0	1	0	1	0	0	0	0	0	1	1	0	0	0	1	1
GVE0224	Bangladesh 2022-2023 (This study)	L5	CS5+CS6	2022	0	0	0	0	0	0	0	0	0	0	0	0	0	0	0	0	0	0	0	0
GVE0225	Bangladesh 2022-2023 (This study)	L11+L15	CFnegative	2022	0	0	0	0	0	0	0	0	0	0	0	0	0	0	0	0	0	1	0	0
GVE0226	Bangladesh 2022-2023 (This study)	L27	CS23	2022	0	0	0	0	1	0	0	0	0	0	0	0	0	0	0	0	0	0	0	0
GVE0227	Bangladesh 2022-2023 (This study)	L25	CFnegative	2022	1	0	0	0	0	0	0	0	1	0	0	0	0	0	0	0	1	0	1	0
GVE0248	Bangladesh 2022-2023 (This study)	L26	CFnegative	2022	1	1	1	1	0	0	0	0	1	0	0	0	0	0	0	0	0	0	1	0
GVE0249	Bangladesh 2022-2023 (This study)	L5	CS17	2022	1	1	1	0	0	0	0	0	0	0	0	0	0	0	0	0	0	0	0	0
GVE0250	Bangladesh 2022-2023 (This study)	L20	CFnegative	2022	0	0	0	0	1	0	0	0	1	0	0	0	0	0	0	0	0	0	0	0
GVE0251	Bangladesh 2022-2023 (This study)	L25	CS23	2022	0	0	0	0	1	0	0	0	1	0	0	0	0	0	0	0	0	0	0	0
GVE0252	Bangladesh 2022-2023 (This study)	L26	CFnegative	2022	1	1	1	1	0	0	0	0	1	0	0	0	0	0	0	0	0	0	1	0
GVE0253	Bangladesh 2022-2023 (This study)	L11+L15	CS27A	2022	0	1	1	0	1	0	0	0	0	0	0	0	0	1	1	0	0	0	0	1
GVE0254	Bangladesh 2022-2023 (This study)	L27	CFnegative	2022	0	0	0	0	1	0	0	0	1	0	0	0	0	0	0	0	0	0	0	0
GVE0255	Bangladesh 2022-2023 (This study)	L25	CFnegative	2022	0	1	1	1	0	1	0	1	1	0	0	0	0	0	1	0	0	0	0	0
GVE0256	Bangladesh 2022-2023 (This study)	L5	CS5+CS6	2022	0	0	0	0	0	0	0	0	0	0	0	0	0	0	0	0	0	0	0	0
GVE0257	Bangladesh 2022-2023 (This study)	L4	CS6+CS21	2022	0	0	0	0	1	0	0	0	0	0	0	0	0	1	1	0	0	0	1	0
GVE0258	Bangladesh 2022-2023 (This study)	L8	CS6+CS21	2022	0	0	0	0	0	0	0	0	0	0	0	0	0	0	0	0	0	0	0	0
GVE0259	Bangladesh 2022-2023 (This study)	L20	CFnegative	2022	1	1	1	1	1	0	0	0	1	0	0	0	0	0	0	0	0	0	1	0
GVE0260	Bangladesh 2022-2023 (This study)	L26	CFnegative	2022	0	1	1	0	0	1	0	1	0	0	0	0	0	1	1	0	0	0	0	0
GVE0261	Bangladesh 2022-2023 (This study)	L18	CFnegative	2022	1	1	1	0	1	0	0	0	1	0	0	1	0	1	0	1	0	0	1	0
GVE0262	Bangladesh 2022-2023 (This study)	L26	CFnegative	2022	1	1	1	1	0	0	0	0	1	0	0	0	0	0	0	0	0	0	1	0
GVE0263	Bangladesh 2022-2023 (This study)	L19	CFnegative	2022	1	0	0	0	0	0	0	0	1	0	0	0	0	0	0	0	0	0	1	0
GVE0264	Bangladesh 2022-2023 (This study)	NA	CFnegative	2022	1	1	1	0	1	0	0	0	1	0	0	0	0	0	0	0	0	0	0	0
GVE0265	Bangladesh 2022-2023 (This study)	L20	CFnegative	2022	1	1	1	0	1	0	0	0	1	0	0	0	0	0	0	0	0	0	0	0
GVE0266	Bangladesh 2022-2023 (This study)	L25	CFnegative	2022	1	1	1	1	0	1	0	1	1	0	0	0	0	0	1	0	0	0	0	0
GVE0267	Bangladesh 2022-2023 (This study)	L29	CS12	2022	0	0	0	0	1	0	0	0	1	0	0	0	0	0	0	0	0	0	0	0
GVE0268	Bangladesh 2022-2023 (This study)	L6	CFA/I+CS21	2022	1	1	1	0	0	0	0	0	0	0	0	0	0	0	0	0	0	0	0	0
GVE0269	Bangladesh 2022-2023 (This study)	L3	CFA/I+CS21	2022	0	1	1	0	1	0	0	0	1	0	0	0	0	0	1	0	0	0	0	1
GVE0270	Bangladesh 2022-2023 (This study)	L18	CS28A	2022	0	1	1	0	0	1	0	1	0	0	0	0	0	1	1	0	0	0	0	0
GVE0271	Bangladesh 2022-2023 (This study)	L4	CS6+CS21	2022	0	0	0	0	1	0	0	0	1	0	0	0	0	1	1	0	0	0	1	0
GVE0272	Bangladesh 2022-2023 (This study)	L19	CS14	2022	0	0	0	0	1	0	0	0	1	0	0	0	0	0	0	0	0	0	0	0
GVE0273	Bangladesh 2022-2023 (This study)	L3	CFA/I+CS21	2022	1	1	1	0	1	0	0	0	1	0	0	0	0	1	1	0	0	0	0	1
GVE0274	Bangladesh 2022-2023 (This study)	L20	CFnegative	2022	1	1	1	0	1	0	0	0	1	0	0	0	0	0	0	0	0	0	0	0
GVE0275	Bangladesh 2022-2023 (This study)	L25	CS23	2022	0	0	0	0	1	0	0	0	1	0	0	0	0	0	0	0	0	0	0	0
GVE0276	Bangladesh 2022-2023 (This study)	L4	CS6+CS21	2022	1	1	1	0	0	0	0	0	1	0	0	0	0	0	0	0	0	0	0	0
GVE0277	Bangladesh 2022-2023 (This study)	L6	CFA/I+CS21	2022	1	1	1	0	0	0	0	0	0	0	0	0	0	0	0	0	0	0	0	0
GVE0278	Bangladesh 2022-2023 (This study)	L19	CS27B	2022	1	1	1	0	0	1	0	1	0	0	0	1	0	1	1	1	0	0	0	0
GVE0279	Bangladesh 2022-2023 (This study)	L20	CS27B	2022	1	1	1	0	1	1	0	1	1	0	0	1	0	1	1	1	0	0	1	0
GVE0280	Bangladesh 2022-2023 (This study)	L27	CS23	2022	0	0	0	0	1	0	0	0	0	0	0	0	0	0	0	0	0	0	0	0
GVE0281	Bangladesh 2022-2023 (This study)	L6	CFA/I+CS21	2022	1	1	1	0	0	0	0	0	0	0	0	0	0	0	0	0	0	0	0	0
GVE0282	Bangladesh 2022-2023 (This study)	NA	CS21	2022	1	1	1	0	0	0	0	0	0	0	0	0	0	0	0	0	0	0	0	0

StrainID	Study Dataset	ETEC_Lineage	CFs	Year	ampicillin.TEM	macrolide.mrx	macrolide.mphA.E.G.	macrolide.msrE	ESBL.CTXM	ESBL.DHA1	ESBL.Carbapenum	Fluoroquinolone.QnrB	Fluoroquinolone.QnrS	Fluoroquinolone.gyrB	Fluoroquiolone.parC	strA.APH6	strB.APH3	Trimethoprim.dfr	sulphonamide.sul1	sulphonamide.sul2	sulphonamide.sul3	chloramphenicol.CAT	tetracyclin.tetA	tetracyclin.tetB
GVE0283	Bangladesh 2022-2023 (This study)	L26	CFnegative	2022	1	1	1	1	0	0	0	0	1	0	0	0	0	0	0	0	0	0	1	0
GVE0284	Bangladesh 2022-2023 (This study)	L19	CS6	2022	1	1	1	0	1	0	0	0	1	0	0	0	0	0	0	0	0	0	0	0
GVE0285	Bangladesh 2022-2023 (This study)	L20	CFnegative	2022	0	0	0	0	0	0	0	0	0	0	0	0	0	0	0	0	0	0	0	0
GVE0286	Bangladesh 2022-2023 (This study)	L26	CFnegative	2022	1	1	1	1	0	0	0	0	1	0	0	0	0	0	0	0	0	0	1	0
GVE0287	Bangladesh 2022-2023 (This study)	L19	CS14	2022	0	0	0	0	1	0	0	0	1	0	0	0	0	0	0	0	0	0	0	0
GVE0288	Bangladesh 2022-2023 (This study)	L26	CFnegative	2022	1	1	1	1	0	0	0	0	1	0	0	0	0	0	0	0	0	0	1	0
GVE0289	Bangladesh 2022-2023 (This study)	L17	CS6	2022	1	1	1	0	0	1	0	1	0	0	0	0	0	1	1	0	0	0	0	0
GVE0294	Bangladesh 2022-2023 (This study)	L9+L10+L21	CS13	2022	1	1	1	0	0	0	0	0	0	0	0	0	0	0	0	0	0	0	0	0
GVE0301	Bangladesh 2022-2023 (This study)	NA	CS20	2022	1	1	1	0	0	0	0	0	0	0	0	0	0	0	0	0	0	0	0	0
GVE0302	Bangladesh 2022-2023 (This study)	L26	CFnegative	2022	1	0	0	0	0	0	0	0	1	0	0	0	0	0	0	0	0	0	1	0
GVE0304	Bangladesh 2022-2023 (This study)	L19	CFnegative	2022	1	0	0	0	0	0	0	0	1	0	0	0	0	1	0	0	0	0	1	0
GVE0305	Bangladesh 2022-2023 (This study)	L26	CFnegative	2022	1	1	1	1	0	0	0	0	1	0	0	0	0	0	0	0	0	0	1	0
GVE0306	Bangladesh 2022-2023 (This study)	NA	CS21	2022	1	1	1	0	0	0	0	0	0	0	0	0	0	0	0	0	0	0	0	0
GVE0307	Bangladesh 2022-2023 (This study)	L19	CS13+CS27A	2022	0	1	1	0	0	1	0	1	0	0	0	0	0	1	1	0	0	0	0	0
GVE0309	Bangladesh 2012-2021 (This study)	L27	CFnegative	2013	1	1	1	0	0	0	0	0	0	0	0	0	0	0	0	0	0	0	0	0
GVE0310	Bangladesh 2012-2021 (This study)	L16	CFnegative	2015	0	1	1	0	1	0	0	0	0	0	1	0	0	0	0	0	0	0	0	0
GVE0311	Bangladesh 2022-2023 (This study)	L11+L13	CFnegative	2022	1	1	1	1	1	0	0	0	1	0	0	0	0	0	0	0	0	0	1	0
GVE0312	Bangladesh 2022-2023 (This study)	L1	CS1+CS3+CS21	2022	0	1	1	0	1	0	0	0	1	0	0	0	0	0	0	0	0	0	0	0
GVE0313	Bangladesh 2012-2021 (This study)	L20	CFnegative	2016	1	1	1	1	1	0	0	0	1	0	0	0	0	0	0	0	0	0	1	0
GVE0314	Bangladesh 2022-2023 (This study)	L18	F41a	2022	0	0	0	0	0	0	0	0	0	0	0	0	0	0	0	0	0	0	0	1
GVE0315	Bangladesh 2022-2023 (This study)	NA	CS6	2022	1	1	1	1	0	0	0	0	1	0	0	0	0	0	0	0	0	0	0	1
GVE0316	Bangladesh 2022-2023 (This study)	L3	CS7	2022	1	1	1	0	0	0	0	0	0	0	0	0	0	0	0	0	0	0	0	0
GVE0317	Bangladesh 2022-2023 (This study)	L4	CS6+CS21	2022	0	0	0	0	1	0	0	0	1	0	0	0	0	0	0	0	0	0	0	0
GVE0318	Bangladesh 2022-2023 (This study)	L29	CS12	2022	0	0	0	0	1	0	0	0	1	0	0	0	0	0	0	0	0	0	0	0
GVE0319	Bangladesh 2022-2023 (This study)	L20	CS14	2022	1	0	0	0	1	0	0	0	1	0	0	0	0	0	0	0	0	0	0	0
GVE0320	Bangladesh 2022-2023 (This study)	L4	CS6+CS8	2022	1	1	1	0	0	0	0	0	0	0	0	0	0	0	0	0	0	0	0	0
GVE0321	Bangladesh 2012-2021 (This study)	L20	CFnegative	2016	1	1	1	0	1	0	0	0	0	0	0	0	0	1	1	0	0	1	0	1
GVE0322	Bangladesh 2022-2023 (This study)	L6	CFA/I+CS21	2022	0	0	0	0	1	0	0	0	1	0	0	1	0	1	1	1	0	0	1	0
GVE0323	Bangladesh 2012-2021 (This study)	L11+L13	CFnegative	2017	1	1	1	0	1	0	0	0	1	0	0	0	0	0	0	0	0	0	0	0
GVE0324	Bangladesh 2022-2023 (This study)	L3	CS7	2022	1	1	1	0	1	0	0	0	0	0	0	0	0	0	0	0	0	0	0	0
GVE0325	Bangladesh 2022-2023 (This study)	L29	1+CS3+CS12+CS	2022	0	0	0	0	1	0	0	0	1	0	0	0	0	0	0	0	0	0	0	0
GVE0326	Bangladesh 2012-2021 (This study)	L20	CFnegative	2013	1	1	1	1	1	0	0	0	1	0	0	0	0	0	0	0	0	0	1	0
GVE0327	Bangladesh 2022-2023 (This study)	L29	CS23	2022	1	0	0	0	0	0	0	0	0	0	0	0	0	0	0	0	0	0	0	0
GVE0328	Bangladesh 2022-2023 (This study)	L19	CS23	2022	0	0	0	0	1	0	0	0	1	0	0	0	0	0	0	0	0	0	0	0
GVE0329	Bangladesh 2022-2023 (This study)	L4	CS6+CS8	2022	1	1	1	0	0	0	0	0	0	0	0	0	0	0	0	0	0	0	0	0
GVE0330	Bangladesh 2022-2023 (This study)	L19	CS23	2022	0	0	0	0	1	0	0	0	1	0	0	0	0	0	0	0	0	0	0	0
GVE0331	Bangladesh 2022-2023 (This study)	NA	CS21	2022	0	0	0	0	0	0	0	0	1	0	0	0	0	0	0	0	0	0	0	0
GVE0332	Bangladesh 2022-2023 (This study)	NA	CS20	2022	1	1	1	0	0	0	0	0	0	0	0	0	0	0	0	0	0	0	0	0
GVE0333	Bangladesh 2022-2023 (This study)	L5	CS5+CS6	2022	1	1	1	0	0	0	0	0	0	0	0	0	0	0	0	0	0	0	0	0
GVE0334	Bangladesh 2022-2023 (This study)	L5	CS5+CS6	2022	0	0	0	0	0	0	0	0	0	0	0	0	0	0	0	0	0	0	0	0
GVE0335	Bangladesh 2022-2023 (This study)	L26	CS23	2022	1	1	1	1	1	0	0	0	1	0	0	0	0	0	0	0	0	0	1	0
GVE0336	Bangladesh 2022-2023 (This study)	L18	CS6	2022	1	1	1	0	0	0	0	0	0	0	0	0	0	0	0	0	0	0	0	0
GVE0337	Bangladesh 2012-2021 (This study)	NA	CFnegative	2017	1	0	0	0	0	0	0	0	1	0	0	0	0	0	0	0	0	0	1	0
GVE0338	Bangladesh 2022-2023 (This study)	L1	CS1+CS3+CS21	2022	1	1	1	1	0	1	0	1	0	0	0	0	0	0	1	0	0	0	0	0
GVE0340	Bangladesh 2022-2023 (This study)	L19	CS23	2022	0	0	0	0	1	0	0	0	1	0	0	0	0	0	0	0	0	0	0	0
GVE0341	Bangladesh 2022-2023 (This study)	L11+L13	CS23	2022	1	1	1	0	1	0	0	0	1	0	0	0	0	0	0	0	0	0	1	0
GVE0342	Bangladesh 2022-2023 (This study)	L17	CS6	2022	0	1	1	0	0	1	0	1	0	0	0	0	0	1	1	0	0	0	0	0
GVE0343	Bangladesh 2022-2023 (This study)	L4	CS6+CS21	2022	1	1	1	0	0	0	0	0	1	0	0	0	0	0	0	0	0	0	0	0
GVE0344	Bangladesh 2022-2023 (This study)	L2	CS2+CS3+CS21	2022	0	1	1	0	1	0	0	0	1	0	0	0	0	1	1	0	0	0	0	0
GVE0345	Bangladesh 2022-2023 (This study)	L29	CS12	2022	0	0	0	0	1	0	0	0	1	0	0	0	0	0	0	0	0	0	0	0
GVE0346	Bangladesh 2012-2021 (This study)	L18	CFnegative	2012	1	1	1	0	1	0	0	0	1	0	0	1	0	1	0	1	0	0	1	0
GVE0347	Bangladesh 2022-2023 (This study)	NA	CS17	2022	1	1	1	0	0	0	0	0	0	0	0	0	0	0	0	0	0	0	0	0
GVE0348	Bangladesh 2022-2023 (This study)	L29	CS12	2022	0	0	0	0	1	0	0	0	1	0	0	0	0	0	0	0	0	0	0	0
GVE0349	Bangladesh 2012-2021 (This study)	L26	CFnegative	2017	1	1	1	1	0	0	0	0	1	0	0	0	0	0	0	0	0	0	1	0
GVE0350	Bangladesh 2012-2021 (This study)	L11+L15	CFnegative	2015	0	0	0	0	0	0	0	0	0	0	0	0	0	0	0	0	0	0	0	0
GVE0351	Bangladesh 2022-2023 (This study)	NA	CFA/I+CS21	2022	1	1	1	0	1	1	0	1	0	0	0	1	0	1	1	1	0	0	1	0
GVE0352	Bangladesh 2022-2023 (This study)	L29	CS12	2022	0	0	0	0	1	0	0	0	1	0	0	0	0	0	0	0	0	0	0	0
GVE0353	Bangladesh 2012-2021 (This study)	L25	CFnegative	2017	0	0	0	0	0	0	0	0	0	0	0	0	0	0	0	0	0	0	0	0
GVE0354	Bangladesh 2022-2023 (This study)	L3	CFA/I+CS21	2022	0	1	1	0	1	0	0	0	1	0	0	0	0	1	1	0	0	0	0	1
GVE0355	Bangladesh 2012-2021 (This study)	L17	CFnegative	2013	1	1	1	0	0	0	0	0	0	0	0	0	0	1	1	0	0	0	0	0
GVE0356	Bangladesh 2022-2023 (This study)	L7	CS6	2022	0	0	0	0	0	0	0	0	0	0	0	0	0	0	0	0	0	0	0	1
GVE0357	Bangladesh 2012-2021 (This study)	L20	CFnegative	2012	1	1	1	1	0	1	0	1	1	0	0	0	0	1	1	0	0	0	1	0
GVE0358	Bangladesh 2022-2023 (This study)	L29	CS6	2022	1	1	1	0	1	0	0	0	1	0	0	0	0	1	0	0	0	0	0	0
GVE0359	Bangladesh 2012-2021 (This study)	NA	CFnegative	2013	0	1	1	0	0	1	0	1	0	0	0	0	0	1	1	0	0	0	0	0
GVE0360	Bangladesh 2022-2023 (This study)	L20	CFnegative	2022	1	1	1	0	0	0	0	0	0	0	0	0	0	1	1	0	0	1	0	0
GVE0361	Bangladesh 2022-2023 (This study)	L19	CS6	2022	0	1	1	0	1	0	0	0	1	0	0	0	0	1	0	0	0	0	0	0
GVE0363	Bangladesh 2022-2023 (This study)	L3	CFA/I+CS21	2022	0	1	1	0	1	0	0	0	1	0	0	0	0	0	1	0	0	0	0	1
GVE0364	Bangladesh 2022-2023 (This study)	L5	CS5+CS6	2022	0	0	0	0	0	0	0	0	0	0	0	0	0	0	0	0	0	0	0	0
GVE0365	Bangladesh 2022-2023 (This study)	L19	CS13+CS27A	2022	0	1	1	0	0	1	0	1	0	0	0	0	0	1	1	0	0	0	0	0
GVE0366	Bangladesh 2022-2023 (This study)	L19	CS23	2022	1	1	1	1	0	1	0	1	1	0	0	1	0	1	0	1	0	0	0	1
GVE0367	Bangladesh 2022-2023 (This study)	NA	CS28A	2022	1	1	1	1	1	1	0	1	0	0	0	0	0	1	1	0	0	0	0	0
GVE0368	Bangladesh 2022-2023 (This study)	L2	CS2+CS3+CS21	2022	0	0	0	0	1	0	0	0	0	0	0	0	0	0	0	0	0	0	0	0
GVE0369	Bangladesh 2022-2023 (This study)	NA	CFnegative	2022	0	1	1	1	1	1	0	1	0	0	0	0	0	1	1	0	0	1	0	1
GVE0370	Bangladesh 2012-2021 (This study)	NA	CFnegative	2020	0	1	1	0	1	0	0	0	1	0	0	0	0	1	0	1	0	0	0	0
GVE0398	Bangladesh 2022-2023 (This study)	L20	CS12	2023	0	1	1	0	0	1	0	1	0	0	0	0	0	1	1	0	0	0	0	0
GVE0400	Bangladesh 2022-2023 (This study)	L20	CFnegative	2023	1	1	1	0	0	1	0	1	1	0	0	0	0	1	1	1	0	0	1	0
GVE0401	Bangladesh 2022-2023 (This study)	L28	CS2+CS3+CS21	2023	0	1	1	0	1	1	0	1	1	0	0	0	0	1	1	0	0	0	0	0
GVE0402	Bangladesh 2022-2023 (This study)	L2	CS2+CS3+CS21	2023	0	1	1	0	1	0	0	0	1	0	0	0	0	1	1	0	0	0	0	0
GVE0403	Bangladesh 2022-2023 (This study)	L1	CS1+CS3+CS21	2023	0	0	0	0	0	0	0	0	0	0	0	0	0	0	0	0	0	0	0	0
GVE0404	Bangladesh 2022-2023 (This study)	L11+L13	CFnegative	2023	0	1	1	0	1	0	0	0	1	0	0	0	0	0	0	0	0	0	0	0
GVE0405	Bangladesh 2022-2023 (This study)	L7	CS6	2023	0	1	1	0	1	0	0	0	1	0	0	0	0	0	0	0	0	0	0	1
GVE0407	Bangladesh 2022-2023 (This study)	L19	CS23	2023	1	1	1	1	0	0	0	0	1	0	0	0	0	0	0	0	0	0	1	0
GVE0425	Bangladesh 2022-2023 (This study)	L5	CS5+CS6	2023	0	0	0	0	0	0	0	0	0	0	0	0	0	0	0	0	0	0	0	0
GVE0426	Bangladesh 2022-2023 (This study)	L5	CS17	2023	0	1	1	0	0	0	0	0	0	0	0	0	0	0	0	0	0	0	0	0
GVE0427	Bangladesh 2022-2023 (This study)	L3	CFA/I+CS21	2023	0	1	1	0	1	1	0	1	1	0	0	0	0	1	1	0	0	0	0	1
GVE0429	Bangladesh 2022-2023 (This study)	L20	CS1+CS3+CS21	2023	0	1	1	0	1	0	0	0	1	0	0	0	0	0	0	0	0	0	0	0

StrainID	Study Dataset	ETEC_Lineage	CFs	Year	ampicillin.TEM	macrolide.mrx	macrolide.mphA.E.G.	macrolide.msrE	ESBL.CTXM	ESBL.DHA1	ESBL.Carbapenum	Fluoroquinolone.QnrB	Fluoroquinolone.QnrS	Fluoroquinolone.gyrB	Fluoroquiolone.parC	strA.APH6	strB.APH3	Trimethoprim.dfr	sulphonamide.sul1	sulphonamide.sul2	sulphonamide.sul3	chloramphenicol.CAT	tetracyclin.tetA	tetracyclin.tetB
GVE0433	Bangladesh 2022-2023 (This study)	L5	CS5+CS6	2023	0	0	0	0	0	0	0	0	0	0	0	0	0	0	0	0	0	0	0	0
GVE0434	Bangladesh 2022-2023 (This study)	L6	CFA/I+CS21	2023	1	1	1	0	0	0	0	0	0	0	0	0	0	0	0	0	0	0	0	0
GVE0436	Bangladesh 2022-2023 (This study)	NA	CFnegative	2023	0	1	1	0	1	1	0	1	1	0	0	0	0	1	1	0	0	0	0	0
GVE0437	Bangladesh 2022-2023 (This study)	L11+L13	CS6	2023	1	1	1	0	1	0	0	0	0	0	0	0	0	1	1	0	0	1	0	0
GVE0441	Bangladesh 2012-2021 (This study)	L19	CS23	2021	0	1	1	1	0	1	0	1	0	0	0	0	0	1	0	0	0	0	0	0
GVE0443	Bangladesh 2012-2021 (This study)	L11+L15	CFnegative	2021	1	1	1	0	0	0	0	0	1	0	0	1	0	1	0	1	0	0	0	1
GVE0445	Bangladesh 2012-2021 (This study)	L19	CS14	2021	1	1	1	0	1	0	0	0	1	0	0	1	0	0	0	1	0	0	1	0
GVE0446	Bangladesh 2012-2021 (This study)	L25	CS23	2021	0	0	0	0	1	0	0	0	1	0	1	0	0	0	0	0	0	0	0	0
GVE0448	Bangladesh 2012-2021 (This study)	L23	CFnegative	2021	0	1	1	0	1	1	0	1	1	0	0	0	0	1	1	0	0	0	0	0
GVE0450	Bangladesh 2012-2021 (This study)	L19	CFnegative	2021	1	0	0	0	0	0	0	0	1	0	0	0	0	1	0	1	0	0	1	0
GVE0451	Bangladesh 2012-2021 (This study)	L5	CS5+CS6	2021	0	1	0	0	0	0	0	0	0	0	0	0	0	1	1	0	0	0	0	0
GVE0452	Bangladesh 2012-2021 (This study)	NA	CS6	2021	0	1	1	0	0	0	0	0	0	0	0	0	0	0	0	0	0	0	0	0
GVE0453	Bangladesh 2012-2021 (This study)	L5	CS5+CS6	2020	0	0	0	0	0	0	0	0	0	0	0	0	0	0	0	0	0	0	0	0
GVE0454	Bangladesh 2012-2021 (This study)	NA	CFnegative	2020	0	1	1	0	0	1	0	1	0	0	0	0	0	1	1	0	0	0	0	0
GVE0455	Bangladesh 2012-2021 (This study)	L23	CS26	2021	1	1	1	0	1	0	0	0	1	0	0	0	0	0	0	0	0	0	0	0
GVE0456	Bangladesh 2012-2021 (This study)	L28	CS20	2021	0	1	1	0	0	1	0	1	0	0	0	0	0	1	1	0	0	0	0	0
GVE0466	Bangladesh 2012-2021 (This study)	L16	CFnegative	2020	0	0	0	0	1	0	0	0	1	0	1	0	0	0	0	0	0	0	0	0
GVE0476	Bangladesh 2012-2021 (This study)	L16	CFnegative	2018	0	0	0	0	1	0	0	0	0	0	1	0	0	0	0	0	0	0	0	0
GVE0477	Bangladesh 2012-2021 (This study)	L20	CFnegative	2018	1	1	1	0	0	0	0	0	0	0	0	0	0	1	1	0	0	1	0	0
GVE0480	Bangladesh 2012-2021 (This study)	L25	CFnegative	2018	0	0	0	0	0	0	0	0	1	0	0	0	0	0	0	0	0	0	1	0
GVE0481	Bangladesh 2012-2021 (This study)	L16	CFnegative	2018	0	0	0	0	1	0	0	0	0	0	1	0	0	0	0	0	0	0	0	0
GVE0482	Bangladesh 2012-2021 (This study)	L25	CFnegative	2018	0	0	0	0	0	0	0	0	1	0	0	0	0	0	0	0	0	0	1	0
GVE0483	Bangladesh 2012-2021 (This study)	L27	CFnegative	2018	0	0	0	0	0	0	0	0	0	0	0	0	0	0	0	0	0	0	0	0
GVE0484	Bangladesh 2012-2021 (This study)	L20	CFnegative	2018	1	1	1	0	1	0	0	0	1	0	0	0	0	0	0	0	0	0	0	0
GVE0485	Bangladesh 2012-2021 (This study)	L17	CS6	2018	0	1	1	0	0	1	0	1	0	0	0	0	0	1	1	0	0	0	0	0
GVE0487	Bangladesh 2012-2021 (This study)	L11+L13	CFnegative	2018	1	1	1	0	0	0	0	0	1	0	0	0	0	0	0	0	0	0	0	0
GVE0488	Bangladesh 2012-2021 (This study)	L11+L13	CFnegative	2018	1	1	1	0	0	0	0	0	1	0	0	0	0	0	0	0	0	0	0	0
GVE0489	Bangladesh 2012-2021 (This study)	L4	CS6+CS8	2018	1	1	1	0	0	0	0	0	0	0	0	0	0	1	0	0	0	0	0	1
GVE0491	Bangladesh 2012-2021 (This study)	NA	CS21	2018	1	1	1	0	0	0	0	0	0	0	0	0	0	0	0	0	0	0	0	0
GVE0492	Bangladesh 2012-2021 (This study)	L25	CS23	2021	1	1	1	0	1	0	0	0	1	0	0	1	0	1	0	1	0	0	0	1
GVE0493	Bangladesh 2012-2021 (This study)	NA	CS17	2018	1	1	1	0	0	0	0	0	0	0	0	0	0	0	0	0	0	0	0	0
GVE0494	Bangladesh 2012-2021 (This study)	NA	CS21	2018	0	0	0	0	0	0	0	0	1	0	0	0	0	0	0	1	0	0	0	0
GVE0495	Bangladesh 2012-2021 (This study)	L20	CFnegative	2018	1	1	1	1	1	0	0	0	1	0	0	0	0	0	0	0	0	0	0	0
GVE0498	Bangladesh 2012-2021 (This study)	NA	CS19	2019	0	1	1	0	0	1	0	1	0	0	0	0	0	1	1	0	0	0	0	0
GVE0499	Bangladesh 2012-2021 (This study)	L27	CFnegative	2019	0	0	0	0	0	0	0	0	0	0	0	0	0	0	0	0	0	0	0	0
GVE0500	Bangladesh 2012-2021 (This study)	L5	CS5+CS6	2019	0	0	0	0	0	0	0	0	0	0	0	0	0	0	0	0	0	0	0	0
GVE0501	Bangladesh 2012-2021 (This study)	L3	CS7	2019	1	1	1	0	0	0	0	0	0	0	0	0	0	0	0	0	0	0	0	0
GVE0502	Bangladesh 2022-2023 (This study)	L27	CS23	2023	1	1	0	0	1	0	0	0	0	0	0	0	0	0	0	0	0	0	0	0
GVE0503	Bangladesh 2022-2023 (This study)	L28	CS23	2023	1	1	1	0	0	0	0	0	0	0	0	0	0	0	0	0	0	0	0	0
GVE0504	Bangladesh 2022-2023 (This study)	L28	CS23	2023	1	1	1	0	0	0	0	0	0	0	0	0	0	0	0	0	0	0	0	0
GVE0505	Bangladesh 2012-2021 (This study)	L17	CS6	2019	0	1	1	0	0	1	0	1	0	0	0	0	0	1	1	0	0	0	0	0
GVE0506	Bangladesh 2012-2021 (This study)	L17	CS14	2019	1	1	1	0	0	0	0	0	1	0	0	0	0	0	0	0	0	0	0	0
GVE0507	Bangladesh 2012-2021 (This study)	L5	CS5+CS6	2019	1	1	1	0	0	0	0	0	0	0	0	0	0	0	0	0	0	0	0	0
GVE0508	Bangladesh 2012-2021 (This study)	L20	CFnegative	2019	1	1	1	0	0	0	0	0	0	0	0	0	0	1	1	0	0	1	0	0
GVE0509	Bangladesh 2012-2021 (This study)	L5	CS17	2019	0	0	0	0	0	0	0	0	0	0	0	0	0	0	0	0	0	0	0	0
GVE0543	Bangladesh 2012-2021 (This study)	L25	CFnegative	2017	0	0	0	0	1	0	0	0	1	0	0	0	0	0	0	0	0	0	0	0
GVE0546	Bangladesh 2012-2021 (This study)	L19	CS14	2016	1	1	1	0	0	0	0	0	1	0	0	0	0	0	0	1	0	0	0	0
GVE0549	Bangladesh 2012-2021 (This study)	L1	CS1+CS3+CS21	2016	1	1	1	0	0	0	0	0	0	0	0	0	0	0	0	0	0	0	0	0
GVE0551	Bangladesh 2012-2021 (This study)	L5	CS5+CS6	2017	0	0	0	0	0	0	0	0	0	0	0	0	0	0	0	0	0	0	0	0
GVE0556	Bangladesh 2012-2021 (This study)	L20	CS6	2016	0	1	1	0	0	0	0	0	0	0	0	0	0	0	0	0	0	0	0	0
GVE0562	Bangladesh 2022-2023 (This study)	L19	CS23	2023	0	0	0	0	1	0	0	0	1	0	0	0	0	0	0	0	0	0	0	0
GVE0569	Bangladesh 2012-2021 (This study)	L1	CS1+CS3+CS21	2014	0	0	0	0	0	0	0	0	0	0	0	0	0	0	0	0	0	0	0	0
GVE0588	Bangladesh 2012-2021 (This study)	L18	CS12	2014	1	1	1	0	0	0	0	0	0	0	0	0	0	0	0	0	0	0	0	0
GVE0627	Bangladesh 2022-2023 (This study)	L26	CFnegative	2023	0	1	1	0	0	1	0	1	0	0	0	0	0	1	1	0	0	0	0	0
GVE0628	Bangladesh 2022-2023 (This study)	NA	CFnegative	2023	0	0	0	0	0	0	0	0	0	0	0	0	0	0	0	0	0	0	0	0
GVE0631	Bangladesh 2012-2021 (This study)	L17	CS14	2014	1	1	1	0	0	0	0	0	0	0	0	0	0	0	0	0	0	0	0	0
GVE0632	Bangladesh 2012-2021 (This study)	NA	CS6	2014	1	1	1	0	1	0	0	0	1	0	0	0	0	0	0	0	0	0	0	1
GVE0633	Bangladesh 2012-2021 (This study)	L4	CS21	2014	0	0	0	0	1	0	0	0	0	0	0	0	0	1	1	0	0	0	1	0
GVE0634	Bangladesh 2012-2021 (This study)	L20	CFnegative	2014	1	0	0	0	1	0	0	0	0	0	0	0	0	1	1	0	0	1	0	0
GVE0635	Bangladesh 2012-2021 (This study)	L2	CS2+CS3+CS21	2014	0	0	0	0	0	0	0	0	0	0	0	0	0	0	0	0	0	0	0	0
GVE0636	Bangladesh 2022-2023 (This study)	L20	CFnegative	2023	1	0	0	0	0	0	0	0	1	0	0	0	0	0	0	0	0	0	1	0
GVE0637	Bangladesh 2022-2023 (This study)	L11+L15	CS6	2023	1	1	1	0	0	1	0	1	0	0	0	0	0	1	1	0	0	0	1	0
GVE0638	Bangladesh 2012-2021 (This study)	L19	CFnegative	2017	0	1	1	0	1	0	0	0	1	0	0	0	0	0	0	0	0	0	0	0
GVE0639	Bangladesh 2022-2023 (This study)	L11+L13	CS23	2023	0	0	0	0	1	0	0	0	1	0	0	0	0	0	0	0	0	0	0	0
GVE0640	Bangladesh 2022-2023 (This study)	L28	CS23	2023	1	1	1	0	0	0	0	0	0	0	0	0	0	0	0	0	0	0	0	0
GVE0641	Bangladesh 2012-2021 (This study)	NA	CS6	2014	0	1	1	0	1	0	0	0	1	0	0	0	0	0	0	0	0	0	0	1
GVE0642	Bangladesh 2012-2021 (This study)	L26	CFnegative	2017	0	1	1	0	0	1	0	1	0	0	0	0	0	1	1	0	0	0	0	0
GVE0643	Bangladesh 2022-2023 (This study)	L20	CFnegative	2023	1	1	1	0	0	1	0	1	0	0	0	0	0	1	1	0	0	0	0	0
GVE0644	Bangladesh 2022-2023 (This study)	L2	CS2+CS3+CS21	2023	1	1	1	0	0	0	0	0	0	0	0	0	0	0	0	0	0	0	0	0
GVE0645	Bangladesh 2012-2021 (This study)	L17	CFnegative	2014	1	1	1	0	0	0	0	0	0	0	0	1	0	1	0	1	0	0	1	0
GVE0646	Bangladesh 2012-2021 (This study)	L16	CFnegative	2015	1	1	1	0	0	0	0	0	0	0	1	1	1	1	0	1	0	1	0	1
GVE0647	Bangladesh 2022-2023 (This study)	L27	CS23	2023	1	1	1	0	1	0	0	0	0	0	0	0	0	0	0	0	0	0	0	0
GVE0648	Bangladesh 2022-2023 (This study)	L4	CS6+CS8	2023	1	1	1	0	1	0	0	0	1	0	0	0	0	0	0	0	0	0	0	0
GVE0649	Bangladesh 2012-2021 (This study)	NA	CS6	2015	0	0	0	0	0	0	0	0	0	0	0	0	0	0	0	0	0	0	0	0
GVE0650	Bangladesh 2012-2021 (This study)	L27	CFnegative	2014	0	0	0	0	0	0	0	0	0	0	0	0	0	0	0	0	0	0	0	0
GVE0651	Bangladesh 2012-2021 (This study)	L19	CFnegative	2014	0	0	0	0	1	0	0	0	0	0	0	0	0	0	0	0	0	0	0	0
GVE0653	Bangladesh 2022-2023 (This study)	L7	CS6	2023	1	1	1	0	0	0	0	0	0	0	0	0	0	0	0	0	0	0	0	1
GVE0654	Bangladesh 2022-2023 (This study)	NA	CS20	2023	1	1	1	0	0	0	0	0	0	0	0	0	0	0	0	0	0	0	0	0
GVE0655	Bangladesh 2022-2023 (This study)	NA	CFnegative	2023	1	1	1	0	0	1	0	1	1	0	0	0	0	1	1	0	0	0	1	0
GVE0656	Bangladesh 2022-2023 (This study)	L2	CS2+CS3+CS21	2023	1	1	1	0	0	0	0	0	0	0	0	0	0	0	0	0	0	0	0	0
GVE0657	Bangladesh 2012-2021 (This study)	L17	CS14	2015	1	1	1	0	1	0	0	0	0	0	0	0	0	0	0	0	0	0	0	0
GVE0658	Bangladesh 2012-2021 (This study)	L28	CS20	2015	0	1	1	0	1	1	0	1	1	0	0	0	0	1	0	1	0	0	0	0
GVE0659	Bangladesh 2012-2021 (This study)	L27	CFnegative	2015	1	1	1	0	0	0	0	0	0	0	0	0	0	0	0	0	0	0	0	0
GVE0660	Bangladesh 2012-2021 (This study)	L27	CFnegative	2015	1	1	1	0	0	0	0	0	0	0	0	0	0	0	0	0	0	0	0	0
GVE0661	Bangladesh 2012-2021 (This study)	NA	CFnegative	2015	1	1	1	0	0	1	0	1	0	0	0	0	0	1	1	0	0	0	0	0
GVE0662	Bangladesh 2012-2021 (This study)	L16	CFnegative	2015	1	1	1	0	0	0	0	0	0	0	1	0	0	0	0	0	0	0	0	0

StrainID	Study Dataset	ETEC_Lineage	CFs	Year	ampicillin.TEM	macrolide.mrx	macrolide.mphA.E.G.	macrolide.msrE	ESBL.CTXM	ESBL.DHA1	ESBL.Carbapenum	Fluoroquinolone.QnrB	Fluoroquinolone.QnrS	Fluoroquinolone.gyrB	Fluoroquiolone.parC	strA.APH6	strB.APH3	Trimethoprim.dfr	sulphonamide.sul1	sulphonamide.sul2	sulphonamide.sul3	chloramphenicol.CAT	tetracyclin.tetA	tetracyclin.tetB
GVE0663	Bangladesh 2022-2023 (This study)	L11+L13	CS23	2023	0	0	0	0	1	0	0	0	1	0	0	0	0	0	0	0	0	0	1	0
GVE0664	Bangladesh 2012-2021 (This study)	L20	CFnegative	2014	1	0	0	0	1	0	0	0	0	0	0	0	0	1	1	0	0	1	0	0
GVE0665	Bangladesh 2022-2023 (This study)	L16	CS23	2023	0	0	0	0	1	0	0	0	0	0	1	0	0	0	0	0	0	0	0	0
GVE0666	Bangladesh 2012-2021 (This study)	L28	CS23	2020	0	0	0	0	0	0	0	0	0	0	0	0	0	0	0	0	0	0	0	0
GVE0667	Bangladesh 2012-2021 (This study)	L18	CS6	2015	0	1	1	0	1	0	0	0	1	0	0	0	0	0	0	0	0	0	0	0
GVE0668	Bangladesh 2012-2021 (This study)	L25	CFnegative	2014	0	0	0	0	0	0	0	0	1	0	0	0	0	0	0	0	0	0	1	0
GVE0669	Bangladesh 2012-2021 (This study)	NA	CFnegative	2014	0	1	1	0	0	0	0	0	1	0	0	0	0	1	0	1	0	0	1	0

StrainID	Study Dataset	ETEC_Lineage	CFs	Year	ampicillin.TEM	macrolide.mrx	macrolide.mphA.E.G.	macrolide.msrE	ESBL.CTXM	ESBL.DHA1	ESBL.Carbapenum	Fluoroquinolone.QnrB	Fluoroquinolone.QnrS	Fluoroquinolone.gyrB	Fluoroquiolone.parC	strA.APH6	strB.APH3	Trimethoprim.dfr	sulphonamide.sul1	sulphonamide.sul2	sulphonamide.sul3	chloramphenicol.CAT	tetracyclin.tetA	tetracyclin.tetB
GVE0670	Bangladesh 2012-2021 (This study)	L20	CFnegative	2014	1	0	0	0	1	0	0	0	0	0	0	0	0	1	1	0	0	1	0	0
GVE0696	Bangladesh 2012-2021 (This study)	L5	CS5+CS6	2016	0	0	0	0	0	0	0	0	0	0	0	0	0	0	0	0	0	0	0	0
GVE0697	Bangladesh 2012-2021 (This study)	L19	CS14	2016	1	1	1	0	1	0	0	0	1	0	0	0	0	0	0	0	0	0	0	0
GVE0698	Bangladesh 2012-2021 (This study)	L5	CS17	2017	1	1	1	0	0	0	0	0	0	0	0	0	0	0	0	0	0	0	0	0
GVE0699	Bangladesh 2022-2023 (This study)	L28	CS23	2023	1	1	1	0	0	0	0	0	0	0	0	0	0	0	0	0	0	0	0	0
GVE0700	Bangladesh 2022-2023 (This study)	L28	CS23	2023	1	1	1	0	0	0	0	0	0	0	0	0	0	0	0	0	0	0	0	0
GVE0701	Bangladesh 2022-2023 (This study)	L16	CS23	2023	0	0	0	0	1	0	0	0	0	0	1	0	0	0	0	0	0	0	0	0
GVE0702	Bangladesh 2012-2021 (This study)	L5	CS5+CS6	2017	0	0	0	0	0	0	0	0	0	0	0	0	0	0	0	0	0	0	0	0
GVE0703	Bangladesh 2012-2021 (This study)	L16	CFnegative	2016	0	0	0	0	0	0	0	0	0	0	1	0	0	0	0	0	0	0	0	0
GVE0704	Bangladesh 2012-2021 (This study)	L16	CFnegative	2020	0	0	0	0	1	0	0	0	0	0	1	0	0	0	0	0	0	0	0	0
GVE0705	Bangladesh 2012-2021 (This study)	L5	CS5+CS6	2016	0	0	0	0	0	0	0	0	0	0	0	0	0	0	0	0	0	0	0	0
GVE0706	Bangladesh 2012-2021 (This study)	NA	CS17	2017	1	1	1	0	0	0	0	0	0	0	0	0	0	0	0	0	0	0	0	0
GVE0707	Bangladesh 2022-2023 (This study)	L27	CS23	2023	0	0	0	0	1	0	0	0	0	0	0	0	0	0	0	0	0	0	0	0
GVE0708	Bangladesh 2012-2021 (This study)	L1	CS1+CS3+CS21	2017	0	0	0	0	0	0	0	0	0	0	0	0	0	0	0	0	0	0	0	0
GVE0709	Bangladesh 2022-2023 (This study)	L20	CS23	2023	1	1	1	0	1	0	0	0	0	0	0	0	0	1	0	0	0	1	0	1
GVE0710	Bangladesh 2012-2021 (This study)	L3	CS7	2017	1	1	1	0	0	0	0	0	0	0	0	0	0	0	0	0	0	0	0	0
GVE0711	Bangladesh 2012-2021 (This study)	L17	CS14	2013	0	0	0	0	0	0	0	0	0	0	0	0	0	0	0	0	0	0	0	0
GVE0712	Bangladesh 2012-2021 (This study)	NA	CS2+CS3+CS21	2016	0	1	1	0	0	1	0	1	0	0	0	0	0	1	1	0	0	0	0	1
GVE0713	Bangladesh 2012-2021 (This study)	L18	CS12	2016	0	0	0	0	0	0	0	0	0	0	0	0	0	0	0	0	0	0	0	0
GVE0714	Bangladesh 2012-2021 (This study)	NA	CFnegative	2016	0	1	1	0	0	1	0	1	0	0	0	0	0	1	1	0	0	0	0	0
GVE0715	Bangladesh 2012-2021 (This study)	NA	CS17	2016	0	1	1	0	0	0	0	0	0	0	0	0	0	0	0	0	0	0	0	0
GVE0716	Bangladesh 2012-2021 (This study)	L18	CS12	2016	1	1	1	0	0	0	0	0	0	0	0	0	0	0	0	0	0	0	0	0
GVE0717	Bangladesh 2022-2023 (This study)	L27	CS23	2023	0	1	1	0	1	1	0	1	0	0	0	0	0	1	1	0	0	0	0	0
GVE0718	Bangladesh 2012-2021 (This study)	L1	CS1+CS3+CS21	2013	0	0	0	0	1	0	0	0	1	0	0	0	0	0	0	0	0	0	0	0
GVE0719	Bangladesh 2012-2021 (This study)	NA	CS6	2013	0	0	0	0	0	0	0	0	0	0	0	0	0	0	0	0	0	0	0	0
GVE0720	Bangladesh 2022-2023 (This study)	L26	CS23	2023	1	0	0	0	0	0	0	0	0	0	0	0	0	0	0	0	0	0	0	0
GVE0721	Bangladesh 2012-2021 (This study)	L25	CFnegative	2013	0	0	0	0	0	0	0	0	1	0	0	0	0	0	0	0	0	0	1	0
GVE0722	Bangladesh 2012-2021 (This study)	L4	CS6+CS8	2013	1	0	0	0	0	0	0	0	0	0	0	1	0	1	0	1	0	0	0	0
GVE0723	Bangladesh 2012-2021 (This study)	L27	CFnegative	2013	1	0	0	0	0	0	0	0	0	0	0	0	0	0	0	0	0	0	1	0
GVE0724	Bangladesh 2012-2021 (This study)	L11+L13	CFnegative	2015	0	1	1	0	1	0	0	0	1	0	1	0	0	0	0	0	0	1	0	1
GVE0725	Bangladesh 2012-2021 (This study)	L5	CS5+CS6	2013	1	1	1	0	0	0	0	0	0	0	0	0	0	0	0	0	0	0	0	0
GVE0726	Bangladesh 2012-2021 (This study)	L25	CFnegative	2013	0	0	0	0	0	0	0	0	1	0	0	0	0	0	0	0	0	0	1	0
GVE0727	Bangladesh 2022-2023 (This study)	L11+L13	CS23	2023	1	1	1	0	1	0	0	0	1	0	0	0	0	0	0	0	0	0	0	0
GVE0728	Bangladesh 2012-2021 (This study)	L3	CS7	2013	1	0	0	0	0	0	0	0	0	0	0	1	0	1	0	1	0	0	0	0
GVE0729	Bangladesh 2012-2021 (This study)	L18	CS6	2013	0	0	0	0	1	0	0	0	1	0	0	0	0	1	0	0	0	0	1	0
GVE0730	Bangladesh 2012-2021 (This study)	L27	CFnegative	2013	1	0	0	0	0	0	0	0	0	0	0	0	0	0	0	0	1	0	1	0
GVE0731	Bangladesh 2022-2023 (This study)	L27	CS23	2023	1	0	0	0	1	0	0	0	0	0	0	0	0	0	0	0	0	0	0	0
GVE0736	Bangladesh 2012-2021 (This study)	L5	CS5+CS6	2017	0	0	0	0	0	0	0	0	0	0	0	0	0	0	0	0	0	0	0	0
GVE0737	Bangladesh 2012-2021 (This study)	NA	CS6	2017	1	1	1	0	0	0	0	0	0	0	0	0	0	0	0	0	0	0	0	1
GVE0738	Bangladesh 2012-2021 (This study)	L19	CFnegative	2019	0	0	0	0	0	0	0	0	0	0	0	0	0	0	0	0	0	0	0	0
GVE0739	Bangladesh 2012-2021 (This study)	L1	CS1+CS3+CS21	2019	0	0	0	0	0	0	0	0	0	0	0	0	0	0	0	0	0	0	0	0
GVE0740	Bangladesh 2012-2021 (This study)	L27	CS23	2020	0	0	0	0	1	0	0	0	0	0	0	0	0	0	0	0	0	0	0	0
GVE0741	Bangladesh 2012-2021 (This study)	L25	CS23	2020	0	1	1	1	1	1	0	1	1	0	0	0	0	1	1	1	0	0	1	0
GVE0742	Bangladesh 2012-2021 (This study)	NA	CFnegative	2020	0	1	1	0	0	0	0	0	1	0	0	0	0	1	0	1	0	0	1	0
GVE0743	Bangladesh 2012-2021 (This study)	L27	CS23	2020	0	0	0	0	1	0	0	0	0	0	0	0	0	0	0	0	0	0	0	0
GVE0744	Bangladesh 2012-2021 (This study)	L7	CS6	2021	1	1	1	0	0	0	0	0	0	0	0	0	0	0	0	0	0	0	0	1
GVE0745	Bangladesh 2012-2021 (This study)	L26	CS23	2021	1	0	0	0	1	0	0	0	1	0	0	0	0	0	0	0	0	0	1	0
GVE0746	Bangladesh 2012-2021 (This study)	L19	CS14	2020	1	1	1	0	0	0	0	0	0	0	0	0	0	0	0	0	0	0	0	0
GVE0747	Bangladesh 2022-2023 (This study)	L26	CS23	2023	1	0	0	0	1	0	0	0	1	0	0	0	0	0	0	0	0	0	1	0
GVE0748	Bangladesh 2012-2021 (This study)	L27	CS23	2020	0	0	0	0	1	0	0	0	0	0	0	0	0	0	0	0	0	0	0	0
GVE0749	Bangladesh 2012-2021 (This study)	L19	CS23	2021	0	0	0	0	1	0	0	0	1	0	0	0	0	0	0	0	0	0	0	0
GVE0751	Bangladesh 2012-2021 (This study)	L25	CFnegative	2016	0	0	0	0	0	0	0	0	1	0	0	0	0	0	0	0	0	0	0	0
GVE0752	Bangladesh 2012-2021 (This study)	L16	CFnegative	2015	1	0	0	0	1	0	0	0	1	0	1	0	0	0	0	0	0	0	1	0
GVE0753	Bangladesh 2012-2021 (This study)	L5	CS17	2020	1	1	1	0	0	0	0	0	0	0	0	0	0	0	0	0	0	0	0	0
GVE0754	Bangladesh 2012-2021 (This study)	L16	CFnegative	2015	1	0	0	0	1	0	0	0	1	0	1	0	0	0	0	0	0	0	1	0
GVE0757	Bangladesh 2012-2021 (This study)	L20	CFnegative	2019	1	1	1	0	1	0	0	0	1	0	0	1	0	1	1	1	0	1	1	0
GVE0758	Bangladesh 2012-2021 (This study)	L17	CS14	2019	0	0	0	0	0	0	0	0	0	0	0	0	0	0	0	0	0	0	0	0
GVE0759	Bangladesh 2012-2021 (This study)	L5	CS5+CS6	2019	0	0	0	0	0	0	0	0	0	0	0	0	0	0	0	0	0	0	0	0
GVE0760	Bangladesh 2012-2021 (This study)	L1	CS1+CS3+CS21	2017	0	0	0	0	0	0	0	0	0	0	0	0	0	0	0	0	0	0	0	0
GVE0761	Bangladesh 2012-2021 (This study)	L27	CS23	2021	0	0	0	0	1	0	0	0	0	0	0	0	0	0	0	0	0	0	0	0
GVE0762	Bangladesh 2012-2021 (This study)	L27	CFnegative	2015	0	0	0	0	1	0	0	0	1	0	0	0	0	0	0	0	0	0	0	0
GVE0763	Bangladesh 2012-2021 (This study)	L28	CS23	2020	1	1	1	0	0	0	0	0	0	0	0	0	0	0	0	0	0	0	0	0
GVE0764	Bangladesh 2012-2021 (This study)	L28	CS23	2020	1	1	1	0	0	0	0	0	0	0	0	0	0	0	0	0	0	0	0	0
GVE0770	Bangladesh 2012-2021 (This study)	L25	CFnegative	2012	0	0	0	0	0	0	0	0	1	0	0	0	0	0	0	0	0	0	1	0
GVE0771	Bangladesh 2012-2021 (This study)	L29	CS12	2012	1	1	1	0	1	0	0	0	1	0	0	0	0	0	0	0	0	0	0	0
GVE0772	Bangladesh 2012-2021 (This study)	L25	CFnegative	2012	0	0	0	0	0	0	0	0	1	0	0	0	0	0	0	0	0	0	1	0
GVE0773	Bangladesh 2012-2021 (This study)	L5	CS5+CS6	2012	0	0	0	0	0	0	0	0	0	0	0	0	0	0	0	0	0	0	0	0
GVE0774	Bangladesh 2012-2021 (This study)	L1	CS1+CS3+CS21	2012	1	1	1	0	0	0	0	0	0	0	0	0	0	0	0	0	0	0	0	0
GVE0775	Bangladesh 2012-2021 (This study)	NA	CS17	2012	1	1	1	0	0	0	0	0	0	0	0	0	0	0	0	0	0	0	0	0
GVE0776	Bangladesh 2022-2023 (This study)	L11+L13	CS23	2023	0	0	0	0	1	0	0	0	1	0	0	0	0	0	0	0	0	0	0	0
GVE0777	Bangladesh 2012-2021 (This study)	NA	CFnegative	2012	1	1	1	0	0	1	0	1	0	0	0	0	0	1	1	0	0	0	0	0
GVE0778	Bangladesh 2022-2023 (This study)	L11+L13	CS23	2023	1	0	0	0	0	0	0	0	1	0	0	0	0	0	0	1	0	0	1	0
GVE0779	Bangladesh 2012-2021 (This study)	L1	CS1+CS3+CS21	2012	0	0	0	0	0	0	0	0	0	0	0	0	0	0	0	0	0	0	0	0
GVE0780	Bangladesh 2012-2021 (This study)	L29	CS12	2012	1	1	1	0	0	0	0	0	1	0	0	0	0	0	0	0	0	0	0	0
GVE0781	Bangladesh 2012-2021 (This study)	L20	CFnegative	2012	0	0	0	0	1	0	0	0	1	0	0	0	0	0	0	0	0	0	1	0
GVE0782	Bangladesh 2012-2021 (This study)	NA	CFnegative	2012	1	1	1	0	0	0	0	0	0	0	0	0	0	0	0	0	0	0	0	0
GVE0783	Bangladesh 2012-2021 (This study)	L3	CS7	2012	1	1	1	0	0	0	0	0	0	0	0	0	0	0	0	0	0	0	0	0
GVE0784	Bangladesh 2012-2021 (This study)	L25	CFnegative	2012	0	0	0	0	0	0	0	0	1	0	0	1	0	1	0	0	0	0	1	0
